# A Standardized Vascular Disease Health Check in Europe: A Cost-Effectiveness Analysis

**DOI:** 10.1371/journal.pone.0066454

**Published:** 2013-07-15

**Authors:** C. Andy Schuetz, Peter Alperin, Swathi Guda, Andrew van Herick, Bertrand Cariou, David Eddy, Janusz Gumprecht, Antonio Nicolucci, Peter Schwarz, Nick J. Wareham, Daniel R. Witte, Ulf Smith

**Affiliations:** 1 Archimedes, Inc., San Francisco, California, United States of America; 2 Clinique d'Endocrinologie, l'Institut du Thorax, CHU Nantes, Nantes, France; 3 Department of Internal Diseases, Diabetology and Nephrology, Medical University of Silesia, Zabrze, Poland; 4 Department of Clinical Pharmacology and Epidemiology, Consorzio Mario Negri Sud, Santa Maria Imbaro, Italy; 5 Department of Medicine III Prevention and Care of Diabetes , University of Dresden, Dresden, Germany; 6 Medical Research Council Epidemiology Unit, Institute of Metabolic Science, Addenbrooke's Hospital, Cambridge, United Kingdom; 7 Steno Diabetes Center, Gentofte, Denmark; 8 Sahlgrenska University Hospital, Gothenburg, Sweden; University of Tor Vergata, Italy

## Abstract

**Background:**

No clinical trials have assessed the effects or cost-effectiveness of health check strategies to detect and manage vascular disease. We used a mathematical model to estimate the cost-effectiveness of several health check strategies in six European countries.

**Methods:**

We used country-specific data from Denmark, France, Germany, Italy, Poland, and the United Kingdom to generate simulated populations of individuals aged 40–75 eligible for health checks in those countries (e.g. individuals without a previous diagnosis of diabetes, myocardial infarction, stroke, or serious chronic kidney disease). For each country, we used the Archimedes model to compare seven health check strategies consisting of assessments for diabetes, hypertension, lipids, and smoking. For patients diagnosed with vascular disease, treatment was simulated in a standard manner. We calculated the effects of each strategy on the incidence of type 2 diabetes, major adverse cardiovascular events (MACE), and microvascular complications in addition to quality of life, costs, and cost per quality-adjusted life-year (QALY).

**Results:**

Compared with current care, health checks reduced the incidence of MACE (6–17 events prevented per 1000 people screened) and diabetes related microvasular complications (5–11 events prevented per 1000 people screened), and increased QALYs (31–59 discounted QALYs) over 30 years, in all countries. The cost per QALY of offering a health check to all individuals in the study cohort ranged from €14903 (France) to cost saving (Poland). Pre-screening the population and offering health checks only to higher risk individuals lowered the cost per QALY. Pre-screening on the basis of obesity had a cost per QALY of €10200 (France) or less, and pre-screening with a non-invasive risk score was similar.

**Conclusions:**

A vascular disease health check would likely be cost effective at 30 years in Denmark, France, Germany, Italy, Poland, and the United Kingdom.

## Introduction

Diabetes and cardiovascular disease (collectively referred to as vascular disease) are leading causes of mortality and morbidity throughout the world [1], [2]. Rates of obesity and diabetes are rising at an alarming pace across Europe [3]. Managing vascular disease over the coming decades will require an integrated approach to addressing established modifiable risk-factors. Population level screening should be a central element of any management strategy, because the early stages of vascular disease are often asymptomatic, and many individuals remain undiagnosed until debilitating and costly complications occur [4], [5], [6], [7].

The NHS Health Check program was developed in the United Kingdom (UK) to address this problem [8], [9]. The Health Checks program integrates the prevention, early detection, and treatment of type 2 diabetes, hypertension, dyslipidemia, and smoking. Further, previous modeling studies have indicated that health checks and screening for diabetes are likely to be cost effective [10], [11], [12].

In this study, we used the Archimedes Model to estimate the cost effectiveness of offering a range of health check strategies to individuals in six European populations, compared to current levels of care in each country. Our study provides a broader understanding than prior studies by forecasting the impact of offering health checks that address multiple aspects of vascular disease, recurring every five years, in six European settings.

## Methods

We simulated a clinical trial comparing seven health check strategies to current levels of care in Denmark, France, Germany, Italy, Poland, and the UK. For each country, we forecasted the impact of each strategy on the incidence of major adverse cardiovascular events, and diabetes related microvascular complications, as well as medical costs, and quality adjusted life years (QALYs).

### Mathematical Model

Our estimates were made with the Archimedes Model, a person-specific simulation model designed to capture what happens in real health care systems at a clinically meaningful level of detail. The Model forecasts health outcomes and health care utilization associated with diabetes and its complications, coronary artery disease, congestive heart failure, stroke, hypertension, obesity, metabolic syndrome, and cancers of the breast, lung, and colon. Including all relevant conditions and the health care system in a single integrated model captures interactions between diseases and comorbidities in a physiologically realistic way. Details of the Model pertinent to diabetes and its complications have been described elsewhere [13], [14], [15], and a description of the model structure and data sources used in the modeling is provided as File S2.

In brief, the Model uses person-specific data to generate simulated individuals, each having a unique physiology that evolves continuously over time, and which can begin to function abnormally in the case of disease, potentially causing symptoms, changes in biomarkers, and ultimately health outcomes. The Model includes detailed representations of the health care system, with acute and ambulatory care settings, physicians, medical tests, and so on. The simulated health care system is calibrated to match patterns of health care delivery observed in the target setting (File S3). The data sources used to model the benefits of lifestyle, anti-platelet, anti-diabetic, anti-hypertensive, and lipid lowering therapies are provided as File S2.

The accuracy of the Archimedes Model has been validated through simulations of a large number of epidemiological, clinical, and health service research studies [16]. More than 50 clinical trials in both US and European settings have been used to validate the Model, and a full validation report is provided as File S4 and on the Archimedes website [15]. Further, two validations have been performed prospectively [17], including the CARDS study [18] which enrolled a UK population.

### Procedure

We simulated the effects of offering health checks to six European cohorts. For each country, we used a three step process to generate a study cohort of virtual individuals aged 40 to 75, without a previous diagnosis of diabetes, myocardial infarction, stroke, or serious chronic kidney disease (CKD) (with estimated glomerular filtration rate (eGFR) <60 mL/min/1.73m2). First, we generated a population cross-section of all adults aged 20 to 85, matching the demographics and distributions of risk factors observed in real-world data. This population was generated via biased sampling of simulated individuals, based on real subjects observed in NHANES 1999–2008 [19]. This approach to constructing simulated European cohorts using biased sampling of virtual individuals based on US data has been demonstrated to be predictive in the trial validations cited above. The specific real-world data used to create the simulated populations varied by country, but included biomarker and risk factor distributions, as well as the prevalence and incidence of diseases addressed by the health check. A complete listing of the data used is provided in File S1. Second, the performance of the simulated health care system was calibrated to match levels of care currently being delivered in the country, in terms of the prevalence and incidence of diagnoses, medication use (e.g., anti-hypertensive, statin, and anti-diabetic treatments), and the disease burden in the country (including type 2 diabetes, nephropathy, retinopathy, neuropathy, myocardial infarction, stroke, cardiovascular death, and mortality). These first two steps we repeated iteratively until we obtained a simulated population and health care system reflective of the target country. Lastly, the study cohort – individuals eligible for the health check – was extracted from the population cross-section using the eligibility criteria stated above. This three-step process was repeated for each country, yielding six study cohorts.

For each country, we simulated a multi-armed clinical trial comparing the health check strategies to a control scenario in which no health checks were offered (see Table 1 for a list of the strategies). The control scenario was reflective of current care in each country. The health check frequency was every five years, but ceasing at age 70 or upon diagnosis of diabetes, myocardial infarction, stroke, or chronic kidney disease. In the base-case health check, all individuals received assessments for diabetes, hypertension, lipids, and smoking. Consistent with current guidelines, diabetes risk was assessed with an HbA1c test [20], [21]. Individuals confirmed by two tests to have HbA1c of 6.5% or higher were diagnosed with type 2 diabetes, and referred to a diabetes management protocol [22]. Individuals with HbA1c above 6.0% but less than 6.5% were offered intensive lifestyle advice. Hypertensive individuals (blood pressure above 140/90 mmHg) were referred to a management protocol based on the JNC7 guideline [23]. Individuals classified as high risk according to the ATP-III guideline [24], [25] and with LDL cholesterol greater than 5.59mmol/L were referred for statin therapy. All smokers were offered a smoking cessation intervention.

**Table 1:** Simulated health check strategies and control.

	Eligibility criteria for risk assessments, for individuals offered a health check		
Strategy	Diabetes risk assessment*	Lipid risk assessment	Lifestyle Interventions†
**Control – No health checks offered**	None	None	None
**Health check – base-case**	All	All	All
**Health check without lifestyle interventions**	All	All	None
**Health check with gated HbA1c test**	BMI ≥ 30 or BP ≥ 140/90	All	All
**Pre-screening scenarios targeting patients meeting the following additional criteria**			
** Age 50 years or greater**	All	All	All
** BMI 30 kg/m^2^ or greater**	All	All	All
** Above median of diabetes and CVD risk**	All	All	All
** Top quartile of diabetes and CVD risk**	All	All	All

In all cases, individuals had to be aged 40–75 and not have a previous diagnosis of diabetes, myocardial infarction, stroke, or serious chronic kidney disease to be eligible for a health check. In the pre-screening strategies, individuals also had to meet the additional criteria listed in the table. Eligible individuals received the health check at a 5 year interval in all strategies considered.

* Eligible individuals were given an HbA1c test.

† Diet and exercise for individuals with 6.0% ≤ HbA1c <6.0%, and smoking cessation interventions for smokers.

We examined providing a health check that only included an HbA1c test for obese (BMI ≥30 kg/m^2^) and/or hypertensive individuals, reflective of the strategy used by the NHS Health Checks program. We also examined the effects of offering a health check without lifestyle interventions (omitting intensive lifestyle advice and smoking cessation) to the entire study cohort to gauge the impact of the interventions on the results, since the benefits of lifestyle interventions are more difficult to quantify than pharmacological interventions.

In consideration of total budget impact, four strategies examined various ways to pre-screen the study cohort, and restrict eligibility for the health check to individuals with elevated risk of vascular disease (also shown in Table 1).

To explore how pre-screening the population with a non-invasive risk test might make the health check more efficient, we created a “virtual”, generic risk test based on simulated data. This generic risk test had the independent risk factors of age, gender, BMI, waist circumference, smoking, family history of diabetes, family history of coronary heart disease, and anti-hypertensive usage. The generic risk test had a logistic functional form, and was based on a logistic regression performed on the presence of undiagnosed diabetes at baseline or the ten-year occurrence of myocardial infarction, stroke, or CV death, in half of the simulated Danish population cross-section of adults aged 20 to 85. Two thresholds were considered for the risk test: individuals in the top quartile of risk, and individuals above median risk, as predicted by the logistic score. The performance of the generic risk test in detecting undiagnosed diabetes at baseline or the ten-year occurrence of myocardial infarction, stroke, or CV death was then tested on the remaining half of the simulated Danish population cross-section, as well as the population cross-sections of the remaining five countries, and the results are shown in Table 2. Further details on the creation and validation of the generic risk test are provided in File S1. Our use of this virtual, generic diabetes and CVD risk test is intended to show what might be achieved with risk test based pre-screening strategies. A real-world program would use a real-world risk test, with similar sensitivity and specificity, evaluated on data provided by the individual (like FINDRISC [26]), or from data in general practice databases (like the Cambridge diabetes risk score [27]).

**Table 2:** Performance of the generic risk score in detecting undiagnosed type 2 diabetes at baseline or the occurrence of CVD at ten years, for individuals with estimated risk in the top quartile (25%) and top half (50%) of the population ranked by risk score.

	Denmark		France		Germany		Italy		Poland		UK	
	25%	50%	25%	50%	25%	50%	25%	50%	25%	50%	25%	50%
Positive Predictive Value	0·206	0·136	0·189	0·131	0·272	0·189	0·194	0·141	0·199	0·138	0·260	0·177
Negative Predictive Value	0·965	0·981	0·963	0·981	0·946	0·971	0·952	0·971	0·961	0·980	0·952	0·976
Sensitivity	0·664	0·879	0·628	0·871	0·625	0·868	0·573	0·831	0·631	0·875	0·646	0·880
Specificity	0·785	0·532	0·781	0·530	0·796	0·545	0·780	0·531	0·783	0·532	0·794	0·543
Likelihood Ratio	3·084	1·877	2·866	1·854	3·061	1·906	2·606	1·772	2·902	1·870	3·142	1·923

Based on expert opinion, we assumed that 50% of patients would adhere to treatments triggered by the health check. We conservatively assumed that patients who had previously not adhered to prescribed treatments would remain non-adherent to care prescribed by the health check. For intensive lifestyle advice, we assumed that 50% of patients referred would maintain a 3% weight loss for life, based on trials of commercial programs [28], [29]. We assumed that 10.5% of smokers who received smoking cessation interventions would successfully quit, and that the cost would be £224 per successful quitter, based on studies from the UK [30], [31], rescaled according to the relative cost of care in each country. Finally, we did not attribute a cost to the evaluation of the generic risk test because the cost is currently unknown. The cost of the test will vary depending on the implementation chosen (e.g. the generic risk test might be performed through mailed forms, web-based forms, or general practice database analyses).

### Study outcomes and statistical analyses

Simulated individuals were followed for 30 years or until death. We tracked the effect of health checks on the incidence of diagnoses of type 2 diabetes, major adverse cardiovascular events (MACE, first occurrence of myocardial infarction, stroke, or cardiovascular death), and a composite of serious microvascular complications (first occurrence of diabetes related blindness, CKD, end stage renal disease, renal death, foot ulcer, or foot amputation). We computed the number needed to screen (NNS) in order to prevent one additional event using the Kaplan­Meier survival curves [32].

Direct medical costs were considered from a governmental payer perspective, adjusted to 2011 amounts, and reported in euros. Country-specific data on medical test, treatment, and health care delivery costs were used to estimate the direct medical costs associated with vascular disease in each country. In instances where specific data were unavailable, we used rescaled Medicare costs. We calculated QALYs based on the time individuals spent with different disorders using published disutilities [33], [34]. Table 3 shows the disutilities, and Table 4 shows the costs of visits, tests, and treatments used. Costs and QALYs were discounted at an annual rate of 3%. Variations and uncertainty about costs, disutilities, and discount rates were studied through sensitivity analyses. Outcomes were considered significant for p<0·05.

**Table 3:** Base-case model input assumptions for quality of life disutilities.

Quality of life disutilities		
Health State	Disutility	Sources
Angina	−0·0412	Sullivan^29^
Myocardial infarction	−0·0409	Sullivan^29^
Stroke	−0·0460	Sullivan^29^
End stage renal disease	−0.0780	Coffey^30^
Blind in one eye	−0.0430	Coffey^30^
Blind in two eyes	−0.1700	Coffey^30^
Foot ulcer	−0·0990	Coffey^30^
Foot amputation	−0.1050	Coffey^30^
Multiple chronic conditions		
2	−0·0942	Sullivan^29^
3	−0·0876	Sullivan^29^
4	−0·0711	Sullivan^29^
5	−0·0547	Sullivan^29^
6	−0·0419	Sullivan^29^
7	−0·0350	Sullivan^29^
8	−0·0344	Sullivan^29^
9	0·0026	Sullivan^29^
10	0·0097	Sullivan^29^

**Table 4:** Base-case model input assumptions for costs, in euros.

Costs (€)	Denmark	France	Germany	Italy	Poland	UK
Outpatient visit*	99.71	23.00	34.77	22.85	6.25	35.48
Blood pressure measurement	0	0	0	0	0	0
HbA1c test	14.52	14.40	21.30	12.66	4.97	11.65
Lipid panel	15.74	14.60	21.60	25.55	5.04	11.81
Treatment costs (per day, unless indicated otherwise)						
Intensive lifestyle advice	0.42	1.09	0.37	1.18	0.14	0.18
Smoking cessation (cost per quitter)	344.04	319.07	472.00	558.45	110.25	248.10
ACE-inhibitor	0.03	0.42	0.14	0.34	0.01	0.05
Thiazide diuretics	0.06	0.10	0.16	0.87	0.02	0.03
Calcium channel blocker	0.08	0.96	0.11	0.20	0.02	0.04
Beta blocker	0.13	0.53	0.07	0.12	0.01	0.03
Metformin	0.16	0.36	0.26	0.12	0.04	0.03
Sulfonylurea	0.24	0.28	0.07	0.09	0.00	0.05
Glitazone	1.91	2.63	2.11	1.39	0.34	1.42
Insulin	0.56	2.42	1.30	0.89	0.66	0.68
Statin	0.19	1.08	0.48	1.03	0.20	0.26

* Cost of outpatient visit included BMI and smoking assessment.

## Results

The baseline characteristics of the six simulated study cohorts are reported in Table 5. The rates of type 2 diabetes, MACE, and microvascular complications in the control (e.g. current care with no health checks) are shown in Table 6. At 30 years, all of the health check scenarios reduced the incidence MACE and microvascular complications relative to control (p<0·0001), as shown in Table 7.

**Table 5:** Baseline characteristics of the individuals eligible for the health check.

Characteristic	Denmark	France	Germany	Italy	Poland	UK
N	25000	24730	25000	25000	25000	24999
Age	53.3	54.3	54.5	54.6	54.6	54.2
Male sex	0.47	0.49	0.46	0.48	0.48	0.47
Blood Pressure (mmHg)						
Systolic	127	127	136	137	140	131
Diastolic	76	79	84	83	80	77
Total cholesterol (mmol/l)	5.46	5.66	5.66	5.59	5.53	5.66
HDL (mmol/l)	1.47	1.45	1.60	1.42	1.50	1.47
LDL (mmol/l)	3.28	3.52	3.54	3.47	3.34	3.44
Triglycerides (mmol/l)	1.52	1.55	1.15	1.54	1.57	1.59
HbA1c (%)	5.30	5.24	5.28	5.24	5.20	5.30
BMI (kg/m^2^)	25.9	25.9	26.9	26.9	27.0	27.3
Current smoker	0.21	0.28	0.24	0.24	0.25	0.21
Diagnoses*						
High-risk dyslipidemia †	0.07	0.09	0.05	0.08	0.09	0.07
Hypertension	0.26	0.26	0.28	0.22	0.34	0.26
Medication Use						
Anti-hypertensive	0.18	0.22	0.12	0.16	0.24	0.16
Statin	0.04	0.05	0.03	0.06	0.06	0.02
Individuals meeting pre-screening criteria						
Age ≥ 50 years	58%	62%	61%	61%	66%	60%
BMI 30 kg/m2 or greater	11%	17%	21%	21%	26%	23%
Top quartile of risk	25%	25%	25%	25%	25%	25%
Above median of risk	50%	50%	50%	50%	50%	50%

Eligibility criteria were ages 40 to 75 years and no prior diagnosis of vascular disease.

* The baseline prevalence of MI, stroke, diabetes, stage 3 CKD or higher, and ESRD was zero because of the inclusion/exclusion criteria.

† High-risk according to the ATP-III guideline.^21–22^

**Table 6:** Expected number of events in the control per 1000 individuals screened after 30 years of follow-up, by participant subgroup.

		Participants identified in pre-screening strategies			
Country/Diagnosis	All participants (Base-case)	Age ≥ 50 years	BMI ≥30 kg/m^2^	Above median risk	Top quartile of risk
**Denmark**					
** Diabetes** *	113.8 (94.2–133.5)	110.3 (90.9–129.8)	366.9 (337.0–396.8)	165.7 (142.6–188.7)	207.7 (182.5–232.8)
** MACE** †	264.0 (236.7–291.3)	311.8 (283.1–340.5)	379.6 (349.6–409.7)	338.7 (309.4–368.1)	376.5 (346.5–406.5)
** Microvascular Composite** ‡	209.6 (184.4–234.9)	253.4 (226.4–280.3)	274.9 (247.2–302.6)	235.8 (209.5–262.1)	266.9 (239.5–294.3)
**France**					
** Diabetes**	88.5 (70.9–106.1)	84.0 (66.8–101.2)	263.2 (235.9–290.5)	130.9 (110.0–151.9)	164.6 (141.6–187.5)
** MACE**	232.1 (206.0–258.3)	263.1 (235.9–290.4)	287.6 (259.6–315.7)	280.5 (252.6–308.3)	303.0 (274.6–331.5)
** Microvascular Composite**	178.8 (155.0–202.5)	210.1 (184.9–235.4)	150.1 (128.0–172.2)	184.9 (160.8–208.9)	195.4 (170.8–219.9)
**Italy**					
** Diabetes**	127.1 (106.4–147.7)	112.4 (92.8–131.9)	312.7 (284.0–341.4)	175.8 (152.2–199.4)	221.8 (196.1–247.6)
** MACE**	286.8 (258.8–314.9)	321.6 (292.6–350.5)	324.9 (295.9–353.9)	343.0 (313.6–372.5)	362.1 (332.3–391.9)
** Microvascular Composite**	195.4 (170.9–220.0)	228.6 (202.6–254.6)	170.5 (147.2–193.9)	213.0 (187.6–238.3)	224.6 (198.8–250.5)
**Germany**					
** Diabetes**	155.5 (133.1–178.0)	132.8 (111.8–153.8)	383.2 (353.1–413.4)	228.0 (202.0–254.0)	265.3 (237.9–292.6)
** MACE**	329.3 (300.2–358.4)	371.3 (341.4–401.3)	417.3 (386.7–447.9)	407.0 (376.5–437.4)	440.0 (409.2–470.8)
** Microvascular Composite**	229.8 (203.7–255.8)	263.5 (236.2–290.8)	247.9 (221.2–274.7)	263.0 (235.7–290.2)	287.0 (259.0–315.1)
**Poland**					
** Diabetes**	143.3 (121.5–165.0)	126.0 (105.4–146.6)	336.9 (307.6–366.2)	209.9 (184.7–235.2)	261.8 (234.6–289.0)
** MACE**	315.8 (287.0–344.7)	351.6 (322.0–381.2)	347.3 (317.8–376.8)	378.2 (348.2–408.3)	397.0 (366.6–427.3)
** Microvascular Composite**	212.0 (186.7–237.3)	237.1 (210.7–263.5)	182.6 (158.7–206.6)	224.9 (199.0–250.8)	240.3 (213.8–266.8)
**UK**					
** Diabetes**	115.1 (95.3–134.9)	105.8 (86.7–124.8)	277.1 (249.4–304.8)	167.0 (143.9–190.2)	204.2 (179.2–229.1)
** MACE**	309.8 (281.1–338.4)	349.8 (320.3–379.4)	390.4 (360.2–420.7)	377.4 (347.3–407.4)	410.6 (380.1–441.1)
** Microvascular Composite**	197.6 (172.9–222.2)	232.3 (206.1–258.4)	210.1 (184.9–235.4)	222.0 (196.2–247.8)	245.1 (218.5–271.8)

* Diagnosis of type 2 diabetes.

† MACE is a composite of the first occurrence of: MI, stroke, or CV death.

‡ Microvascular composite outcome is the first occurrence of blindness, CKD or higher, ESRD, renal death, foot ulcer, or amputation.

**Table 7:** Expected number of events prevented by each screening strategy compared with control, per 1000 individuals screened, after 30 years of follow-up.

		Events Averted (95% CI)		Number Needed to Screen	
Country	Health Check Strategy	MACE*	Microvascular Composite†	MACE	Microvascular Composite
**Denmark**	Base-case	11.0 (9.6–12.4)	8.8 (7.5–10.1)	72	87
	Gated HbA1c test	9.0 (7.7–10.2)	6.8 (5.7–8.0)	89	111
	Without lifestyle	8.9 (7.7–10.1)	5.8 (4.7–6.9)	91	134
	Pre-screening:				
	Age ≥ 50 years	10.8 (8.9–12.6)	9.0 (7.3–10.8)	75	78
	BMI ≥30 kg/m^2^	28.4 (21.8–35.0)	27.3 (20.6–33.9)	30	28
	Above median risk	15.4 (13.1–17.7)	12.5 (10.3–14.7)	48	55
	Top quartile of risk	21.0 (17.3–24.6)	13.3 (10.1–16.5)	32	55
**France**	Base-case	6.1 (4.9–7.2)	5.2 (4.2–6.2)	132	143
	Gated HbA1c test	5.6 (4.5–6.7)	4.4 (3.5–5.3)	140	175
	Without lifestyle	5.3 (4.3–6.3)	3.4 (2.6–4.2)	153	221
	PS: Age ≥ 50 years	6.2 (4.7–7.7)	5.8 (4.5–7.2)	126	118
	PS: BMI ≥30 kg/m^2^	11.8 (8.0–15.6)	11.1 (7.6–14.6)	69	69
	PS: Above median risk	8.2 (6.3–10.0)	7.4 (5.7–9.0)	90	92
	PS: Top quartile of risk	9.4 (6.5–12.4)	9.1 (6.5–11.8)	81	72
**Italy**	Base-case	14.8 (13.2–16.5)	9.2 (7.9–10.5)	51	76
	Gated HbA1c test	14.2 (12.6–15.8)	8.6 (7.3–9.8)	54	82
	Without lifestyle	13.0 (11.5–14.5)	7.4 (6.2–8.5)	59	95
	PS: Age ≥ 50 years	14.3 (12.3–16.4)	9.3 (7.6–11.0)	49	67
	PS: BMI ≥30 kg/m^2^	23.5 (19.1–27.9)	16.0 (12.4–19.7)	33	44
	PS: Above median risk	16.8 (14.3–19.3)	11.4 (9.3–13.4)	40	53
	PS: Top quartile of risk	17.3 (13.7–20.9)	11.5 (8.6–14.4)	35	46
**Germany**	Base-case	17.8 (16.1–19.5)	11.8 (10.3–13.2)	44	60
	Gated HbA1c test	17.2 (15.5–18.9)	10.7 (9.3–12.1)	46	66
	Without lifestyle	16.2 (14.5–17.8)	9.0 (7.7–10.2)	49	78
	PS: Age ≥ 50 years	17.4 (15.2–19.6)	9.8 (8.1–11.5)	41	68
	PS: BMI ≥30 kg/m^2^	34.3 (29.2–39.3)	26.5 (21.9–31.0)	23	26
	PS: Above median risk	24.3 (21.5–27.1)	14.7 (12.4–17.0)	28	41
	PS: Top quartile of risk	26.9 (22.7–31.0)	16.6 (13.1–20.2)	24	31
**Poland**	Base-case	12.6 (11.1–14.1)	7.6 (6.4–8.8)	62	98
	Gated HbA1c test	12.4 (10.9–13.9)	7.3 (6.1–8.5)	62	101
	Without lifestyle	10.6 (9.2–11.9)	5.8 (4.7–6.8)	74	130
	PS: Age ≥ 50 years	12.6 (10.7–14.4)	7.8 (6.3–9.3)	58	86
	PS: BMI ≥30 kg/m^2^	15.4 (11.9–18.8)	12.5 (9.4–15.5)	50	59
	PS: Above median risk	15.1 (12.8–17.4)	11.0 (9.0–13.1)	47	58
	PS: Top quartile of risk	13.4 (10.1–16.7)	14.1 (10.8–17.3)	52	42
**UK**	Base-case	13.2 (11.7–14.7)	9.6 (8.3–11.0)	59	74
	Gated HbA1c test	11.9 (10.5–13.3)	7.5 (6.3–8.7)	65	97
	Without lifestyle	11.3 (9.9–12.6)	7.1 (5.9–8.3)	68	99
	PS: Age ≥ 50 years	13.2 (11.3–15.1)	9.7 (7.9–11.5)	55	66
	PS: BMI ≥30 kg/m^2^	24.7 (20.4–28.9)	20.8 (16.6–25.0)	34	35
	PS: Above median risk	17.8 (15.4–20.2)	12.6 (10.4–14.9)	40	49
	PS: Top quartile of risk	20.8 (17.1–24.5)	15.0 (11.5–18.5)	34	39

The number needed to screen to prevent one event at 30 years is also listed. In all strategies, the number of events averted was significant, with p<0·0001. See Table 1 for definitions of the screening strategies.

* MACE is a composite of the first occurrence of: MI, stroke, or CV death.

† Microvascular composite outcome is the first occurrence of blindness, CKD or higher, ESRD, renal death, foot ulcer, or amputation.

PS  =  Pre-screening.

**Figure 1:**
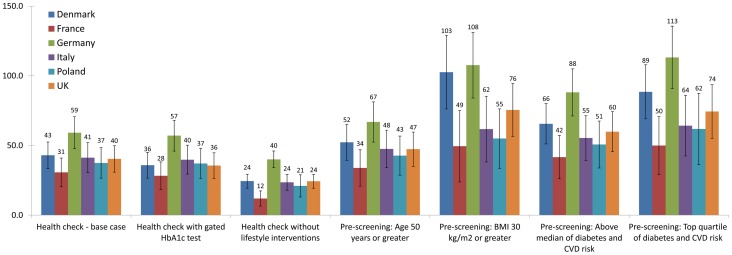
QALYs gained at 30 years per 1000 individuals offered a health check. PS  =  Pre-screening.

**Figure 2:**
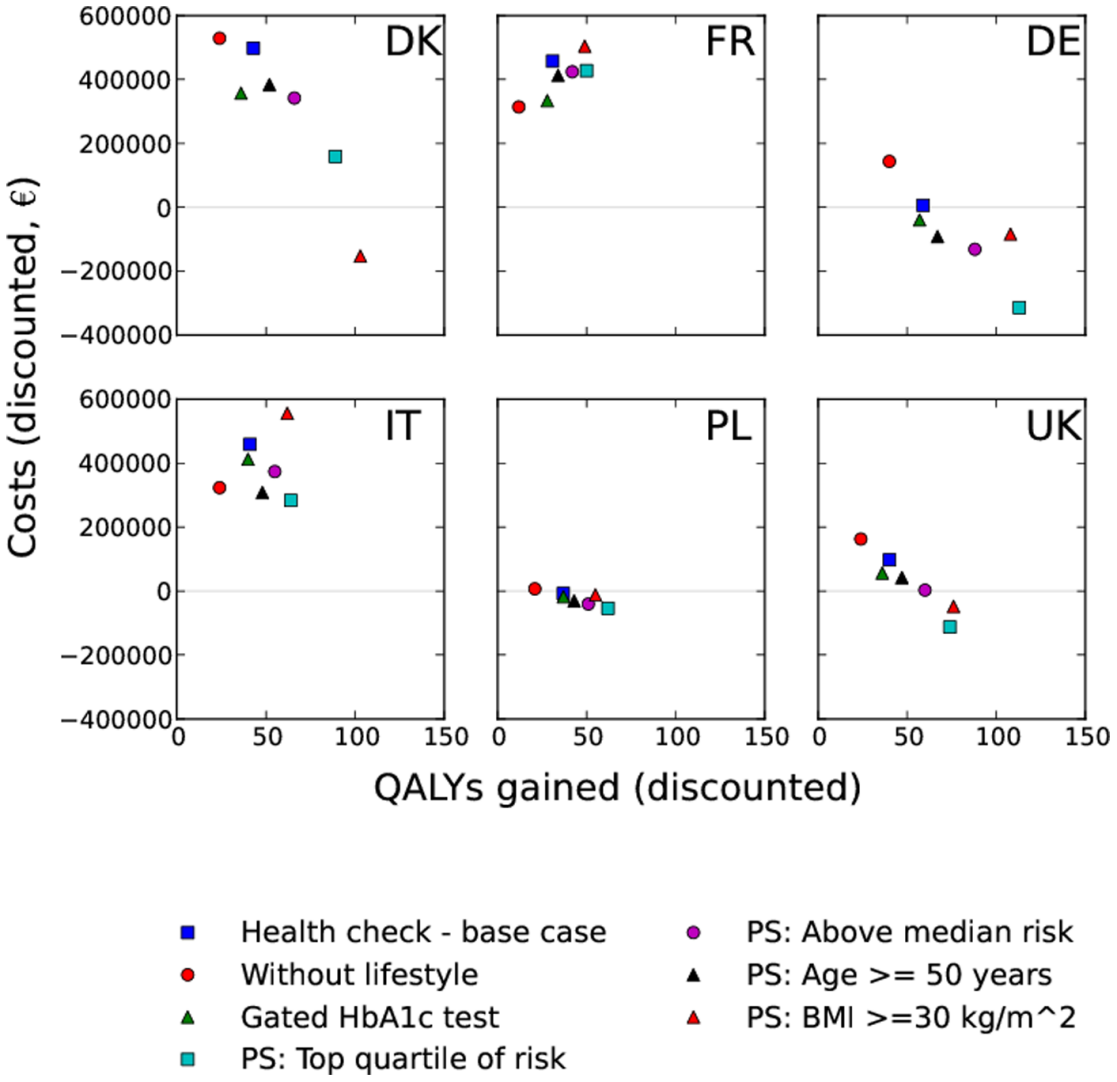
Total medical costs versus QALYs gained at 30 years (discounted) per 1000 individuals screened.

The base-case health check added significant QALYs in all countries at 30 years (Figure 1, p<0·0001). The gated HbA1c test health check strategy provided a similar QALY gain to the base-case, while the strategy omitting lifestyle interventions provided substantially fewer QALYs. Each of the pre-screening scenarios provided a greater QALY gain than the base-case on a per-screened individual basis. Plots of discounted total medical costs vs. QALYs gained for each country are shown in Figure 2. The discounted cost per QALY for the strategies at 30 years, in each country, is shown in Table 8.

**Table 8:** Base-case estimates for the cost per quality-adjusted life-year (QALY) gained by offering health checks (discounted), compared with control after 30 years of follow-up.

	Denmark	France	Germany	Italy	Poland	UK
Base-case health check	11595	14903	115	11113	Cost saving	2426
Without lifestyle	21694	26323	3593	13733	326	6684
Gated HbA1c test	9981	11825	Cost saving	10344	Cost saving	1577
PS: Age ≥ 50 years	7350	12194	Cost saving	6482	Cost saving	887
PS: BMI ≥30 kg/m^2^	Cost saving	10200	Cost saving	9001	Cost saving	Cost saving
PS: Top quartile of risk	1800	8549	Cost saving	4413	Cost saving	Cost saving
PS: Above median risk	5214	10180	Cost saving	6752	Cost saving	48

Costs are reported in euros.

PS  =  Pre-screening.

Table 9 shows the sensitivity of the cost per QALY estimates to variations in key assumptions. Across the sensitivity analyses, our results were fairly insensitive to variations in the assumptions. The cost per QALY was most sensitive to the addition of a disutility associated with the diagnosis of type 2 diabetes, which caused the cost per QALY to increase by 124% in UK and 71% in Denmark. A primary effect of the health check is to diagnose more individuals living with undiagnosed diabetes, so it follows that the QALYs gained from health checks would be sensitive to assumptions about the quality of life with diagnosed vs. undiagnosed diabetes.

**Table 9:** Sensitivity of cost per quality-adjusted life-year to different assumptions about quality of life with diagnosed diabetes, health check effectiveness, costs of screening, treatment costs, discount rates, and time horizon.

	Denmark	France	Germany	Italy	Poland	UK
Reference Health check – base-case, 30 years	11595	14903	115	11113	Cost Saving	2426
**Assumptions**						
Including disutility associated with diabetes diagnosis*	19778	20760	158	13226	Cost Saving	5425
Effectiveness of the health check −20%†	14030	16223	1086	12184	124	3525
Costs of screening +20%	13546	15951	906	11984	49	3332
Costs of screening −20%	9644	13856	Cost Saving	10241	Cost Saving	1520
Treatment costs +20%	11972	17309	910	13956	Cost Saving	2602
Treatment costs −20%	11217	12497	Cost Saving	8271	Cost Saving	2251
Discount rate 5%	15694	17978	1815	14697	343	1592
Discount rate 1%	8337	12353	Cost Saving	8218	Cost Saving	849
**Time horizon**						
10 years	126912	67432	36665	107144	12552	49731
20 years	27369	26148	6330	26930	1559	10407
40 years	7582	10641	Cost Saving	7197	Cost Saving	829

* The disutility for type 2 diabetes was assumed to be −0·0351 [33].

† Scenario in which the health check screening costs are the same, but adherence to the interventions offered in follow-up is reduced by 20%.

## Discussion

Our study shows that offering health checks would likely reduce the 30-year incidence of both MACE and serious microvascular complications. Under the most comprehensive strategy (base-case), the number needed to screen (NNS) to prevent one instance of MACE ranged from 44 in Germany to 132 in France, and the figures were similar for microvascular complications. The NNS values were lower in the higher risk populations identified by pre-screening, corresponding to the higher control scenario event rates.

The 30-year QALY benefit of the base-case was smallest in France (31 QALYs gained per 1000 people), and largest in Germany (59 QALYs gained). The base-case health check had a cost per QALY of €14903 (in France) or less (cost saving in Poland). Variations in cost effectiveness across the countries were driven primarily by the burden of disease (lowest in France and highest in Germany, as shown in Table 6), and the costs of treatments and medical care. For example, Denmark had the second highest base-case cost per QALY in spite of a relatively high disease burden and low treatment costs, largely due to a high outpatient visit cost (as evidenced by the sensitivity to screening costs). Poland had high disease burden, and low screening and treatments costs, rendering the base-case cost saving at 30 years. While the cost per QALY does vary country-to-country, the cost effectiveness conclusions are consistent in that the base-case health check would be cost-effective in all six countries examined.

Offering all individuals a health check, but restricting diabetes risk assessments to obese and/or hypertensive individuals reduced the cost per QALY by €3079 (in France) or less. Such a strategy may have a larger impact on costs if the more expensive OGTT test were used to assess diabetes risk, as in the NHS Health Check in the UK. However, in the present study the lipid assessment required a blood draw for each individual receiving the health check, and thus the incremental cost of an HbA1c test was modest. This result underscores the advantages of using HbA1c for diabetes risk screening, especially in situations where multiple tests can be performed on a single blood draw.

The opportunity for the health check to address modifiable risk factors through lifestyle measures is clear from our study cohort. At baseline, between 28% (France) and 21% (Denmark and UK) of the health check populations were smokers, and the obesity rate ranged from 26% in Poland to 11% in Denmark. Including lifestyle interventions reduced the cost per QALY by as much as 11420 €/QALY in France, as evidenced by comparing the base-case to the strategy without lifestyle interventions. We modeled intensive lifestyle advice and smoking cessation based on commercial and national programs in the UK. Therefore, our findings underscore the importance of prioritizing lifestyle and prevention measures, while recognizing the challenge they present for patients.

The pre-screening strategies explore ways that policy makers could reduce the total budget impact of launching a health check program across Europe. Of the pre-screening strategies considered, starting health checks at age 50, rather than 40, provided the least health benefit, per person screened (shown in Figure 1). Pre-screening on the basis of obesity had much greater benefit, adding between 49 (in France) and 108 QALYs (in Germany), per 1000 people at 30 years. All four pre-screening strategies had a lower Cost/QALY than the base-case, with the obesity-based prescreening having a Cost/QALY ranging from €10200 (France) to cost saving in Denmark, Germany, Poland, and the UK. Risk test based pre-screening provided similar benefit. Comparing the control scenario rates of diabetes diagnosis and MACE (Table 6) shows that obesity based pre-screening more strongly selected for diabetes, and our virtual risk test more strongly selected for MACE. Correspondingly, the obesity-based prescreening had a higher Cost/QALY in countries with higher anti-diabetic treatment costs (France and Italy) and lower Cost/QALY in countries with low treatment costs and higher disease burden (Germany, Poland).

Our study is unique in its European scope, exploring the health benefits and costs of seven health check strategies to six distinct European populations, while a prior analysis examined the health check in a UK setting only [10]. Our examination of a multifaceted health check intervention represents a more integrated approach to addressing vascular disease than prior studies of screening for a single disorder [11], [12]. Our modeling approach also allowed us to examine the benefit a health check recurring every five years, whereas prior studies considered a one-time screenings [12]. This study provides health care decision makers with a realistic pan-European view of strategies for managing vascular disease that are likely to be cost-effective over the next three decades.

These results must be considered along with the limitations of the analysis. This study used the Archimedes Model to forecast outcomes and costs over 30 years. While the Model has been extensively validated on US and European trials, the present analysis has forecasted outcomes in settings for which no direct validations have yet been performed. To mitigate the uncertainty, we used detailed country-specific data to model the disease burden, and health care system performance in each population considered. However, practical limitations in the available data restricted our ability to confirm the predictions of the Model. For example while we had access to multiple estimates for the prevalence and incidence of almost all health outcomes related to the health check in Denmark, for the UK we only had disease prevalence data and no incidence figures. It is possible that unknown limitations or biases in the available cost data for each country may account for some of the country-to-country variations we observed. To simplify the analysis, we also assumed that medical guidelines across the six countries examined were the same, and focused on capturing country-specific risk factor levels, disease burdens, and health care system performance levels. While there are substantive differences between guidelines in the six countries examined, our assumptions applied equally to all strategies examined and the control, and so should minimally impact the findings. We calibrated each country's simulated health care system to match the standard of care reported in country specific data by matching metrics such as biomarker values, rates of disease diagnoses, and medication use (as summarized in File S1). The observed real-world level of care fell short of the guidelines in all countries considered, and we captured these gaps in our analysis. Thus, the population and health care system calibrations reduced the impact of our approximation of each country's guidelines on the analysis, as the simulated individuals received imperfect care (as opposed to perfect guideline care), which yielded more pragmatic cost-effectiveness estimates. The example of HbA1c management for individuals with type 2 diabetes, and our analysis of Denmark shows how the impact of approximating regional guidelines was reduced via model calibration. For all countries, we assumed a management goal of HbA1c <7% for patients with type 2 diabetes. The actual guidelines in the six countries range from <6.5% to <7%, and Denmark follows a guideline of HbA1c <6.5% for most patients (<7.5% for patients with cardiovascular disease)[35]. However, at a population level Danish patients with type 2 diabetes are not uniformly controlled to the guideline. Examining the real-world evidence and our model of Demark, Tables S1 and S2 in File S1 show that our model matched the adult population mean HbA1c, the prevalence of diagnosed type 2 diabetes among adults stratified by gender and age, the annual incidence of type 2 diabetes, the mean HbA1c of individuals with type 2 diabetes stratified by age, and the prevalence of patients taking oral diabetic agents and insulin. Further, the comparison of the simulated population to the real-world evidence shows agreement in mean HbA1c among diabetic patients spanning the following age bins 20–40, 40–60 years, 60–80, and 20–85 years. Thus, by capturing risk factors (such as population level mean HbA1c), diagnosis prevalence, and diagnosis incidence, we generated a reflection of the country's population and health care system in spite of our approximation the national guidelines. Finally, we made assumptions about adherence to care prescribed by the health check that should reflect what could be achieved in a real-world program. Our assumptions about costs and QALYs were explored through a sensitivity analysis. In the present analysis we considered only direct medical costs. Future studies should examine other costs associated with launching such a program, including the costs for training health care providers and program management.

Despite these limitations, this study provides a realistic estimate of the likely benefits and costs of health checks in six European populations. We have modeled the settings unique to these six countries in detail, and employed the Archimedes Model to provide 30-year cost effectiveness estimates. Through trial validations, the Archimedes Model has been demonstrated to predict the effects of the screening, prevention, and management actions addressed by the health check.

Our study shows that a health check assessing diabetes, hypertension, lipids and smoking would likely be cost effective in all of the countries considered, Denmark, France, Italy, Germany, Poland, and the UK. Pre-screening strategies would likely improve the cost effectiveness and minimize the total budget impact of a health checks program, while still providing meaningful improvements in health.

## Supporting Information

File S1 **Description of the data sources used in the modeling.** **Figure S1,** Receiver operating characteristic (ROC) curves showing the discrimination of the generic risk test on each simulated population. **Table S1,** Characteristics for Denmark population (aged 20–85, unless specified otherwise). **Table S2,** Characteristics for Denmark subpopulations. **Table S3,** Characteristics for France population (aged 20–85, unless specified otherwise). **Table S4,** Characteristics for France subpopulations. **Table S5,** Characteristics for Germany population (aged 20–85, unless specified otherwise). **Table S6,** Characteristics for Germany subpopulations. **Table S7,** Characteristics for Italy population (aged 20–85, unless specified otherwise). **Table S8,** Characteristics for Italy subpopulations. **Table S9,** Characteristics for Poland population (aged 20–85, unless specified otherwise). **Table S10,** Characteristics for Poland subpopulations. **Table S11,** Characteristics for UK population (aged 20–85, unless specified otherwise). **Table S12,** Characteristics for UK subpopulations. **Table S13,** Cost assumptions used for Denmark. **Table S14,** Cost assumptions used for France. Table **S15,** Cost assumptions used for Germany. **Table S16,** Cost assumptions used for Italy. Table **S17,** Cost assumptions used for Poland. **Table S18,** Cost assumptions used for the United Kingdom.
(DOCX)

File S2 **Description of the model structure.**
(PDF)

File S3 **Description of the model calibration.**
(PDF)

File S4 **Description of the model validation.**
(PDF)
