# Supplementary material for: A Standardized Vascular Disease Health Check in Europe: A Cost-Effectiveness Analysis
Source: PLoS One. 2013 Jul 15;8(7):e66454. doi: 10.1371/journal.pone.0066454 (PMC3712021; doi:10.1371/journal.pone.0066454)
Supplement: File S1 — Description of the data sources used in the modeling. Figure S1, Receiver operating characteristic (ROC) curves showing the discrimination of the generic risk test on each simulated population. Table S1, Characteristics for Denmark population (aged 20–85, unless specified otherwise). Table S2, Characteristics for Denmark subpopulations. Table S3, Characteristics for France population (aged 20–85, unless specified otherwise). Table S4, Characteristics for France subpopulations. Table S5, Characteristics for Germany population (aged 20–85, unless specified otherwise). Table S6, Characteristics for Germany subpopulations. Table S7, Characteristics for Italy population (aged 20–85, unless specified otherwise). Table S8, Characteristics for Italy subpopulations. Table S9, Characteristics for Poland population (aged 20–85, unless specified otherwise). Table S10, Characteristics for Poland subpopulations. Table S11, Characteristics for UK population (aged 20–85, unless specified otherwise). Table S12, Characteristics for UK subpopulations. Table S13, Cost assumptions used for Denmark. Table S14, Cost assumptions used for France. Table S15, Cost assumptions used for Germany. Table S16, Cost assumptions used for Italy. Table S17, Cost assumptions used for Poland. Table S18, Cost assumptions used for the United Kingdom. (DOCX) [file pone.0066454.s001.docx]

**Web Appendix: Supplementary Material**

**A standardized vascular disease health check in Europe: a cost-effectiveness analysis**

# Section A: Baseline characteristics for simulated country-specific populations

Please note that the data sources used for the purpose of the present discussion have listed one of the following two data representations – population means, or, the proportion of the population with specified characteristics. Some data sources have not listed standard deviations, and consequently, we do not report standard deviations in the following tables.

| Outcome (subgroup) | Simulated Mean | Denmark Data | Denmark Data Reference |
| --- | --- | --- | --- |
| Baseline biomarkers |  |  |  |
| Smoking | 0•23 | 0•23 | (1) |
| Age (Among adults aged 30-60) | 44•5 | 44•9 | (2) |
| Total Cholesterol (Among adults aged 30-60) | 5•2 | 5•5 | (3) |
| Diastolic Blood Pressure (Among adults aged 30-60) | 75•1 | 81•0 | (3) |
| Systolic Blood Pressure (Among adults aged 30-60) | 121•8 | 129•0 | (3) |
| A1C (Among adults aged 30-60) | 5•41% | 5•80% | (3) |
| Prevalence of diagnosed condition |  |  |  |
| ESRD | 0•003 | 0•001 | (4) |
| TypeIIDM (Among males aged 30-60) | 0•049 | Range of values: ( 0, 0•058) | (5) |
| TypeIIDM (Among females aged 30-60) | 0•039 | Range of values: (0•005, 0•09) | (5) |
| HTN (Among males) | 0•238 | 0•250 | (6) |
| HTN (Among females) | 0•216 | 0•250 | (6) |
| Prevalence of treatments taken at baseline |  |  |  |
| ACEInhibitor | 0•078 | 0•078 | (7) |
| Antihypertensive | 0•171 | 0•151 | (7) |
| BetaBlocker | 0•045 | 0•044 | (7) |
| CCB | 0•045 | 0•049 | (7) |
| Diuretic | 0•072 | 0•074 | (7) |
| Insulin | 0•004 | 0•004 | (8) |
| OralDMAgent | 0•027 | 0•028 | (8) |
| Annual Incidence |  |  |  |
| ESRD | 0•0005 | 0•0001 | (9) |
| MI | 0•003 | 0•003 | (10) |
| Stroke | 0•003 | 0•002 | (10) |
| TypeIIDM | 0•003 | 0•005 | (11) |
| Death | 0•009 | 0•009 | (12) |

**Table S1. Characteristics for Denmark population (aged 20-85, unless specified otherwise)**

| Outcome | Simulated Mean | Denmark Data | Denmark Data Reference |
| --- | --- | --- | --- |
| Baseline Biomarkers | | | |
| Smoking | | | |
| Males aged 20-85 | 0•27 | 0•24 | (1) |
| Females aged 20-85 | 0•19 | 0•22 | (1) |
| Males aged 25-35 | 0•33 | 0•20 | (13) |
| Females aged 25-35 | 0•22 | 0•16 | (13) |
| Males aged 35-45 | 0•29 | 0•23 | (13) |
| Females aged 35-45 | 0•22 | 0•20 | (13) |
| Males aged 45-55 | 0•28 | 0•27 | (13) |
| Females aged 45-55 | 0•22 | 0•25 | (13) |
| Males aged 55-65 | 0•22 | 0•27 | (13) |
| Females aged 55-65 | 0•17 | 0•23 | (13) |
| Males aged 65-75 | 0•11 | 0•22 | (13) |
| Females aged 65-75 | 0•10 | 0•19 | (13) |
| Males aged 75-85 | 0•06 | 0•17 | (13) |
| Females aged 75-85 | 0•05 | 0•13 | (13) |
| Diabetics aged 20-85 | 0•11 | 0•28 | (14), (9), (2) |
| Age | | | |
| Male Diabetics aged 30-45 | 39•8 | 37•3 | (2) |
| Female Diabetics aged 30-45 | 40•1 | 37•3 | (2) |
| Male Diabetics aged 45-60 | 52•8 | 52•2 | (2) |
| Female Diabetics aged 45-60 | 53•4 | 52•2 | (2) |
| Male Diabetics aged 61-75 | 67•9 | 66•9 | (2) |
| Female Diabetics aged 61-75 | 68•4 | 67•1 | (2) |
| Diastolic Blood Pressure | | | |
| Diabetics aged 20-40 | 78 | 78 | (14) |
| Diabetics aged 40-60 | 80 | 81 | (14) |
| Diabetics aged 60-80 | 71 | 77 | (14) |
| Systolic Blood Pressure | | | |
| Diabetics aged 20-40 | 125 | 126 | (14) |
| Diabetics aged 40-60 | 135 | 135 | (14) |
| Diabetics aged 60-80 | 143 | 140 | (14) |
| Diabetics aged 20-85 | 139 | 138 | (15) |
| A1C | | | |
| Diabetics aged 20-40 | 8•4% | 8•2% | (14) |
| Diabetics aged 40-60 | 8•2% | 8•0% | (14) |
| Diabetics aged 60-80 | 8•1% | 7•7% | (14) |
| Diabetics aged 20-85 | 8•1% | 7•8% | (15) |
| LDL | | | |
| Diabetics aged 20-85 | 2•79 | 2•81 | (15) |
| Total Cholesterol | | | |
| Diabetics aged 20-85 | 5•07 | 4•32 | (15) |
| Triglycerides | | | |
| Diabetics aged 20-85 | 2•18 | 1•88 | (15) |
| BMI | | | |
| Diabetics aged 20-85 | 32•5 | 30•0 | (15) |
| Prevalence of diagnosed conditions | | | |
| HTN | | | |
| Males aged 20-85 | 0•238 | 0•250 | (6) |
| Females aged 20-85 | 0•216 | 0•250 | (6) |
| Diabetics aged 20-85 | 0•741 | 0•245 | (14), (9), (2) |
| Males aged 20-35 | 0•047 | 0•050 | (6) |
| Females aged 20-35 | 0•014 | 0•025 | (6) |
| Males aged 35-65 | 0•268 | 0•250 | (6) |
| Females aged 35-65 | 0•195 | 0•175 | (6) |
| Males aged 65-75 | 0•511 | 0•500 | (6) |
| Females aged 65-75 | 0•574 | 0•500 | (6) |
| Males aged 75-85 | 0•654 | 0•625 | (6) |
| Females aged 75-85 | 0•767 | 0•750 | (6) |
| ESRD | | | |
| Diabetics aged 20-85 | 0•0177 | 0•0040 | (14), (9), (2) |
| Foot Ulcer | | | |
| Diabetics aged 20-85 | 0•255 | 0•087 | (14), (9), (2) |
| MI | | | |
| Diabetics aged 20-85 | 0•120 | 0•060 | (14), (9), (2) |
| PDR | | | |
| Diabetics aged 20-85 | 0•060 | 0•059 | (14), (9), (2) |
| Stage3 CKD | | | |
| Diabetics aged 20-85 | 0•277 | 0•019 | (14), (9), (2) |
| Stroke | | | |
| Diabetics aged 20-85 | 0•077 | 0•071 | (14), (9), (2) |
| Type II DM | | | |
| Males aged 30-40 | 0•02 | 0•01 | (11), (2) |
| Females aged 30-40 | 0•01 | 0•02 | (11), (2) |
| Males aged 40-50 | 0•05 | 0•03 | (11), (2) |
| Females aged 40-50 | 0•04 | 0•03 | (11), (2) |
| Males aged 50-60 | 0•09 | 0•08 | (11), (2) |
| Females aged 50-60 | 0•07 | 0•06 | (11), (2) |
| Males aged 60-70 | 0•15 | 0•13 | (11), (2) |
| Females aged 60-70 | 0•12 | 0•10 | (11), (2) |
| Males aged 70-80 | 0•21 | 0•18 | (11), (2) |
| Females aged 70-80 | 0•17 | 0•15 | (11), (2) |
| Annual Incidence | | | |
| ESRD | | | |
| Diabetics aged 20-85 | 0•004 | 0•002 | (14), (9), (2) |
| Foot Ulcer | | | |
| Diabetics aged 20-85 | 0•022 | 0•012 | (14), (9), (2) |
| HTN | | | |
| Diabetics aged 20-85 | 0•016 | 0•035 | (14), (9), (2) |
| MI | | | |
| Diabetics aged 20-85 | 0•013 | 0•008 | (14), (9), (2) |
| PDR | | | |
| Diabetics aged 20-85 | 0•006 | 0•006 | (14), (9), (2) |
| Stage3 CKD | | | |
| Diabetics aged 20-85 | 0•014 | 0•003 | (14), (9), (2) |
| Stroke | | | |
| Diabetics aged 20-85 | 0•007 | 0•010 | (14), (9), (2) |
| CHD Death | | | |
| Males aged 25-45 | 0•0001 | 0•0001 | (12) |
| Females aged 25-45 | 0•0000 | 0•0001 | (12) |
| Males aged 45-65 | 0•001 | 0•001 | (12) |
| Females aged 45-65 | 0•0003 | 0•0003 | (12) |
| Males aged 65-85 | 0•002 | 0•004 | (12) |
| Females aged 65-85 | 0•001 | 0•002 | (12) |
| Stroke Death | | | |
| Males aged 25-45 | 0•00011 | 0•00004 | (12) |
| Females aged 25-45 | 0•0001 | 0•0001 | (12) |
| Males aged 45-65 | 0•0006 | 0•0004 | (12) |
| Females aged 45-65 | 0•0004 | 0•0003 | (12) |
| Males aged 65-85 | 0•0028 | 0•0018 | (12) |
| Females aged 65-85 | 0•0028 | 0•0012 | (12) |
| Death | | | |
| Males aged 25-45 | 0•003 | 0•005 | (12) |
| Females aged 25-45 | 0•001 | 0•003 | (12) |
| Males aged 45-65 | 0•010 | 0•027 | (12) |
| Females aged 45-65 | 0•006 | 0•017 | (12) |
| Males aged 65-85 | 0•038 | 0•070 | (12) |
| Females aged 65-85 | 0•029 | 0•061 | (12) |

**Table S2. Characteristics for Denmark subpopulations**

| Outcome (subgroup) | Simulated Mean | France Data | France Data Reference |
| --- | --- | --- | --- |
| Baseline biomarkers |  |  |  |
| Fraction with age 20-40 | 0•36 | 0•35 | (16) |
| Fraction with age 40-65 | 0•46 | 0•45 | (16) |
| Fraction with age 65-85 | 0•18 | 0•20 | (16) |
| Age (among men aged 20-74) | 44•97 | 44•20 | (17) |
| Age (among women aged 20-74) | 46•89 | 45•10 | (17) |
| Current smoker (among men aged 20-74) | 0•35 | 0•34 | (17) |
| Current smoker (among women aged 20-74) | 0•27 | 0•26 | (17) |
| BMI (among men aged 20-74) | 26•17 | 26•10 | (17) |
| BMI (among women aged 20-74) | 25•15 | 25•10 | (17) |
| FPG (among men aged 20-74) | 97•20 | 97•56 | (17) |
| FPG (among women aged 20-74) | 96•69 | 91•26 | (17) |
| Systolic blood pressure (among men aged 20-74) | 124•57 | 129•00 | (17) |
| Systolic blood pressure (among women aged 20-74) | 121•63 | 119•00 | (17) |
| Diastolic blood pressure (among men aged 20-74) | 78•94 | 79•00 | (17) |
| Diastolic blood pressure (among women aged 20-74) | 75•99 | 76•00 | (17) |
| Total Cholesterol (among men aged 20-74) | 5•32 | 5•31 | (17) |
| Total Cholesterol (among women aged 20-74) | 5•48 | 5•44 | (17) |
| LDL (among men aged 20-74) | 3•33 | 3•31 | (17) |
| LDL (among women aged 20-74) | 3•26 | 3•29 | (17) |
| Prevalence of diagnosed condition |  |  |  |
| DM (among men) | 0•06 | 0•06 | (18) |
| DM (among women) | 0•06 | 0•05 | (18) |
| Angina | 0•03 | 0•01 | (19-20) |
| MI | 0•02 | 0•01 | (19-20) |
| Stroke | 0•03 | 0•02 | (19-20) |
| CAD (among people with DM) | 0•13 | 0•17 | (21) |
| Stroke (among people with DM) | 0•06 | 0•07 | (21) |
| Macroalbuminuria (among people with DM) | 0•02 | 0•05 | (21) |
| CKD stage 3 or higher (among people with DM) | 0•20 | 0•18 | (21) |
| Diabetic foot ulcer (among people with DM) | 0•23 | 0•10 | (21) |
| Diabetic retinopathy (among people with DM) | 0•13 | 0•08 | (21) |
| Diabetes-related blindness (among people with DM) | 0•04 | 0•03 | (21) |
| Prevalence of treatments taken at baseline |  |  |  |
| ACEInhibitor | 0•14 | 0•14 | (1) |
| BetaBlocker | 0•05 | 0•06 | (1) |
| CCB | 0•05 | 0•05 | (1) |
| Dyslipidemia Medication | 0•08 | 0•09 | (1) |
| ACEInhibitor (among people with DM) | 0•40 | 0•60 | (21) |
| BetaBlocker (among people with DM) | 0•17 | 0•25 | (21) |
| CCB (among people with DM) | 0•15 | 0•26 | (21) |
| Diuretic (among people with DM) | 0•25 | 0•31 | (21) |
| Statins (among people with DM) | 0•49 | 0•47 | (21) |
| Insulin (among people with DM) | 0•17 | 0•16 | (21) |
| OralDMAgent (among people with DM) | 0•69 | 0•73 | (21) |
| Revascularization (among people with DM) | 0•07 | 0•11 | (21) |
| Diabetic foot amputation (among people with DM) | 0•04 | 0•02 | (21) |
| Annual Incidence |  |  |  |
| TypeIIDM (among men) | 0•003 | 0•002 | (18) |
| TypeIIDM (among women) | 0•003 | 0•002 | (18) |
| Hypertension | 0•010 | 0•022 | (22), (23) |
| MI | 0•002 | 0•002 | (19-20) |
| ESRD | 0•0004 | 0•0001 | (24), (25) |
| Stroke Death (men) | 0•0007 | 0•0003 | (26) |
| Stroke Death (women) | 0•0006 | 0•0001 | (26) |
| CHD Death (among men) | 0•0002 | 0•0006 | (26) |
| CHD Death (among women) | 0•0002 | 0•0001 | (26) |
| CV Death (among men) | 0•001 | 0•002 | (26) |
| CV Death (among women) | 0•001 | 0•001 | (26) |
| Death (among men) | 0•010 | 0•009 | (26) |
| Death (among women) | 0•008 | 0•004 | (26) |

**Table S3. Characteristics for France population (aged 20-85, unless specified otherwise)**

| Outcome | Simulated Mean | France Data | France Data Reference |
| --- | --- | --- | --- |
| Baseline Biomarkers |  |  |  |
| Smoking |  |  |  |
| Diabetic aged 20-85 | 0•108 | 0•103 | (21) |
| Age |  |  |  |
| Diabetic aged 20-85 | 62•533 | 64•900 | (21) |
| Prevalence of diagnosed conditions |  |  |  |
| DM |  |  |  |
| Aged 20-85 | 0•059 | 0•063 | (18) |
| Annual Incidence |  |  |  |
| DM |  |  |  |
| Aged 20-85 | 0•003 | 0•001 | (27) |
| Male aged 30-65 | 0•004 | 0•007 | (28), (29) |
| Female aged 30-65 | 0•003 | 0•004 | (28), (29) |
| MI |  |  |  |
| Male age 50-60 | 0•002 | 0•003 | (30) |

**Table S4. Characteristics for France subpopulations**

| Outcome (subgroup) | Simulated Mean | Germany Data | Germany Data Reference |
| --- | --- | --- | --- |
| Baseline biomarkers |  |  |  |
| Smoking | 0•25 | 0•25 | (31) |
| Age | 50•1 | 51•7 | (31) |
| Total Cholesterol | 5•46 | 5•36 | (31) |
| HDL | 1•56 | 1•58 | (31) |
| LDL | 3•38 | 3•30 | (31) |
| Diastolic Blood Pressure | 82•1 | 80•1 | (31) |
| Systolic Blood Pressure | 134•3 | 130•5 | (31) |
| Prevalence of diagnosed condition |  |  |  |
| DM | 0•080 | 0•089 | (32) |
| Dyslipidemia | 0•204 | 0•208 | (33) |
| HTN | 0•294 | 0•306 | (34) |
| Prevalence of treatments taken at baseline |  |  |  |
| ACEInhibitor | 0•082 | 0•083 | (35) |
| BetaBlocker | 0•026 | 0•027 | (35) |
| CCB | 0•022 | 0•023 | (35) |
| Diuretic | 0•024 | 0•024 | (35) |
| Insulin | 0•010 | 0•010 | (36) |
| OralDMAgent | 0•025 | 0•025 | (35) |
| Sulfonylurea | 0•006 | 0•006 | (36) |
| Annual Incidence |  |  |  |
| ESRD | 0•0008 | 0•0005 | (37) |
| Stroke | 0•004 | 0•003 | (38) |
| TypeIIDM (Among people aged 55-75) | 0•006 | 0•016 | (39) |
| Death (Male) | 0•012 | 0•008 | (40) |
| Death (Female) | 0•012 | 0•004 | (40) |

**Table S5. Characteristics for Germany population (aged 20-85, unless specified otherwise)**

| Outcome | Simulated Mean | Germany Data | Germany Data Reference |
| --- | --- | --- | --- |
| Baseline Biomarkers | | | |
| Smoking | | | |
| Males aged 20-85 | 0•29 | 0•28 | (31) |
| Females aged 20-85 | 0•21 | 0•24 | (31) |
| All aged 20-25 | 0•37 | 0•36 | (41) |
| All aged 25-30 | 0•37 | 0•38 | (41) |
| All aged 30-35 | 0•32 | 0•35 | (41) |
| All aged 35-40 | 0•32 | 0•34 | (41) |
| All aged 40-45 | 0•30 | 0•34 | (41) |
| All aged 45-50 | 0•30 | 0•35 | (41) |
| All aged 50-55 | 0•26 | 0•33 | (41) |
| All aged 55-60 | 0•20 | 0•26 | (41) |
| All aged 60-65 | 0•21 | 0•21 | (41) |
| All aged 65-70 | 0•12 | 0•14 | (41) |
| All aged 70-75 | 0•12 | 0•09 | (41) |
| All aged 75 and above | 0•07 | 0•05 | (41) |
| Age | | | |
| Males aged 20-85 | 47•6 | 53•0 | (31) |
| Females aged 20-85 | 52•5 | 50•9 | (31) |
| Diastolic Blood Pressure | | | |
| Males aged 20-85 | 83 | 81 | (31) |
| Females aged 20-85 | 81 | 79 | (31) |
| Males aged 20-30 | 78 | 78 | (42) |
| Females aged 20-30 | 74 | 75 | (42) |
| Males aged 30-40 | 84 | 84 | (42) |
| Females aged 30-40 | 81 | 78 | (42) |
| Males aged 40-50 | 87 | 88 | (42) |
| Females aged 40-50 | 84 | 82 | (42) |
| Males aged 50-60 | 87 | 89 | (42) |
| Females aged 50-60 | 85 | 86 | (42) |
| Males aged 60-70 | 84 | 88 | (42) |
| Females aged 60-70 | 83 | 86 | (42) |
| Males aged 70-80 | 79 | 83 | (42) |
| Females aged 70-80 | 78 | 83 | (42) |
| Systolic Blood Pressure | | | |
| Males aged 20-85 | 133•0 | 133•6 | (31) |
| Females aged 20-85 | 135•6 | 128•5 | (31) |
| Males aged 20-30 | 122•5 | 129•0 | (42) |
| Females aged 20-30 | 113•6 | 119•0 | (42) |
| Males aged 30-40 | 126•2 | 130•0 | (42) |
| Females aged 30-40 | 121•6 | 122•0 | (42) |
| Males aged 40-50 | 131•0 | 135•0 | (42) |
| Females aged 40-50 | 129•8 | 130•0 | (42) |
| Males aged 50-60 | 136•2 | 143•0 | (42) |
| Females aged 50-60 | 137•9 | 143•0 | (42) |
| Males aged 60-70 | 142•9 | 150•0 | (42) |
| Females aged 60-70 | 145•6 | 153•0 | (42) |
| Males aged 70-80 | 146•0 | 153•0 | (42) |
| Females aged 70-80 | 151•5 | 155•0 | (42) |
| LDL | | | |
| Males aged 20-85 | 3•43 | 3•30 | (31) |
| Females aged 20-85 | 3•33 | 3•30 | (31) |
| HDL | | | |
| Males aged 20-85 | 1•39 | 1•40 | (31) |
| Females aged 20-85 | 1•73 | 1•70 | (31) |
| Total Cholesterol | | | |
| Males aged 20-85 | 5•35 | 5•30 | (31) |
| Females aged 20-85 | 5•57 | 5•40 | (31) |
| Males aged 20-30 | 4•81 | 5•07 | (42) |
| Females aged 20-30 | 4•79 | 5•33 | (42) |
| Males aged 30-40 | 5•37 | 5•81 | (42) |
| Females aged 30-40 | 5•10 | 5•50 | (42) |
| Males aged 40-50 | 5•60 | 6•33 | (42) |
| Females aged 40-50 | 5•41 | 5•89 | (42) |
| Males aged 50-60 | 5•62 | 6•34 | (42) |
| Females aged 50-60 | 5•87 | 6•53 | (42) |
| Males aged 60-70 | 5•50 | 6•41 | (42) |
| Females aged 60-70 | 6•03 | 6•90 | (42) |
| Males aged 70-80 | 5•16 | 6•36 | (42) |
| Females aged 70-80 | 5•90 | 6•75 | (42) |
| BMI | | | |
| Males aged 20-25 | 25•8 | 23•4 | (42) |
| Females aged 20-25 | 26•1 | 22•0 | (42) |
| Males aged 25-30 | 26•5 | 24•9 | (42) |
| Females aged 25-30 | 26•3 | 23•7 | (42) |
| Males aged 30-40 | 28•0 | 26•3 | (42) |
| Females aged 30-40 | 27•9 | 24•7 | (42) |
| Males aged 40-50 | 27•9 | 27•2 | (42) |
| Females aged 40-50 | 27•7 | 26•1 | (42) |
| Males aged 50-60 | 27•3 | 27•9 | (42) |
| Females aged 50-60 | 27•6 | 27•8 | (42) |
| Males aged 60-70 | 27•3 | 27•9 | (42) |
| Females aged 60-70 | 27•5 | 28•4 | (42) |
| Males aged 70-75 | 26•9 | 27•0 | (42) |
| Females aged 70-75 | 27•0 | 26•5 | (42) |
| Males aged 25-70 | 27•5 | 27•7 | (42) |
| Females aged 25-70 | 27•6 | 26•8 | (42) |
| Prevalence of diagnosed conditions | | | |
| Foot Ulcer | | | |
| Diabetics aged 25-75 | 0•246 | 0•050 | (43) |
| Diabetics aged 20-85 | 0•264 | 0•040 | (44) |
| Microalbuminuria | | | |
| Diabetics aged 25-75 | 0•166 | 0•190 | (43) |
| Diabetics aged 20-85 | 0•197 | 0•150 | (43) |
| Retinopathy | | | |
| Diabetics aged 25-75 | 0•141 | 0•130 | (43) |
| Diabetics aged 20-85 | 0•143 | 0•160 | (43) |
| MI | | | |
| Diabetics aged 20-85 | 0•103 | 0•106 | (45) |
| Stage3 CKD | | | |
| Diabetics aged 20-85 | 0•252 | 0•080 | (43) |
| Stroke | | | |
| Diabetics aged 20-85 | 0•092 | 0•067 | (45) |
| Annual Incidence | | | |
| Blindness | | | |
| Diabetics aged 20-85 | 0•004 | 0•0006 | (46) |
| MI | | | |
| All aged 15-45 | 0•0004 | 0•0003 | (38) |
| All aged 45-65 | 0•003 | 0•003 | (38) |
| All aged 65 and above | 0•011 | 0•008 | (38) |
| Stroke | | | |
| Non-Diabetic Males aged 20-85 | 0•004 | 0•003 | (47) |
| Diabetic Males aged 20-85 | 0•009 | 0•005 | (47) |
| Non-Diabetic Females aged 20-85 | 0•004 | 0•002 | (47) |
| Diabetic Females aged 20-85 | 0•008 | 0•003 | (47) |

**Table S6. Characteristics for Germany subpopulations**

| Outcome (subgroup) | Simulated Mean | Italy Data | Italy Data Reference |
| --- | --- | --- | --- |
| Baseline biomarkers |  |  |  |
| Fraction aged 20-30 | 0•14 | 0•14 | (48) |
| Fraction aged 30-40 | 0•19 | 0•19 | (48) |
| Fraction aged 40-50 | 0•21 | 0•20 | (48) |
| Fraction aged 50-60 | 0•17 | 0•16 | (48) |
| Fraction aged 60-70 | 0•15 | 0•15 | (48) |
| Fraction aged 70-80 | 0•12 | 0•12 | (48) |
| Fraction aged 80-85 | 0•03 | 0•04 | (48) |
| Fraction with BMI between 25-30 (ages 35-75) | 0•41 | 0•42 | (49) |
| Fraction with BMI >= 30 (ages 35-75) | 0•21 | 0•20 | (49) |
| BMI (age 35-75) | 27•1 | 26•5 | (49) |
| Total Cholesterol (age 35-75) | 5•46 | 5•33 | (49) |
| LDL (age 35-75) | 3•35 | 3•28 | (49) |
| HDL (age 35-75) | 1•40 | 1•40 | (49) |
| Triglycerides (age 35-75) | 1•56 | 1•43 | (49) |
| FPG (age 35-75) | 99•9 | 90•0 | (49) |
| Systolic Blood Pressure (age 35-75) | 135•5 | 133•0 | (49) |
| Diastolic Blood Pressure (age 35-75) | 82•5 | 84•0 | (49) |
| Smoker (age 35-75) | 0•24 | 0•25 | (49) |
| FPG | 98•8 | 98•9 | (42) |
| Fraction with Total Cholesterol >= 193•4 | 0•63 | 0•65 | (42) |
| Fraction with Total Cholesterol >= 239•8 | 0•21 | 0•22 | (42) |
| Prevalence of diagnosed condition |  |  |  |
| DM (age 18-68) | 0•04 | 0•05 | (50) |
| HTN | 0•22 | 0•22 | (50) |
| Angina | 0•03 | 0•04 | (51) |
| MI | 0•02 | 0•01 | (51) |
| Stroke | 0•04 | 0•01 | (51) |
| ESRD | 0•003 | 0•001 | (52) |
| Prevalence of treatments taken at baseline |  |  |  |
| Antihypertensive | 0•18 | 0•23 | (53) |
| ACE Inhibitor + ARB | 0•12 | 0•12 | (53) |
| BetaBlocker | 0•04 | 0•04 | (53) |
| CCB | 0•04 | 0•04 | (53) |
| Diuretic | 0•06 | 0•06 | (53) |
| Statin | 0•10 | 0•10 | (53) |
| Aspirin | 0•08 | 0•08 | (53) |
| Metformin (diagnosed DM) | 0•36 | 0•37 | (53) |
| Sulfonylurea (diagnosed DM) | 0•18 | 0•17 | (53) |
| TZD (diagnosed DM) | 0•05 | 0•05 | (53) |
| Insulin (diagnosed DM) | 0•12 | 0•12 | (53) |
| Annual Incidence |  |  |  |
| DM | 0•004 | 0•004 | (54) |
| ESRD | 0•0006 | 0•0002 | (52) |
| MI (men with DM age 40-85) | 0•01 | 0•01 | (55) |
| MI (women with DM age 40-85) | 0•008 | 0•005 | (55) |
| Stroke (men with DM age 40-85 no CVD) | 0•004 | 0•006 | (56) |
| Stroke (women with DM age 40-85 no CVD) | 0•003 | 0•006 | (56) |
| Stroke (men with DM age 40-85 with CVD) | 0•02 | 0•01 | (56) |
| Stroke (women with DM age 40-85 with CVD) | 0•03 | 0•01 | (56) |

**Table S7. Characteristics for Italy population (aged 20-85, unless specified otherwise)**

| Outcome | Simulated Mean | Italy Data | Italy Data Reference |
| --- | --- | --- | --- |
| Baseline Biomarkers |  |  |  |
| BMI |  |  |  |
| Ages 20-85 | 26•6 | 26•1 | (42) |
| Men | 26•8 | 26•8 | (42) |
| Women | 26•4 | 25•4 | (42) |
| Men aged 35-75 | 27•2 | 27•0 | (49) |
| Women aged 35-75 | 27•0 | 26•0 | (49) |
| Fraction with BMI between 25-30 (men age 35-75) | 0•49 | 0•50 | (49) |
| Fraction with BMI between 25-30 (women age 35-75) | 0•34 | 0•34 | (49) |
| Fraction with BMI >= 30 (men age 35-75) | 0•18 | 0•18 | (49) |
| Fraction with BMI >= 30 (women age 35-75) | 0•23 | 0•22 | (49) |
| Diagnosed DM | 30•96 | 29•40 | (57) |
| Total Cholesterol |  |  |  |
| Ages 20-85 | 5•32 | 5•45 | (42) |
| Men | 5•23 | 5•40 | (42) |
| Women | 5•41 | 5•50 | (42) |
| Fraction with Total Cholesterol >= 193•4 (men) | 0•60 | 0•64 | (42) |
| Fraction with Total Cholesterol >= 239•8 (men) | 0•19 | 0•20 | (42) |
| Fraction with Total Cholesterol >= 193•4 (women) | 0•65 | 0•67 | (42) |
| Fraction with Total Cholesterol >= 239•8 (women) | 0•24 | 0•25 | (42) |
| Men aged 35-75 | 5•37 | 5•30 | (49) |
| Women aged 35-75 | 5•55 | 5•35 | (49) |
| Diagnosed DM | 5•13 | 4•85 | (57) |
| LDL |  |  |  |
| Men aged 35-75 | 3•35 | 3•31 | (49) |
| Women aged 35-75 | 3•34 | 3•26 | (49) |
| Diagnosed DM | 2•92 | 3•00 | (57) |
| HDL |  |  |  |
| Men aged 35-75 | 1•27 | 1•27 | (49) |
| Women aged 35-75 | 1•53 | 1•50 | (49) |
| Diagnosed DM | 1•29 | 1•29 | (57) |
| Triglycerides |  |  |  |
| Men aged 35-75 | 1•63 | 1•60 | (49) |
| Women aged 35-75 | 1•49 | 1•26 | (49) |
| Diagnosed DM | 2•01 | 1•63 | (57) |
| Systolic Blood Pressure |  |  |  |
| Age 20-85 | 132•9 | 131•0 | (42) |
| Men | 133•0 | 133•9 | (42) |
| Women | 132•7 | 128•2 | (42) |
| Men aged 35-75 | 134•9 | 135•0 | (49) |
| Women aged 35-75 | 136•0 | 132•0 | (49) |
| Diagnosed DM | 137•5 | 139•6 | (57) |
| Diastolic Blood Pressure |  |  |  |
| Men age 35-75 | 83•4 | 86•0 | (49) |
| Women age 35-75 | 81•6 | 82•0 | (49) |
| Diagnosed DM | 79•8 | 79•8 | (57) |
| Fasting Plasma Glucose |  |  |  |
| Men | 99•9 | 102•6 | (42) |
| Women | 97•7 | 95•4 | (42) |
| Men aged 35-75 | 101•3 | 93•0 | (49) |
| Women aged 35-75 | 98•5 | 87•0 | (49) |
| Smoking |  |  |  |
| Age 20-85 | 0•25 | 0•22 | (42) |
| Men | 0•30 | 0•29 | (42) |
| Women | 0•21 | 0•16 | (42) |
| Men aged 35-75 | 0•28 | 0•30 | (49) |
| Women age 35-75 | 0•21 | 0•21 | (49) |
| Diagnosed DM | 0•16 | 0•18 | (57) |
| Age |  |  |  |
| Fraction with Age 20-30 (women) | 0•13 | 0•13 | (58) |
| Fraction with Age 30-40 (women) | 0•18 | 0•18 | (58) |
| Fraction with Age 40-50 (women) | 0•20 | 0•20 | (58) |
| Fraction with Age 50-60 (women) | 0•17 | 0•16 | (58) |
| Fraction with Age 60-70 (women) | 0•15 | 0•15 | (58) |
| Fraction with Age 70-80 (women) | 0•13 | 0•13 | (58) |
| Fraction with Age 80-85 (women) | 0•03 | 0•05 | (58) |
| Fraction with Age 20-30 (men) | 0•14 | 0•14 | (58) |
| Fraction with Age 30-40 (men) | 0•20 | 0•20 | (58) |
| Fraction with Age 40-50 (men) | 0•21 | 0•21 | (58) |
| Fraction with Age 50-60 (men) | 0•17 | 0•16 | (58) |
| Fraction with Age 60-70 (men) | 0•14 | 0•14 | (58) |
| Fraction with Age 70-80 (men) | 0•11 | 0•11 | (58) |
| Fraction with Age 80-85 (men) | 0•02 | 0•03 | (58) |
| Prevalence of diagnosed conditions |  |  |  |
| DM |  |  |  |
| Ages 20-80 | 0•06 | 0•05 | (59) |
| Men aged 18-68 | 0•05 | 0•05 | (50) |
| Women aged 18-68 | 0•03 | 0•05 | (50) |
| Hypertension |  |  |  |
| Men | 0•24 | 0•22 | (50) |
| Women | 0•21 | 0•21 | (50) |
| Angina |  |  |  |
| Men | 0•03 | 0•03 | (51) |
| Women | 0•03 | 0•04 | (51) |
| Diagnosed DM | 0•09 | 0•13 | (60) |
| Foot Ulcer |  |  |  |
| Diagnosed DM | 0•21 | 0•07 | (61) |
| Diabetic Retinopathy |  |  |  |
| NPDR (diagnosed DM) | 0•39 | 0•16 | (60) |
| PDR (diagnosed DM) | 0•10 | 0•03 | (60) |
| ESRD |  |  |  |
| Diagnosed DM | 0•01 | 0•01 | (60) |
| MI |  |  |  |
| Men | 0•03 | 0•02 | (51) |
| Women | 0•015 | 0•004 | (51) |
| Diagnosed DM | 0•09 | 0•08 | (60) |
| Stroke |  |  |  |
| Men | 0•05 | 0•01 | (51) |
| Women | 0•03 | 0•01 | (51) |
| Diagnosed DM | 0•08 | 0•03 | (60) |
| Prevalence of treatments |  |  |  |
| Antihypertensive |  |  |  |
| Anthihypertensive (men) | 0•20 | 0•22 | (53) |
| Anthihypertensive (women) | 0•17 | 0•24 | (53) |
| Antihypertensive (age 20-85) | 0•18 | 0•36 | (62) |
| ACE Inhibitor (age 20-85) | 0•12 | 0•14 | (62) |
| Beta Blocker (age 20-85) | 0•04 | 0•04 | (62) |
| CCB (age 20-85) | 0•04 | 0•06 | (62) |
| Diuretic (age 20-85) | 0•06 | 0•02 | (62) |
| ACE Inhibitor (diagnosed DM) | 0•51 | 0•51 | (57) |
| Beta Blocker (diagnosed DM) | 0•17 | 0•17 | (57) |
| Antihyperglycemic |  |  |  |
| DM Medication (age 20-85) | 0•04 | 0•07 | (53) |
| DM Medication (men) | 0•04 | 0•07 | (53) |
| DM Medication (women) | 0•03 | 0•06 | (53) |
| DM Medication (diagnosed DM) | 0•58 | 0•90 | (57) |
| Oral DM Medication (diagnosed DM) | 0•50 | 0•74 | (57) |
| Insulin (diagnosed DM) | 0•12 | 0•29 | (57) |
| Metformin (diagnosed DM) | 0•36 | 0•52 | (57) |
| Sulfonylurea (diagnosed DM) | 0•18 | 0•35 | (57) |
| Insulin (diagnosed DM) | 0•12 | 0•27 | (63) |
| Metformin (diagnosed DM) | 0•36 | 0•55 | (63) |
| Sulfonylurea (diagnosed DM) | 0•18 | 0•35 | (63) |
| TZD (diagnosed DM) | 0•05 | 0•03 | (63) |
| Antidyslipidemic |  |  |  |
| Dyslipidemia Medication (age 20-85) | 0•10 | 0•06 | (62) |
| Statin (age 20-85) | 0•10 | 0•05 | (62) |
| Statin (diagnosed DM) | 0•49 | 0•34 | (57) |
| Annual Incidence |  |  |  |
| DM |  |  |  |
| Ages 20-85 | 0•004 | 0•008 | (64) |

**Table S8. Characteristics for Italy subpopulations**

| Outcome (subgroup) | Simulated Mean | Poland Data | Poland Data Reference |
| --- | --- | --- | --- |
| Baseline biomarkers |  |  |  |
| Fraction with age 20-30 | 0•20 | 0•20 | (65) |
| Fraction with age 30-40 | 0•20 | 0•20 | (65) |
| Fraction with age 40-50 | 0•16 | 0•16 | (65) |
| Fraction with age 50-60 | 0•20 | 0•20 | (65) |
| Fraction with age 60-70 | 0•13 | 0•13 | (65) |
| Fraction with age 70-80 | 0•08 | 0•08 | (65) |
| Fraction with age 80-85 | 0•03 | 0•03 | (65) |
| Fraction with BMI >- 25 | 0•58 | 0•59 | (42) |
| Fraction with BMI >= 30 | 0•25 | 0•25 | (42) |
| BMI | 26•6 | 26•6 | (42) |
| Total Cholesterol | 5•24 | 5•25 | (42) |
| FPG | 93•4 | 93•5 | (42) |
| Systolic Blood Pressure | 133•4 | 134•0 | (42) |
| Smoker | 0•27 | 0•27 | (42) |
| Fraction with Total Cholesterol >= 193•4 | 0•58 | 0•59 | (42) |
| Fraction with Total Cholesterol >= 239•8 | 0•18 | 0•19 | (42) |
| Prevalence of diagnosed condition |  |  |  |
| DM | 0•05 | 0•06 | (66) |

**Table S9. Characteristics for Poland population (aged 20-85, unless specified otherwise)**

| Outcome | Simulated Mean | Poland Data | Poland Data Reference |
| --- | --- | --- | --- |
| Baseline Biomarkers |  |  |  |
| BMI |  |  |  |
| Men | 26•8 | 26•8 | (42) |
| Women | 26•5 | 26•4 | (42) |
| Fraction with BMI >= 25 (men) | 0•63 | 0•63 | (42) |
| Fraction with BMI >= 25 (women) | 0•55 | 0•55 | (42) |
| Fraction with BMI >= 30 (men) | 0•24 | 0•24 | (42) |
| Fraction with BMI >= 30 (women) | 0•27 | 0•27 | (42) |
| Diagnosed DM | 32•3 | 30•4 | (67) |
| Diagnosed DM | 32•3 | 31•0 | (67) |
| Total Cholesterol |  |  |  |
| Men | 5•24 | 5•30 | (42) |
| Women | 5•24 | 5•20 | (42) |
| Fraction with Total Cholesterol >= 193•4 (men) | 0•60 | 0•60 | (42) |
| Fraction with Total Cholesterol >= 239•8 (men) | 0•19 | 0•19 | (42) |
| Fraction with Total Cholesterol >= 193•4 (women) | 0•57 | 0•57 | (42) |
| Fraction with Total Cholesterol >= 239•8 (women) | 0•18 | 0•18 | (42) |
| Fraction with Total Cholesterol (diagnosed DM) | 200•5 | 212•1 | (67) |
| Diagnosed DM | 5•19 | 5•48 | (67) |
| Diagnosed DM | 5•19 | 5•11 | (67) |
| LDL |  |  |  |
| Diagnosed DM | 2•84 | 3•40 | (67) |
| Diagnosed DM | 2•84 | 3•13 | (67) |
| Diagnosed DM | 2•84 | 3•89 | (67) |
| HDL |  |  |  |
| Diagnosed DM | 1•35 | 1•26 | (67) |
| Diagnosed DM | 1•35 | 1•16 | (67) |
| Diagnosed DM | 1•35 | 1•44 | (67) |
| Triglycerides |  |  |  |
| Diagnosed DM | 2•20 | 1•80 | (67) |
| Diagnosed DM | 2•20 | 2•31 | (67) |
| Systolic Blood Pressure |  |  |  |
| Men | 133•6 | 136•0 | (42) |
| Women | 133•3 | 132•1 | (42) |
| Diagnosed DM | 146•4 | 137•0 | (67) |
| Diagnosed DM | 146•4 | 148•0 | (67) |
| Diagnosed DM | 146•4 | 138•0 | (67) |
| Diastolic Blood Pressure |  |  |  |
| Diagnosed DM | 74•8 | 83•0 | (67) |
| Diagnosed DM | 74•8 | 87•0 | (67) |
| Fasting Plasma Glucose |  |  |  |
| Men | 93•0 | 95•4 | (42) |
| Women | 93•8 | 91•8 | (42) |
| Smoking |  |  |  |
| Men | 0•34 | 0•34 | (67) |
| Women | 0•21 | 0•21 | (67) |
| Age |  |  |  |
| Fraction with age 20-30 (women) | 0•19 | 0•19 | (65) |
| Fraction with age 30-40 (women) | 0•19 | 0•19 | (65) |
| Fraction with age 40-50 (women) | 0•16 | 0•16 | (65) |
| Fraction with age 50-60 (women) | 0•19 | 0•20 | (65) |
| Fraction with age 60-70 (women) | 0•14 | 0•14 | (65) |
| Fraction with age 70-80 (women) | 0•10 | 0•10 | (65) |
| Fraction with age 80-85 (women) | 0•04 | 0•04 | (65) |
| Fraction with age 20-30 (men) | 0•21 | 0•21 | (65) |
| Fraction with age 30-40 (men) | 0•21 | 0•21 | (65) |
| Fraction with age 40-50 (men) | 0•17 | 0•17 | (65) |
| Fraction with age 50-60 (men) | 0•20 | 0•20 | (65) |
| Fraction with age 60-70 (men) | 0•12 | 0•12 | (65) |
| Fraction with age 70-80 (men) | 0•07 | 0•07 | (65) |
| Fraction with age 80-85 (men) | 0•02 | 0•02 | (65) |
| Fraction with age (diagnosed DM) | 62•6 | 60•0 | (67) |
| Fraction with age (diagnosed DM) | 62•6 | 59•0 | (67) |
| Prevalence of diagnosed conditions |  |  |  |
| DM |  |  |  |
| Men | 0•04 | 0•06 | (66) |
| Women | 0•06 | 0•06 | (66) |
| Hypertension |  |  |  |
| Diagnosed DM | 0•75 | 0•79 | (67) |
| Diagnosed DM | 0•75 | 0•76 | (67) |
| Diabetic Retinopathy |  |  |  |
| Diagnosed DM | 0•29 | 0•22 | (67) |
| Diagnosed DM | 0•29 | 0•09 | (67) |
| Stroke |  |  |  |
| Diagnosed DM | 0•09 | 0•04 | (67) |
| Diagnosed DM | 0•09 | 0•05 | (67) |

**Table S10. Characteristics for Poland subpopulations**

| Outcome (subgroup) | Simulated Mean | UK Data | UK Data Reference |
| --- | --- | --- | --- |
| Baseline biomarkers |  |  |  |
| Smoking | 0•22 | 0•22 | (68) |
| HDL | 1•45 | 1•46 | (69) |
| HbA1c | 5•4% | 5•6% | (69) |
| Diastolic Blood Pressure | 73•9 | 72•8 | (68) |
| Systolic Blood Pressure | 128•4 | 126•5 | (68) |
| Prevalence of diagnosed condition |  |  |  |
| DM | 0•063 | 0•055 | (68) |
| Stage3CKD | 0•055 | 0•053 | (68) |
| MI (Among Males) | 0•030 | 0•041 | (70) |
| Stroke (Among Males) | 0•037 | 0•024 | (70) |
| MI (Among Females) | 0•017 | 0•017 | (70) |
| Stroke (Among Females) | 0•028 | 0•022 | (70) |
| Prevalence of treatments taken at baseline |  |  |  |
| Antihypertensive | 0•151 | 0•140 | (71) |
| Insulin (Among Diabetics) | 0•246 | 0•255 | (72) |
| OralDMAgent (Among Diabetics) | 0•643 | 0•651 | (72) |

**Table S11. Characteristics for UK population (aged 20-85, unless specified otherwise)**

| Outcome | Simulated Mean | UK Data | UK Data Reference |
| --- | --- | --- | --- |
| Prevalence of diagnosed conditions | | | |
| DM | | | |
| All aged 16-24 | 0•003 | 0•004 | (71) |
| All aged 25-34 | 0•008 | 0•009 | (71) |
| All aged 35-44 | 0•025 | 0•025 | (71) |
| All aged 45-54 | 0•057 | 0•058 | (71) |
| All aged 55-64 | 0•103 | 0•084 | (71) |
| All aged 65-74 | 0•139 | 0•123 | (71) |
| All aged 75 and above | 0•176 | 0•155 | (71) |
| MI | | | |
| All Males aged 35-44 | 0•003 | 0•006 | (70) |
| All Females aged 35-44 | 0•001 | 0•001 | (70) |
| All Males aged 45-54 | 0•014 | 0•021 | (70) |
| All Females aged 45-54 | 0•005 | 0•007 | (70) |
| All Males aged 55-64 | 0•044 | 0•063 | (70) |
| All Females aged 55-64 | 0•016 | 0•016 | (70) |
| All Males aged 65-74 | 0•103 | 0•144 | (70) |
| All Females aged 65-74 | 0•044 | 0•033 | (70) |
| All Males aged 75 and above | 0•177 | 0•166 | (70) |
| All Females aged 75 and above | 0•081 | 0•107 | (70) |
| Stroke | | | |
| All Males aged 35-44 | 0•013 | 0•005 | (70) |
| All Females aged 35-44 | 0•005 | 0•004 | (70) |
| All Males aged 45-54 | 0•026 | 0•012 | (70) |
| All Females aged 45-54 | 0•013 | 0•009 | (70) |
| All Males aged 55-64 | 0•053 | 0•030 | (70) |
| All Females aged 55-64 | 0•027 | 0•023 | (70) |
| All Males aged 65-74 | 0•107 | 0•071 | (70) |
| All Females aged 65-74 | 0•061 | 0•042 | (70) |
| All Males aged 75 and above | 0•186 | 0•121 | (70) |
| All Females aged 75 and above | 0•130 | 0•107 | (70) |

**Table S12. Characteristics for UK subpopulations**

**Cost data**

We used country specific cost data when available and rescaled the US costs (Medicare Current Beneficiary Survey (MCBS)(73), Medicare Limited Data Set (LDS)(74) and Medicare Part D Drug data(75)) by constant factors corresponding to the country and type of cost: health care, or treatment, for cost items when specific data was not available. The cost scaling factors for each country were based on the mean ratio of target country costs to US costs, among items for which costs were available. All references for available data cost are listed below; if a reference is not provided for a cost item, it was scaled from US Medicare costs.

All costs are reported in euros, converted using the rate from http://www.xe.com/ucc/ on 2011.03.04.

All health care cost values are unit costs, unless noted otherwise. All treatment costs are per-day, unless noted otherwise.

| **Cost Event Name** | **Cost used for Denmark (€)** | **Reference** |
| --- | --- | --- |
| Cardiac Catheterization (Ambulatory) | 537•85 | Cost data provided by Novo Nordisk |
| Ambulatory visit cost | 300•96 | Cost data provided by Novo Nordisk |
| Laser Photocoagulation (Outpatient) | 278•63 | (59) |
| FPG Test (Outpatient) | 9•57 |  |
| Full Lipid Panel (Outpatient) | 15•74 |  |
| HbA1c Test (Outpatient) | 14•52 | Cost data provided by Novo Nordisk |
| Ketones Test (Outpatient) | 6•18 |  |
| Serum Creatinine Test (Outpatient) | 13•98 | Cost data provided by Novo Nordisk |
| Urinary Albumin Test (Outpatient) | 9•84 |  |
| Outpatient visit cost | 99•71 | Cost data provided by Novo Nordisk |
| CABG (Inpatient) | 21851•00 | Cost data provided by Novo Nordisk |
| Cardiac catheterization (Inpatient) | 537•85 | Cost data provided by Novo Nordisk |
| Diabetes amputation (Inpatient) | 11558•86 | (59) |
| Diabetes DKA (Inpatient) | 3156•02 | (59) |
| Kidney transplant (Inpatient) | 30053•95 | (59, 76-77) |
| MI (Inpatient) | 7762•57 | (59, 76-77) |
| PCI (Inpatient) | 537•85 | Cost data provided by Novo Nordisk |
| Renal failure (Inpatient) | 2852•34 | Cost data provided by Novo Nordisk |
| Stable angina (Inpatient) | 1727•83 | (59, 76-77) |
| Stroke (Inpatient) | 11053•46 | (59, 76-77) |
| Unstable angina (Inpatient) | 1727•83 | (59, 76-77) |
| Chest pain (Inpatient) | 1727•83 | (59, 76-77) |
| Atrial Fibrillation (annual cost) | 6052•99 | (59, 76-77) |
| CHF (annual cost) | 8304•75 | (59, 76-77) |
| Foot Ulcer (one-time cost) | 158•86 | (59, 76-77) |
| Stroke (annual cost) | 3558•34 | (59, 76-77) |
| ACE-Inhibitor (Treatment) | 0•03 | (76) |
| Aspirin (Treatment) | 0•06 | (76) |
| Statin (Treatment) | 0•19 | (76) |
| Beta Blocker (Treatment) | 0•13 | (76) |
| Calcium Channel Blocker (Treatment) | 0•08 | (76) |
| Clopidogrel (Treatment) | 0•14 | (76) |
| Standard lifestyle change (Treatment) | 0•42 |  |
| Thiazide Diuretic (Treatment) | 0•06 | (76) |
| Glitazone (Treatment) | 1•91 | (76) |
| Hemodialysis (Treatment) | 141•32 | (78) |
| Insulin (Treatment) | 0•56 | (76) |
| Lasix (Treatment) | 0•15 | (76) |
| LMW Heparin (Treatment) | 0•94 |  |
| Metformin (Treatment) | 0•16 | (76) |
| Smoking Cessation (cost per quitter, Treatment) | 344•04 | (79-80) |
| Sulfonylurea (Treatment) | 0•24 | (76) |
| Warfarin (Treatment) | 0•14 | (76) |
| Intensive lifestyle advice (Treatment) | 0•42 | (81) |

Table S13. Cost assumptions used for Denmark.

| **Cost Event Name** | **Cost used for France (€)** | **Reference** |
| --- | --- | --- |
| Cardiac Catheterization (Ambulatory) | 1205•64 |  |
| Ambulatory visit cost | 104•64 |  |
| Laser Photocoagulation (Outpatient) | 448•22 | (82) |
| FPG Test (Outpatient) | 8•88 |  |
| Full Lipid Panel (Outpatient) | 14•60 |  |
| HbA1c Test (Outpatient) | 14•40 |  |
| Ketones Test (Outpatient) | 5•73 |  |
| Serum Creatinine Test (Outpatient) | 9•30 |  |
| Urinary Albumin Test (Outpatient) | 9•13 |  |
| Outpatient visit cost | 23•00 | (83) |
| CABG (Inpatient) | 15214•13 |  |
| Cardiac catheterization (Inpatient) | 5988•52 |  |
| Diabetes amputation (Inpatient) | 9178•04 | (84-85) |
| Diabetes DKA (Inpatient) | 3487•02 | (84-85) |
| Kidney transplant (Inpatient) | 19812•32 | (84-85) |
| MI (Inpatient) | 3930•57 | (84-86) |
| PCI (Inpatient) | 6208•98 |  |
| Renal failure (Inpatient) | 3762•29 |  |
| Stable angina (Inpatient) | 3190•58 | (84-86) |
| Stroke (Inpatient) | 5030•94 | (84-86) |
| Unstable angina (Inpatient) | 3190•58 | (84-86) |
| Chest pain (Inpatient) | 3190•58 | (84-86) |
| Atrial Fibrillation (annual cost) | 7210•97 |  |
| CHF (annual cost) | 3844•78 | Cost data provided by Novo Nordisk |
| Foot Ulcer (one-time cost) | 371•27 | (87) |
| Stroke (annual cost) | 1359•48 | (84-85) |
| ACE-Inhibitor (Treatment) | 0•42 | (84-85) |
| Aspirin (Treatment) | 0•09 | (84-85) |
| Statin (Treatment) | 1•08 | (84-85) |
| Beta Blocker (Treatment) | 0•53 |  |
| Calcium Channel Blocker (Treatment) | 0•96 |  |
| Clopidogrel (Treatment) | 2•30 |  |
| Standard lifestyle change (Treatment) | 1•09 |  |
| Thiazide Diuretic (Treatment) | 0•10 |  |
| Glitazone (Treatment) | 2•63 |  |
| Hemodialysis (Treatment) | 260•69 | (84-85) |
| Insulin (Treatment) | 2•42 |  |
| Lasix (Treatment) | 0•31 |  |
| LMW Heparin (Treatment) | 2•43 |  |
| Metformin (Treatment) | 0•36 |  |
| Smoking Cessation (cost per quitter, Treatment) | 319•07 | (79-80) |
| Sulfonylurea (Treatment) | 0•28 |  |
| Warfarin (Treatment) | 0•38 |  |
| Intensive lifestyle advice (Treatment) | 1•09 | (81) |

Table S14. Cost assumptions used for France.

| **Cost Event Name** | **Cost used for Germany (€)** | **Reference** |
| --- | --- | --- |
| Cardiac Catheterization (Ambulatory) | 1783•50 |  |
| Ambulatory visit cost | 325•26 | (88) |
| Laser Photocoagulation (Outpatient) | 296•37 | (89) |
| FPG Test (Outpatient) | 13•13 |  |
| Full Lipid Panel (Outpatient) | 21•60 |  |
| HbA1c Test (Outpatient) | 21•30 |  |
| Ketones Test (Outpatient) | 8•48 |  |
| Serum Creatinine Test (Outpatient) | 13•76 |  |
| Urinary Albumin Test (Outpatient) | 2•11 | (89-90) |
| Outpatient visit cost | 34•77 | (89, 91) |
| CABG (Inpatient) | 22506•32 |  |
| Cardiac catheterization (Inpatient) | 8858•84 |  |
| Diabetes amputation (Inpatient) | 15405•10 | (92) |
| Diabetes DKA (Inpatient) | 2919•30 | (93-94) |
| Kidney transplant (Inpatient) | 55293•45 | (95-96) |
| MI (Inpatient) | 6938•23 | (96-97) |
| PCI (Inpatient) | 9184•97 |  |
| Renal failure (Inpatient) | 5565•57 |  |
| Stable angina (Inpatient) | 3842•00 | (93, 96, 98) |
| Stroke (Inpatient) | 14295•81 | (96, 99) |
| Unstable angina (Inpatient) | 3842•00 | (93, 96, 98) |
| Chest pain (Inpatient) | 2156•14 |  |
| Atrial Fibrillation (annual cost) | 10667•22 |  |
| CHF (annual cost) | 6700•24 | (99) |
| Foot Ulcer (one-time cost) | 462•18 | (87) |
| Stroke (annual cost) | 7472•97 | (96) |
| ACE-Inhibitor (Treatment) | 0•14 | Cost data provided by Novo Nordisk |
| Aspirin (Treatment) | 0•16 | Cost data provided by Novo Nordisk |
| Statin (Treatment) | 0•48 | Cost data provided by Novo Nordisk |
| Beta Blocker (Treatment) | 0•07 | Cost data provided by Novo Nordisk |
| Calcium Channel Blocker (Treatment) | 0•11 | Cost data provided by Novo Nordisk |
| Clopidogrel (Treatment) | 0•44 | Cost data provided by Novo Nordisk |
| Standard lifestyle change (Treatment) | 0•37 |  |
| Thiazide Diuretic (Treatment) | 0•16 | Cost data provided by Novo Nordisk |
| Glitazone (Treatment) | 2•11 | Cost data provided by Novo Nordisk |
| Hemodialysis (Treatment) | 129•14 | Cost data provided by Novo Nordisk |
| Insulin (Treatment) | 1•30 | Cost data provided by Novo Nordisk |
| Lasix (Treatment) | 0•14 | Cost data provided by Novo Nordisk |
| LMW Heparin (Treatment) | 0•84 |  |
| Metformin (Treatment) | 0•26 | Cost data provided by Novo Nordisk |
| Smoking Cessation (cost per quitter, Treatment) | 472•00 | (79-80) |
| Sulfonylurea (Treatment) | 0•07 | Cost data provided by Novo Nordisk |
| Warfarin (Treatment) | 0•33 | Cost data provided by Novo Nordisk |
| Intensive lifestyle advice (Treatment) | 0•37 | (81) |

Table S15. Cost assumptions used for Germany.

| **Cost Event Name** | **Cost used for Italy (€)** | **Reference** |
| --- | --- | --- |
| Cardiac Catheterization (Ambulatory) | 2110•18 |  |
| Ambulatory visit cost | 183•15 |  |
| Laser Photocoagulation (Outpatient) | 131•83 | (100) |
| FPG Test (Outpatient) | 15•54 |  |
| Full Lipid Panel (Outpatient) | 25•55 |  |
| HbA1c Test (Outpatient) | 12•66 | Cost data provided by Novo Nordisk |
| Ketones Test (Outpatient) | 10•03 |  |
| Serum Creatinine Test (Outpatient) | 16•28 |  |
| Urinary Albumin Test (Outpatient) | 15•97 |  |
| Outpatient visit cost | 22•85 | (100) |
| CABG (Inpatient) | 26628•67 |  |
| Cardiac catheterization (Inpatient) | 10481•47 |  |
| Diabetes amputation (Inpatient) | 8956•91 | (100) |
| Diabetes DKA (Inpatient) | 2373•66 | (100) |
| Kidney transplant (Inpatient) | 30002•73 | (101) |
| MI (Inpatient) | 13247•20 | (102-103) |
| PCI (Inpatient) | 10867•33 |  |
| Renal failure (Inpatient) | 6584•99 |  |
| Stable angina (Inpatient) | 13247•20 | (102-103) |
| Stroke (Inpatient) | 18032•74 | (104) |
| Unstable angina (Inpatient) | 13247•20 | (102-103) |
| Chest pain (Inpatient) | 13247•20 | (102-103) |
| Atrial Fibrillation (annual cost) | 12621•08 |  |
| CHF (annual cost) | 1768•13 | (105) |
| Foot Ulcer (one-time cost) | 342•06 | (87) |
| Stroke (annual cost) | 1382•30 | (104) |
| ACE-Inhibitor (Treatment) | 0•34 | Cost data provided by Novo Nordisk |
| Aspirin (Treatment) | 0•12 | Cost data provided by Novo Nordisk |
| Statin (Treatment) | 1•03 | Cost data provided by Novo Nordisk |
| Beta Blocker (Treatment) | 0•12 | Cost data provided by Novo Nordisk |
| Calcium Channel Blocker (Treatment) | 0•20 | Cost data provided by Novo Nordisk |
| Clopidogrel (Treatment) | 0•57 | Cost data provided by Novo Nordisk |
| Standard lifestyle change (Treatment) | 1•18 |  |
| Thiazide Diuretic (Treatment) | 0•87 | Cost data provided by Novo Nordisk |
| Glitazone (Treatment) | 1•39 | Cost data provided by Novo Nordisk |
| Hemodialysis (Treatment) | 127•38 | Cost data provided by Novo Nordisk |
| Insulin (Treatment) | 0•89 | Cost data provided by Novo Nordisk |
| Lasix (Treatment) | 0•06 | Cost data provided by Novo Nordisk |
| LMW Heparin (Treatment) | 2•64 |  |
| Metformin (Treatment) | 0•12 | Cost data provided by Novo Nordisk |
| Smoking Cessation (cost per quitter, Treatment) | 558•45 | (79-80) |
| Sulfonylurea (Treatment) | 0•09 | Cost data provided by Novo Nordisk |
| Warfarin (Treatment) | 0•07 | Cost data provided by Novo Nordisk |
| Intensive lifestyle advice (Treatment) | 1•18 | (81) |

Table S16. Cost assumptions used for Italy.

| **Cost Event Name** | **Cost used for Poland (€)** | **Reference** |
| --- | --- | --- |
| Cardiac Catheterization (Ambulatory) | 416•59 |  |
| Ambulatory visit cost | 36•16 |  |
| Laser Photocoagulation (Outpatient) | 176•03 | Cost data provided by Novo Nordisk |
| FPG Test (Outpatient) | 3•07 |  |
| Full Lipid Panel (Outpatient) | 5•04 |  |
| HbA1c Test (Outpatient) | 4•97 |  |
| Ketones Test (Outpatient) | 1•98 |  |
| Serum Creatinine Test (Outpatient) | 3•21 |  |
| Urinary Albumin Test (Outpatient) | 3•15 |  |
| Outpatient visit cost | 6•25 | Cost data provided by Novo Nordisk |
| CABG (Inpatient) | 5257•06 |  |
| Cardiac catheterization (Inpatient) | 2069•26 |  |
| Diabetes amputation (Inpatient) | 1733•70 | Cost data provided by Novo Nordisk |
| Diabetes DKA (Inpatient) | 1060•99 | Cost data provided by Novo Nordisk |
| Kidney transplant (Inpatient) | 19733•06 | Cost data provided by Novo Nordisk |
| MI (Inpatient) | 1634•24 | Cost data provided by Novo Nordisk |
| PCI (Inpatient) | 2145•44 |  |
| Renal failure (Inpatient) | 1300•01 |  |
| Stable angina (Inpatient) | 988•63 | Cost data provided by Novo Nordisk |
| Stroke (Inpatient) | 1695•50 | Cost data provided by Novo Nordisk |
| Unstable angina (Inpatient) | 988•63 | Cost data provided by Novo Nordisk |
| Chest pain (Inpatient) | 988•63 | Cost data provided by Novo Nordisk |
| Atrial Fibrillation (annual cost) | 2491•66 |  |
| CHF (annual cost) | 819•61 | Cost data provided by Novo Nordisk |
| Foot Ulcer (one-time cost) | 35•26 | Cost data provided by Novo Nordisk |
| Stroke (annual cost) | 1789•63 | Cost data provided by Novo Nordisk |
| ACE-Inhibitor (Treatment) | 0•01 | Cost data provided by Novo Nordisk |
| Aspirin (Treatment) | 0•000 | Cost data provided by Novo Nordisk |
| Statin (Treatment) | 0•20 | Cost data provided by Novo Nordisk |
| Beta Blocker (Treatment) | 0•01 | Cost data provided by Novo Nordisk |
| Calcium Channel Blocker (Treatment) | 0•02 | Cost data provided by Novo Nordisk |
| Clopidogrel (Treatment) | 0•18 | Cost data provided by Novo Nordisk |
| Standard lifestyle change (Treatment) | 0•14 |  |
| Thiazide Diuretic (Treatment) | 0•02 | Cost data provided by Novo Nordisk |
|  | 0•14 |  |
| Glitazone (Treatment) | 0•34 |  |
| Hemodialysis (Treatment) | 27•78 | Cost data provided by Novo Nordisk |
| Insulin (Treatment) | 0•66 | Cost data provided by Novo Nordisk |
| Lasix (Treatment) | 0•00 | Cost data provided by Novo Nordisk |
| LMW Heparin (Treatment) | 0•31 |  |
| Metformin (Treatment) | 0•04 | Cost data provided by Novo Nordisk |
| Smoking Cessation (cost per quitter, Treatment) | 110•25 | (79-80) |
| Sulfonylurea (Treatment) | 0•00 | Cost data provided by Novo Nordisk |
| Warfarin (Treatment) | 0•10 | Cost data provided by Novo Nordisk |
| Intensive lifestyle advice (Treatment) | 0•14 | (81) |

Table S17. Cost assumptions used for Poland.

| **Cost Event Name** | **Cost used for UK (€)** | **Reference** |
| --- | --- | --- |
| Cardiac Catheterization (Ambulatory) | 975•62 |  |
| Ambulatory visit cost | 84•68 |  |
| Laser Photocoagulation (Outpatient) | 320•75 | (106) |
| FPG Test (Outpatient) | 7•18 |  |
| Full Lipid Panel (Outpatient) | 11•81 |  |
| HbA1c Test (Outpatient) | 11•65 |  |
| Ketones Test (Outpatient) | 4•64 |  |
| Serum Creatinine Test (Outpatient) | 7•53 |  |
| Urinary Albumin Test (Outpatient) | 7•38 |  |
| Outpatient visit cost | 35•48 | (107) |
| CABG (Inpatient) | 12311•50 |  |
| Cardiac catheterization (Inpatient) | 4846•00 |  |
| Diabetes amputation (Inpatient) | 4914•77 | (106) |
| Diabetes DKA (Inpatient) | 1102•55 | (108) |
| Kidney transplant (Inpatient) | 24793•66 | (109) |
| MI (Inpatient) | 5006•84 | (110) |
| PCI (Inpatient) | 5024•40 |  |
| Renal failure (Inpatient) | 3044•50 |  |
| Stable angina (Inpatient) | 1329•68 | (111) (112) |
| Stroke (Inpatient) | 7613•06 | (113) |
| Unstable angina (Inpatient) | 1329•68 | (111) (112) |
| Chest pain (Inpatient) | 1329•68 | (111) (7) |
| Atrial Fibrillation (annual cost) | 5835•23 |  |
| CHF (annual cost) | 2789•55 | (113) |
| Foot Ulcer (one-time cost) | 284•43 | (87) |
| Stroke (annual cost) | 2799•09 | (113) |
| ACE-Inhibitor (Treatment) | 0•05 | (114) |
| Aspirin (Treatment) | 0•01 | (114) |
| Statin (Treatment) | 0•26 | (114) |
| Beta Blocker (Treatment) | 0•03 | (114) |
| Calcium Channel Blocker (Treatment) | 0•04 | (114) |
| Clopidogrel (Treatment) | 0•07 | (114) |
| Standard lifestyle change (Treatment) | 0•18 |  |
| Thiazide Diuretic (Treatment) | 0•03 | (114) |
| Glitazone (Treatment) | 1•42 | (114) |
| Hemodialysis (Treatment) | 114•95 | (114) |
| Insulin (Treatment) | 0•68 | (114) |
| Lasix (Treatment) | 0•05 |  |
| LMW Heparin (Treatment) | 0•41 |  |
| Metformin (Treatment) | 0•03 | (114) |
| Smoking Cessation (cost per quitter, Treatment) | 248•10 | (79-80) |
| Sulfonylurea (Treatment) | 0•05 | (114) |
| Warfarin (Treatment) | 0•04 | (114) |
| Intensive lifestyle advice (Treatment) | 0•18 | (81) |

Table S18. Cost assumptions used for the United Kingdom.

# Section B: Description of the *generic risk test* used for pre-screening

- The assessment tool used to target individuals for the Health Check must be non-invasive (e.g. not require any biochemical measurements), so that risk may be evaluated with data available in general practice databases or from individuals themselves.
- Our study aims to evaluate the Health Check, not to develop risk tools for each country.
- Therefore, we employed a *generic risk test*, to show the likely cost effectiveness of a targeted Health Check strategy, were a suitable, validated tool to exist.
  - A real-world program could use the FINDRISC or Cambridge diabetes risk scores in countries where they have been validated
- Our *generic risk test* had a logistic functional form:

- With independent risk factors (X_i_):
  - Age, Gender, BMI, Waist, Smoking, Family History of Diabetes, Family History of CHD, Antihypertensive Usage
  - The risk factors are similar to those of the FINDRISC and Cambridge risk scores
- The coefficients of the *generic risk test* were obtained with a regression on the presence of undiagnosed diabetes at baseline OR 10-year risk of MI, stroke, or CVD Death on a population cross section of adults ages 20-85.
  - The regression was performed on half of the simulated Danish population.
  - The coefficients from the regression are not published because the score is only intended for use within the Archimedes Model.
- The performance of the *generic risk test* in detecting undiagnosed diabetes at baseline or the ten-year occurrence of myocardial infarction, stroke, or CV death was tested on the remaining half of the simulated Danish population cross-section, as well as the population cross-sections of the remaining five countries. The corresponding ROC curves are shown in Figure 1.


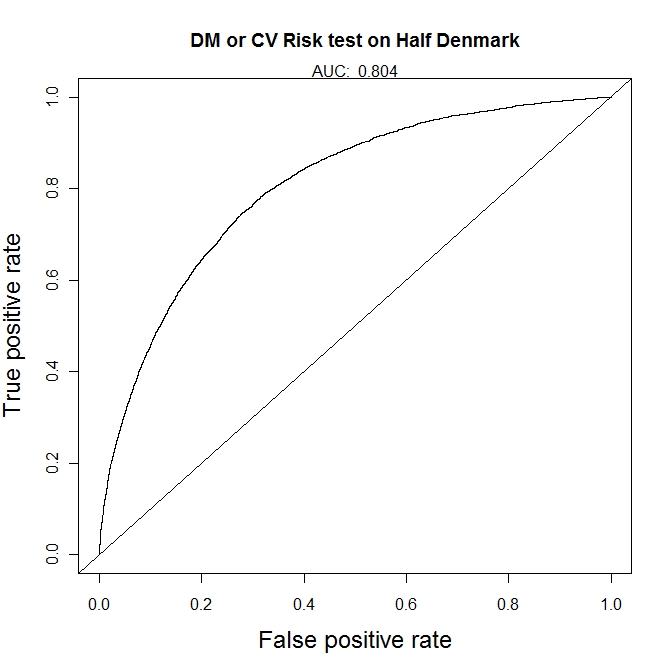

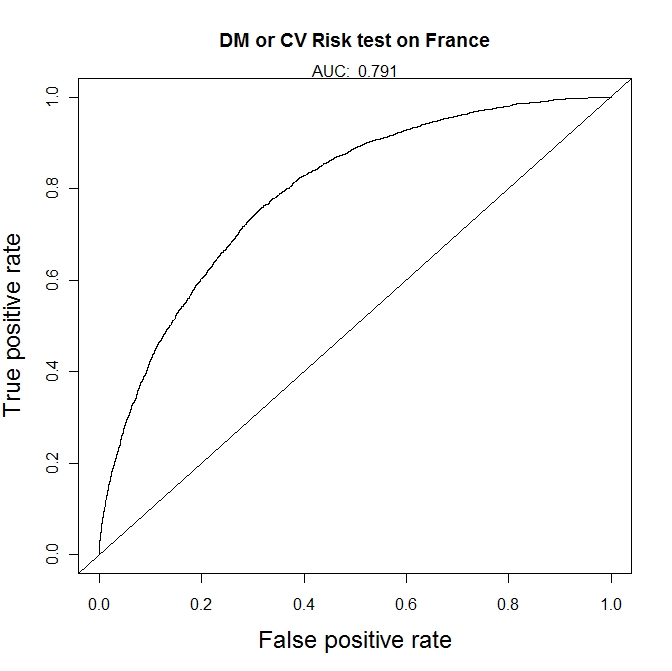


**
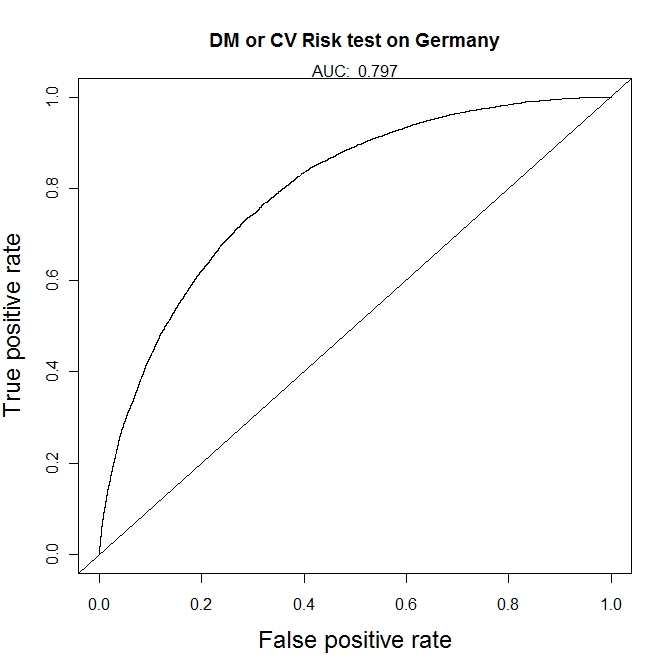

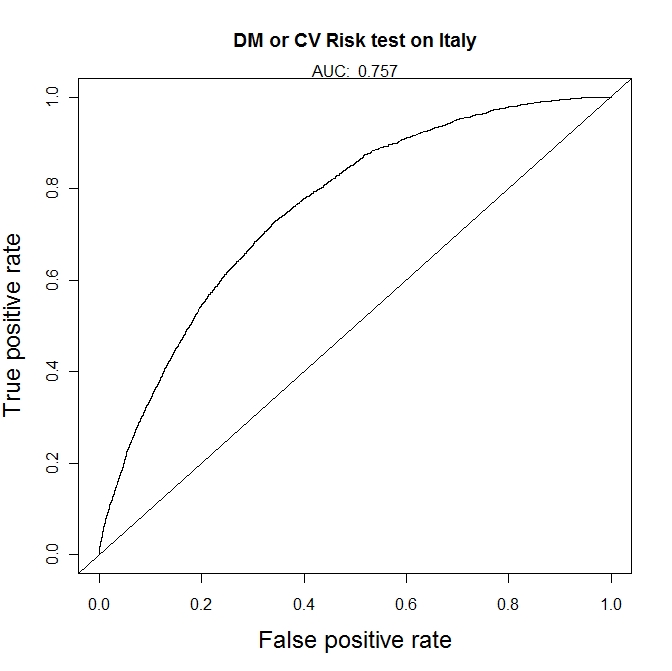
**

**
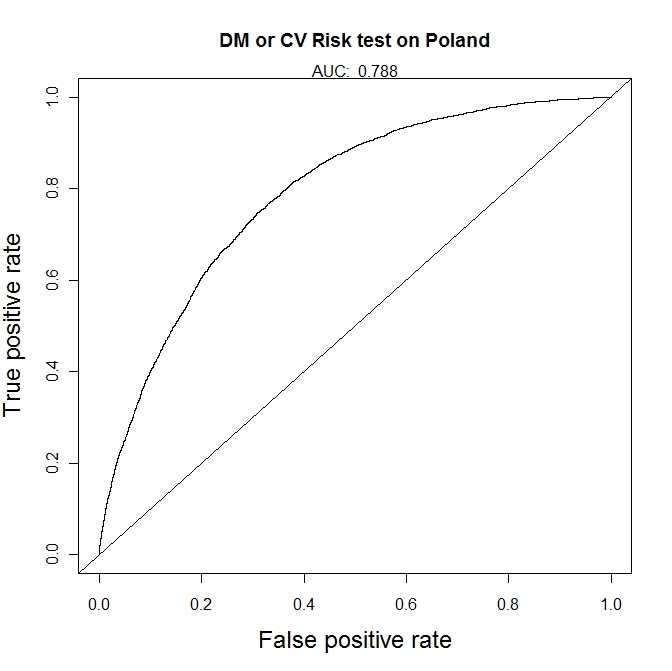
**
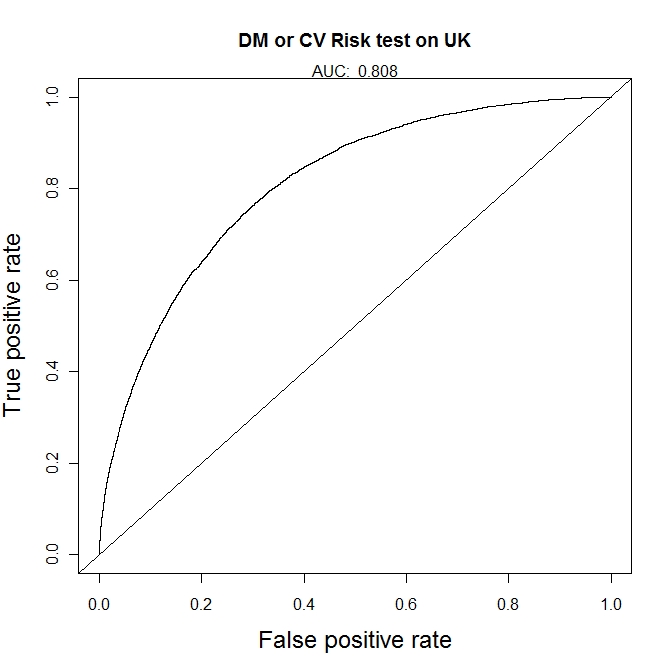


Figure S1. Receiver operating characteristic (ROC) curves showing the discrimination of the *generic risk test* on each simulated population.

**1. OECD Statistics. Available from:** [**http://stats.oecd.org/Index.aspx?QueryId=23066**](http://stats.oecd.org/Index.aspx?QueryId=23066)**.**

**2. Danmarks Statistik.**

**3. Glumer C, Carstensen B, Sandbaek A, Lauritzen T, Jorgensen T, Borch-Johnsen K. A Danish Diabetes Risk Score for Targeted Screening. Diabetes Care. 2004;27(3):727-33.**

**4. Dansk Nefrologisk Selskab, Rapport for Danmark 2005.**

**5. Glumer C, Jorgensen T, Borch-Johnsen K. Prevalences of Diabetes and Impaired Glucose Regulation in a Danish Population. Diabetes Care. 2003 August 2003;26(8):2335-40.**

**6. Hjertestatistik. 2004.**

**7. Statistik over behandling med blodtryksmidler, Lægemiddelstyrelsen 2009.**

**8. MedStat. Available from: Medstat.dk**

**9. Dansk Nefrologisk Selskab, Rapport for Danmark 2005.**

**10. Schulze og Schroeder: Basisbog i Sygdomslære, Munksgaard 2007. 2007.**

**11. Det Nationale Diabetes Register 2007.**

**12. Dødsårsagsregistret. 2009.**

**13. Sundhedsprofil. 2010; Available from: sundhedsprofil2010.dk.**

**14. Det Nationale Indikatorprojekt (NIP).**

**15. Cohort of people with Type II DM from Copenhagen: Det Nationale Indikatorprojekt (NIP).**

**16. INSEE. 2010; Available from:** [**http://www.insee.fr/fr/themes/detail.asp?ref_id=bilan-demo&page=donnees-detaillees/bilan-demo/pop_age2.htm**](http://www.insee.fr/fr/themes/detail.asp?ref_id=bilan-demo&page=donnees-detaillees/bilan-demo/pop_age2.htm)**.**

**17. Castebon. Dietary intake, physical activity and nutritional status in adults: the French nutrition and health survey Br J Nutr. 2006;102:733-43.**

**18. Ricci P. Diabète traité : quelles évolutions entre 2000 et 2009 en France? BEH. 2010(42-43):425-31.**

**19. Cambou J, Ferrières J, Ruidavets J, Ducimetière P. Épidémiologie à l’échelle européenne et française de l’infarctus du myocarde. Données du projet MONICA. Arch Mal Coeur 1996;89(13).**

**20. Allonier C, Dourgnon P, Sante RT. soins et protection sociale en 2004. Enquête Santé et Protection Sociale 2006.**

**21. Ndong J-R. Caractéristiques, risque vasculaire, complications et qualité des soins des personnes diabétiques dans les départements d’outre-mer et comparaison à la métropole BEH. 2010;42-43:432-6.**

**22. Tourdiman M. Archives des maladies du coeur et des vaisseaux.100:615-9.**

**23. Incidence à 10 ans de l’HTA dans la population générale : rôle des paramètres démographiques et cliniques, et implication pour la surveillance des normotendus.**

**24. Couchod C. REIN annual report 2005. Renal Epidemiology and Information Network & Agence de la biomédecine. 2005.**

**25. Nephrol Ther. 2007(Suppl 1):S1-82.**

**26. Roger V. Heart Disease and Stroke Statistics--2011 Update : A Report From the American Heart Association. 2011.**

**27. Fagot-Campagna A. The ENTRED Study demonstrates improvements in diabetes care process indicators among people treated with oral antidiabetic agents, France. 1998-2001.**

**28. Droumaguet C. Use of HbA1c in Predicting Progression to Diabetes in French Men and Women. Diabetes Care. 2006;29(7):1619-25.**

**29. Data from an Epidemiological Study on the Insulin Resistance Syndrome (DESIR)**

**30. Ducimetière P. Five-year incidence of angina pectoris and other forms of coronary heart disease in healthy men aged 50-59 in France and Northern Ireland: the Prospective Epidemiological Study of Myocardial Infarction (PRIME) Study. Int J Epidemiol. 2001 Oct. 2001;30(5):6.**

**31. Moebus S, Balijepalli C. Age- and sex-specific prevalence and ten-year risk for cardiovascular disease of all 16 risk factor combinations of the metabolic syndrome - A cross-sectional study. Cardiovascular Diabetology. 2010;9(34).**

**32. Kanavos P, Aardweg Svd, Schurer W. Diabetes expenditure, burden of disease and management in 5 EU countries**

**LSE.**

**33. Physician-diagnosed high blood fat levels in the last 12 months (percentage of respondents in percent). Classification: years, region, age, gender, education. Available from:** [**http://translate.googleusercontent.com/translate_c?act=url&hl=en&ie=UTF8&prev=_t&rurl=translate.google.com&sl=auto&tl=en&u=http://www.gbe-bund.de/oowa921-install/servlet/oowa/aw92/dboowasys921.xwdevkit/xwd_init%3Fgbe.isgbetol/xs_start_neu/%26p_aid%3Di%26p_aid%3D27256493%26nummer%3D763%26p_sprache%3DD%26p_indsp%3D-%26p_aid%3D42303119&usg=ALkJrhhI-TxwnN4KjccUlje6aF5SSNcuBw**](http://translate.googleusercontent.com/translate_c?act=url&hl=en&ie=UTF8&prev=_t&rurl=translate.google.com&sl=auto&tl=en&u=http://www.gbe-bund.de/oowa921-install/servlet/oowa/aw92/dboowasys921.xwdevkit/xwd_init%3Fgbe.isgbetol/xs_start_neu/%26p_aid%3Di%26p_aid%3D27256493%26nummer%3D763%26p_sprache%3DD%26p_indsp%3D-%26p_aid%3D42303119&usg=ALkJrhhI-TxwnN4KjccUlje6aF5SSNcuBw)**.**

**34. Known high blood pressure or hypertension (percentage of respondents in percent). Available from:** [**http://translate.google.com/translate?sl=de&tl=en&js=n&prev=_t&hl=en&ie=UTF-8&layout=2&eotf=1&u=http%3A%2F%2Fwww.gbe-bund.de%2Foowa921-install%2Fservlet%2Foowa%2Faw92%2Fdboowasys921.xwdevkit%2Fxwd_init%3Fgbe.isgbetol%2Fxs_start_neu%2F%26p_aid%3Di%26p_aid%3D7465483%26nummer%3D750%26p_sprache%3DD%26p_indsp%3D-%26p_aid%3D46909008&act=url**](http://translate.google.com/translate?sl=de&tl=en&js=n&prev=_t&hl=en&ie=UTF-8&layout=2&eotf=1&u=http%3A%2F%2Fwww.gbe-bund.de%2Foowa921-install%2Fservlet%2Foowa%2Faw92%2Fdboowasys921.xwdevkit%2Fxwd_init%3Fgbe.isgbetol%2Fxs_start_neu%2F%26p_aid%3Di%26p_aid%3D7465483%26nummer%3D750%26p_sprache%3DD%26p_indsp%3D-%26p_aid%3D46909008&act=url)**.**

**35. Pharmaceuticals prescribed at the expense of the statutory health insurance. Available from: https://**[**www.gbe-bund.de/oowa921-install/servlet/oowa/aw92/dboowasys921.xwdevkit/xwd_init?gbe.isgbetol/xs_start_neu/&p_aid=i&p_aid=16033748&nummer=613&p_sprache=E&p_indsp=-&p_aid=81228542**](http://www.gbe-bund.de/oowa921-install/servlet/oowa/aw92/dboowasys921.xwdevkit/xwd_init?gbe.isgbetol/xs_start_neu/&p_aid=i&p_aid=16033748&nummer=613&p_sprache=E&p_indsp=-&p_aid=81228542)**.**

**36. Antidiabetic drugs that were prescribed by the statutory health insurance. Available from:** [**http://translate.google.com/translate?sl=de&tl=en&js=n&prev=_t&hl=en&ie=UTF-8&layout=2&eotf=1&u=http%3A%2F%2Fwww.gbe-bund.de%2Foowa921-install%2Fservlet%2Foowa%2Faw92%2Fdboowasys921.xwdevkit%2Fxwd_init%3Fgbe.isgbetol%2Fxs_start_neu%2F%26p_aid%3D3%26p_aid%3D27944252%26nummer%3D348%26p_sprache%3DD%26p_indsp%3D-%26p_aid%3D40268785&act=url**](http://translate.google.com/translate?sl=de&tl=en&js=n&prev=_t&hl=en&ie=UTF-8&layout=2&eotf=1&u=http%3A%2F%2Fwww.gbe-bund.de%2Foowa921-install%2Fservlet%2Foowa%2Faw92%2Fdboowasys921.xwdevkit%2Fxwd_init%3Fgbe.isgbetol%2Fxs_start_neu%2F%26p_aid%3D3%26p_aid%3D27944252%26nummer%3D348%26p_sprache%3DD%26p_indsp%3D-%26p_aid%3D40268785&act=url)**.**

**37. Chronic renal failure, Chapter 5:23 [Health Report for Germany, 1998]. Available from:** [**http://translate.googleusercontent.com/translate_c?act=url&hl=en&ie=UTF8&prev=_t&rurl=translate.google.com&sl=de&tl=en&u=http://www.gbe-bund.de/gbe10/ergebnisse.prc_tab%3Ffid%3D939%26suchstring%3D%26query_id%3D%26sprache%3DD%26fund_typ%3DTXT%26methode%3D%26vt%3D%26verwandte%3D1%26page_ret%3D0%26seite%3D1%26p_lfd_nr%3D1%26p_news%3D%26p_sprachkz%3DD%26p_uid%3Dgast%26p_aid%3D60976292%26hlp_nr%3D2%26p_janein%3DJ&usg=ALkJrhg6x6ti4hjdNmwODs9HZ4ARqLaClg**](http://translate.googleusercontent.com/translate_c?act=url&hl=en&ie=UTF8&prev=_t&rurl=translate.google.com&sl=de&tl=en&u=http://www.gbe-bund.de/gbe10/ergebnisse.prc_tab%3Ffid%3D939%26suchstring%3D%26query_id%3D%26sprache%3DD%26fund_typ%3DTXT%26methode%3D%26vt%3D%26verwandte%3D1%26page_ret%3D0%26seite%3D1%26p_lfd_nr%3D1%26p_news%3D%26p_sprachkz%3DD%26p_uid%3Dgast%26p_aid%3D60976292%26hlp_nr%3D2%26p_janein%3DJ&usg=ALkJrhg6x6ti4hjdNmwODs9HZ4ARqLaClg)**.**

**38. Diagnostic data of the hospitals starting from 2000. Available from: https://**[**www.gbe-bund.de/oowa921-install/servlet/oowa/aw92/dboowasys921.xwdevkit/xwd_init?gbe.isgbetol/xs_start_neu/&p_aid=3&p_aid=76225239&nummer=594&p_sprache=E&p_indsp=104&p_aid=50493337**](http://www.gbe-bund.de/oowa921-install/servlet/oowa/aw92/dboowasys921.xwdevkit/xwd_init?gbe.isgbetol/xs_start_neu/&p_aid=3&p_aid=76225239&nummer=594&p_sprache=E&p_indsp=104&p_aid=50493337)**.**

**39. Rathmann W, Strassburger K, Heier M, Holle R, Thorand B, Giani G, et al. Incidence of Type 2 diabetes in the elderly German population and the effect of clinical and lifestyle risk factors: KORA S4/F4 cohort study. Diabet Med. 2009 Dec 2009;26(12):8.**

**40. Anderson JL, Adams CD, Antman EM, Bridges CR, Califf RM, Casey DE, Jr., et al. ACC/AHA 2007 guidelines for the management of patients with unstable angina/non ST-elevation myocardial infarction: a report of the American College of Cardiology/American Heart Association Task Force on Practice Guidelines (Writing Committee to Revise the 2002 Guidelines for the Management of Patients With Unstable Angina/Non ST-Elevation Myocardial Infarction): developed in collaboration with the American College of Emergency Physicians, the Society for Cardiovascular Angiography and Interventions, and the Society of Thoracic Surgeons: endorsed by the American Association of Cardiovascular and Pulmonary Rehabilitation and the Society for Academic Emergency Medicine. Circulation. 2007 Aug 14;116(7):e148-304.**

**41. Distribution of the population to groups in terms of smoking habits in percent. Available from: https://**[**www.gbe-bund.de/oowa921-install/servlet/oowa/aw92/dboowasys921.xwdevkit/xwd_init?gbe.isgbetol/xs_start_neu/&p_aid=i&p_aid=15564488&nummer=436&p_sprache=E&p_indsp=-&p_aid=40615154**](http://www.gbe-bund.de/oowa921-install/servlet/oowa/aw92/dboowasys921.xwdevkit/xwd_init?gbe.isgbetol/xs_start_neu/&p_aid=i&p_aid=15564488&nummer=436&p_sprache=E&p_indsp=-&p_aid=40615154)**.**

**42. WHO Global Infobase. [Oct 17, 2011]; Available from: https://apps.who.int/infobase/Indicators.aspx.**

**43. Icks A, Rathmann W, Haastert B, Mielck A, Holle R, Lowel H, et al. Quality of care and extent of complications in a population-based sample of patients with type 2 diabetes mellitus. The KORA Survey 2000. Dtsch Med Wochenschr. Jan 2006;131(3):6.**

**44. Liebl A, Neiss A, Spannheimer A, Reitberger U, Wieseler B, Stammer H, et al. Complications, co-morbidity, and blood glucose control in type 2 diabetes mellitus patients in Germany--results from the CODE-2 study. Exp Clin Endocrinol Diabetes. 2002;110(1).**

**45. Liebl A, Neiss A, Spannheimer A, Reitberger U, Wieseler B, Stammer H, et al. Complications, co-morbidity, and blood glucose control in type 2 diabetes mellitus patients in Germany--results from the CODE-2 study. Exp Clin Endocrinol Diabetes. 2002;110(1).**

**46. Trautner C, Icks A, Haastert B, Plum F, Berger M. Incidence of blindness in relation to diabetes. A population-based study. Diabetes Care. 1997;20(7):1147-53.**

**47. Icks A, Scheer M, Genz J, Giani G, Glaeske G, Hoffmann F. Stroke in the diabetic and non-diabetic population in Germany: relative and attributable risks, 2005-2007. J Diabetes Complications. 2011;25(2).**

**48. United Nations, Department of Economic and Social Affairs, Population Division (2011). World Population Prospects: The 2010 Revision, CD-ROM Edition.2011.**

**49. The Italian Atlas of Cardiovascular Diseases, 2nd edition 2004, National Center of Epidemiology, Surveillance and Health Promotion, Italian Institute of Health, Rome, 2004. 2004.**

**50. Sistema di Sorveglianza PASSI**

**(Progressi delle Aziende Sanitarie**

**per la Salute in Italia):**

**risultati 2007. 2007.**

**51. Indicators of disease - Incidence. 2011 [Oct. 17, 2011]; Available from:** [**http://www.cuore.iss.it/indicatori/incidenza.asp**](http://www.cuore.iss.it/indicatori/incidenza.asp)**.**

**52. 2008 USRDS Annual Data Report 2008 [Oct 17, 2011]; Available from:** [**http://www.usrds.org/2008/view/esrd_12.asp**](http://www.usrds.org/2008/view/esrd_12.asp)**.**

**53. VI Report Health Search (2009-2010) (GPs database). 2009-10.**

**54. Monesi L. Prevalence, incidence and mortality of diagnosed diabetes: evidence from an Italian population-based study. Diabet Med. 2012 Mar 2012;29(3):8.**

**55. Diabetes Care. 2007;30:1241-47.**

**56. Stroke 2007;38:1154-1160.**

**57. AMD Annals 2009 – Longitudinal analysis of quality indicators of diabetes care in Italy. 2009 [19 October 2011]; Available from:** [**http://www.changingdiabetesbarometeritaly.com**](http://www.changingdiabetesbarometeritaly.com) **/pdf/aprile-2010/2009_annali_AMD_eng.pdf**

**58. United Nations, Department of Economic and Social Affairs, Population Division (2011). World Population Prospects: The 2010 Revision, CD-ROM Edition. 2011.**

**59. ; Available from:** [**www.laeger.dk**](http://www.laeger.dk)**.**

**60. Giorda CB. Incidence and Risk Factors for Stroke in Type 2 Diabetic Patients.**

**61. The relationship between physicians' self-reported target fasting blood glucose levels and metabolic control in type 2 diabetes. The QuED Study Group--quality of care and outcomes in type 2 diabetes. Diabetes Care. 2001.**

**62. Agenzia Italiana del Farmaco. Osservatorio sull'impiego dei medicinali (OsMED) Rapporto OSMED 20102010.**

**63. AMD Annals 2011 (diabetes centers database including over 450.000 patients; in Italy, over 60% of the patients are cared for by diabetes clinics). 2011.**

**64. Bonora E. Population-Based Incidence Rates and Risk Factors for Type 2 Diabetes in White Individuals: The Bruneck Study**

**65. International database. Available from:** [**http://www.census.gov/population/international/data/idb/informationGateway.php**](http://www.census.gov/population/international/data/idb/informationGateway.php)

**66. Wittek. Prevalence of Diabetes and Cardiovascular Risk Factors of Industrial Area in Southern Poland.**

**67. Malgorzata. Characteristics of patients with type 2 diabetes of short duration in Poland.**

**68. Health Survey for England, 2009. 2009.**

**69. Health Survey for England. Available from:** [**http://nesstar.esds.ac.uk/webview/index.jsp?v=2&mode=documentation&submode=abstract&study=http%3A%2F%2Fnesstar.esds.ac.uk%3A80%2Fobj%2FfStudy%2F6732&top=yes**](http://nesstar.esds.ac.uk/webview/index.jsp?v=2&mode=documentation&submode=abstract&study=http%3A%2F%2Fnesstar.esds.ac.uk%3A80%2Fobj%2FfStudy%2F6732&top=yes)**.**

**70. Table 2.1: Health Survey for England 2006: CVD and risk factors adults, obesity and risk factors children. Available from:** [**http://www.ic.nhs.uk/webfiles/publications/HSE06/HSE%2006%20report%20VOL%201%20v2.pdf**](http://www.ic.nhs.uk/webfiles/publications/HSE06/HSE%2006%20report%20VOL%201%20v2.pdf)**.**

**71. Health Survey for England: 2009. Available from:** [**http://www.ic.nhs.uk/statistics-and-data-collections/health-and-lifestyles-related-surveys/health-survey-for-england/health-survey-for-england--2009-trend-tables**](http://www.ic.nhs.uk/statistics-and-data-collections/health-and-lifestyles-related-surveys/health-survey-for-england/health-survey-for-england--2009-trend-tables)**.**

**72. Health Survey for England 2009: . Available from:** [**http://nesstar.esds.ac.uk/webview/index.jsp?v=2&mode=documentation&submode=abstract&study=http%3A%2F%2Fnesstar.esds.ac.uk%3A80%2Fobj%2FfStudy%2F6732&top=yes**](http://nesstar.esds.ac.uk/webview/index.jsp?v=2&mode=documentation&submode=abstract&study=http%3A%2F%2Fnesstar.esds.ac.uk%3A80%2Fobj%2FfStudy%2F6732&top=yes)**.**

**73. Medicare Current Beneficiary Survey (MCBS). 2002-2006.**

**74. Medicare Limited Data Set (LDS) 2008.**

**75. Medicare Part D Drug Data. 2006.**

**76. Danish Medicines Agency. Available from:** [**www.medicinpriser.dk**](http://www.medicinpriser.dk)**.**

**77. The Danish E-Health Portal. Available from:** [**www.sundhed.dk**](http://www.sundhed.dk)**.**

**78. National Board of Health Danish Centre for Evaluation and Health Technology Assessment. Dialysis in chronic renal failure, a health technology assessment. Copenhagen: National Board of Health, Danish Centre for Evaluation and Health Technology Assessment2006.**

**79. Lader D. Omnibus Survey Report No. 32: Smoking-related Behaviour and Attitudes, 2006. Newport: Office for National Statistics2007.**

**80. Ferguson J, Bauld L, Chesterman J, Judge K. The English smoking treatment services: one-year outcomes. Addiction. 2005 Apr;100 Suppl 2:59-69.**

**81. Tsai AG, Wadden TA. Systematic Review: An Evaluation of Major Commercial Weight Loss Programs in the United States. Ann Intern Med. 2005 January 2005;142(1):56-66.**

**82. Bonastre J, Le Pen C, Anderson P, Ganz A, Berto P, Berdeaux G. The epidemiology, economics and quality of life burden of age-related macular degeneration in France, Germany, Italy and the United Kingdom. Eur J Health Econ. 2002;3(2):94-102.**

**83. l'Assurance Maladie en ligne. Available from:** [**http://www.ameli.fr/assures/soins-et-remboursements/combien-serez-vous-rembourse/consultations/les-consultations-en-metropole/dans-le-cadre-du-parcours-de-soins-coordonnes.php**](http://www.ameli.fr/assures/soins-et-remboursements/combien-serez-vous-rembourse/consultations/les-consultations-en-metropole/dans-le-cadre-du-parcours-de-soins-coordonnes.php)**.**

**84. Statistiques PMSI, ATIH 2002-2011 Base Nationale Publique et Privée Available from:** [**http://stats.atih.sante.fr/cgi-bin/broker?_service=default&_program=mcoprog.affiche_cata.sas&debug=0&base=deux&typt=cim&annee=2009&niveau=4&codh=04020001%20**](http://stats.atih.sante.fr/cgi-bin/broker?_service=default&_program=mcoprog.affiche_cata.sas&debug=0&base=deux&typt=cim&annee=2009&niveau=4&codh=04020001%20)

**85. Journal Officiel de la République Française A N N E X E I TARIFS DES GHS ET DES SUPPLÉMENTS DES ÉTABLISSEMENTS DE SANTÉ MENTIONNÉS AUX a, b ET c DE L’ARTICLE L. 162-22-6 DU CODE DE LA SÉCURITÉ SOCIALE.**

**86. Colin X, Lafuma A, Gueron B. Costs of cardiovascular events of diabetic patients in the French hospitals. Diabetes Metab. 2007 Sep;33(4):310-3.**

**87. Ghatnekar O, Willis M, Persson U. Cost-effectiveness of treating deep diabetic foot ulcers with Promogran in four European countries. J Wound Care. 2002 Feb;11(2):70-4.**

**88. Holler D. Gesundheitsökonomische Aspekte der Versorgung chronisch Kranker am Beispiel der peripheren arteriellen Verschlusskrankheit. Eine Analyse aus Sicht der Gesellschaft und der Krankenversicherung. Versicherungswissenschaft in Hannover, Hannover Reihe, Band 23 (Verlag Versicherungswirtschaft GmbH, Karlsruhe).**

**89. National Association of Statutory Health Insurance Physicians, Doctor’s fee scale (EBM) 2011. 2011; Available from:** [**http://www.kbv.de/ebm2011/EBMGesamt.htm**](http://www.kbv.de/ebm2011/EBMGesamt.htm)**.**

**90. Hasslacher C, Wolf G, Kempe P, Ritz E. Diabetische Nephropathie 2008(Diabetologie und Stoffwechsel 2008; 3):S143-S6.**

**91. Hagen B, Altenhofen L, Haß W, Blaschy S, Kretschmann J. Qualitätssicherungsbericht 2009- Disease Management Programme in Nordrhein 2010**

**92. Scherbaum WA, Goodall G, Erny-Albrecht KM, Massi-Benedetti M, Erdmann E, Valentine WJ. Cost-effectiveness of pioglitazone in type 2 diabetes patients with a history of macrovascular disease: a German perspective. Cost Eff Resour Alloc. 2009;7:9.**

**93. InEK. G-DRG 2009/2011 Report-Browser. [cited 2011]; Available from:** [**http://www.g-drg.de/cms/G-DRG-System_2011/Abschlussbericht_zur_Weiterentwicklung_des_G-DRG-Systems_und_Report_Browser/Report-Browser_2009_2011**](http://www.g-drg.de/cms/G-DRG-System_2011/Abschlussbericht_zur_Weiterentwicklung_des_G-DRG-Systems_und_Report_Browser/Report-Browser_2009_2011)**.**

**94. Quelle. Available from:** [**http://www.brk.de/Ober-Mittelfranken/kreisverband-coburg/rettungsdienst/informationen/kosten/?searchterm=notarzteinsatz%20%20kosten**](http://www.brk.de/Ober-Mittelfranken/kreisverband-coburg/rettungsdienst/informationen/kosten/?searchterm=notarzteinsatz%20%20kosten)**.**

**95. Ray KK, Cannon CP, Cairns R, Morrow DA, Rifai N, Kirtane AJ, et al. Relationship between uncontrolled risk factors and C-reactive protein levels in patients receiving standard or intensive statin therapy for acute coronary syndromes in the PROVE IT-TIMI 22 trial. J Am Coll Cardiol. 2005;46(8):1417-24.**

**96. German Federal Insurance Office. Scientific report for the choice of 50 to 80 diseases to be considered in the disease-oriented risk structure compensation2007.**

**97. Brüggenjürgen B, Rupprecht H, Willich S, Spannagl H, Ehlken B, Smala A, et al. Cost of atherothrombotic diseases—myocardial infarction, ischaemic stroke and peripheral arterial occlusive disease—in Germany. Journal of Public Health. 2005;13:216-24.**

**98. Stollenwerk B, Stock S, Lauterbach K. Attributable KHK-Kosten und Einfluss der koronaren Herzkrankheit auf die Herzinsuffizienz. Deutsche Gesellschaft für Medizinische Informatik, Biometrie und Epidemiologie 50 Jahrestagung der Deutschen Gesellschaft für Medizinische Informatik, Biometrie und Epidemiologie (gmds), 12 Jahrestagung der Deutschen Arbeitsgemeinschaft für Epidemiologie2005.**

**99. Valentine WJ, Goodall G, Aagren M, Nielsen S, Palmer AJ, Erny-Albrecht K. Evaluating the cost-effectiveness of therapy conversion to insulin detemir in patients with type 2 diabetes in Germany: a modelling study of long-term clinical and cost outcomes. Adv Ther. 2008 Jun;25(6):567-84.**

**100. Ambulatory tariffs Lombardia. 2009; Available from:** [**http://www.codicesanita.info/default.aspx?Action=strumenti_sing&I0=a9e5bea6-4239-4a07-a2fe-c4e9955667ce**](http://www.codicesanita.info/default.aspx?Action=strumenti_sing&I0=a9e5bea6-4239-4a07-a2fe-c4e9955667ce)**.**

**101. Palmer AJ, Annemans L, Roze S, Lamotte M, Berto P, Ravera M, et al. Costo-efficacia di irbesartan in pazienti con diabete di tipo 2, ipertensione e nefropatia: prospettiva italiana. PharmacoEconomics Italian Research Articles. 2005;7(1):43-57 10.2165/00136178-200507010-00004.**

**102. Taylor MJ, Scuffham PA, McCollam PL, Newby DE. Acute coronary syndromes in Europe: 1-year costs and outcomes. Curr Med Res Opin. 2007 Mar;23(3):495-503.**

**103. Levy E, Gabriel S, Dinet J. The comparative medical costs of atherothrombotic disease in European countries. Pharmacoeconomics. 2003;21(9):651-9.**

**104. Capri S, Perlini S. Cost-effectiveness in Italy of preventive treatment with ramipril in patients at high risk of cardiovascular events. Curr Med Res Opin. 2005 Jun;21(6):913-21.**

**105. Politi C, Deales A, Cicchitelli F, Marcobelli A, Barbadoro P, Zorzan R, et al. Analisi dei costi sanitari per lo scompenso cardiaco nella regione Marche. PharmacoEconomics Italian Research Articles. 2005;7(3):165-75 10.2165/00136178-200507030-00002.**

**106. DH - PbR - Finance and Costing team. Reference Costs 2009-10 Publication: Department of Health2011.**

**107. Personal Social Services Research Unit. Unit Costs of Health and Social Care 2009/10; Available from:** [**http://www.pssru.ac.uk**](http://www.pssru.ac.uk)**.**

**108. Ray JA, Boye KS, Yurgin N, Valentine WJ, Roze S, McKendrick J, et al. Exenatide versus insulin glargine in patients with type 2 diabetes in the UK: a model of long-term clinical and cost outcomes. Curr Med Res Opin. 2007;23(3):609-22.**

**109. National Institute for Health and Clinical Excellence. Chronic kidney disease - NICE clinical guideline 73. London: National Institute for Health and Clinical Excellence2008.**

**110. National Institute for Health and Clinical Excellence. MI: secondary prevention - NICE clinical guideline 48. London: National Institute for Health and Clinical Excellence2007.**

**111. Dyer MT, Goldsmith KA, Khan SN, Sharples LD, Freeman C, Hardy I, et al. Clinical and cost-effectiveness analysis of an open label, single-centre, randomised trial of spinal cord stimulation (SCS) versus percutaneous myocardial laser revascularisation (PMR) in patients with refractory angina pectoris: The SPiRiT trial. Trials. 2008;9:40.**

**112. Cameron CG, Bennett HA. Cost-effectiveness of insulin analogues for diabetes mellitus. CMAJ. 2009 Feb 17;180(4):400-7.**

**113. National Institute for Health and Clinical Excellence. Statins for the prevention of cardiovascular events - Technology Appraisal 94. London: National Institute for Health and Clinical Excellence2008.**

**114. MIMS. Available from:** [**http://www.mims.co.uk/**](http://www.mims.co.uk/)**.**
