# Supplementary material for: A Standardized Vascular Disease Health Check in Europe: A Cost-Effectiveness Analysis
Source: PLoS One. 2013 Jul 15;8(7):e66454. doi: 10.1371/journal.pone.0066454 (PMC3712021; doi:10.1371/journal.pone.0066454)
Supplement: File S2 — Description of the model structure. (PDF) [file pone.0066454.s002.pdf]

DESCRIPTION OF THE ARCHIMEDES MODEL

ARCHeS Simulator 2.3

December 2011

David Eddy, MD, PhD
Marc-david Cohen, PhD

ARCHIMEDES
Quantifying Healthcare

Table of Contents

Introduction 5

Applications 6

Overview of the Model 7

Physiology Model 7

Population Model 11

Healthcare System Model 15

Outcomes Model 18

Executing the Model 19

Validating the Model 20

Physiology Model Variables 20

Identification and Selection of Data Sources 20

Disease Diagrams 21

Diabetes Metabolism Model 22

Type 2 Diabetes Progression Function 23

Fasting Plasma Glucose, Insulin Efficiency, and HbA1c 24

Effects on Other Disease Models 25

Diabetic Retinopathy Model 26

Risk Factors for Non-Proliferative Diabetic Retinopathy 27

Risk Factors for Proliferative Diabetic Retinopathy 27

Risk Factors for Diabetic Macular Edema 28

Blindness 28

Diabetic Neuropathy Model 28

Risk Factors for Sensory Neuropathy 29

Risk Factors for Foot Ulcer 29

Risk Factors for Amputation 30
Nephropathy Model 30
Risk Factors for Urinary Albumin 31
Risk Factors for Glomerular Filtration Rate 32
Coronary Artery Disease Model 32
Risk Factors for First Myocardial Infarction 34
Risk Factors for Unstable Angina 34
Risk Factors for Stable Angina 34
Circulation Model 35
Myocardial Damage Model 36
Chronic Atrial Fibrillation 38
Lipids Model 38
Stroke Model 39
First Ischemic Stroke 40
First Hemorrhagic Stroke 40
Recurrent Stroke 41
Mortality 42
Interventions 44
Antihypertensives 45
Antidiabetic Medications 47
Dyslipidemia Medications 49
Diabetes Diet 50
Healthcare System Model 50
Guidelines Implemented in the Model 50
Datasets Used for Model Calibration 53

Limitations of the Model 54

Acknowledgements 57

Sources of Funding 57

© 2011-2012

Introduction

The Archimedes Model is a simulation model of human physiology, patient populations, and healthcare systems. It creates a virtual world with simulated people, each of whom has a simulated anatomy and physiology. They get diseases, develop symptoms, seek care by scheduling appointments with their doctors or visiting emergency rooms, are seen by simulated physicians or other healthcare providers, are prescribed tests and treatments, choose whether or not to comply, and respond to the treatments. These events occur and recur continuously, as they do in real life, until the simulation ends or the patient dies.

This report is a non-technical description of the parts of the Model used in ARCHeS.

ARCHeS

ARCHeS (Archimedes Healthcare Simulator) is a web-based interface to the Archimedes Model. It enables users to set up, online, analyses using the ARCHeS Setup Tool, run the Model (also called the "Simulator"), and analyze the results using the ARCHeS Outcomes Analyzer tool. ARCHeS is the first publicly available version of ARCHeS[1]. It addresses risk factors, interventions, and outcomes related to cardio-metabolic risk, including diabetes and its complications, coronary artery disease, stroke, dyslipidemia, hypertension, obesity, and smoking. It can be used to analyze a wide range of questions about the occurrence and management of those conditions.

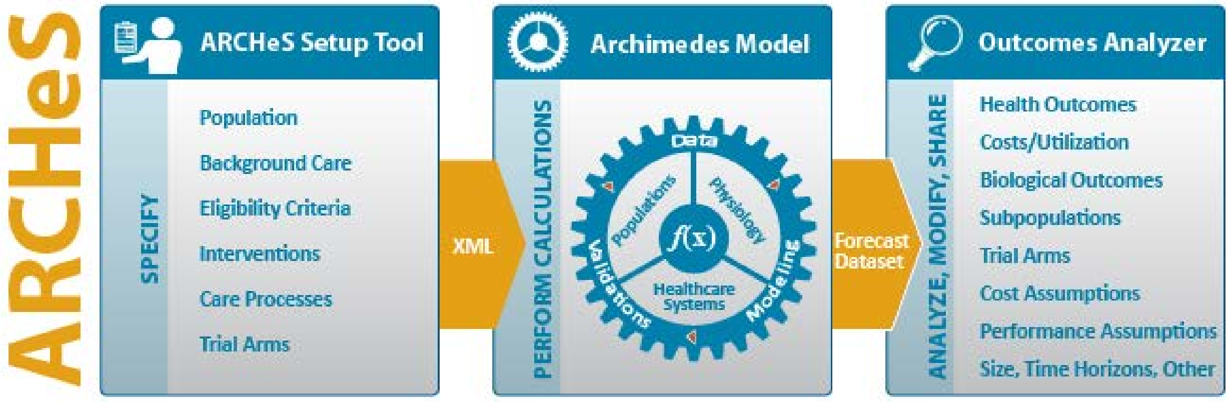

Figure 1. ARCHeS: Archimedes Healthcare Simulator.

Simulator 2.3

The Archimedes Model was designed for multiple applications and repeated use. To date, more than 100 analyses have been conducted using the Model. To enable application of the Model to multiple problems, many of them being conducted simultaneously, we maintain a "base version" of the Model,

[1] Future versions of ARCHeS will be periodically released as the Archimedes Model is improved to incorporate new science and evidence, and as the functionality of ARCHeS is increased.

or “Base Model,” that includes all the variables and equations needed to calculate a person’s physiology, the occurrence and outcomes of diseases, and the effects of tests and interventions. Many questions can be answered with the Base Model. When there is a need to conduct an analysis that cannot be done with the Base Model – e.g., it requires a higher level of physiological or pharmacological detail, involves emerging risk factors or novel interventions, or involves different settings and care processes – then appropriate parts of the Base Model are expanded or modified to address the new question. The modified, project-specific versions are saved for those particular analyses, but the Base Model is maintained unaltered. Over time, new science, technology, and evidence, and/or improvements in software may warrant changes to the Base Model. At that point, a new version of the Base Model is released. ARCHeS uses version 2.3 of the Base Model, called “Simulator 2.3.” This is the version of the Model that is described in this report.

Non-technical description

This report is a non-technical description of the parts of Simulator 2.3 that are accessed by ARCHeS. For convenience, in this report we will use the terms “ARCHeS Simulator 2.3,” and more simply “the Model,” to refer to these parts of the Archimedes Model, with the understanding that the full Archimedes Model includes other diseases that are not in ARCHeS, as well as other versions built using the Archimedes Modeling Framework. This report describes the types of applications ARCHeS is designed to address, the main parts of the Model, the variables and relationships in each of the disease sub-models, the sources used to build the disease sub-models, the sources used to build and calibrate the care processes for preventing or managing the diseases, and limitations of the Model. Thus this report describes *what* the Model does. Validation of Simulator 2.3 is described in the report “Validation Methodology and Results: ARCHeS Simulator 2.3.” All reports can be found on our website at www.archimedesmodel.com/tech-reports.

This report is intended for all audiences. Parties interested in a more technical description of the Model or ARCHeS can arrange for a technical, quantitative description by contacting us at www.archimedesmodel.com/contact. This report is not intended to be a manual or tutorial on ARCHeS. Additional information about ARCHeS and its functionality can be found on our website.

Applications

The Archimedes Model is designed to address questions at a variety of levels, including physiology, pharmacology, management of individual patients, and management of populations. For some case studies and examples of how the Archimedes Model has been applied, see www.archimedesmodel.com/case-studies. ARCHeS is designed to address questions relating to the management of cardio-metabolic risk in populations. Applications of ARCHeS include: forecasting, cost- and cost-effectiveness analysis, comparative-effectiveness analysis, priority setting, clinical trial design and prediction, performance improvement and incentives, guideline design, drug portfolio selection, the translation of efficacy (for example, clinical trial results) into effectiveness (results that can be expected in real settings), and the design of population-level policies.

© 2011-2012

Overview of the Model

The Archimedes Model is composed of four main components that work closely together: the physiology model, the population model, the healthcare system model, and the outcomes model (Figure 2). This section provides an overview of each component, focusing in particular on the variables and their relationships. Methods for validating the Model and the results of a standardized suite of validations are available in the report “Validation Methodology and Results: ARCHeS Simulator 2.3,” available at www.archimedesmodel.com/tech-reports

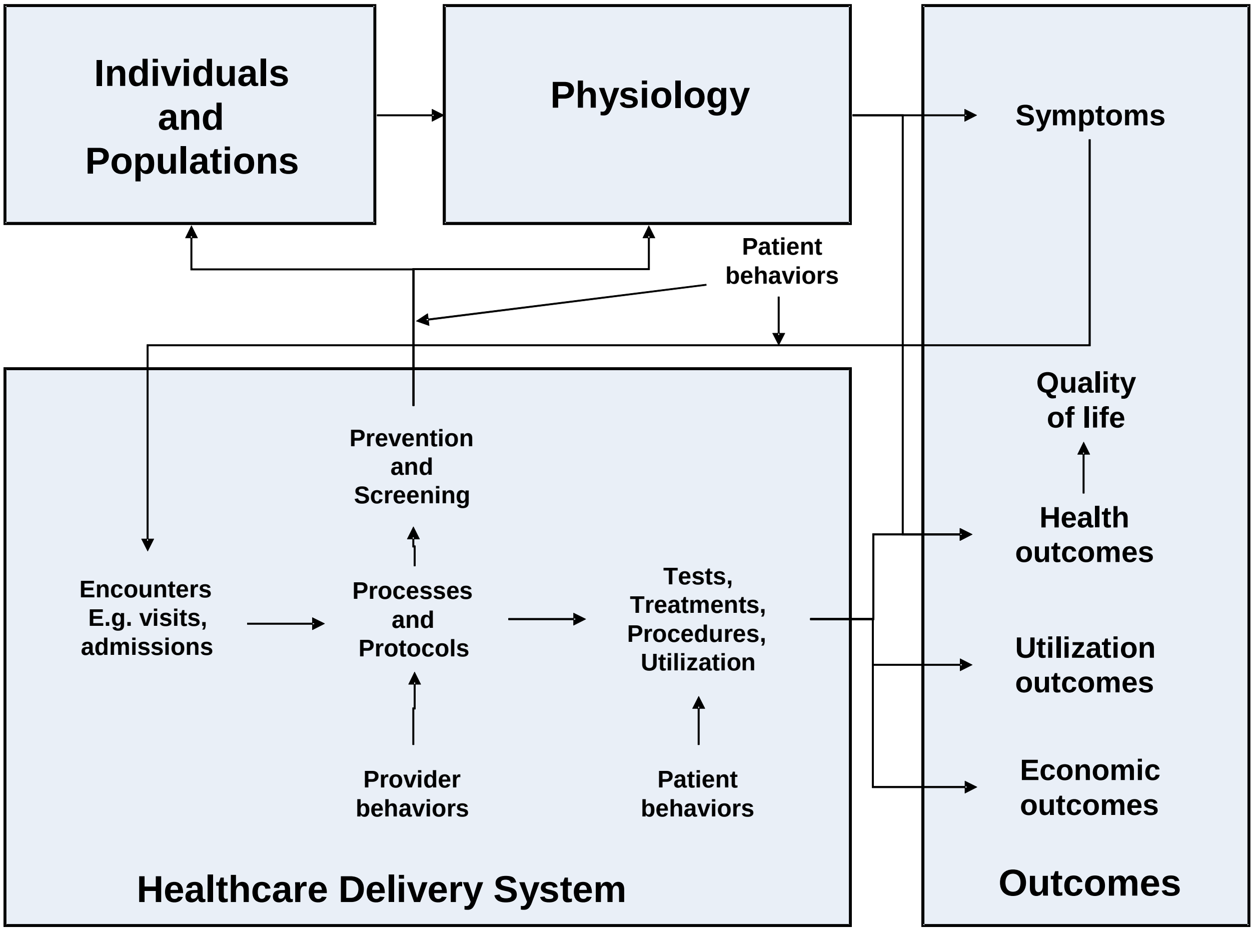

Figure 2. Components of the Archimedes Model.

Physiology Model

The physiology component is the foundation of the Model. It represents the physiology of each simulated person and causes them to get diseases, have symptoms, seek care, and so forth. It includes human anatomy, physiology, disease processes, the effects of interventions (e.g., preventive activities, tests, treatments, procedures), and outcomes.

Physiological variables

The fundamental building blocks are the physiological variables pertinent to clinical decisions and clinical policies. These other variables are considered by physicians and other providers when making decisions about individual patients, considered by those designing and interpreting clinical trials, and considered by those designing and applying policies such as guidelines, performance measures, and incentives. Thus the physiology model includes objects and variables that represent organs and their major parts, physiological variables and biomarkers (e.g. plasma glucose, various cholesterols, cardiac output), demographic variables, health-related behaviors, physical examination findings, past medical history, symptoms, interventions, and health outcomes. The particular variables in each disease sub-model are described below.

Level of detail

The particular variables included in the physiology model are determined by the questions the Model is intended to address. For example, because we want the Model to be able to analyze various weight-loss programs, the physiology model was built to include the variable "weight," each simulated person has a weight; weight can be measured in various ways (e.g., weight, body mass index (BMI)); a person's weight affects the progression of other variables (e.g. lipids, fasting plasma glucose (FPG); blood pressure), and a person's weight can be modified by interventions. The Model does not include phenomena at the cellular or sub-cellular level, although the Archimedes Modeling Framework enables extension of the Model to deeper levels to address specific questions.

Trajectories of physiological variables

While some variables are fixed (e.g. sex, race/ethnicity), most variables are continuously valued, continuously changing, and continuously interacting. For example, as in reality each simulated person in the Model at any time has a systolic blood pressure (SBP) which is continuously increasing or decreasing depending on various factors and treatments. The SBP in turn affects the development of atherosclerotic plaque, which can cause an MI. An MI can affect cardiac output, and so forth. Thus the value of any particular variable is a function of time, as well as of the values of other variables. We use the term "trajectory" to describe how the value of a variable changes over time and as a function of other variables.

Tests and symptoms

The physiological variables determine test results, symptoms, and health outcomes. When a test is done (e.g., a FPG test), it reads the value of the pertinent variable and reports it back, possibly modified by any random or systematic biases in the test or its interpretation. In this context, the concept of a test is very general and includes not only laboratory tests but any other activity that collects information about a patient, such as a provider taking a history or doing a physical exam, or a patient filling out a survey. When the values of variables reach certain levels they can cause symptoms and health outcomes. For example, when the value of the variable that represents the occlusion of a coronary artery approaches 75%, the person might experience angina.

Diseases

© 2011-2012

Diseases are defined in terms of the underlying physiological variables. For example, a person is said to have "diabetes" when their FPG > 125 mg/dL. This reflects the fact that in reality diseases are not physiological "states" that people are "in," but are actually labels that are applied when physiological variables meet certain criteria. By defining diseases in terms of the underlying variables the Model is able to address several issues: concepts of abnormality change; many diseases are "man made" based solely on the results of tests (e.g. "mild hypertension," "pre-diabetes"); many diseases have multiple – often competing – definitions (e.g. "metabolic syndrome"); definitions can change over time (e.g. prior to 1998 the diagnosis of diabetes was based on FPG > 140, not FPG > 125); definitions can be applied differently in different settings' and "diseases" can overlap (e.g., "coronary artery disease," "diabetes," "cardio-metabolic syndrome"). This approach also enables the Model to accommodate evidence collected at times when different definitions were used.

Treatments

Treatments can modify either the value of a variable (e.g. performing bypass surgery can open an occluded coronary artery), or the rate of change of a variable (e.g. lowering a person's low-density lipoprotein (LDL) cholesterol will slow the rate of progression of the variable that represents plaque in a coronary artery), or both. If a treatment is known to affect a symptom or health outcome, but the mechanism of action of that effect is not known, the effect can be specified directly. For example, the risks or side effects of a treatment can be modeled either by specifying the treatment's effect on another feature if that is known, or by specifying an effect directly on the outcome.

Disease progression variables and functions

In addition to variables that correspond to real physiological phenomena, the Model includes variables that represent physiological constructs for which there are no directly measurable counterparts in reality. An example is the variable that represents insulin resistance. Clinicians and researchers talk about "insulin resistance" as a "cause" of diabetes, even though "resistance" is an abstract construct that cannot be directly observed or measured. In the same way, the Model includes a closely related variable called "insulin efficiency" (the inverse of insulin resistance), which in the Model is the main cause of diabetes in the simulated people. Another example is "plaque," the main variable that causes MIs. We call these "disease progression variables," and the equations that determine their progression over time are called "disease progression functions" [2]. Every disease includes at least one disease progression function, usually named to represent the clinical condition of which it is the cause (e.g. "retinopathy progression variable").

Like other variables in the Model, disease progression functions are defined at a clinical level of detail. The "plaque progression variable" is a good example; the Base Model does not try to include the development of fatty deposits, introduction of foam cells, or the clotting cascade. The Model can be extended to include these if needed for particular projects (provided there are sufficient data), but they

---

[2] In previous publications we used the term "features" as a synonym for "variables." For example, see Eddy DM, Schlessinger L. Archimedes: a Trial Validated Model of Diabetes, Diabetes Care 2003, 26:3093-3101.

© 2011-2012

are not included in the Base Model or ARCHeS Simulator 2.3. Because the Model is written at a clinical level of detail, the equations for the disease progression functions frequently take the form of proportional hazard models.

Equations

The Physiological model contains algebraic and differential equations that describe how each variable relates to other variables and how all the variables together change over time (as the patient ages). Thus the equations are not static like a typical risk calculator which takes a person's current values of a small number of variables and calculates a ten-year risk of some event such as a heart attack. Rather, in the equations in the physiology model, virtually all of the variables are continuous functions of time and other variables. This is important for several reasons. Most importantly, it enables the Model to calculate the effects of changes in risk factors over time. This enables the Model to capture past medical history and past values of risk factors and other variables that affect the occurrence and progression of diseases. It also enables the Model to capture the effects of interventions and other events that may occur in the future.

The equations are based on observed data as recorded in physiological research, surveys, epidemiological studies, registries, and clinical trials. The variables and their relationships for each disease, as well as the sources used to derive the equations for the trajectories and relationships, are described in the section on disease models below.

Data sources

The equations are estimated from a variety of sources, which are listed in the sections that describe the variables and their relationships for each disease. The equations for progression functions are estimated from data on age-specific incidence rates of the clinical events the progression functions are causing. For example, the equation for the type 2 diabetes progression function (corresponding clinically to the "cause" of insulin resistance) can be estimated from data on age-specific incidence rates of type 2 diabetes. This can be done for different populations defined by race/ethnicity or other variables, when there are incidence-rate data for those populations.

Random factors

Many of the physiology equations include random variables to replicate individual variations in such things as the occurrence and progression of diseases, and the development of symptoms. Random variables are also used in the creation of simulated populations, in care processes, and in patient and physician behaviors, as described below.

© 2011-2012

Single integrated model

Because we want the Model to be able to compare a variety of interventions that affect different diseases (e.g. for comparative-effectiveness research or priority setting), the Model includes all the variables, outcomes, and interventions in a single integrated model. Use of a single integrated model also enables the Model to address co-morbidities, syndromes that affect multiple organ systems, use of multiple drugs, and drugs that have multiple effects.

User control

Users of ARCHeS cannot modify the physiology model through the ARCHeS interface. If a modification or extension of the Model is desired, contact us at www.archimedesmodel.com/contact.

Population Model

The second main component of the Model is the population model, which creates simulated people according to specifications provided by the users of ARCHeS.

Project population

Two types of populations can be specified. The first is the "project population" in which the analysis will be done and for which results can be reported. For example, if the purpose of a project is to evaluate several different interventions to reduce cardiovascular risk in adults between age 40 and 85 in the United States, then the project population would be defined as "40 < age < 85." The project population can be defined by a wide variety of variables (e.g., demographics, biomarkers, behaviors, past medical history, and current medications). The ARCHeS interface includes tools that enable creation of simulated populations whose baseline characteristics match those of any population for which a user of ARCHeS has baseline data, such as populations defined by geographic regions, employers, health plan membership, and insurance coverage.

Target population

The second type of population is the "target population" for a particular treatment, with the requirement that the target population is within (a subset of) the project population. There can be as many target populations as there are treatments. For example, if one intervention is to give antihypertensive medications to everyone with SPB > 140 mg/dL, then the target population for that intervention is defined by "SBP > 140." Because the target population is always a subset of the project population, the target population in this example is "((40 < age < 85) and (SBP > 140))." This ability to define both project and target populations is particularly important for projects that include or compare multiple interventions that have different target populations. It also facilitates the identification of populations that are most appropriate for particular treatments.

Creation of a simulated population

In the base version of Archimedes, simulated populations are created using person-specific data from the NHANES IV survey of the US population. The methods are best described by walking through the process for creating a project population. The process begins by the user specifying inclusion and exclusion criteria for the project population, as is done for clinical trials and other studies. The

© 2011-2012

specifications can include multiple variables, combinations of criteria, and nested criteria. (The ARCHeS interface includes a tool for using pull-down lists of variables and logical tests, and parentheses for specifying nested criteria.) The population model then searches the NHANES database to identify people who meet those criteria.

For each of those real persons, the Model then creates a simulated person who, when he or she is aged from his or her birth (age = 0) to the age of the real person at the time of the survey, will match the real person with respect to all the variables pertinent to cardio-metabolic risk. This is done by calculating parameters for the equations that define the trajectories of the variables; specifically, setting the parameters so that the trajectories of all of the variables in the simulated person match the values of the variables observed in the real person[3]. The result of this process is that for every real person, there is a simulated person whose variables very closely match the variables of the real person. The match is not exact because of random factors in the physiology model that affect the values of the simulated person's variables while the person is "aging" (i.e., while the person's physiology is being calculated starting from age = 0).

This method of aging the simulated person from birth is important because it captures a person's past medical history and the effects on variables that are important determinants of future events but that cannot be measured and therefore do not appear in surveys or other data sources. An example is the plaque/MI progression function (representing the plaque in the person's coronary arteries), which is not measured directly but is crucial for calculating the probability that an individual will have a coronary artery event in the future.

The results of this part of the process are illustrated in Table 1, which shows the characteristics of the simulated population and compares them to the characteristics of the real NHANES population, in this case for the age group 20 – 85. Table 1 shows the marginal values of the demographic and biomarker variables. The method also recreates all the correlations between variables.

---

[3] The concept can be illustrated with a simple non-medical example. Suppose the variable we are interested in is the distance a person has travelled from some start point. Suppose that after half an hour (i.e., the "age" of the person after the start of the travel = 30 minutes) the person has traveled 30 miles. The equation for the trajectory of distance as a function of time is "Distance = Rate x Time." Given the available information, we can calculate the value of "rate" for this real person; it is 60 mph. We can then create a simulated person and assign them a rate of 60 mph, and know that after a half an hour the simulated person will also have traveled 30 miles. By calculating the parameter "rate" based on information about the real person, and assigning that rate to a simulated person, we have created a simulated person whose distance matches that of the real person. In the Model, this is done for all variables for all people.

© 2011-2012

Table 1. Comparison of Characteristics of Simulated Population and NHANES Population Age 20 – 85.

Variable	Simulation	NHANES
% Male	0.48	0.48
% Black	0.11	0.11
% Hispanic	0.05	0.05
% Mexican	0.08	0.08
% White	0.71	0.72
Age	46.37	45.44
Albumin	32.12	34.76
BMI	28.23	28.25
Cholesterol	199.65	201.62
Creatinine	0.90	0.90
DBP	71.63	71.68
FPG	101.00	103.73
HDL	54.04	52.68
Hemoglobin	5.48	5.47
LDL	117.97	119.19
SBP	121.72	122.47
% Smoke	0.24	0.26
Triglycerides	139.19	150.73

After the simulated population has been created, the population model then displays the population-level data (i.e., the first two columns of Table 1) in the ARCHeS interface. The user of ARCHeS can then review the information, and either accept the values or modify any or all of them. If the user is conducting an analysis for the US population, he or she would not need to modify the population created from the NHANES dataset. However, the user might be interested in a particular geographical area where smoking rates are higher than the US average, or the user might have baseline characteristics from a real trial that they want to replicate. If the user chooses to change any of the population level values, the population model will resample the simulated people to find a subset that

© 2011-2012

has the desired levels of the variables, still subject to the inclusion and exclusion criteria the user originally specified. The population model contains automated methods that select virtual people in a way that causes the selected population to converge on any specified targets for biomarkers and other variables, retaining the correlations between variables, as closely as possible given the size of the NHANES database.

The target population is defined in a similar way, except that users can only specify the inclusion and exclusion criteria. The population model will then apply those criteria to the project population[4]. An example is the ALLHAT hypertension trial, which had the following inclusion and exclusion criteria.

Inclusion criteria

- Age 55 years or older
- Known hypertensive with BP ≤ 160/100 mmHg on treatment, or BP ≥ 140/90 mmHg and ≤ 180/110 without treatment
- At least one of the following:
    - Left ventricular hypertrophy on electrocardiogram or echocardiogram
    - Known atherosclerotic cardiovascular disease (CVD)
    - Type 2 diabetes mellitus
    - HDL cholesterol <35 mg/dl
    - Current cigarette smoker

Exclusion criteria

- Recent MI or stroke
- Known congestive heart failure or angina pectoris
- Need for any study drug for reasons other than hypertension
- Need for more than two antihypertensive drugs to control BP
- Serious systemic disease
- Elevated serum creatinine (2 mg/dl or greater)

If those criteria are applied to the US population they create a simulated population which can be compared to the ALLHAT population. Table 2 shows the results.

[4] The user cannot modify the characteristics of the target populations because doing so would alter the characteristics of the project population which had previously been set.

Table 2. Comparison of Baseline Characteristics in ALLHAT and Simulated Populations.

Characteristic	Simulation mean	ALLHAT mean	Simulation std. dev.	ALLHAT std. dev.
Age	66.9	66.9	7.6	7.7
BMI	29.9	29.7	6.2	6.2
DBP	88.8	89.0	10.1	10.0
FPG	125.2	123.5	57.0	58.3
HDL	48.5	46.8	14.5	14.7
SBP	156.0	156.0	13.3	16.0
Total cholesterol	212.1	216.2	43.4	43.0
% Had type 2 diabetes	0.37	0.361		
% Had MI or stroke	0.24	0.232		
% Had HDL-C < 35	0.14	0.116		
% Male	0.54	0.531		
% Smoke	0.22	0.219		

User control

As the above discussion makes clear, unlike the Archimedes physiology model, which users are not able to modify, users have greater control over the project population and target populations used in an analysis. Again, the match is never exact because of random factors. This is analogous to the random factors that cause the baseline characteristics of treatment and control groups in clinical trials to be slightly different.

Healthcare System Model

Care processes

Health and economic outcomes are determined not only by the characteristics of the population (as determined by the population model) and the physiology of the diseases of interest (as determined by the physiology model) but also by the level of care people are receiving. The Model is designed to represent care as it is delivered in the US today, on average. Thus the Model includes care processes that represent current national guidelines. Examples are the Adult Treatment Panel (ATP) III guideline for cholesterol and the American Diabetes Association (ADA) guideline for managing HbA1c in people

with diabetes. A list of guidelines incorporated in the base version of the Model is given in the section on the healthcare system model below.

The guidelines are incorporated in the Model by laying them out in the form of pathways (if they are not already in that form), with logical tests at branch points (e.g., "If SBP > 140 mg/dL, then…"). An example is Figure 3, which shows a pathway for testing a patient seen in the emergency room with chest pain. The pathways are then converted to equations and inserted into the healthcare system model.

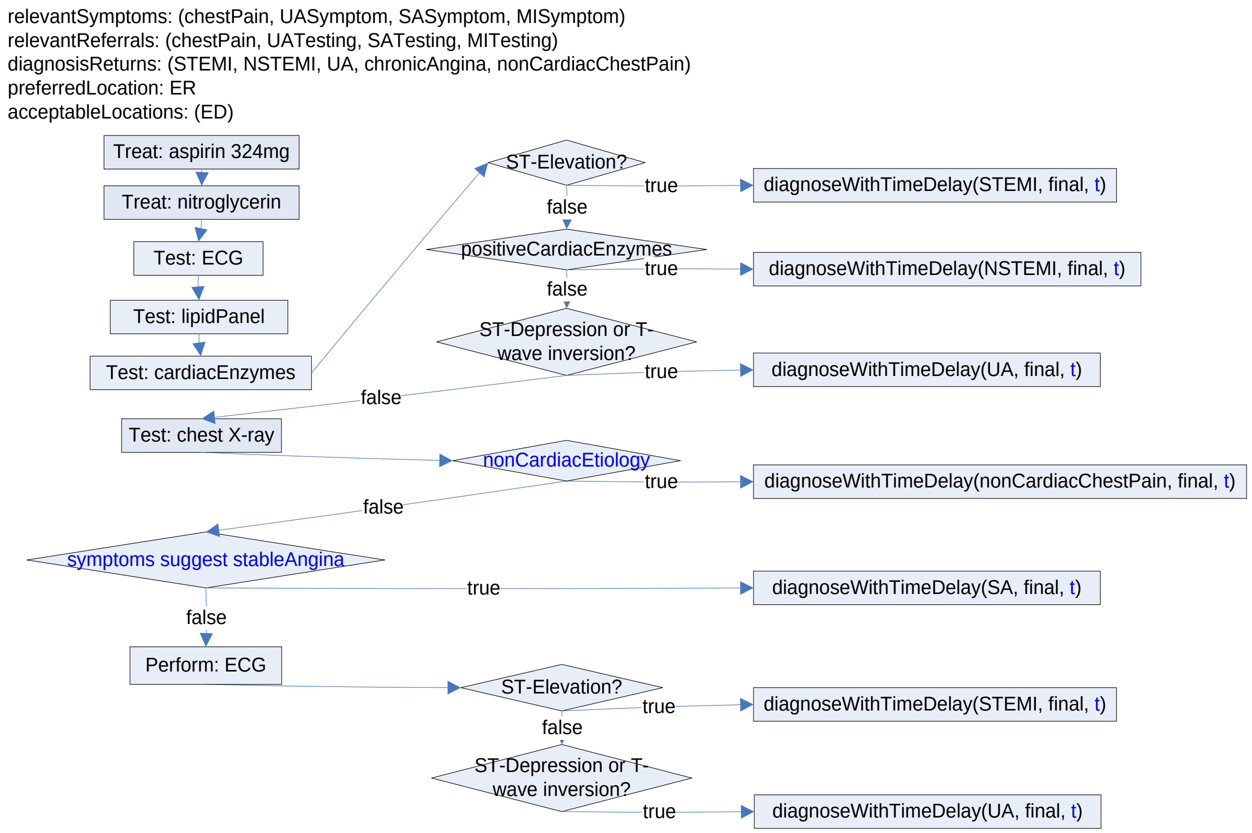

Figure 3. Pathway for Testing a Person Presenting in Emergency Department with Chest Pain.

Ambiguities in a guideline, such as "Test A should be considered" or "Available drugs include…," are examined by in-house physicians and external advisors who have knowledge of the pertinent subject area, supplemented by utilization data if available, to determine how the ambiguities should be addressed in the pathway. If appropriate, probabilities or random variables are inserted at branch points to reflect uncertainties about ambiguous parts of a guideline or variations in practices.

Provider behaviors

Guidelines represent "ideal care" – what the designers of guidelines believe is best supported by the evidence and recommend be followed by all practitioners and patients. However, not all providers follow guidelines, and not all patients adhere to recommended tests and treatments. This is addressed in the Model by assigning probabilities to appropriate branch points, even if the guideline itself is

© 2011-2012

unambiguous, to more realistically represent behaviors relating to performance and adherence. These probabilities can then be calibrated to try to match observed levels performance, compliance, biomarker control, and utilization. Large national datasets, such as the National Ambulatory Care Survey, are used for this purpose. Other sources for calibrating the care processes are listed in the section on the healthcare system model below.

Calibration of care processes

This is done to the greatest extent permitted by the data. However, the different data sources used to calibrate care processes and behaviors often involve different populations, data collection methods (e.g., surveys, claims data, death certificates, hospital admissions), and definitions of events (e.g., different codes for "coronary heart disease" events). The result is that there can be uncertainty about which source is "correct" for a particular variable or application. This is illustrated in Table 3, which shows six different estimates of the prevalence of diabetes in the US.

Table 3. Six Estimates of Prevalence of Type 2 Diabetes in the US.

NHANES	7.61%
NAMCS and NHAMCS-OPD combined (2006)	10.85%
National Diabetes Fact Sheet (2007)	8.10%
AHA Heart and Stroke Update (2009)	7.70%
BRFSS Prevalence and Trends Data (CDC)	8.30%
Heart and Stroke Update (2005)	7.30%

Furthermore, for some variables such as utilization rates of procedures, the observed rates do not match what is called for by a guideline, under any set of assumptions about performance and adherence. Finally, it is well-known that care practices vary widely in different settings. There is no such thing as a single "customary care" or "standard of care" that is applied uniformly throughout the country.

Because of these and other factors, it is not possible to achieve perfect matches for all the variables related to the healthcare delivery system. When there are multiple sources for the same variable, judgments have to be made about which source to use. When it is mathematically impossible to reconcile the sources with each other, or reconcile the observed data with current guidelines, judgments have to be made about which variables are the most important to match. The Model should be thought of as representing "usual" or "customary" care in the same abstract sense as those terms are used in common parlance; it is the "best fit possible" between national guidelines and actual care as reflected in the available data.

Uses of specifying care processes

Despite the limitations of the available data and unavoidable or irreconcilable inconsistencies between national guidelines and actual practices, the explicit incorporation of care processes and behaviors does enable the Model to represent practice patterns more accurately than would otherwise be possible. In

© 2011-2012

particular, it avoids the need to assume that the background care given to patients in a simulation is the same as the background care that was given to people in whatever study that was used to estimate parameters for the Model. (We use the term “background care” to describe all aspects of care other than the interventions being studied in a project.) For example, imagine a state transition model in which the incidence rate of MI (the transition probability from the state “No MI” to the state “MI”) is calculated using the Framingham equation used in the ATP III guideline[5]. This rate reflects the behaviors, screening practices, use of preventive interventions, definitions of co-morbid conditions (e.g. the definition of “diabetes” has changed), and definitions of outcomes in use during the period the Framingham data were collected. Use of that equation to analyze a problem today requires an assumption that care today is the same as the care delivered between 1971 and 1986 in Framingham MA.

Based on the calibrated care protocols just described, the healthcare system model simulates the most important aspects of the delivery system, including visits, admissions, laboratory tests, diagnostic and therapeutic procedures, and treatments.

User control

With ARCHeS, users are able to define care processes for delivering interventions relating to cardio-metabolic risk. Currently it is not possible for users to modify or recalibrate guidelines and care processes that represent background care.

Outcomes Model

The outcomes model is quite simple mathematically, although it involves a large number of calculations. For each person, the outcomes model tracks every event that could affect any of the four main types of outcomes calculated by the Model: utilization, costs, health outcomes, and quality of life. These events are tracked for each person and the times they occur are recorded. Utilization events include visits, admissions, tests, procedures, and treatments. Costs are calculated by multiplying every cost-generating event (e.g., a visit or lab test) by the cost of that event. Quality of life is calculated by recording the time a patient spends with a particular symptom or health outcome, and multiplying by the factor that represents the decrement in quality of life associated with that symptom or health outcome. Outcomes that occur in any given year can be discounted, and present values calculated.

User control

In the base version of the Archimedes Model, costs other than the costs of interventions such as drugs and smoking cessation programs are based primarily on Medicare. ARCHeS enables users to modify the costs assigned to particular interventions such as drugs and smoking cessation programs. It does not enable users to modify the cost of other cost-generating events in the Model, such as the cost of an emergency room visit for chest pain or the cost of caring for a patient with end-stage renal disease. The

[5] Wilson PWF, D'Agostino RB, Levy D, Belanger AM, Silbershatz H, Kannel WB. Prediction of coronary heart disease using risk factor categories, Circulation 1998; 97:1837-47.

© 2011-2012

ability to change the costs of particular aspects of background care will be available in a future release of ARCHeS. However, ARCHeS does enable users to set a parameter that will modify the overall cost of background care. For example, if a health plan knows that on average its costs are 10% higher than those specified by Medicare, it can set this parameter to 1.1 and the costs of all aspects of background care will be increased by 10%. ARCHeS also enables users to modify discount rates for health and/or economic outcomes. Finally, ARCHeS enables users to modify the quality-of-life weights assigned to various health states, and performance/adherence rates for various interventions.

Executing the Model

Set up the simulation

Using the ARCHeS interface, users define the project population, the interventions, and the target populations for each of the interventions. They define the trial arms they want to compare, specifying the particular interventions that will be given in each trial arm (combinations of interventions are allowed), and specifying the proportion of people in each target population who will actually receive each intervention. These steps are equivalent to designing the population and treatment arms of a clinical trial, or the interventions to be applied in a demonstration program. Users then submit the specifications for calculation.

Run the simulation

When the specifications are received by the Simulator, the calculations begin. At the start of the simulation, the physiology of each person in the project population functions according to the person's baseline characteristics and other variables, and the equations in the physiology model. Each person in the simulation is different, with different baseline characteristics, trajectories for physiological variables, behaviors, random factors, and so forth. If a physiological variable for a particular simulated individual reaches a value at which symptoms occur, the person seeks care, either an outpatient visit or an emergency room visit depending on the symptom and other factors. Simulated people can also make contact with the healthcare system if the project interventions call for that. For example, a project intervention might be to screen people for a disease, in which case a simulated provider in the healthcare system will contact the simulated person and offer the screening. When a simulated person makes contact with the healthcare system, either at the initiative of the healthcare system or the initiative of the person, then simulated providers will follow care processes that are based on guidelines, subject to variations in provider behaviors as described above. The person gets tests and treatments according to the care processes and their own behaviors relating to following recommendations for tests and treatments. Any treatments the person receives can change one or more physiological variables, which in turn can change the progression of the disease, and the occurrence of symptoms and health outcomes. Any interventions specified by the user during the setup of the project will also be applied, either in conjunction with or instead of the background care processes, as defined by the user. During the entire process, all events relating to utilization, costs, health outcomes, and quality of life are recorded. After the simulation is complete and all the results have been recorded, a data file is created and sent back to the user through the ARCHeS interface.

© 2011-2012

Analyze the results

The user can then use the part of ARCHeS called the "Outcomes Analyzer" to create a wide variety of tables and charts. The user can also change a variety of assumptions, and ARCHeS will immediately recalculate the tables and charts. The number of functions that can be performed with the Archimedes Outcomes Analyzer is too large to describe here. Access to a demonstration copy of ARCHeS can be obtained by contacting us at www.archimedesmodel.com/contact.

Validating the Model

The Model is validated for face validity, internal validity, cross-validity, external validity, and predictive validity, using best practices defined by a national task force[6]. Particularly important are the external validations that compare the results of the Model with the results observed in clinical trials, cohort follow-up studies, registries, and large national databases. Historically more than 50 major clinical trials have been used to validate the Model at various stages in its development. Furthermore, particular parts of the Model are continually being validated as part of the model-building process. With ARCHeS, a major part of the validation process has been automated; before the release of each new version of the Model, the Model is validated against a standardized suite of studies using an automated "one-click" process. The methods and results are described in the report "Validation Methodology and Results: ARCHeS Simulator 2.3," available at www.archimedesmodel.com/tech-reports.

Physiology Model Variables

As described above, the core of the Archimedes Model is a collection of algebraic and differential equations that represent the physiological variables and relationships pertinent to diseases and their complications – the physiology model. This section introduces non-technical descriptions the variables and relationships in the equations used in each of the disease sub-models and the sources used to derive the equations. Parties with a serious interest in working with Archimedes can contact us at www.archimedesmodel.com/contact for additional, quantitative information about the Model.

Identification and Selection of Data Sources

To identify sources for building the disease models we search PubMed and Google Scholar to identify appropriate articles that meet pre-determined criteria based on:

- study design
- number of participants
- patient characteristics (for example, men and women over age 65 with a diagnosis of CVD)
- study duration

[6] Eddy DM, Hollingworth W, Caro JJ, Tsevat J, McDonald KM, Wong JB. Model Transparency and Validation: A Report of the ISPOR-SMDM Modeling Good Research Practices Task Force Working Group - Part 4, Value in Health 2011, in press.

- outcomes of interest (for example, MI, angina, or stroke)

Individual PubMed MeSH terms are combined for the search. After the search is performed, each abstract found is reviewed by in-house physicians and analysts to determine if the study meets the inclusion criteria. Studies that meet the criteria are read and evaluated for use in building the Model. References from studies retrieved from the search are also reviewed when appropriate to identify any papers that might not be identified in the initial search. The following aspects of the search are documented for future use:

- search date
- databases searched
- terms used
- number of papers originally found
- number of papers that met the inclusion criteria

Disease Diagrams

The variables and relationships between variables that are used to model the diseases in Simulator 2.3 are illustrated in what we will call "disease diagrams." In the diagrams the rectangles represent variables that in clinical parlance are often called "risk factors" for the disease. Ovals represent variables that are directly related to the disease and its progression. Rectangles with rounded corners represent outcomes of the disease that are experienced by patients such as symptoms and health outcomes. When variables related to the disease affect other organs and other diseases, they are represented in squares. Variables that can be modified with treatments or other inventions are indicated by "Rxs." Variables that can be measured by tests are indicated by "Tests." The arrows represent relationships between the elements, with the direction of the arrow representing the direction of causality. In general, arrows represent equations that relate the variables. None of the elements in these diagrams represent "states" as found in state-transition models, and the arrows do not represent transition probabilities.

Relationships between variables are continuous and dynamic

When viewing these diagrams it is important to bear in mind that virtually all of the variables are continuously valued and changing over time. (The main exceptions are sex and race/ethnicity.) For example, in Figure 4 below, which describes the metabolic changes in type 2 diabetes, the progression toward diabetes (the type 2 diabetes progression function) is a continuous function of a person's weight along with other variables. That is, if a person's weight changes, perhaps due to a diet or exercise program, then the trajectory of the progression function will change to reflect the change in weight. This is very different than, for example, a risk equation such as the Framingham risk equation for MI, which is static in the sense that it takes a person's current values of variables such as age, sex, and SBP, and calculates a ten-year risk of a heart attack from that point. That type of equation does not incorporate information about changes in risk factors in an individual's past or future. In the Archimedes Model variables such as age, SBP, and cholesterol levels are continuously changing, and the progression of the variables that represents plaque and the occurrence of MI are continuously changing with them.

© 2011-2012

Diabetes Metabolism Model

The variables relating to glucose metabolism and the development of type 2 diabetes are shown in Figure 4. The propensity to develop type 2 diabetes is represented by the type 2 diabetes progression variable. Its associated function, the type 2 diabetes progression function, determines how the propensity to develop the disease progresses as a function of time and other variables. The progression function is different for people of different sexes and races or ethnicities, and depends as well on a person's age, weight (BMI), and family history. The progression function affects the variable in the model that represents the efficiency with which insulin affects plasma glucose levels. It corresponds clinically to how insulin affects the uptake of glucose by fat and muscle. Insulin efficiency is the inverse of insulin resistance. FPG levels are determined by insulin efficiency and by basal hepatic glucose production. The latter is estimated from data on people who do not have diabetes and is a function of age. HbA1c is a function of plasma glucose levels. The chance a person with diabetes will develop a macrovascular or microvascular complication is determined by HbA1c (which affects coronary artery disease (CAD), retinopathy, and neuropathy), by FPG levels (which affects albuminuria and nephropathy), and by the progression function itself (which affects strokes and nephropathy). The main sources used for this part of the Model are NHANES II (for the progression function), Diabetes in America second edition (for FPG levels), and UKPDS33 (for insulin efficiency). Additional sources are listed in the remainder of this section.

© 2011-2012

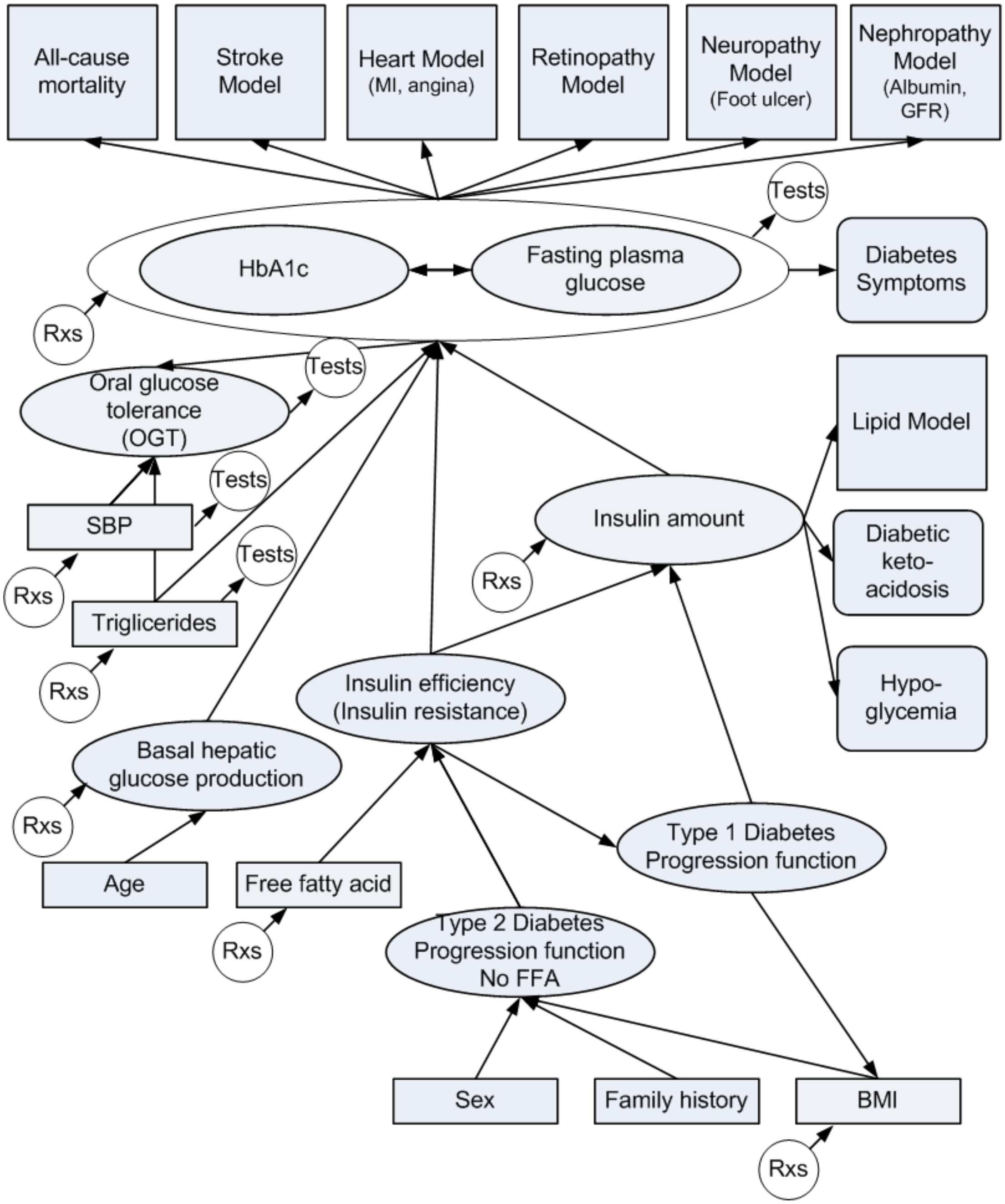

Figure 4. Diagram of Diabetes Metabolism and Pathology.

Type 2 Diabetes Progression Function

The type 2 diabetes progression function determines the probability that an individual will develop type 2 diabetes and the age at which he or she will develop it. Clinically, it corresponds to the "cause" of insulin resistance. The type 2 diabetes progression function is estimated from data on the prevalence of type 2 diabetes at various ages. In Simulator 2.3, diabetes progression functions are calculated separately for men and women and for each of three ethnic groups: white, black, and Hispanic/Mexican

ARCHIMEDES
© 2011-2012

American, all using prevalence data from NHANES III. The progression functions for these three groups are also affected by three other risk factors: BMI, age, and family history. These risk factors are assumed to be independent of sex and race/ethnicity and are incorporated in the equation as relative risks.

The effect of BMI on a person's risk of developing diabetes is based on the following epidemiological studies, which focused on men, women, and extremely obese persons, respectively.

- Field AE et al. Impact of overweight on the risk of developing common chronic diseases during a 10-year period, Arch Intern Med 2001; 161:1581-1586.
- Colditz GA et al Weight gain as a risk factor for clinical diabetes mellitus in women, Ann Intern Med 1995; 122(7):481-486.
- Sjöström L et al. The Swedish Obese Subjects Study Scientific Group, Lifestyle, diabetes, and cardiovascular risk factors 10 years after bariatric surgery, N Engl J Med 2004; 351:2683-93.

The effect of family history on a person's risk of developing diabetes is based on:

- Grill V et al. Family history of diabetes in middle-aged Swedish men is a gender unrelated factor which associates with insulinopenia in newly diagnosed diabetic subjects, Diabetologia 1999; 42:15-23.
- Meigs JB et al. Parental transmission of type 2 diabetes, The Framingham Offspring Study, Diabetes 2000; 49:2201-2207.
- Millar WJ and Young TK. Tracking diabetes: Prevalence, incidence and risk factors, Health Rep 2003; 14:35-47.
- Harrison TA et al. Family history of diabetes as a potential public health tool, Am J of Prev Med 2003; 24:152-159.

Fasting Plasma Glucose, Insulin Efficiency, and HbA1c

The incidence and progression of type 2 diabetes is characterized by elevated and gradually increasing levels of plasma glucose and FPG, caused by increasing degrees of insulin resistance. The Archimedes Model includes a variable "insulin efficiency" that corresponds to insulin resistance; it is the inverse of insulin resistance. (As resistance to insulin goes up, the efficiency with which insulin controls glucose levels goes down and vice versa). In the Model, insulin efficiency is determined by the type 2 diabetes progression function. As diabetes progresses, insulin efficiency declines. The main source for estimating the relationship between insulin deficiency and FPG is the UKPDS trial. A random component has been added to represent variability among individuals.

In people who do not have diabetes, basal hepatic glucose production and the relationship between FPG and age are based on data from

- Diabetes In America, 2nd edition. National Diabetes Data Group, National Institutes of Health, National Institute of Diabetes and Digestive and Kidney Diseases, NIH Publication No. 95–1468, 1995. (Available at http://diabetes.niddk.nih.gov/dm/pubs/america/index.htm.)

HbA1c is determined as a function of FPG based on data from simultaneous measurements of FPG and HbA1c in NHANES III.

Effects on Other Disease Models

As illustrated in Figure 4, the progression of diabetes affects many organs in the body. These effects are modeled based on the current understanding of the mechanisms by which the disease affects those organs. Diabetic neuropathy and retinopathy are driven by HbA1c levels. Cardiac complications of diabetes are directly affected both HbA1c and insulin efficiency. The type 2 diabetes progression function directly affects angina, kidney function, strokes, and overall death rates.

The effect of diabetes on cardiovascular disease is based on many references, the most important of which are:

- The Action to Control Cardiovascular Risk in Diabetes Study Group. Effects of intensive glucose lowering in type 2 diabetes, N Engl J Med 2008; 358:2545-59.
- The ADVANCE Collaborative Group. Intensive blood glucose control and vascular outcomes in patients with type 2 diabetes, N Engl J Med 2008; 358:2560-72.
- Maka KH and Haffnerb SM. Diabetes abolishes the gender gap in coronary heart disease, European Heart Journal 2003; 24(15):1385-1386. (http://eurheartj.oxfordjournals.org/cgi/content/full/24/15/1385).
- Anderson KM et al. Cardiovascular disease risk profiles, American Heart Journal 1991; 121:293-298.
- Coutinho M et al. The relationship between glucose and incident cardiovascular events: A metaregression analysis of published data from 20 studies of 95,783 individuals followed for 12.4 years, Diabetes Care 1999; 22:233–240.
- Howard BV et al. Adverse effects of diabetes on multiple cardiovascular disease risk factors in women. The Strong Heart Study, Diabetes Care 1998; 21(8):1258-65.
- UK Prospective Diabetes Study (UKPDS) Group. Intensive blood-glucose control with sulphonylureas or insulin compared with conventional treatment and risk of complications in patients with type 2 diabetes (UKPDS 33), Lancet 1998; 352(9131):837-53.
- Clarke PM et al. on behalf of the UK Prospective Diabetes Study (UKPDS) Group. A model to estimate the lifetime health outcomes of patients with type 2 diabetes: the United Kingdom Prospective Diabetes Study (UKPDS) outcomes model (UKPDS no. 68), Diabetologia 2004; 47:1747–1759.
- Coleman RL et al. Framingham, SCORE, and DECODE risk equations do not provide reliable cardiovascular risk estimates in type 2 diabetes, Diabetes Care 2007; 30:1292-1293. (http://care.diabetesjournals.org/cgi/content/full/30/5/1292.)

The effects of diabetes on development of kidney disease, stroke, and mortality are discussed below.

© 2011-2012

Diabetic Retinopathy Model

The variables in the diabetic retinopathy model and their relationships are illustrated in Figure 5. In people with diabetes the first manifestation of diabetic retinopathy is non-proliferative retinopathy. The disease can then progress to proliferative retinopathy and blindness. The progression variables that determine whether and when a person with diabetes will develop retinopathy and the rate at which it will progress are determined by the duration of diabetes (defined as time since FPG > 125 mg/dL), SBP, and HbA1c level. (In the Archimedes Model, unless specifically stated otherwise, duration of diabetes is always defined as time since FPG > 125 mg/dL. Notice that this is different from onset of symptoms or first diagnosis.) The rate at which proliferative diabetic retinopathy (PDR) will develop is a function of how long the person has had non-proliferative diabetic retinopathy (NPDR). The occurrence of unilateral or bilateral blindness is determined by the progression of PDR. A patient with NPDR can also develop macular edema, with the progression to that condition affected by how long the patient has had non-proliferative diabetic retinopathy, as well as the patient's SBP and LDL cholesterol.

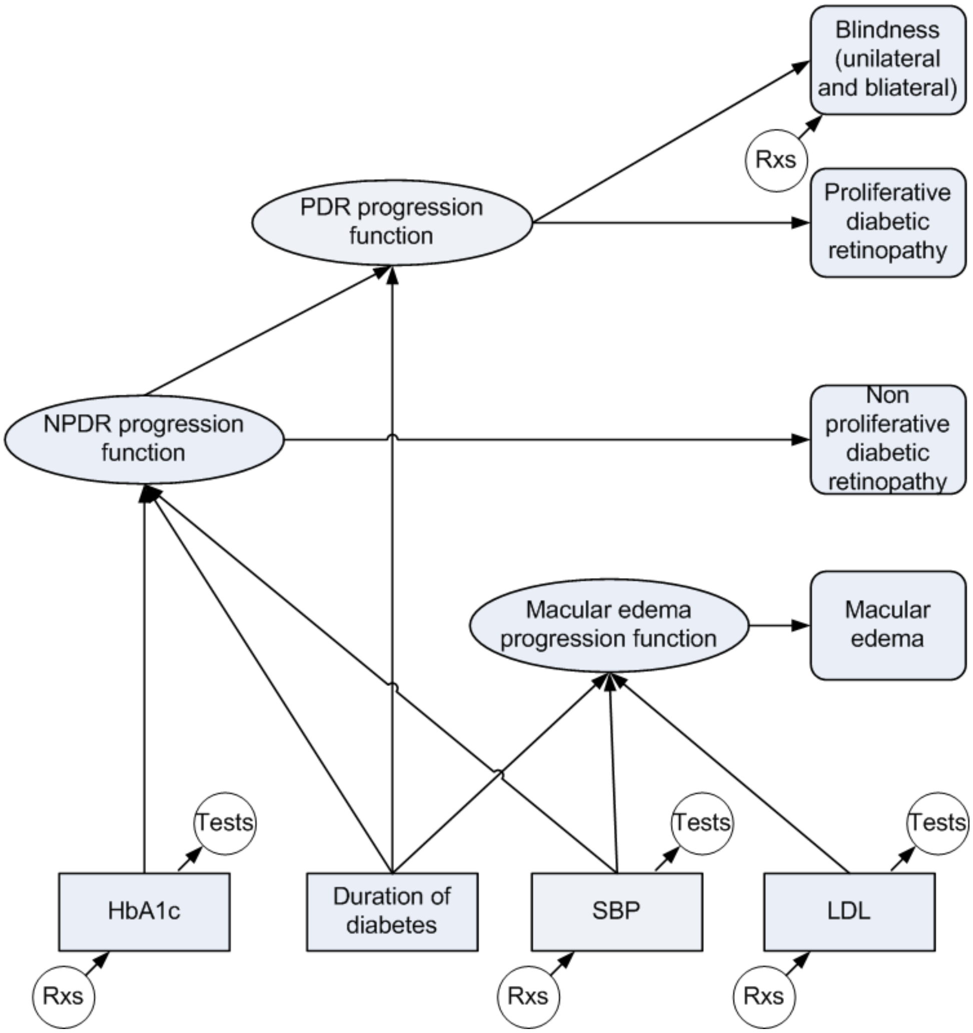

Figure 5. Diagram of Diabetic Retinopathy.

ARCHIMEDES
© 2011-2012

Risk Factors for Non-Proliferative Diabetic Retinopathy

Risk factors in the Model for NPDR are blood glucose levels (represented by HbA1c), SBP, duration of diabetes, and diabetes type (type 1 or type 2). The risk of developing NPDR as a function of HbA1c is based on data from the following five studies:

- Harris M et al. Is the risk of diabetic retinopathy greater in non-Hispanic blacks and Mexican Americans than in non-Hispanic whites with type 2 diabetes?, Diabetes Care 1998; 21(8)1230-1235.
- Haffner SM et al. Diabetic retinopathy in Mexican Americans and non-Hispanic whites, Diabetes 1988; 37(7):878-84.
- Tapp RJ et al. The prevalence of and factors associated with diabetic retinopathy in the Australian population, Diabetes Care 2003; 26(6):1731-7.
- Brown JB et al. Diabetic retinopathy: contemporary prevalence in a well-controlled population, Diabetes Care 2003; 26(9):2637-42.
- Klein R et al. Relationship of hyperglycemia to the long-term incidence and progression of diabetic retinopathy, Arch Intern Med 1994; 154(19):2169-78.

The effect of SBP levels on the risk of developing NPDR is based on data from the first four papers listed above, plus the following two papers:

- Klein R et al, DeMets DL. Is blood pressure a predictor of the incidence or progression of diabetic retinopathy? Arch Intern Med 1989; 149(11):2427-32.
- Adler AI et al. Association of systolic blood pressure with macrovascular and microvascular complications of type 2 diabetes (UKPDS 36): prospective observational study, BMJ 2000; 321(7258):412-9.

The risk of people with type 2 diabetes developing NPDR is based on data from:

- Klein R et al. The Wisconsin epidemiologic study of diabetic retinopathy. III. Prevalence and risk of diabetic retinopathy when age at diagnosis is 30 or more years, Arch Ophthalmol 1984; 102(4):527-32.
- Brown JB et al. Diabetic retinopathy: contemporary prevalence in a well-controlled population, Diabetes Care 2003; 26(9):2637-42.

Risk Factors for Proliferative Diabetic Retinopathy

The Model assumes that no one develops PDR without first developing NPDR. The risk of developing PDR, given that the individual has already developed NPDR, depends only on the duration of diabetes.

The risk of developing PDR for people with type 2 diabetes is based on:

ARCHIMEDES
© 2011-2012

- Klein R et al. The Wisconsin epidemiologic study of diabetic retinopathy. III. Prevalence and risk of diabetic retinopathy when age at diagnosis is 30 or more years, Arch Ophthalmol 1984; 102(4):527-32.
- Brown JB et al. Diabetic retinopathy: contemporary prevalence in a well-controlled population, Diabetes Care 2003; 26(9):2637-42.

Risk Factors for Diabetic Macular Edema

As with PDR, no one will develop macular edema without first developing NPDR. The risk of developing macular edema, given that the individual has already developed NPDR, depends on duration of diabetes, SBP, and LDL cholesterol. The development of macular edema as a function of LDL cholesterol level is based on data from the following paper:

- Rema M et al. Association of serum lipids with diabetic retinopathy in urban South Indians--the Chennai Urban Rural Epidemiology Study (CURES) Eye Study—2, Diabet Med 2006; 23(9):1029-36.

The risk of developing macular edema as a function of SBP level is based on:

- Brown JB et al. Diabetic retinopathy: contemporary prevalence in a well-controlled population, Diabetes Care 2003; 26(9):2637-42.

The relationship between the duration of diabetes and the development of macular edema is based on:

- Klein R et al. The Wisconsin epidemiologic study of diabetic retinopathy. IV. Diabetic macular edema, Ophthalmology 1984; 91(12):1464-74.
- Klein R et al. The Wisconsin Epidemiologic Study of diabetic retinopathy. XIV. Ten-year incidence and progression of diabetic retinopathy, Arch Ophthalmol 1994; 112(9):1217-28.
- Brown JB et al. Diabetic retinopathy: contemporary prevalence in a well-controlled population, Diabetes Care 2003; 26(9):2637-42.

Blindness

The rate at which people who have been diagnosed with PDR lose vision in one or both eyes depends strongly on whether the person has had laser treatment, which is included explicitly in the care processes in the Model. The rates of blindness in patients with type 2 diabetes are based on data from:

- Klein R et al. Visual impairment in diabetes, Ophthalmology 1984; 91(1):1-9.

Diabetic Neuropathy Model

The variables in the diabetic neuropathy model are shown in Figure 6. The model includes three main conditions: sensory neuropathy, foot ulcers, and amputations. Each has its own progression variable and progression function, and each is determined by the patient’s HbA1c. In addition, amputation is affected by a person’s sex as well as the person’s level of proteinuria and the length of time a person has had

diabetes (FPG > 125 mg/dL). The development of foot ulcers is a function of proteinuria and duration of diabetes as well as HbA1c. The development of sensory neuropathy is a function of HbA1c and the person's age and height. The dependence on duration of diabetes reflects the fact that foot ulcers occur more frequently in individuals who have had diabetes longer.

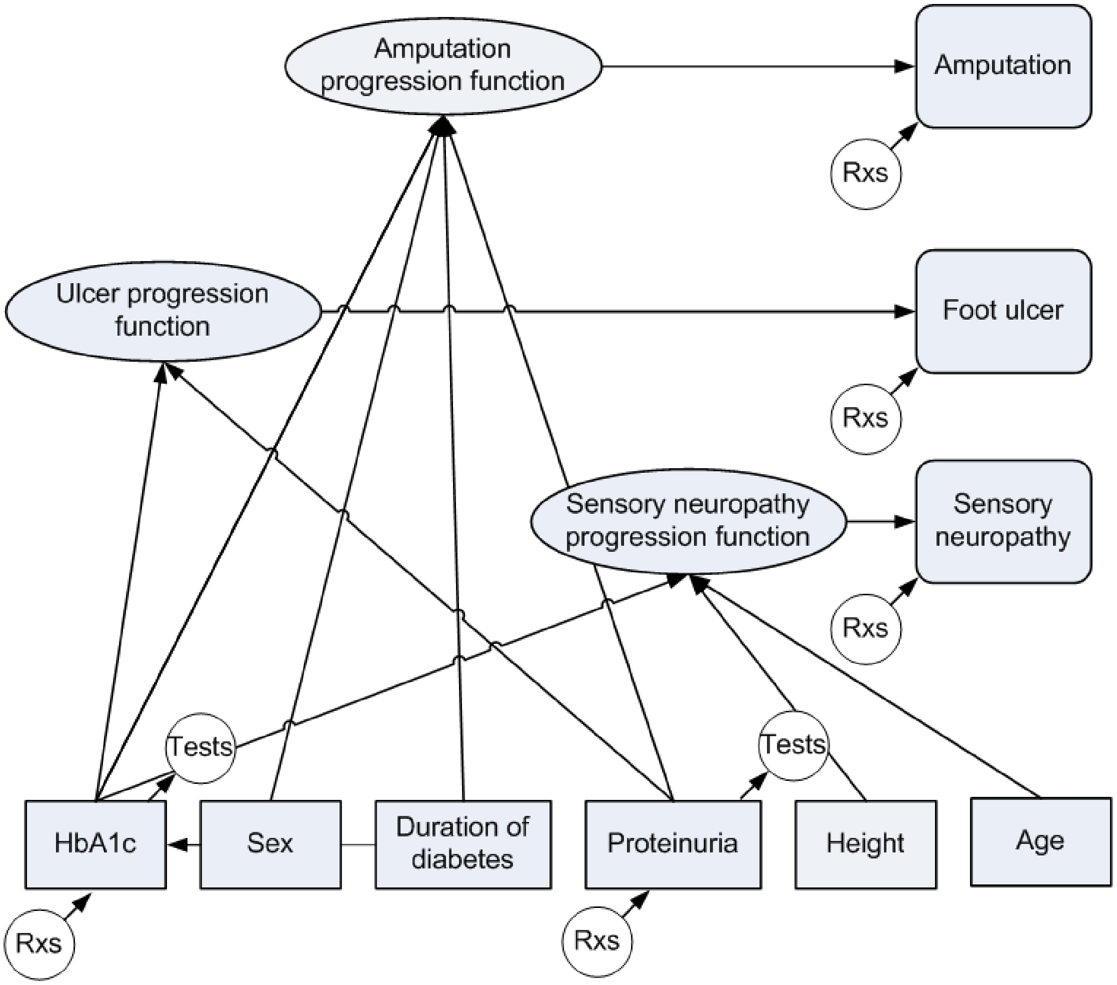

Figure 6. Diagram of Diabetic Neuropathy.

Risk Factors for Sensory Neuropathy

The risk of sensory neuropathy as a function of age, height, and HbA1c is based on:

- Adler AJ et al. Risk factors for diabetic peripheral sensory neuropathy: results of Seattle Prospective Diabetic Foot Study, Diabetes Care 1997; 20(7).

Risk Factors for Foot Ulcer

The risk of developing foot ulcers as a function of HbA1c, duration of diabetes, and proteinuria is based on:

ARCHIMEDES
© 2011-2012

- Moss SE et al. The prevalence and incidence of lower extremity amputation in diabetic population, Arch Intern Med 1992; 152:610-616.

Foot ulcers can recur after treatment. The recurrence rate is based on:

- Apelqvist J et al. Long-term prognosis of diabetic patients with foot ulcers, J Intern Med 1993; 233:485-491.

Risk Factors for Amputation

Risk factors for lower limb amputation are blood glucose levels as measured by HbA1c, duration of diabetes, proteinuria, sex, and history of foot ulcer. A total of eight amputation sites are used in the Model: toe, foot/ankle, below knee, and above knee for each leg. The function for amputation and amputation recurrence rate are based on the sources listed above for foot ulcer.

Nephropathy Model

The nephropathy model is shown in Figure 7. It is based on two progression functions: one for urinary albumin and one for glomerular filtration as measured by the glomerular filtration rate (GFR). The urinary albumin progression function affects GFR. Urinary albumin determines the development of micro- and macro-albuminuria. GFR determines the stage of chronic kidney disease (CKD). In the final stage, CKD stage 5, patients become candidates for dialysis or kidney transplants. GFR depends on sex, age, diabetes status (whether or not a person has diabetes), SBP, urinary albumin, presence of retinopathy, current smoking status, and baseline value of GFR. Urinary albumin depends on age, race/ethnicity, FPG, BMI, SBP, current smoking status, presence of CVD (history of angina, stroke, MI, or revascularization), sex, and baseline value of urinary albumin. The presence of retinopathy affects a person's risk of having a reduced GFR.

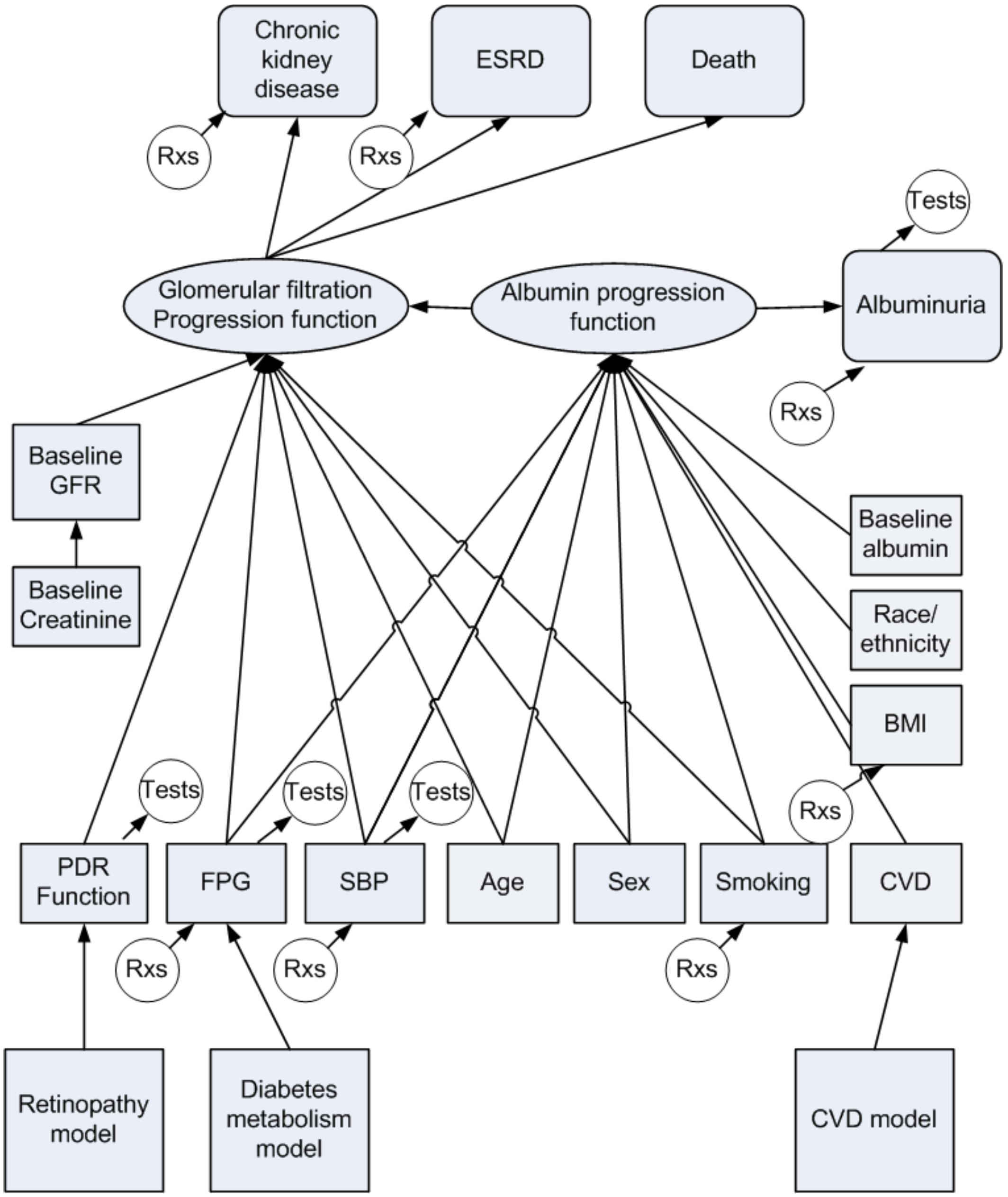

Figure 7. Diagram of Diabetic Nephropathy.

Risk Factors for Urinary Albumin

The dependence on age, race/ethnicity, FPG, BMI, SBP, and current smoking status is based on data from the Framingham Offspring longitudinal dataset, which is described in:

- Feinleib M et al. The Framingham Offspring Study. Design and preliminary data, Prev Med 1975; 4(4):518-25.

The increased risk of albuminuria associated with the presence of CVD and sex is based on:

- Retnakaran R, Cull CA, Thorne KI, Adler AI, Holman RR for the UKPDS study group. Risk factors for renal dysfunction in type 2 diabetes: UK Prospective Diabetes Study 74, Diabetes 2006; 55:1832-1839.

Risk Factors for Glomerular Filtration Rate

The dependence on sex and diabetes status is based on:

- United States Renal Data System. USRDS 2006 Annual Data Report. Bethesda, MD: National Institute of Diabetes and Digestive and Kidney Diseases (NIDDK), National Institutes of Health (NIH), U.S. Department of Health and Human Services (DHHS); 2006. www.usrds.org.

Dependence on SBP is based on data from the Framingham Offspring longitudinal dataset, cited above.

Three of the remaining risk factors (urinary albumin, presence of retinopathy, and current smoking status) are based on

- Retnakaran R, Cull CA, Thorne KI, Adler AI, Holman RR for the UKPDS study group. Risk factors for renal dysfunction in type 2 diabetes: UK Prospective Diabetes Study 74, Diabetes 2006; 55:1832-1839.

Coronary Artery Disease Model

The coronary artery disease (CAD) model is illustrated in Figure 8. It is driven by three progression functions that cause people to get stable angina, unstable angina, and MI. Stable angina corresponds clinically to occlusive plaque which causes symptoms well before complete occlusion and MI. It is measured by tests such as angiograms and treated by procedures such as PTCA and bypass grafts. Unstable angina corresponds to unstable plaque, which causes intermittent angina symptoms and indicates a high risk of MI due to sudden occlusion. Tests such as carotid intima-medial thickness (CIMT) indicate a person's risk of unstable plaque. The MI progression function determines the occurrence of an occlusive event such as rupture of plaque, clotting, and occlusion of the coronary artery. In the Model an MI can occur in any of the coronary arteries, and at any place within any artery. The proportions of MIs occurring at particular spots in particular arteries are set to match observed frequencies. Depending on the artery and the spot in the artery at which the occlusion occurs, the myocardium can be deprived of blood, causing symptoms and reduction in cardiac output. Because stable angina, unstable angina, and MI can all occur independently of each other, and because there is no progression from one to the other, they are represented in the Archimedes Model with their own progression functions. However, their occurrences are highly correlated because they share the same risk factors, and the presence of one implies (but does not cause) an increased risk of occurrence of the others. The correlations between the three types of coronary artery events is captured in the equations that calculate the progression functions for each event as functions of risk factors they share.

Person-specific data from two main sources are used to write the equations for the progression functions. Data from Atherosclerosis Risk in Communities (ARIC) are used to write progression functions for first MI, unstable angina, and unstable angina. Data from the Framingham Heart Study (FHS) are

used to write progression functions for first MI and recurrent MIs. Information about these datasets is in the following references:

- The ARIC Investigators. The Atherosclerosis Risk in Communities (ARIC) Study: design and objectives, Am J Epidemiology 1989; 129(4):687-702.
- McGee D. The Framingham Study: An epidemiologic investigation of cardiovascular disease, Section 27. Bethesda, MD: US Government Printing Office, 1973.
- Anderson KM et al. Cardiovascular disease risk profiles, American Heart Journal 1991; 121(1):293-298.

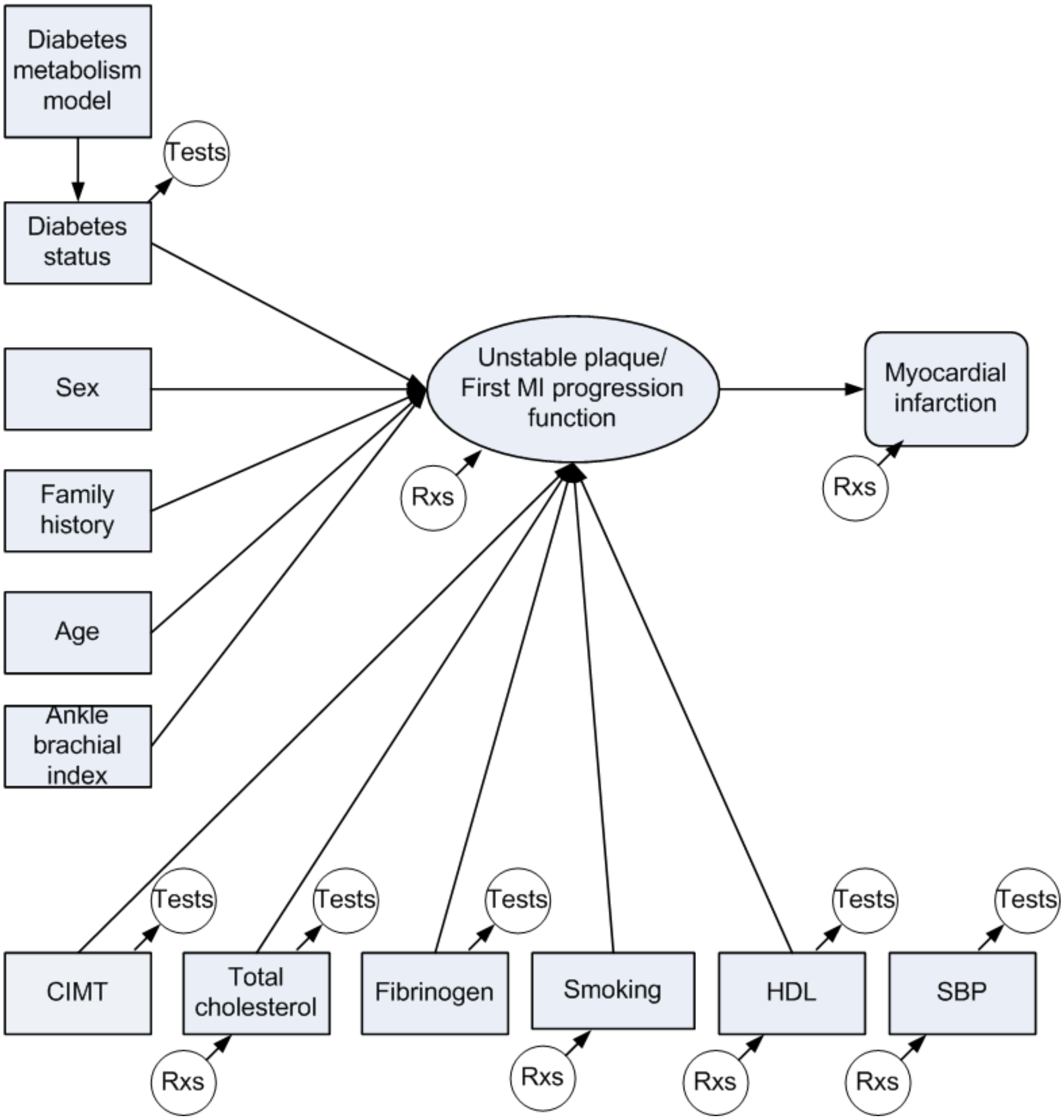

Figure 8. Diagram of First MI.

Risk Factors for First Myocardial Infarction

Risk factors for a person's first MI are age, sex, SBP, smoking status, HbA1c, family history, total cholesterol, HDL cholesterol, fibrinogen, ankle-brachial index (ABI), and CIMT. An additional risk factor is the presence of diabetes; this factor is calculated separately for men and women. Risk factors for first MI are based on the ARIC and Framingham datasets, cited above. Recurrent MI depends on time since the previous MI, diabetic status, and a randomizing factor. Risk factors for recurrent MI are based on the Framingham and ARIC datasets, cited above.

Risk Factors for Unstable Angina

The risk factors for unstable angina are the same as those for first MI and are also based on the ARIC dataset.

Risk Factors for Stable Angina

Risk factors for stable angina are age, sex, smoking status, family history, total cholesterol, HDL cholesterol, presence and severity of diabetes (calculated from the type 1 and type 2 diabetes progression function and FPG), waist-to-hip ratio (WHR), ABI, and CIMT. The progression function for stable angina is estimated using the ARIC dataset.

© 2011-2012

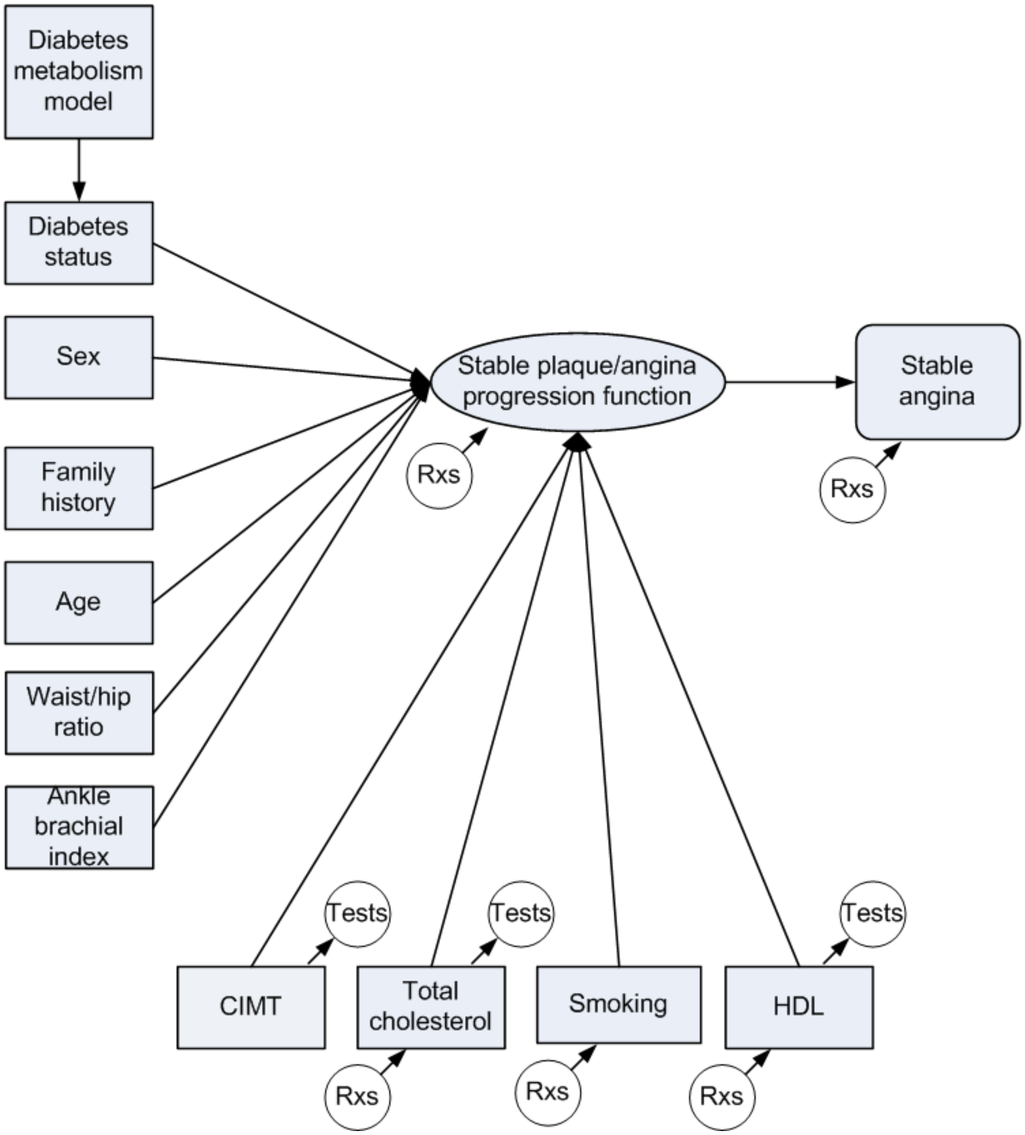

Figure 9. Diagram of Stable Angina.

Circulation Model

The circulation model (Figure 10) calculates a person's SBP and DBP. They are calculated as functions of left ventricular pressure and peripheral resistance. The circulation model illustrated in Figure 10 represents events with myocardial function (i.e., in the absence of any MI). SBP and DBP are calculated as exact solutions to a three-element Windkessel model[7].

[7] Kerner DR. Solving Windkessel Models with MLAB, www.civilized.com/mlabexamples/winkesmodel.htmld.

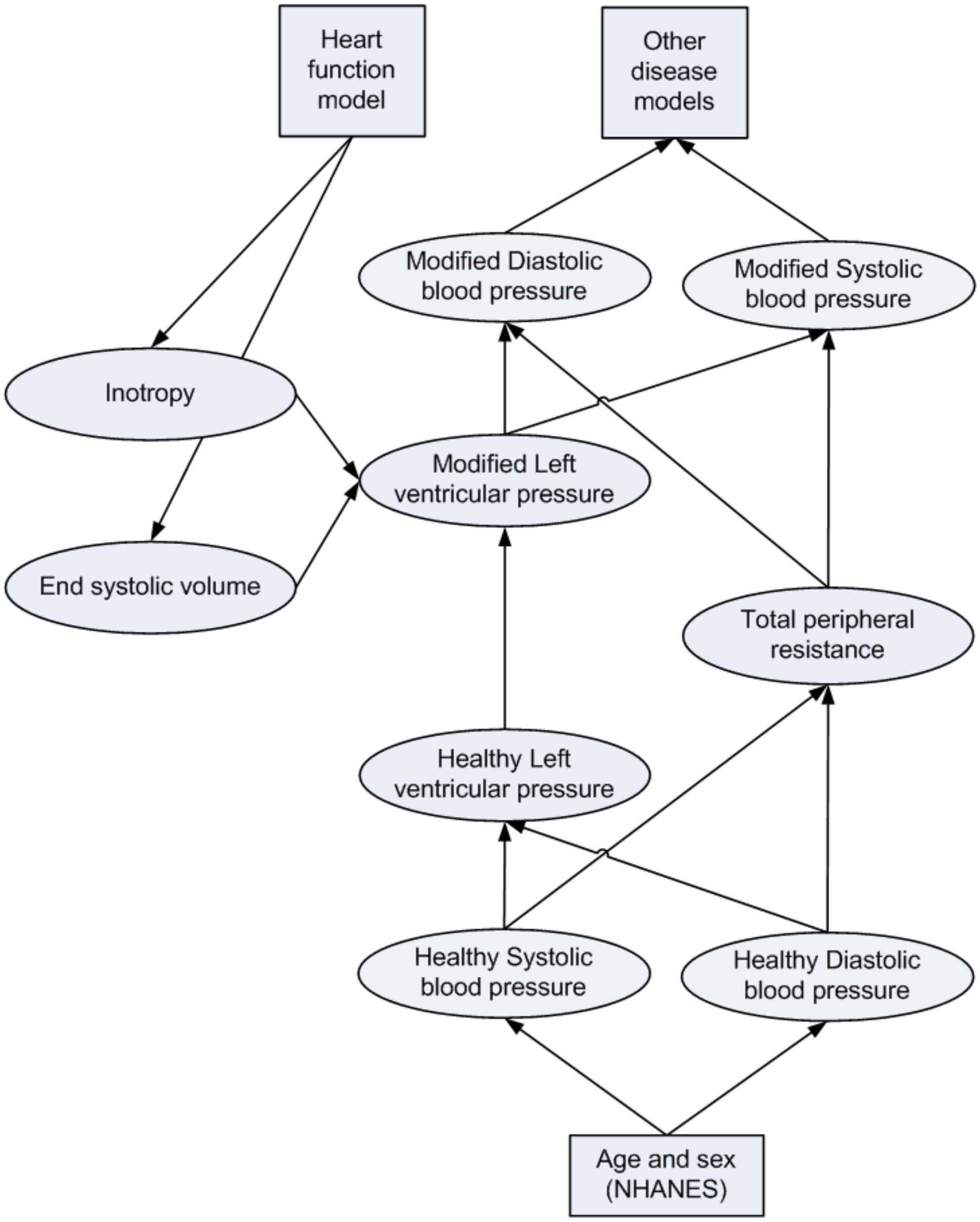

Figure 10. Diagram of Circulation Model.

Myocardial Damage Model

The Archimedes Model includes a representation of the pathological events that occur following an MI. They are illustrated in Figure 11. When an MI occurs due to CAD, there is damage to the myocardium. The amount of damage is determined by which coronary artery was occluded and the spot in the artery of the occlusion. More proximal occlusions (occlusions closer to the origin of the artery) will cause greater damage than more distal occlusions (occlusions closer to the tip of the artery). The damage reduces myocardial contractility which effects end systolic volume, stroke volume, and cardiac output. Reduction in cardiac output in turn affects blood pressures and blood flow to the coronary arteries, in a

ARCHIMEDES
© 2011-2012

feedback loop. Myocardial damage is a function of time, and can be halted or reversed depending on the timing of treatments. This part of the Model is used to study various tests (e.g. electrocardiograms, enzymes), treatments (e.g. thrombolytic agents), and the timing of treatments that are important for the management of acute MI. This part of the Model is in operation in the base version used by the ARCHeS interface. However ARCHeS itself does not include treatments for acute MI, and users of ARCHeS cannot access this part of the Model.

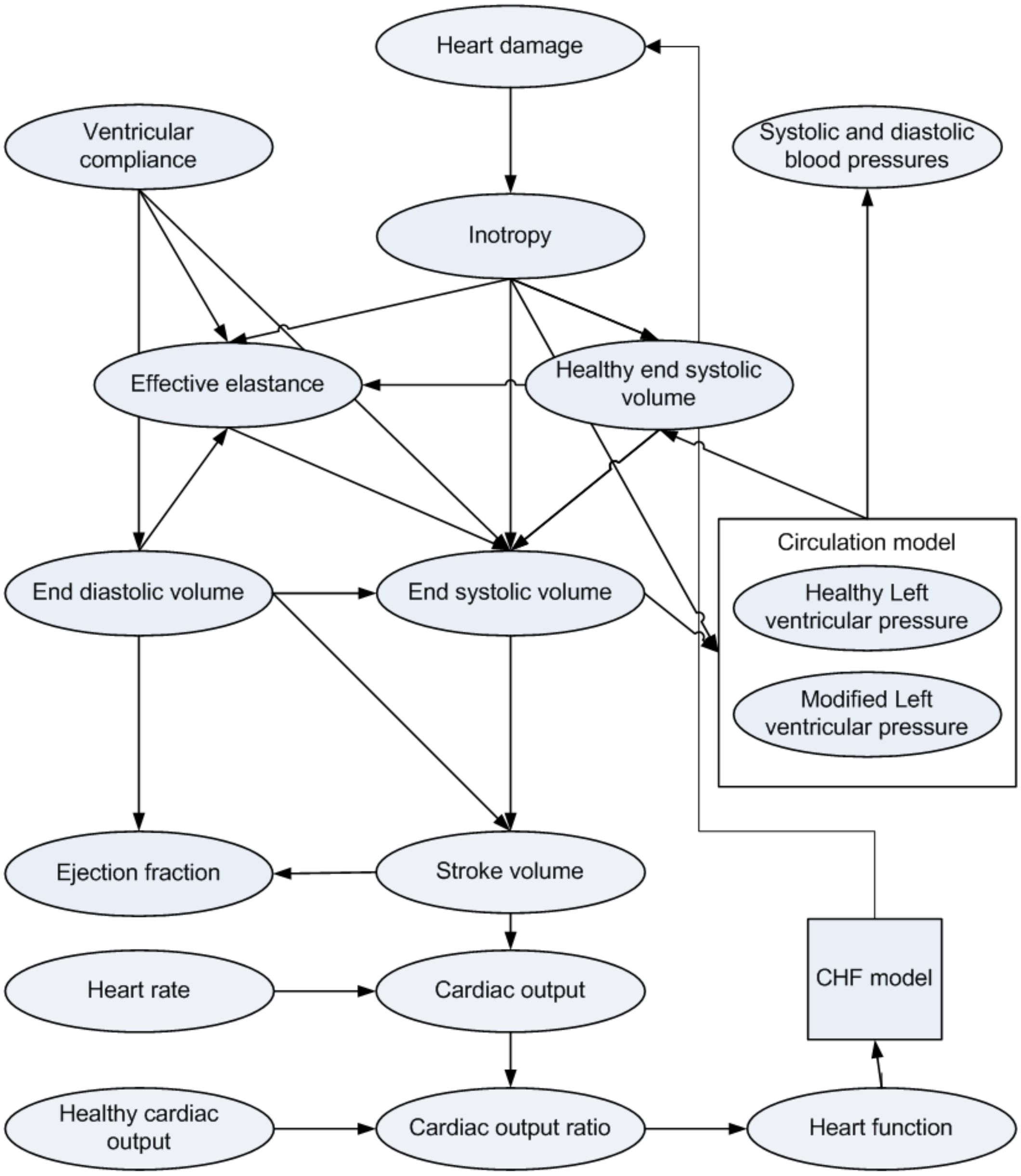

Figure 11. Diagram of Heart Function.

© 2011-2012

Chronic Atrial Fibrillation

Chronic atrial fibrillation is important not only as a condition in its own right, but also because of the risk it imposes for ischemic stroke. Its occurrence is determined by the chronic atrial fibrillation progression function, which is a function of age, sex, body-mass index (BMI), SBP, DBP, history of CAD, and smoking status (Figure 12).

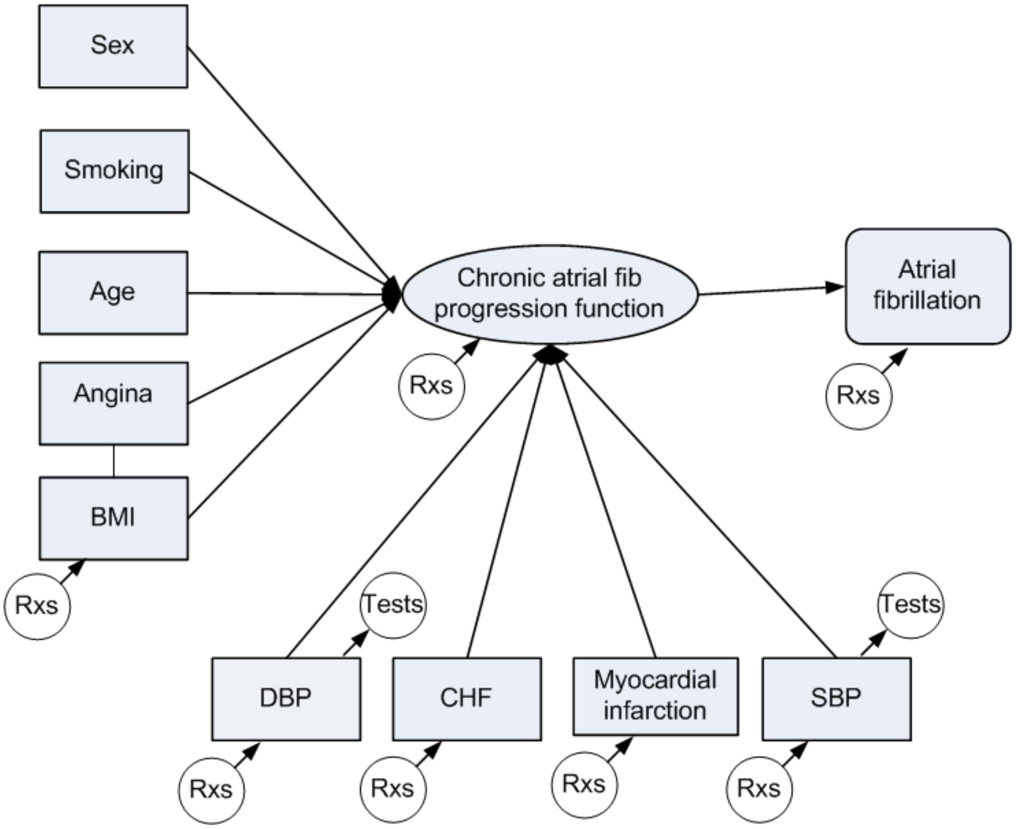

Figure 12. Diagram of Chronic Atrial Fibrillation.

The progression function for chronic atrial fibrillation is based on:

- Ruigomez A et al. Incidence of chronic atrial fibrillation in general practice and its treatment pattern, Journal of Clinical Epidemiology 2002; 55:358-363.

Lipids Model

The Archimedes Model includes a physiologically motivated model of cholesterol and triglyceride levels (Figure 13). Triglycerides (TG) depend on age, sex, and the severity of type 2 diabetes. The effect of diabetes on TGs is modeled using information about the relationship between TGs and FPG in a large dataset from a health maintenance organization. TGs affect the trajectories for HDL and total cholesterol (TC). LDL cholesterol is calculated using the Friedewald equation: TC= HDL+LDL+TG/5.

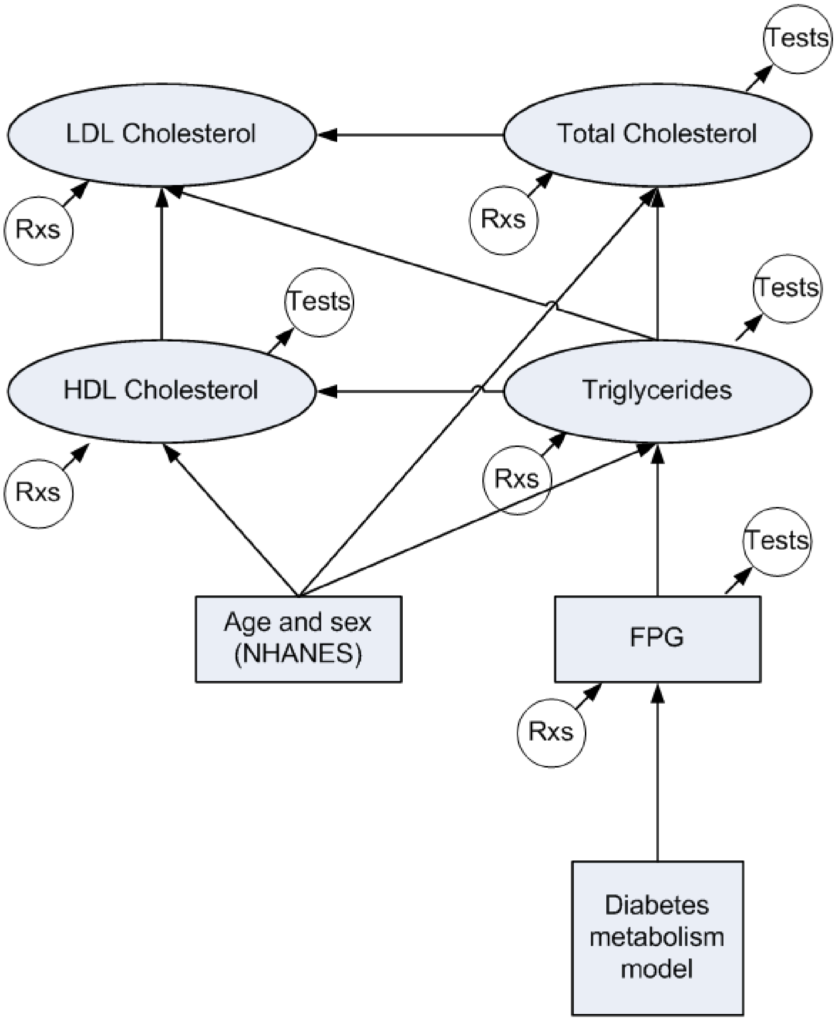

Figure 13. Diagram of the Lipids Model.

Stroke Model

The Archimedes stroke model includes two types of stroke: ischemic stroke, caused by a blocked blood vessel in the brain, and hemorrhagic stroke, caused by bleeding into the brain. Because the pathophysiology and risk factors are different for ischemic and hemorrhagic strokes, the stroke model uses separate progression functions for the two types. Also, because the probability distribution for subsequent strokes is different from that of initial strokes, the model uses separate progression functions for "first stroke" and "recurrent stroke." Three main datasets were used to build the stroke model. The ARIC dataset was used to estimate progression functions for ischemic stroke and hemorrhagic stroke. The Framingham Heart Study was used to help estimate the progression functions for ischemic stroke. The Cardiovascular Health Study (CHS) was used to help write the equations for hemorrhagic stroke. Additional papers are listed below.

First Ischemic Stroke

The progression function for first ischemic stroke depends on race (black versus nonblack), age, sex, smoking, diabetes (HbA1c), previous MI, angina, SBP, and atrial fibrillation (Figure 14). Progression functions for first ischemic stroke are determined separately for men and women based on:

- Chambless LE et al. Prediction of Ischemic Stroke Risk in the Atherosclerosis Risk in Communities Study, Am J Epidemiol 2004; 160:259-269.
- Rosamund WA et al. Stroke incidence and survival among middle-aged adults: 9-year follow-up of the Atherosclerosis Risk in Communities (ARIC) cohort, Stroke 1999; 30:736-743.
- The National Survey of Stroke: National Institute of Neurological and Communicative Disorders and Stroke, Stroke 1981; 12(suppl. 1):i1-91.

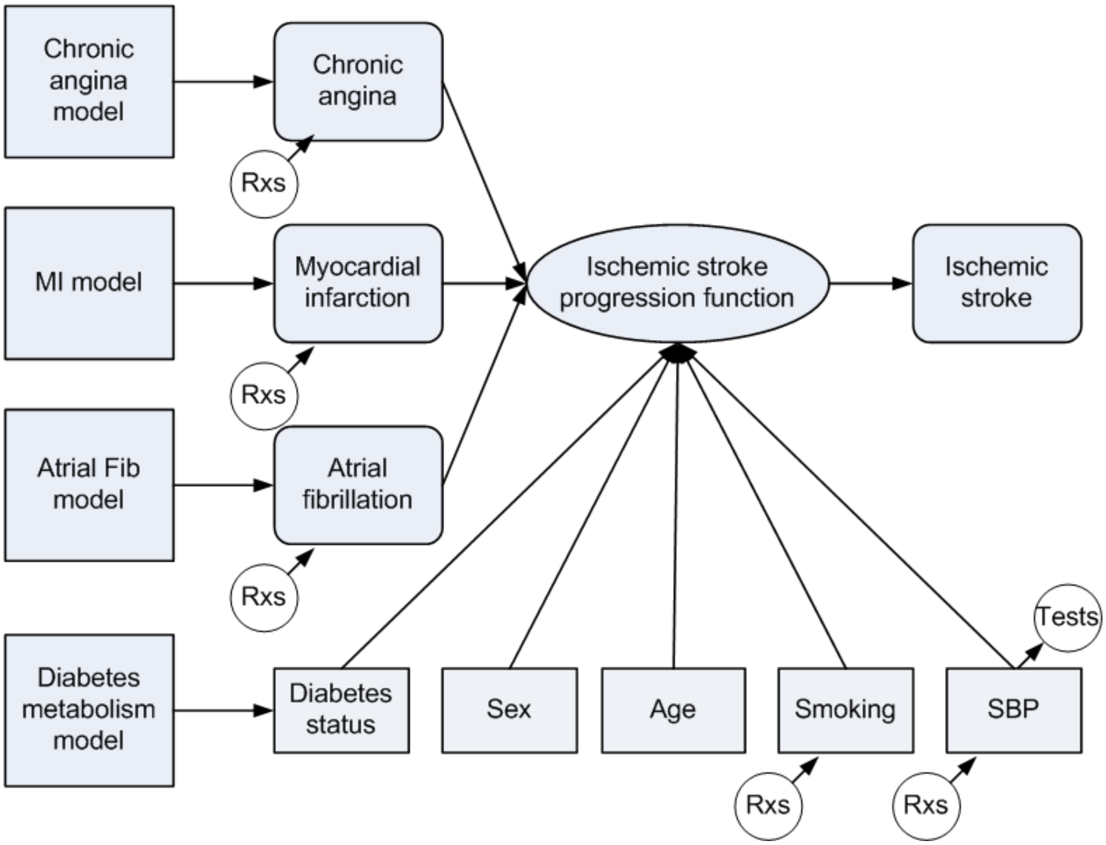

Figure 14. Diagram of First Ischemic Stroke Model.

First Hemorrhagic Stroke

The model includes two types of hemorrhagic stroke: primary intra-cerebral hemorrhage (PICH) and sub-arachnoidal hemorrhage (SAH) (Figure 15). The progression function for PICH stroke is a function of sex, age, race (black versus nonblack), SBP, and DBP. Separate progression functions are calculated for men and women. The following source is used to estimate the progression functions for PICH:

ARCHIMEDES
© 2011-2012

- Sturgeon JD et al. Risk factors for intracerebral hemorrhage in a pooled prospective study, Stroke 2007; 38:2718-2725.

To calculate the occurrence of SAHs, we assume that they are affected by the same risk factors as PICH strokes. We then apply a ratio for SAH/PICH estimated from:

- Smeeton NC et al. Incidence of hemorrhagic stroke in black Caribbean, black African, and white populations. The South London Stroke Register, 1995-2004, Stroke 2007; 38:3133-3138.

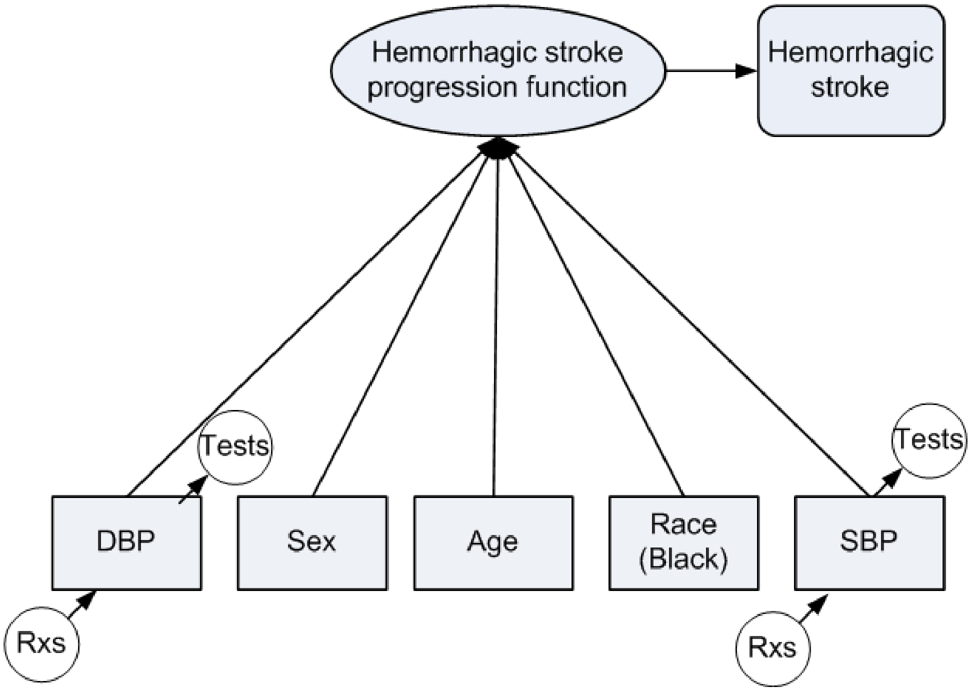

Figure 15. Diagram of First Hemorrhagic Stroke Model.

Recurrent Stroke

After an individual has a first stroke, he or she is at increased risk for a subsequent stroke (Figure 16). The progression function for recurrent stroke depends on age, sex, race, and the age at which the first stroke occurred. The type of stroke (ischemic or hemorrhagic) depends on the type of the first stroke; there is an increased probability that a recurrent stroke will be the same type as the first.

The progression functions for recurrent strokes are based on:

- Lloyd-Jones D et al. Heart disease and stroke statistics--2010 update: a report from the American Heart Association, 2010; 121:e46-e215.

- Hankey GJ et al. Five-year survival after first-ever stroke and related prognostic factors in the Perth Community Stroke Study, Stroke 2000; 31:2080-2086.

- Hardie K, et al. Ten-year risk of first recurrent stroke and disability after first-ever stroke in the Perth Community Stroke Study, Stroke 2004; 35:731-735.

- Hillen T et al. Cause of stroke recurrence is multifactorial: patterns, risk factors, and outcomes of stroke recurrence in the South London stroke register, Stroke 2003; 34:1457-1463.

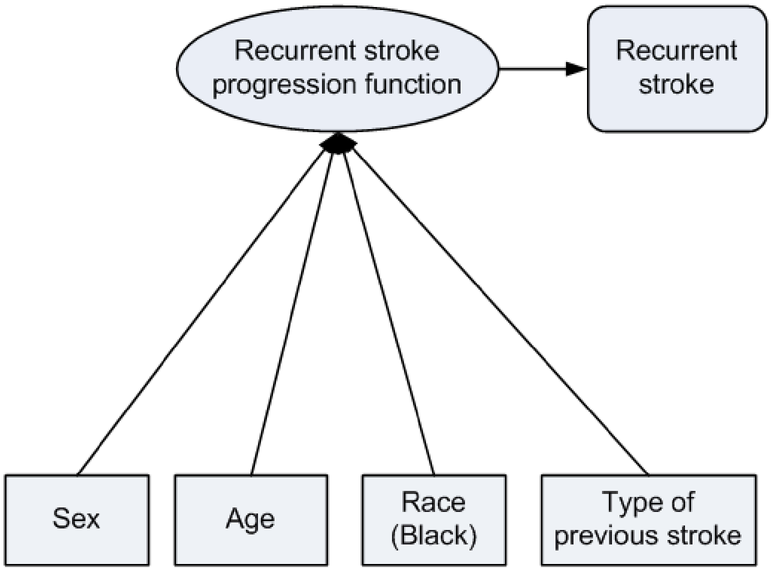

Figure 16. Diagram of Recurrent Stroke Model.

Mortality

The Archimedes Model includes a model for calculating all-cause mortality based on data from the National Vital Statistics System (NVSS) (Figure 17). In the Model, disease-specific mortalities are generated by the individual disease models. The Model then adjusts the all-cause mortality rate to determine a progression function for "other-cause" mortality. "Other-cause" mortality therefore includes deaths from all causes other than the diseases explicitly included in the Archimedes Model. Deaths due to neuropathy and nephropathy are included in the analysis, but are not shown in Figure 17 due to their small number.

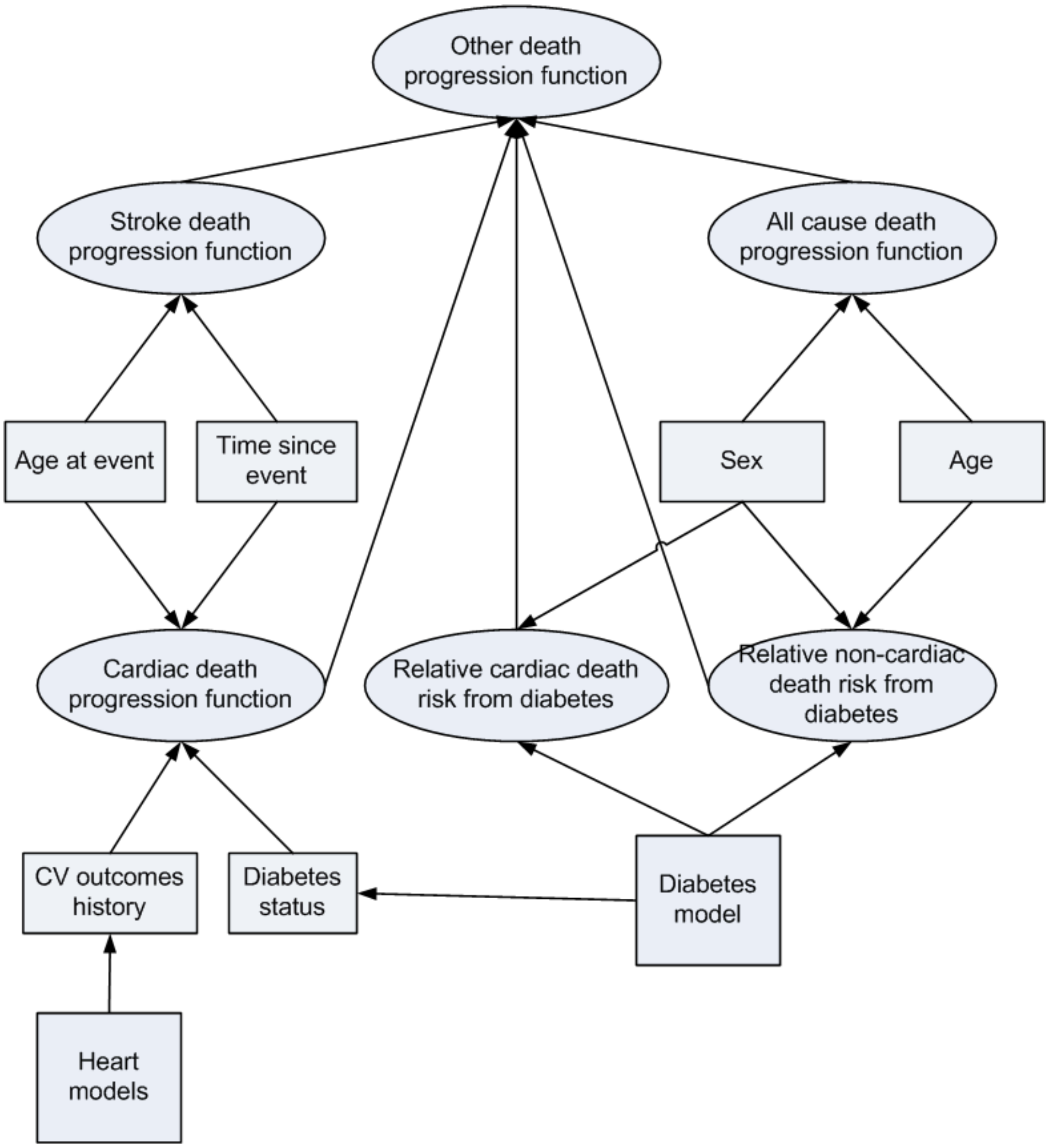

Figure 17. Diagram of "Other-Cause" Mortality.

The mortality models are based on the following publications from the National Center for Health Statistics:

- Arias E. United States Life Tables, 2006. National Vital Statistics Reports; vol. 58 no 21. Hyattsville, MD: National Center for Health Statistics. 2010.
- Arias E. United States Life Tables by Hispanic Origin. National Center for Health Statistics. Vital Health Stat 2000; 2(152).

ARCHIMEDES
© 2011-2012

- Death rates from 113 selected causes, specified Hispanic origin, race for non-Hispanic population, United States, 2006. (Available at http://www.cdc.gov/nchs/nvss/mortality/gmwkh210r.htm.)

Interventions

The Archimedes Model includes a large number of tests and treatments. Many but not all of them are accessible through the ARCHeS interface. This section describes the interventions that are accessible through ARCHeS. We use the term "intervention" very broadly to include not only drugs and procedures, but also anything that directly or indirectly affects either a risk factor for a disease or the progression and outcome of a disease. Thus interventions can include behavior changes such as diet and exercise, and public health measures such as reducing salt or substituting trans fats in foods. Interventions can also include activities whose effects on risk factors are very indirect. For example a media-based program to increase the rate at which patients adhere to recommended treatments is an intervention and can be evaluated using the Archimedes Model and ARCHeS. Even a policy initiative such as increasing insurance coverage can be addressed if the changes in coverage affect risk factors and diseases that are in the Model. For interventions whose effects on risk factors and diseases are indirect, it is necessary to have evidence about the effect of the intervention on risk factors or diseases. For example, to analyze the effects on health and economic outcomes of a media-based program to increase patient adherence to recommendations for hypertension treatment, it is necessary to have evidence from a trial or demonstration program showing the effect of the program on use of blood pressure medications, or preferably on actual blood pressure levels.

In the Model, interventions can affect biomarkers directly and can have additional "pleiotropic" effects on the progression of diseases (the disease progression functions). Pleiotropic effects of an intervention are additional changes in the risk of developing a disease (or having a disease progress, for those who already have it), beyond the effects attributable to changes in specific biomarkers.

Table 4 shows the biomarkers and disease processes affected by each intervention in the part of the Model that is included in ARCHeS. Note that the biomarker changes that result from an intervention may themselves affect other biomarkers and disease progressions due to linkages within the Model.

Table 4. Biomarkers and Disease Processes Affected by Each Intervention.

Intervention	Effects
Aspirin	Stable angina, MI, ischemic stroke, CHD death
Clopidogrel	MI, ischemic stroke, CHD death
Diuretic	Stable angina, MI, blood pressure, ischemic stroke, hemorrhagic stroke, CHD death
ACE	Stable angina, MI, blood pressure, ischemic stroke, hemorrhagic stroke, CHD

ARCHIMEDES © 2011-2012

inhibitor/ARB	death, renal disease
Beta blocker	Stable angina, MI, blood pressure, ischemic stroke, hemorrhagic stroke, CHD death
Calcium channel blocker	Stable angina, MI, blood pressure, ischemic stroke, hemorrhagic stroke, CHD death
Metformin	Weight, FPG, total cholesterol, HDL, TGs, blood pressure
Sulfonylurea	Weight, insulin amount, FPG, possibility of hypoglycemia
Glitazones	Weight, FPG, total cholesterol, HDL, TGs
Insulin	Weight, insulin amount, FPG, possibility of hypoglycemia
Statin	Total cholesterol, HDL, TGs, MI, ischemic stroke, CHD death
Diabetes diet	Weight, blood pressure

The effects of aspirin are based on:

- Berger JS et al. Aspirin for the primary prevention of cardiovascular events in women and men: a sex-specific meta-analysis of randomized controlled trials, JAMA 2006; 295(3):306-313.
- ATT Collaboration. Aspirin in the primary and secondary prevention of vascular disease: collaborative meta-analysis of individual participant data from randomized trials, Lancet 2009; 373:1849-1860.
- Collaborative Group of the Primary Prevention Project. Low-dose aspirin and vitamin E in people at cardiovascular risk: a randomized trial in general practice, Lancet 2001; 357:89-95.

The clopidogrel model is based on:

- Bhatt DL et al. Clopidogrel and aspirin versus aspirin alone for the prevention of atherothrombotic events, N Engl J Med 2006; 354:1-12.
- COMMIT. Addition of clopidogrel to aspirin in 45,852 patients with acute myocardial infarction: randomized pacebo controlled trial, Lancet 2005; 366:1607-1621.
- Steinhubl SR et al. Early and sustained dual oral antiplatelet therapy following percutaneous coronary intervention: a randomized controlled trial, JAMA 2002; 288:2411-2420.
- CURE. Effects of clopidogrel in addition to aspirin in patients with acute coronary syndromes without ST-segment elevation, N Engl J Med 2001; 345:494-502.

Antihypertensives

Antihypertensive medications included in the Model include thiazide diuretics, ACE inhibitors/ARBs, beta blockers, and calcium-channel blockers (CCBs). In the Model, antihypertensive use reduces blood

pressure and also has pleiotropic effects, reducing the risk of MI and stroke. The effects of antihypertensive medications are determined based on a network meta-analysis using data from a large number of clinical trials, including the following:

- Psaty BM et al. Health outcomes associated with various antihypertensive therapies used as first-line agents, JAMA 2003; 289(19):2534-2544.
- Blood Pressure Lowering Treatment Trialists' Collaboration. Effects of different regimens to lower blood pressure on major cardiovascular events in older and younger adults: meta-analysis of randomised trials, BMJ 2008; 336:1121-1123.
- Sever PS et al. Prevention of coronary and stroke events with atorvastatin in hypertensive patients who have average or lower-than-average cholesterol concentrations, in the Anglo-Scandinavian Cardiac Outcomes Trial—Lipid Lowering Arm (ASCOT-LLA): a multicentre randomised controlled trial, Lancet 2003; 361:1149.
- The ALLHAT Officers and Coordinators for the ALLHAT Collaborative Research Group. Major outcomes in high-risk hypertensive patients randomized to angiotensin-converting enzyme inhibitor or calcium channel blocker vs diuretic: The Antihypertensive and Lipid-Lowering Treatment to Prevent Heart Attack Trial (ALLHAT), JAMA 2002; 288:2981-2997.
- Wing LMH et al. A comparison of outcomes with angiotensin-converting‑enzyme inhibitors and diuretics for hypertension in the elderly, N Engl J Med 2003; 348:583-92.
- Hansson L et al. Effect of angiotensin-converting-enzyme inhibition compared with conventional therapy on cardiovascular morbidity and mortality in hypertension: the Captopril Prevention Project (CAPPP) randomised trial, Lancet 1999; 353:611-616.
- Black HR et al. Principal results of the Controlled Onset Verapamil Investigation of Cardiovascular End Points (CONVINCE) Trial, JAMA 2003; 289(16):2073-2082.
- D'Agostino et al. General cardiovascular risk profile for use in primary care: The Framingham Heart Study, Circulation 2008; 117:743-753.
- The Heart Outcomes Prevention Evaluation Study Investigators. Effects of an angiotensin-converting-enzyme inhibitor, ramipril, on cardiovascular events in high-risk patients, N Engl J Med 2000; 342:145-153.
- Beckett NS et al. Treatment of hypertension in patients 80 years of age or older, N Engl J Med 2008; 358:1887-1898.
- Pitt B et al. for the PREVENT Investigators. Effect of amlodipine on the progression of atherosclerosis and the occurrence of clinical events, Circulation 2000; 102:1503-1510.
- SHEP Cooperative Research Group. Prevention of stroke by antihypertensive drug treatment in older persons with isolated systolic hypertension, JAMA 1991; 265(24):3255-3264.
- Brenner BM et al. Effects Of losartan on renal and cardiovascular outcomes in patients with type 2 diabetes and nephropathy, N Engl J Med 2001; 345:861-869.

© 2011-2012

- Staessen JA et al. Randomised double-blind comparison of placebo and active treatment for older patients with isolated systolic hypertension, Lancet 1997; 350:757–764.
- Wang J-G et al. Prevention of stroke and myocardial infarction by amlodipine and angiotensin receptor blockers: a quantitative overview, Hypertension 2007; 50:181-188.
- Pepine C et al. A calcium antagonist vs a non-calcium antagonist hypertension treatment strategy for patients with coronary artery disease. The International Verapamil-Trandolapril Study (INVEST): a randomized controlled trial, JAMA 2003; 290:2805-2816.
- Dahlof B et al. Cardiovascular morbidity and mortality in the Losartan Intervention For Endpoint reduction in hypertension study (LIFE): a randomised trial against atenolol, Lancet 2002; 359:995–1003.
- PROGRESS Collaborative Group, Randomised trial of a perindopril-based blood-pressure-lowering regimen among 6105 individuals with previous stroke or transient ischaemic attack, Lancet 2001; 358:1033–1041.
- Zanchetti A et al. Calcium antagonist lacidipine slows down progression of asymptomatic carotid atherosclerosis: principal results of the European Lacidipine Study on Atherosclerosis (ELSA), a randomized, double-blind, long-term trial, Circulation 2002; 106:r47-r52.
- Schrader J et al. Morbidity and mortality after stroke, eprosartan compared with nitrendipine for secondary prevention: principal results of a prospective randomized controlled study (MOSES), Stroke 2005; 36:1218-1226.
- Anderson KM et al. Cardiovascular disease risk profiles, Am Heart J 1991; 121(1):293-298.

Antidiabetic Medications

Antidiabetic agents included in the Model are metformin, sulfonylureas, glitazones, and insulin. Diabetes medications do not act directly on the diabetes progression function, but instead affect an individual's FPG. The effect of metformin on FPG is based on data from:

- Wulffele MG et al. The effect of metformin on blood pressure, plasma cholesterol and triglycerides in type 2 diabetes mellitus: a systematic review, J Intern Med 2004; 256:1–14.
- Johnson JA et al. Reduced cardiovascular morbidity and mortality associated with metformin use in subjects with Type 2 diabetes, Diabet Med; 22:497-502.
- Saenz A et al. Metformin monotherapy for type 2 diabetes mellitus (Review), The Cochrane Library 2008; Issue 4.
- Salpeter SR et al. Meta-analysis: metformin treatment in persons at risk for diabetes mellitus, Am J Med 2008; 121:149-157.

Information from these studies is also used to determine the effects of metformin on weight, cholesterol values, FPG, and blood pressure.

The effects of sulfonylureas on FPG and weight are based on the following studies:

- Schade DS et al. A placebo-controlled, randomized study of glimepiride in patients with type 2 diabetes mellitus for whom diet therapy is unsuccessful, J Clin Pharmacol 1998; 38:636-641.
- Schernthaner G et al. GUIDE study: double-blind comparison of once-daily gliclazide MR and glimepiride in type 2 diabetic patients, Eur J Clin Invest 2004; 34:535–542.
- Garber AJ et al. Efficacy of glyburide/metformin tablets compared with initial monotherapy in type 2 diabetes, J Clin Endocrinol Metab 2003;88(8):3598–3604.
- Rosenstock J et al. Glimepiride, a new once-daily sulfonylurea. A double-blind placebo-controlled study of NIDDM patients, Diabetes Care 1996; 19(11):1194-1199.
- Simonson DC et al. Efficacy, safety, and dose-response characteristics of glipizide gastrointestinal therapeutic system on glycemic control and insulin secretion in NIDDM. Results of two multicenter, randomized, placebo-controlled clinical trials, Diabetes Care 1997; 20(4):597-606.
- Blonde L et al. Glyburide/metformin combination product is safe and efficacious in patients with type 2 diabetes failing sulphonylurea therapy, Diabetes Obes Metab 2002; 4:368-375.
- Fischer S et al. Influence of treatment with acarbose or glibenclamide on insulin sensitivity in type 2 diabetic patients, Diabetes Obes Metab 2003; 5:38-44.
- Kitabchi AE et al. Comparative efficacy and potency of long-term therapy with glipizide or glyburide in patients with type 2 diabetes mellitus, Am J Med Sci 2000;319(3):143-148.
- Eriksson JG et al. Long-term beneficial effects of glipizide treatment on glucose tolerance in subjects with impaired glucose tolerance, J Intern Med 2006; 259:553–560.
- Bautista JL et al. Efficacy and safety profile of glimepiride in Mexican American patients with type 2 diabetes mellitus: a randomized, placebo-controlled study, Clin Ther 2003; 25(1):194-209.
- Garber AJ et al. Simultaneous glyburide/metformin therapy is superior to component monotherapy as an initial pharmacological treatment for type 2 diabetes, Diabetes Obes Metab 2002; 4:201-208.

The effects of glitazones on weight, FPG, total cholesterol, HDL, and triglycerides are based on data from:

- Goldberg RB et al. A comparison of lipid and glycemic effects of pioglitazone and rosiglitazone in patients with type 2 diabetes and dyslipidemia, Diabetes Care 2005; 28(7):1547-1554.
- Deeg MA et al. Pioglitazone and rosiglitazone have different effects on serum lipoprotein particle concentrations and sizes in patients with type 2 diabetes and dyslipidemia, Diabetes Care 2007; 30(10):2458-2464.
- Tan MH et al. Comparison of pioglitazone and gliclazide in sustaining glycemic control over 2 years in patients with type 2 diabetes, Diabetes Care 2005; 28(3):544-550.

© 2011-2012

- Tan MH et al. Sustained effects of pioglitazone vs. glibenclamide on insulin sensitivity, glycaemic control, and lipid profiles in patients with type 2 diabetes, Diabet Med 2004; 21:859-866.
- Charbonnel BH et al. Pioglitazone elicits long-term improvements in insulin sensitivity in patients with type 2 diabetes: comparisons with gliclazide-based regimens, Diabetologia 2005; 48(3): 553-560.
- Mannucci MA et al. Pioglitazone and cardiovascular risk. A comprehensive meta-analysis of randomized clinical trials, Diabetes Obes Metab 2008; 10(12):1221-1238.
- Khan M et al. Effects of pioglitazone on the components of diabetic dyslipidaemia: results of double-blind, multicentre, randomised studies, Int J Clin Pract 2004; 58(10):907-912.
- Jain R et al. Long-term safety of pioglitazone versus glyburide in patients with recently diagnosed type 2 diabetes mellitus, Pharmacotherapy 2006; 26(10):1388-1395.
- Belcher G et al. Cardiovascular effects of treatment of type 2 diabetes with pioglitazone, metformin and gliclazide, Int J Clin Pract 2004; 58(9):833-837.

Insulin, unlike most medications in the Model, is not given in a predetermined dose. Instead, the healthcare provider specifies a target FPG or HbA1c value, and the insulin dose required to achieve that goal is calculated based on the patient's current levels of FPG or HbA1c. The effect of insulin on weight is based on the UKPDS33 study.

Dyslipidemia Medications

The following daily dosages of simvastatin are included in the interventions: 5 mg, 10 mg, 20 mg, 40 mg, 80 mg. The effects of statins on TC, HDL cholesterol, and TGs, as well as a direct effect of statins on MI and ischemic stroke, are modeled based on:

- Jones PH et al. Comparison of the efficacy and safety of rosuvastatin versus atorvastatin, simvastatin, and pravastatin across doses (STELLAR Trial), Am J Cardiol 2003; 93:152-160.
- Colhoun HM et al. Primary prevention of cardiovascular disease with atorvastatin in type 2 diabetes in the Collaborative Atorvastatin Diabetes Study (CARDS): multicentre randomised placebo-controlled trial, Lancet 2004; 364:685-696.
- Pedersen TR et al. Randomised trial of cholesterol lowering in 4444 patients with coronary heart disease: the Scandinavian Simvastatin Survival Study (4S), Lancet 1994; 344;1383-89.
- Heart Protection Study Collaborative Group, MRC/BHF Heart Protection Study of cholesterol lowering with simvastatin in 20,536 high-risk individuals: a randomised placebo-controlled trial, Lancet 2002; 360:7-22.
- Sever PS et al. Prevention of coronary and stroke events with atorvastatin in hypertensive patients who have average or lower-than-average cholesterol concentrations, in the Anglo-Scandinavian Cardiac Outcomes Trial—Lipid-Lowering Arm (ASCOT-LLA): a multicentre randomised controlled trial, Lancet 2003; 361:1149-1158.

© 2011-2012

- LaRosa JC et al. Intensive lipid lowering with atorvastatin in patients with stable coronary disease, N Engl J Med 2005; 3352(14):1425-1435.

As noted above, LDL cholesterol levels are calculated using the Friedewald equation : TC= HDL+LDL+TG/5. The three variables TC, HDL, and TG are “independent” variables in the Archimedes Model; statins act directly on these, and through them modify LDL. In the Model, statins do not affect the risk of hemorrhagic stroke. The effects of statins are assumed to be the same whether they are used for primary or secondary prevention.

Diabetes Diet

The diabetes diet intervention produces a 3% weight loss (and thus a decrease in 3% BMI). This degree of weight loss is based on:

- UKPDS Group. UK Prospective Diabetes Study 7: response of fasting plasma glucose to diet therapy in newly presenting type II diabetic patients, Metabolism 1990; 39(9):905-912.
- UKPDS Group. Effect of three months’ diet after diagnosis of type 2 diabetes on plasma lipids and lipoproteins (UKPDS 45), Diabet Med 2000; 17:518-523.
- UK Prospective Diabetes Study (UKPDS) Group. Intensive blood-glucose control with sulphonylureas or insulin compared with conventional treatment and risk of complications in patients with type 2 diabetes (UKPDS 33), Lancet 1998; 352(9131):837-853.

Weight loss in turn affects other biomarkers such as FPG, lipids, and blood pressure. The effect of weight loss on blood pressure is based on:

- Neter JE et al. Influence of weight reduction on blood pressure: a meta-analysis of randomized controlled trials, Hypertension 2003; 42:878-884.

Healthcare System Model

The Archimedes Model includes a detailed model of the healthcare system. As described above in the overview of the Model, simulated healthcare providers follow protocols that are based on published U.S. guidelines for diagnosing and treating diseases. The care provided by the simulated providers is adjusted to represent the average care provided in the United States today. That is, because guidelines are not applied perfectly in the real world, the Model is calibrated to account for healthcare providers who may not apply guidelines exactly as they are written, and to account for patients who may not adhere to prescribed tests, treatments, or follow-up care.

Guidelines Implemented in the Model

A literature search was performed to identify guidelines that have been issued by various organizations for each disease included in the Model. The main source for this was www.guidelines.gov. The guidelines were reviewed by in-house medical staff, as well as external advisers. A major consideration was to choose guidelines that are considered to be national in scope, such as guidelines issued by

government-sponsored panels (e.g., ATP III guideline for cholesterol management), national-level professional societies (e.g., American Academy of Ophthalmology), and voluntary health organizations (e.g., American Heart Association and American Diabetes Association). The guidelines incorporated in the Archimedes Model and used by ARCHeS are in Table 5.

Table 5. Guidelines Implemented in the Model.

Disease	Sources
Hypertension	• The seventh report of the Joint National Committee on Prevention, Detection, Evaluation, and Treatment of High Blood Pressure (JNC-7), 2003.<br>• American Diabetes Association, Standards of medical care in diabetes—2009, Diabetes Care 2009; 32 Suppl 1:S13-61.<br>• Sacco RL et al. Guidelines for prevention of stroke in patients with ischemic stroke or transient ischemic attack, Stroke 2006; 37:577-617.
Dyslipidemia	• Executive summary of the third report of the National Cholesterol Education Program (NCEP) Expert Panel on Detection, Evaluation, and Treatment of High Blood Cholesterol in Adults (Adult Treatment Panel III), 2002.
Obesity	• Institute for Clinical Systems Improvement (ICSI): Health care guideline: prevention and management of obesity (mature adolescents and adults), 2009.
Diabetes	• American Diabetes Association, Standards of medical care in diabetes—2009, Diabetes Care 2009; 32 Suppl 1:S13-61.
Diabetic retinopathy	• American Academy of Ophthalmology Retina Panel. Preferred Practice Pattern® Guidelines. Diabetic Retinopathy. San Francisco, CA: American Academy of Ophthalmology; 2008. (Available at: http://www.aao.org/ppp.)<br>• American Diabetes Association, Standards of medical care in diabetes—2009, Diabetes Care 2009; 32 Suppl 1:S13-61.
Diabetic neuropathy	• Boulton AJM et al. Diabetic neuropathies: a statement by the American Diabetes Association, Diabetes Care 2005;28(4):956-962.<br>• American Diabetes Association, Standards of medical care in diabetes—2009, Diabetes Care 2009; 32 Suppl 1:S13-61.
Diabetic nephropathy	• American Diabetes Association, Standards of medical care in diabetes—2009, Diabetes Care 2009; 32 Suppl 1:S13-61.

Disease	Sources
Heart disease (includes MI, angina, CAD, and chest pain)	• ACC/AHA 2007 Guidelines for the management of patients with unstable angina/non-ST-elevation myocardial infarction, Circulation 2007; 116:803-877.<br>• ACC/AHA Guidelines for the management of patients with ST-elevation myocardial infarction, Circulation 2004; 110:e82-e293.<br>• ACC/AHA 2005 Guideline update for the diagnosis and management of chronic heart failure in the adult, Circulation 2005; 112:e154-e235.<br>• Gibbons RJ, Abrams J, Chatterjee K, et al. ACC/AHA 2002 guideline update for the management of patients with chronic stable angina: a report of the American College of Cardiology/American Heart Association Task Force on Practice Guidelines (Committee to Update the 1999 Guidelines for the Management of Patients with Chronic Stable Angina), 2002. (Available at www.acc.org/clinical/guidelines/stable/stable.pdf.)
Stroke	• Adams HP et al. Guidelines for the early management of adults with ischemic stroke, Stroke 2007; 38(5):1655-1711.
Atrial fibrillation	• Adams RJ et al. Update to the AHA/ASA recommendations for the prevention of stroke in patients with stroke and transient ischemic attack, Stroke 2008; 39:1647-1652.

Datasets Used for Model Calibration

Approximately 30 national datasets were surveyed to identify a collection of data that would most completely span the diseases and healthcare processes represented in the Archimedes Model. The goal was to identify sources of data that represent standard care within the general US population and also included information on subpopulations. The main datasets used to estimate the calibration targets are listed in Table 6.

Table 6. Datasets Used for Model Calibration.

Dataset	Year	Survey Content	Survey Sample Design	Sample Size
NHANES<br><br>National Health And Nutrition Examination Survey	1999 - 2006	Chronic disease prevalence and conditions (including undiagnosed conditions), risk factors, diet and nutritional status, immunization status, infectious disease prevalence, health insurance, and measures of environmental exposures. Other topics addressed include hearing, vision, mental health, anemia, diabetes, cardiovascular disease, osteoporosis, obesity, oral health, mental health, and physical fitness.	Uses a stratified multistage probability sample, nationally representative of the U.S. civilian non-institutionalized population.	Approximately 5,000 people are examined each year.
NAMCS<br><br>National Ambulatory Medical Care Survey	2006	Information is obtained on various aspects of office visits, including physician practice characteristics, patient characteristics, and other visit characteristics. Among the items collected are patient's age, sex, race, and ethnicity; patient's expressed reason for visiting the physician; intentionality of injury, if any; physician's diagnoses; diagnostic services ordered or provided; therapeutic services; ambulatory surgical procedures performed; medications; providers seen; visit disposition; time spent with physician; and expected source of payment.	National probability sample survey of visits to office-based physicians in the United States.	In 2004, 25,286 survey forms (each representing one physician-patient visit) were collected.

© 2011-2012

NHAMCS<br><br>National Hospital Ambulatory Care Survey	2006	Includes two files: ED visits and OPD visits. Information is obtained on various aspects of patient visits, including patient, hospital, and visit characteristics. Among the items collected are patient's age, sex, race, and ethnicity; patient's expressed reason for visit; intentionality of injury, if any; physician's diagnoses; diagnostic services ordered or provided; procedures provided; medications; providers seen; visit disposition; immediacy with which patient should be seen; and expected source of payment. Items collected that are specific to the ED include mode of arrival, waiting time, duration of time in the ED. initial vital signs, and cause of injury.	National probability sample survey of visits to emergency departments (EDs) and outpatient departments (OPDs) of non-federal, short-stay, and general hospitals in the United States.	About 400 EDs and 225 OPDs participate each year. In 2004, 36,589 ED forms and 31,783 OPD forms were completed.
NHDS<br><br>National Hospital Discharge Survey	2006	Variables collected include age; sex; race; ethnicity; admission and discharge dates (length of stay); discharge status; source of payment; hospital size, ownership, and region; from one to seven diagnoses coded using the ICD-9-CM; and, from zero to four procedures using the ICD-9-CM.	Uses a three-stage national probability design that includes primary sampling units (PSUs), hospitals within the PSUs, and discharges within the hospitals.	Approximately 300,000 discharges are sampled each year from about 500 hospitals.

Limitations of the Model

The Model has several limitations.

Population

The default population in the Model is the US population, as described by the NHANES survey. The Model can be configured to analyze other populations, based on either person-specific data or aggregated data from the new population of interest. This can be done either in consulting projects or through the ARCHeS interface.

Care processes

© 2011-2012

The default care processes in the Model are based on those currently used in the United States. Sources for the specific guidelines are described in the section the healthcare system model. The Model's implementation of these guidelines is calibrated as described in that section to match population characteristics and biomarkers, performance levels, and compliance levels seen in the United States. Care processes can be modified and customized to different settings in consulting projects, but at present cannot be modified through the ARCHeS interface.

Missing and inconsistent data

Setting up and calibrating the Model, specifically the population and care processes, requires using data from multiple sources, such as NHANES, NAMCS, NHAMCS, and NHDS. Because different data sources are derived from different populations and use different definitions and methods, their results are often not entirely consistent, and judgments have to be made about the most appropriate target to use for calibration and the range of uncertainty to explore.

Diseases, interventions, and outcomes

While the Archimedes Model is a single integrated model that includes multiple conditions, there are many diseases that are not in the Model. This document describes only the diseases, interventions, and outcomes included in the Model that are available via the ARCHeS interface. Additional diseases, interventions, and outcomes are available in the Model for consulting projects.

Standard care remains constant

When calculating outcomes and the effects of interventions in the future, the Model assumes that, in general, care will be delivered at the same level as today. The exceptions would be whatever interventions are being studied in any particular analysis.

Adults only

Currently, the Archimedes Model creates populations of age 20-85. It does not include pediatric conditions, interventions, or outcomes.

Population remains the same through the simulation period

In ARCHeS, the default is that a simulation includes only people who are in the population at the simulation start date. New individuals do not enter the population after the start of the simulation. People in the initial population leave the simulation only when they die. The Model can be configured to incorporate people entering and leaving the simulation such as might happen with individuals moving in and out of health plans. However, this must be done through a consulting project; it cannot be set up in the ARCHeS interface.

No patient interactions

The Model does not yet allow for interactions between patients. Thus it is currently not able to address the spread of infections in a population.

© 2011-2012

No competing resources

The Model tracks the use of resources at a high level of detail for each individual, and these data can be combined to determine the total amount of resources used. However, the Model assumes that the amount of resources is unlimited. It does not explicitly include resource shortages, competition for resources, queues, or bottlenecks.

Disease-based

The Archimedes Model is built up from the underlying physiology and pathophysiology that causes diseases. It is designed to address questions that relate to the incidence and progression of diseases, and their management. Because only certain diseases are in the Model, the Model is not designed to answer system-wide questions or general questions about the delivery or financing of a total healthcare system. For example, it is not designed to answer questions about healthcare workforces or facilities, or very general policies about coverage. Questions of this nature can be addressed but need to be narrowed so as to apply to the individual diseases that are in the Model. For example, because diabetes is in the Model, the Model could be used to address the need for diabetes specialists in the future and how that is affected by trends in obesity and its management.

Socioeconomic status and insurance

Currently, the Model does not include socioeconomic status or insurance coverage. If there are descriptive data about a subpopulation that has a particular socioeconomic or insurance coverage status, then those descriptive data can be used to create a simulated population that matches the subpopulation of interest. This would need to be done through a consulting project; it cannot be set up in the ARCHeS interface.

Unknown or unmeasured covariates

To the greatest extent permitted by the available data, the Model incorporates information about factors that affect the incidence and progression of diseases – "covariates." However, in any population, whether it is based on geography or the inclusion/exclusion criteria of a clinical trial, there are inevitably covariates that affect the outcomes of interest that are unknown, unmeasured, or unreported. It is not possible to incorporate the effects of missing or unmeasured covariates. For this reason, there will always be uncertainty about the Model's results for any new population. This limitation of the Model will gradually decrease as future epidemiological studies identify new covariates.

Unknown risks and side effects

The Model does not include any outcomes or the effects of any interventions that have not been observed and reported. If an intervention has been shown to affect a biomarker that is in the Model, then the Model can be used to predict implications of the effect on that biomarker on longer-term health outcomes. For example, if a side effect of a glucose control agent is an increase in LDL cholesterol, then the effects on LDL cholesterol will be incorporated in the Model's calculations of the effects of the glucose control agent on cardiovascular outcomes.

© 2011-2012

Acknowledgements

The Model has been evolving since originally conceived by Dr. Eddy and first given life by Dr. Schlesinger so many years ago. Many scientists, engineers, and physicians have been involved in crafting the current Model. We are indebted to Kathleen Thompson for her work on the early drafts of this document and for leading the modeling effort in the past four years; to the cancer modeling team, Tuan Dinh and Joyce Noah-Vanhoucke; to the heart modeling team, Ian Blumenfeld and Adam Guetz; to the diabetes modeling team, Stuart Samuel and Kenny Shum; to the healthcare modeling team, Julia Dziuba and Swathi Guda; to Carl Anderson, Andrew Van Herick, Helene Grossman, Andy Schuetz, Roshan Shah, and Amory Schlender for their contributions; to Barbara Peskin, Andrea Kress, Jennifer Burton, and Patrick van der Valk for supporting the efforts to produce this version of the Model and this document; to Brian Zuzga and Paul Jasper for providing leadership and direction in architectural integrity and coding standards; to Katrina Montinola for her leadership and support in realizing the science embodied in the Model in “military grade” software; and finally, to all the former Archimedeans on whose shoulders we stand.

Sources of Funding

Archimedes, Inc. is a for-profit healthcare modeling company owned by Kaiser Permanente. Kaiser Health Plans is a not-for-profit company.

Primary Contributors

- Kaiser Permanente
- Robert Wood Johnson Foundation
- American Diabetes Association

ARCHIMEDES
... a KAISER PERMANENTE Innovation

123 Mission Street, 11th floor
San Francisco, CA 94105, USA
www.archimedesmodel.com
info@archimedesmodel.com
+1 415 490 0400

© 2011-2012
