# Supplementary material for: A Standardized Vascular Disease Health Check in Europe: A Cost-Effectiveness Analysis
Source: PLoS One. 2013 Jul 15;8(7):e66454. doi: 10.1371/journal.pone.0066454 (PMC3712021; doi:10.1371/journal.pone.0066454)
Supplement: File S3 — Description of the model calibration. (PDF) [file pone.0066454.s003.pdf]

Care Processes:

Calibration Methodology and Results

ARCHeS Simulator 2.3

April 2012

David Eddy, MD, PhD
Marc-david Cohen, PhD
Julia Dziuba, BSc

Table of Contents

Introduction 7

Role of Care Processes in the Archimedes Model 7

Objectives and Limitations of Calibration 8

Objectives 8

Limitations 9

Overview of Care Processes 10

Initiation of Care Processes 10

Representation of Care Processes 11

Non-acute evaluation and diagnosis 11

Non-acute treatment and monitoring 11

Acute care processes and prioritization of care 12

Multiple care processes 12

Scheduling of care processes 13

Resolution of ambiguities in guidelines 13

Parameterization of Care Processes 14

Calibration Parameters 14

Sources for Guidelines 16

Sources for Calibration Data 18

Calibration Measures 20

Subpopulations 21

Target Values for Calibration Measures 22

General Methods 22

Calculation of Target Values Used for Calibration 22

Target values for demographic information and biomarkers 22

© 2012

Target values for prevalence of diagnosed conditions 23
Target values for incidence of diagnosed conditions (other than death) 23
Target values for diagnosed deaths 23
Target values for incidence of treatments/procedures 24
Target values for prevalence of individuals receiving interventions 24
Analysis of Measures Not Used for Calibration 24
Target values for hospitalizations 24
Target values for outpatient visits 25
Target values for tests 25
Example of Estimating a Target Value: Incidence of MIs 26
Use of NAMCS and NHAMCS Datasets 26
Specification of Lower and Upper Bounds and Best Estimates for MI Incidence Based on NAMCS and NHAMCS 27
Use of NHDS Dataset 27
MI Incidence Estimates 28
Caveats 28
Calibration Methods 29
Prioritization of Measures 29
Objectives of Measures 29
Calculation of Goodness of Fit of the Model to the Target Value 30
Criteria for a measure passing 31
Calibration Results 31
Demographics 31
Biomarkers 32
US Population Age 20 to 85 32
Age 20 to 40 33

© 2012

Age 40 to 65 33
Age 65 to 85 33
Male 34
Female 34
Diabetic 34
CAD 35
Diagnoses – Prevalence 35
US Population Age 20 to 85 35
Age 20 to 40 35
Age 40 to 65 36
Age 65 to 85 36
Male 36
Female 36
Diabetic 37
CAD 37
Diagnoses – Incidence 37
US Population Age 20 – 85 37
Age 20 to 40 38
Age 40 to 65 38
Age 65 to 85 38
Male 38
Female 39
Diabetic 39
CAD 39
Deaths 40

© 2012

US Population Age 20 – 85 40
Age 20 – 45 40
Age 45 – 65 40
Age 65 – 85 40
Male 40
Female 40
Diabetic 41
CAD 41
Procedures – Incidence 41
US Population Age 20 – 85 41
Age 20 – 40 41
Age 40 – 65 41
Age 65 – 85 41
Male 42
Female 42
Diabetic 42
CAD 42
Interventions – Prevalence 42
US Population Age 20 – 85 42
Age 20 – 40 43
Age 40 – 65 43
Age 65 – 85 44
Male 44
Female 44
Diabetic 45

© 2012

CAD......45

Summary and Conclusions......46

Results......46

Future Work......47

References for Measures......48

© 2012

Introduction

This report describes the calibration of the care processes in the Archimedes Model, specifically focusing on the care processes used in the Simulator 2.3 version of the Model and used by ARCHeS, the web-based interface to the Model. As reported elsewhere[1], the Archimedes Model has several parts. Two of the most important are the sub-model of physiology, which determines the occurrence and progression of diseases, and the representations of care processes, which determine how patients are cared for to prevent or manage diseases[2]. It is the latter that is the subject of this report. Methods for building and validating the physiology part of the Model are discussed elsewhere[3].

The calibration of care processes involves several steps. The first is to identify measures against which outcomes of the Model should be compared, and targets for those measures. The target values for the measures should be based on empirical observations in the setting of interest. The second is to run the Model to calculate values of the measures. The third is to compare the calculated values to the empirically-observed values, and make judgments about the goodness of fit. The fourth is to modify parameters of the care processes and repeat the steps until the calculated and observed values match within an acceptable range.

The report describes each of these steps, as well as the role of care processes and the importance of calibrating care processes to match actual practices in settings of interest, limitations of calibration of care processes, how care processes are implemented in the Archimedes Modeling framework, sources and methods for performing the calibration, the results of the calibration, and conclusions.

Role of Care Processes in the Archimedes Model

An integral part of the Archimedes Model is the set of care processes that describe how diseases are prevented and managed. These care processes are a critical part of every analysis. There are three main reasons. The first is that the effectiveness of an intervention depends on how it is delivered. For example, the effect of a drug will be different if it is simply given at a particular dose, versus titrated to achieve a goal, versus included in a step-wise algorithm (e.g., first-order drug, second-order drug, and so forth). The second reason is that the effect of an intervention also depends on the other care processes used to manage patients. More specifically, the effects of interventions are always measured relative to some baseline or reference standard of care. Thus the same intervention will have different effects in different settings, depending on what care processes are being practiced in the settings. For example, a protocol for determining when patients seen in an emergency room for chest pain should be referred to a specialist will have a different effect in a setting where the current practice is to have every chest-pain patient referred to a specialist than in a setting where the current practice is to refer no one to a

---

[1] See: www.archimedesmodel.com/tech-reports

[2] We use the term “patient” in a general sense to include people who are not yet in contact with the healthcare system, as well as those who are.

[3] See: www.archimedesmodel.com/tech-reports

© 2012

specialist[4]. A corollary is that to the greatest extent possible the background care processes in a simulation should match the background care processes in the setting of interest. Stated another way, analyses should be customized to match the settings of interest. Failure to explicitly include and calibrate care processes to the settings of interest amounts to an implicit assumption that the care processes in the setting of interest are the same as the care processes that were being followed in the setting where the effect of the intervention was originally measured[5]. A third reason care processes are important is that they themselves are often the targets of interventions. The entire field of process improvement is devoted to increasing the efficiency, cost, and safety of processes.

It is for these reasons that, for the interventions it is intended to analyze, the Archimedes Model explicitly separates the physiology of diseases from the management of those diseases – the care processes followed to prevent, diagnose, and treat them. By addressing each of these separately, the Model can more accurately represent how an intervention will be implemented, and the background care into which the intervention will be introduced and against which it will be compared. This approach also enables the user to customize the Model to different settings and to analyze process improvement projects.

Objectives and Limitations of Calibration

Objectives

The objective of calibration is to create a model that represents care as it is actually delivered in the setting of interest. For Simulator 2.3 and ARCHeS, the reference setting is current care in the United States. Representing actual or current care involves three main steps. The first is to develop a systematic representation of care processes in a mathematical form. The method should be sufficiently flexible and general to enable representation of current guidelines at a realistic level of complexity and detail. The method should also include parameters that can be set to modify a care process to match the care that is actually being delivered in a particular setting. The second step is to identify the guidelines that are recommended by professional groups as the standard of care. We will call this “nominal” or "ideal" care and use it as the starting point for representing actual care. However, it is well known that not all providers follow guidelines precisely, and not all patients follow the recommendations of their

[4] For another example, the effect of a guideline that patients with diabetes should have their LDL-cholesterol levels controlled to 100 will be entirely different in a setting where with current practices the mean LDL level is 140, than in a setting where the current practice is delivering a mean LDL level of 110. Whenever a clinical trial uses "current care" as the control for a drug, the "effect" of the drug will be determined as much by what constitutes current care as by the drug itself.

[5] For example, imagine a state transition model that obtained its estimate of the probability of making a transition from "uncomplicated diabetes" to "diabetes with retinopathy" from the United Kingdom Prospective Diabetes Study (UKPDS). That transition probability would reflect the type of care that was being delivered in the United Kingdom in the 1980s and 90s, and the results of using the model would be correspondingly inaccurate if applied to, say, the management of patients with diabetes in Los Angeles today. Indeed, care processes are at the heart of the discrepancies between "efficacy" (what happens in a clinical trial) and "effectiveness" (what happens when the same intervention is applied in the real world).

© 2012

providers. Thus a third step is needed in which the parameters in the care processes are tuned or calibrated to represent how providers and patients behave with respect to following recommended guidelines. It is the combination of the recommended care and the patient and provider behaviors that determines what actually happens — what we will call "actual" or "current" care.

Limitations

The ability to calibrate a model to current care is subject to a number of limitations. First, practices are continually changing to incorporate new technologies, new science, new evidence, and modifications of guidelines. At any time, the Model is calibrated to the most current available data, but the calibration is subject to change to keep the Model current.

A second limitation is that while the objective is to represent current care as it is practiced in the US, in reality practice patterns vary widely across different geographic regions, medical centers, physician groups, and providers. Practices also vary with socioeconomic status, insurance coverage, urban versus rural settings, and other non-medical factors. There is no such thing as a single practice pattern that applies to all settings in the US. Thus, current care as incorporated in the Archimedes Model is suitable for national policies and can be used as the starting point for delivery systems that, on average, follow national patterns. Because the Archimedes Model explicitly represents care processes and behaviors, it can be modified or customized to different settings as needed, provided sufficient data are available to perform the calibrations.

Guidelines also change over time. Because of this, in reality a person can be subject to a variety of different guidelines as he or she ages. Because it is impossible to know for each patient the spectrum and timing of guidelines to which the patient was subject, it is necessary to make simplifying assumptions. For calibrating the care processes in Simulator 2.3, we assumed that simulated patients were subject to current guidelines and healthcare utilization rates for their entire lives. For example, for a simulated 70-year-old man who was diagnosed with dyslipidemia at age 30, we will assume that he has been taking a statin for 40 years, whereas in reality statins have only been on the market since 1987.

A fourth set of limitations relate to the available data. Because there is no single data source that defines all important aspects of care, calibration requires the use of several different sources, which can involve different populations, methods, definitions, time periods, and other factors. The data do not always map perfectly to outcomes and events calculated by the Model, or even to other data sources. Furthermore, for many conditions, the outcomes and events of interest are relatively infrequent, raising issues about statistical variability and wide ranges of uncertainty. Thus for many of the measures used to calibrate a model, there is no single unambiguous number to serve as the target. Rather, it is necessary to review several sources, make judgments about the applicability of each source, and define targets that, in the judgment of appropriately chosen experts, best represent current US care.

A fourth limitation derives from the fact that the starting point for calibration is the set of guidelines published by national organizations. Branch points in the guidelines, where providers and/or patients may take different actions, are identified. Probabilities are assigned to represent the proportions of providers and patients who choose various branches. Examples are the probability that a physician

© 2012

applies a guideline at all, the probability that the physician orders a recommended test, and the probability that a patient follows a provider's recommendation. As will be described below, parameters such as these are "tuned" to fit the observed levels of biomarkers and utilization rates in the population. The issue is that for some practices it is not possible to find any set of parameter values that fit all the available data. This is caused by the fact that some providers do not start with the national guidelines at all, but rather follow other practices which are rarely described and virtually never measured.

For all these reasons and others, it is not possible to find a set of parameters for care processes that cause the Model to fit all the data from all the sources, and there will always be some discrepancies that for all intents and purposes are unavoidable. The objectives of calibration are to get as close as possible to observed levels of care, and to identify places where potentially important discrepancies exist. Calibration is not a test of the accuracy or validity of the model. If calibration tests anything, it tests the extent to which actual practices are consistent with nationally recommended guidelines.

Overview of Care Processes

Initiation of Care Processes

In a simulation using the Archimedes Model, care processes are initiated when a patient encounters the healthcare system. There are two main categories of care processes based on whether or not the encounter is caused by an acute or non-acute condition. Encounters can be initiated when a patient develops a symptom of a disease. In the Model, simulated patients have behaviors that recognize the occurrence of a symptom, determine whether they will seek care at that time or wait until the symptom progresses, and determine where they will seek care. Depending on the urgency of the symptom and the patient's behavior, a patient may seek care acutely in an emergency room or non-acutely in an outpatient setting. Acute care processes triggered by emergency room presentations can lead to ambulatory or inpatient testing and treatment. Each of these behaviors has parameters that can be modified to match observed behaviors.

While some encounters are initiated by the patient, encounters can also be initiated by healthcare providers. This can occur if the person meets criteria for some outreach or follow-up protocol, or it can occur during the course of managing a patient, where an evaluation done as part of one care process reveals a test result or diagnosis that in turn triggers another care process. Thus once initiated, care processes can occur in cascades depending on what information is revealed, how patients respond to treatments, and what events unfold.

For the calibration process, a screening visit is scheduled for all patients at the age of twenty. At this visit they are screened for a number of conditions (following guideline recommendations). Like any other visit, this visit can lead to a cascade of subsequent care processes, or a healthy patient may not return for years.

© 2012

Representation of Care Processes

Care processes vary widely in their complexity. To enable representation and parameterization of the full range of care processes issued by national organizations, the Archimedes Modeling framework includes methods for breaking care processes into components which can then be used as building blocks to represent care processes at high levels of detail and complexity. Here we will describe the components of the non-acute care processes. Processes for the evaluation, diagnosis, treatment, and management of all modeled non-acute care processes are modeled using this framework.

There are two main phases: the first involves evaluation and diagnosis. The second involves treatment and monitoring.

Non-acute evaluation and diagnosis

The evaluation/diagnosis phase has four steps:

1. Candidacy. Determine if a patient meets the inclusion and exclusion criteria for the care process. Eligibility can be based on a wide variety of factors such as demographic information, symptoms, risk factors, or past medical history. Some care processes involve multiple sets of inclusion and exclusion criteria, such as “”low-,” “medium-,” and “high-” risk groups. If so, then in this step a patient will be placed in the appropriate group.
2. Eligibility. If a patient meets criteria for a care process or a particular group in a care process, the next step is to determine if the patient is eligible or indicated for any tests. This takes into account not only the list of indicated tests but any rules for the timing of tests. For example, a care process may call for a person on cholesterol medication to be tested every 6–12 months, in which case this step will search the records to determine the time of the last test and determine if 6 months has passed.
3. Testing. Based on the results of the previous step, perform any tests indicated by the care process.
4. Diagnosis. Finally, make a diagnosis based on the results of the tests and other information. The diagnosis can indicate one or more conditions, or no condition.

Non-acute treatment and monitoring

After a diagnosis is made, the second phase of the care process – treatment and monitoring – begins. This has five steps.

1. Candidacy. Determine that the patient meets the inclusion and exclusion criteria for the treatment. In this phase the criteria will include the results of tests and diagnoses made in the evaluation/diagnosis phase, along with other relevant information.
2. Eligibility. Determine the treatments for which the patient is eligible and check any conditions on the timing of treatments. For example, newly diagnosed patients should receive treatment immediately, whereas the interval of follow-up care varies for each guideline and treatment.
3. Set goal. Determine whether any treatment goal applies, such as "if a patient has a history of myocardial infarction, LDL treatment should be given with the goal of producing the LDL to <100 mg/dL."

© 2012

4. Treatment. Order any treatment(s) specified by the guideline. Examples are writing a prescription and scheduling a procedure. Depending on the treatment, this step could trigger additional care processes such as those relating to the performance of a procedure. If there is a treatment goal in the guideline, then the treatment will be titrated depending on whether the patient has reached the goal.
5. Monitoring. After a treatment is given, arrange for any follow-up visits and tests that are recommended in the care process. These protocols frequently include recommendations for timing such as, "after a patient is placed on cholesterol-lowering treatment, retest the cholesterol in 4 to 6 months." Monitoring can also include referrals to specialists. The monitoring step is usually dynamic, with specific recommendations depending on the patient's response to treatment, the results of follow-up tests, and so forth.

The non-acute components as well as the potential for a cascade of care processes are illustrated in Figure 1.

Acute care processes and prioritization of care

In the Archimedes Model acute care processes are also broken down into components with the objective of being able to prioritize the diagnosis and treatment of conditions based on the urgency of the condition. For example, if a patient seeks care in an emergency department for chest pain, an EKG finds signs of a myocardial infarction with ST elevation (STEMI), and a blood pressure test finds the patient has hypertension, and then the care process for managing STEMI will take precedence over the care process for evaluating hypertension. Prioritization of the care processes is based on standard clinical practices as described in the guidelines themselves, supplemented by the judgments of clinicians and experts.

The components of acute care processes include two phases: testing and treatment. When a patient presents with one or more symptoms, the relevant testing processes are exercised in a way that will allow the most urgent condition to be diagnosed first. Once a diagnosis is made, treatment of that condition is initiated. When multiple conditions are diagnosed, treatment of those conditions is also ordered such that the most urgent condition is treated first.

Multiple care processes

Any of these components can trigger additional care processes. For example, if the evaluation component in one care process calls for certain tests to be performed, then depending on the results of those tests the patient may become eligible for another care process, in addition to the care process that is in progress. Or, depending on how a patient responds to a treatment, additional evaluations and treatments may become indicated. The components can be sequenced, repeated, and branched to any extent necessary to represent the full complexity of the progression of diseases and the application of care processes to manage them.

© 2012

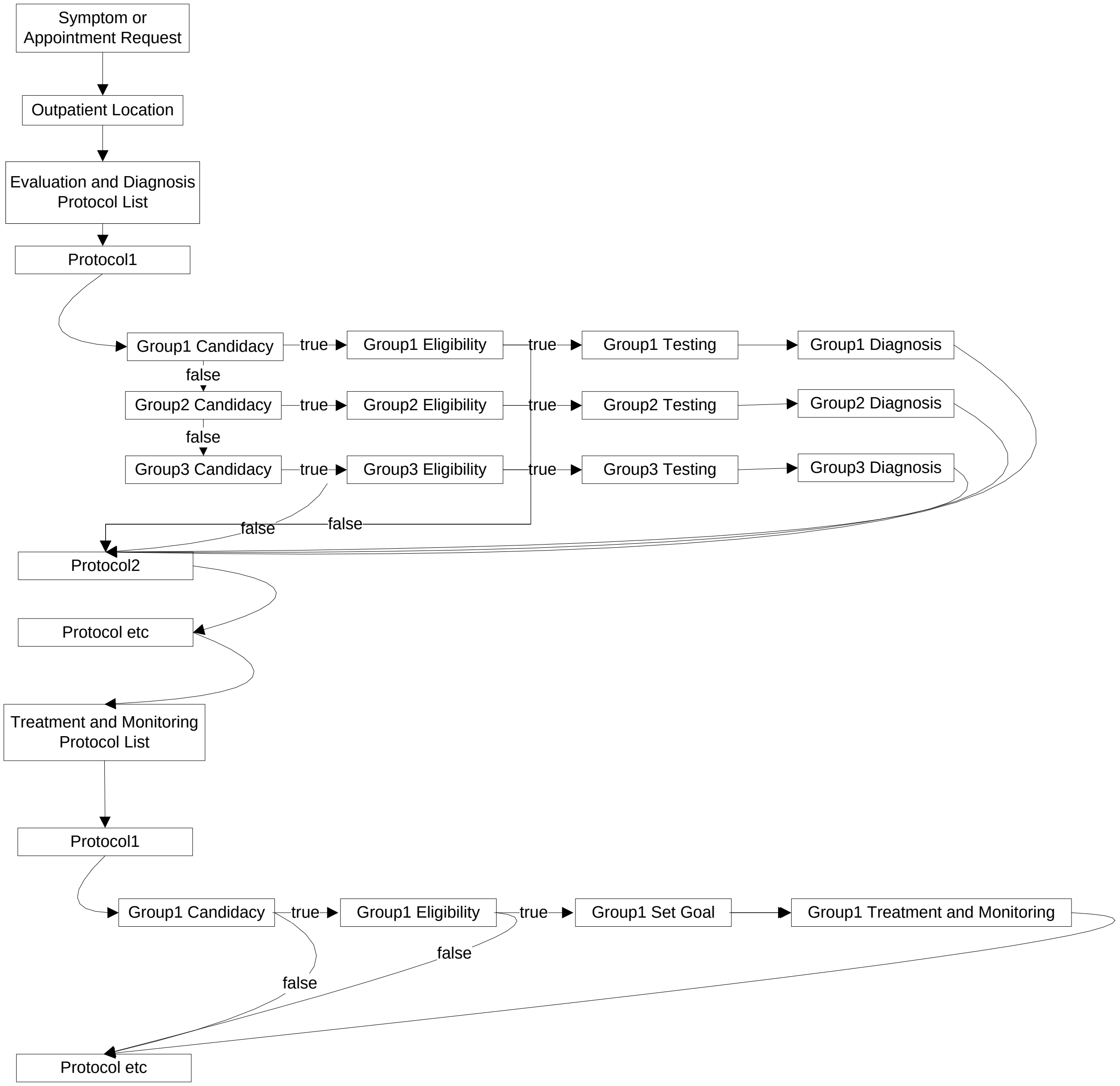

Figure 1. Illustration of Flow through Non-Acute Protocols.

Scheduling of care processes

Because a patient may be subject to multiple care processes, each of which has monitoring and follow-up visits, the methods for representing care processes include time windows for visits as well as algorithms for coordinating visits and combining tests and treatments that occur at visits, as occurs in reality.

Resolution of ambiguities in guidelines

In our experience it is possible to represent virtually all existing guidelines using these building blocks. Ambiguities in existing guidelines such as "[Test A] *may be considered* in some cases" are resolved when possible using data on utilization, or by expert judgment and observation of common clinical practice

when utilization data are not available. Ambiguities in existing guidelines and lack of sufficient data to resolve ambiguities are important limitations of the calibration process.

Parameterization of Care Processes

Construction of care processes is only the first step in the calibration of the Model to match current practice patterns. The care processes built using the methods just described represent what we are calling "ideal care" – the care that is specified by the national organizations. This level of care is not realistic in the sense that not all providers and patients behave as called for in the care process. Discrepancies between the "ideal care" specified by the guideline, versus the "actual care" or “current care” that is actually practiced, are addressed by calibration.

To enable calibration of the care processes from the ideal to the real practices, each step in the care process includes two sets of parameters. One set specifies the probabilities that the provider will execute that step in the care process as specified – e.g., correctly determine a patient's eligibility, conduct the evaluation as specified, order recommended tests, make a diagnosis accurately, give a treatment as recommended, schedule follow-up visits as recommended, and so forth. The other set of parameters specifies the probabilities that the patient will adhere to the actions recommended by the provider – e.g., take the test, take the treatment, show up for the follow-up visits, see the specialist, and so forth. The parameters can be simple probabilities, or can be calculated as functions of other variables in the model such as a patient's age, risk factors, and so forth. It is these parameters or equations for parameters that are tuned to calibrate the “ideal” care processes to represent observed behaviors and levels of utilization.

Calibration Parameters

Because the structure for the care processes is general and all care processes are built using the same building blocks, it is possible to specify a set of parameters that can be used to calibrate any care process. By setting the parameters to different values, the Model can be calibrated or customized to a wide variety of different settings, and to different patterns of practice within any particular setting (e.g., conservative, average, aggressive practice). The parameters are divided into three main categories: patient response to symptoms, provider performance, and patient adherence. They are described in Table 1.

© 2012

Table 1. Healthcare Process Calibration Parameters.

Parameter Number	Description
1	Probability that the person seeks care for a symptom.
2	Probability that the person presents to the emergency room when symptomatic.
3	Probability that the person presents to a specialist for care when symptomatic.
4	Probability that an individual does not receive a care process for which he or she is eligible.
5	Probability that a patient with a disease is not diagnosed with that disease.
6	Probability that a condition that is controlled is incorrectly determined to be uncontrolled (possibly causing intensification of treatment).
7	Probability that a condition that is not controlled is incorrectly determined to be controlled (possibly leading to an inappropriate withholding of treatment).
8	Probability that a patient has a prescribed test.
9	Probability that a patient takes a prescribed treatment.
10	Probability that a patient attends a scheduled appointment.
11	A window of time in which a follow-up visit can occur.

When a simulated individual "perceives" a symptom, a part of the Model that addresses patient behavior determines how the person responds to the symptom. The probability that the patient seeks care is determined by a parameter (parameter #1). If the patient decides not to seek care, he or she does not enter the healthcare system. If the patient does seek care, there are two parameters that determine where the patient will seek care, at an emergency department (parameter #2), from a specialist (probability #3), or from his or her primary care physician. For example, if parameter # 1 is 0.2 and parameter #2 is 0.5 and parameter #3 is 0.25, then 20% of individuals who perceive symptoms will ignore them. Of the remaining 80% that seek care for their symptom 50% will present to the emergency department (40% of the total group). Of the remaining 40%, 25% will present to a specialist (10% of the total group). The other 30% of those who perceive symptoms will present to their primary care physician. This level of detail is important because it is an important determinant of the cost of care.

In the non-acute setting, the performance of providers is affected by four parameters (parameters #4 though #7). Parameters #4 and #5 affect the flow of patients through the evaluation phase of care, while the other two affect monitoring.

The acute care setting does not include parameters associated with provider performance. In this setting providers are assumed to follow recommended care processes with 100% performance. This aspect of the care-process model can be made more realistic if needed.

In addition to parameters that affect the performance of providers, the Model includes parameters that affect the probabilities that patients will adhere to particular steps in the care processes (parameters #8

© 2012

through #10). The patient adherence parameters apply to specific tests, treatments, and appointment requests and can be used in both the non-acute and acute settings. The methods for setting these parameters are very general; adherence to a specific test, treatment, or encounter can be a simple probability, or it can be a more complex function of such factors as the patient's history, co-morbidities, past adherence, and other measures of health and behavior.

The final parameter (parameter # 11) affects the scheduling of care processes. This parameter defines a window of time in which a follow-up visit can occur. This is used by the Model to coordinate visits.

Figure 2 illustrates how the calibration parameters are integrated into the non-acute components.

Sources for Guidelines

Because the reference version of Archimedes Simulator 2.3 is designed to represent current care as delivered in the US, the care processes are based on US national guidelines, and are calibrated using data from the US national datasets. The guidelines currently incorporated in Simulator 2.3 are shown in Table 2.

Table 2. List of Guidelines and Protocols Included in Archimedes Simulator 2.3.

Protocol	Guideline Source
Hypertension	JNC-7/ADA: Standards of Medical Care in Diabetes – 2008
Dyslipidemia	ATP-III
Diabetes mellitus (DM)	ADA: Standards of Medical Care in Diabetes
Nephropathy	ADA: Standards of Medical Care in Diabetes
Stroke	American Stroke Association/AHA guideline
Myocardial Infarction with ST elevation (STEMI)	ACC-AHA STEMI
Unstable angina or MI without ST elevation (UA/NSTEMI)	ACC-AHA UA & non-STEMI guidelines
Chest pain	ACC-AHA UA & non-STEMI guidelines
Possible acute coronary syndrome (ACS)	ACC-AHA UA & non-STEMI guidelines
Revascularization	ACC-AHA Stable Angina
Retinopathy	ADA: Standards of Medical Care in Diabetes
Coronary artery disease (CAD)	ACC/AHA guideline
Neuropathy and diabetic foot ulcer (DFU)	ADA: Standards of Medical Care in Diabetes and ADA: Management of neuropathy
End-stage renal disease (ESRD) symptoms (nephropathy)	National Kidney Foundation KDOQI
Amputations (neuropathy)	IDSA guidelines for the diagnosis and treatment of diabetic foot infections

© 2012

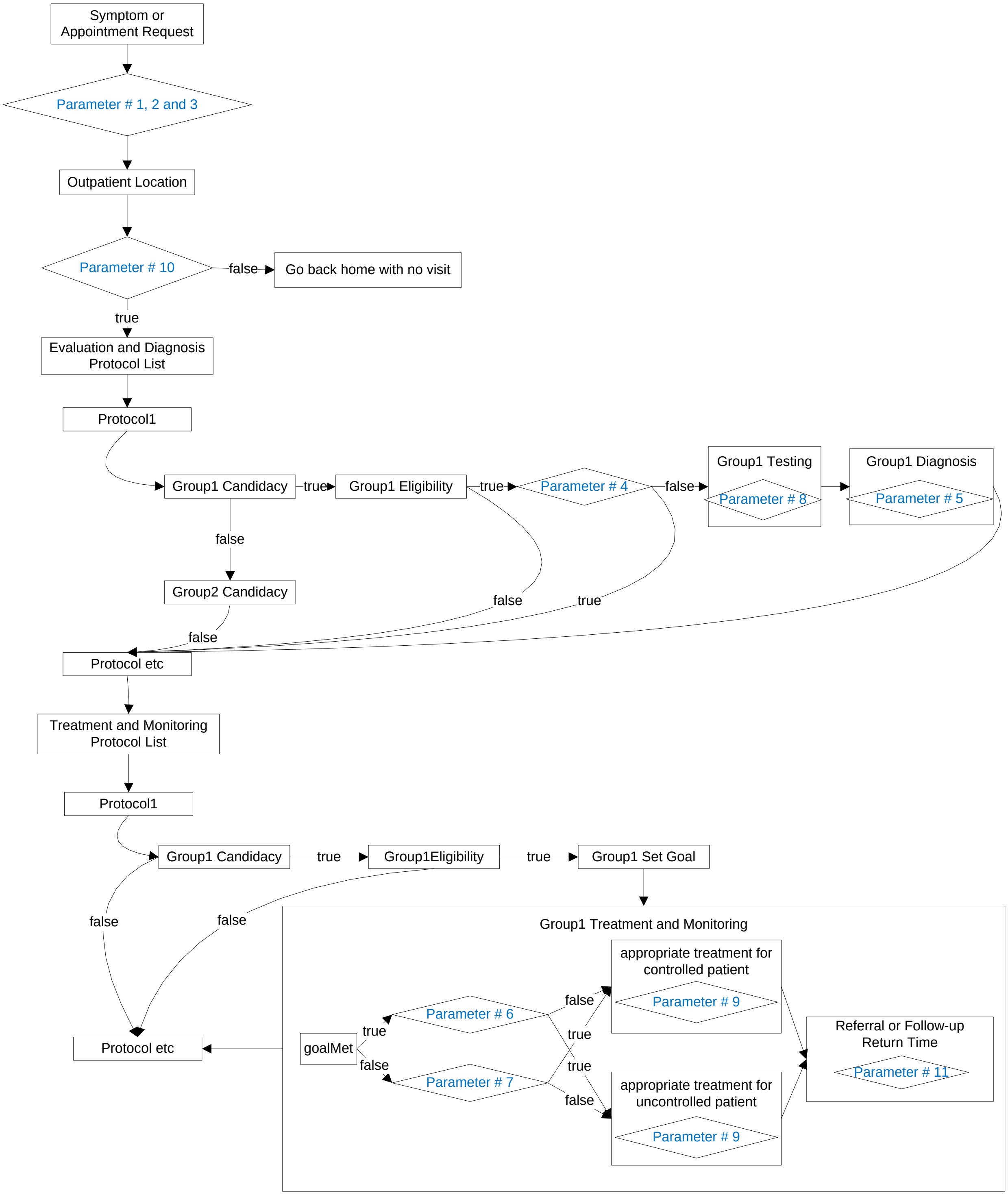

Figure 2. Parameterization of Non-Acute Care Processes.

Sources for Calibration Data

Approximately thirty national-level datasets were surveyed to identify measures and target values for the calibration process. Five main datasets were chosen because they provided data that were nationally representative, spanned the diseases and care processes in the Archimedes Model, and included information on subpopulations. They are listed in Table 3. Additional sources were used when needed to calibrate aspects of the Model that were not adequately covered by these sources.

Table 3. Main Sources for Calibration Data.

Dataset	Year	Survey Content	Survey Sample Design	Sample Size
NHANES National Health And Nutrition Examination Survey	1999-2006	Chronic disease prevalence and conditions (including undiagnosed conditions), risk factors, diet and nutritional status, immunization status, infectious disease prevalence, health insurance, and measures of environmental exposures. Other topics addressed include hearing, vision, mental health, anemia, diabetes, cardiovascular disease, osteoporosis, obesity, oral health, mental health, and physical fitness.	Uses a stratified multistage probability sample, nationally representative of the US civilian non-institutionalized population.	Approximately 5,000 people are examined each year.
NAMCS National Ambulatory Medical Care Survey	2006	Information is obtained on various aspects of office visits, including physician practice characteristics, patient characteristics, and other visit characteristics. Among the items collected are patient's age, gender, race, and ethnicity; patient's expressed reason for visiting the physician; intentionality of injury, if any; physician's diagnoses; diagnostic services ordered or provided; therapeutic services; ambulatory surgical procedures performed; medications; providers seen; visit disposition; time spent with physician; and expected source of payment.	National probability sample survey of visits to office-based physicians in the United States.	In 2004, 25,286 survey forms (each representing one physician-patient visit) were collected.

NHAMCS National Hospital Ambulatory Care Survey	2006	The NHAMCS includes two files: emergency department (ED) visits and outpatient department (OPD) visits. Information is obtained on various aspects of patient visits, including patient, hospital, and visit characteristics. Among the items collected are patient's age, gender, race, and ethnicity; patient's expressed reason for visit; intentionality of injury, if any; physician's diagnoses; diagnostic services ordered or provided; procedures provided; medications; providers seen; visit disposition; immediacy with which patient should be seen; and expected source of payment. Items collected that are specific to the ED include mode of arrival, waiting time, duration of time in the ED, initial vital signs, and cause of injury.	National probability sample survey of visits to emergency departments (EDs) and outpatient departments (OPDs) of non-federal, short-stay, and general hospitals in the United States.	About 400 EDs and 225 OPDs participate each year. In 2004, 36,589 ED forms and 31,783 OPD forms were completed.
NHDS National Hospital Discharge Survey	2006	Variables collected include age; gender; race; ethnicity; admission and discharge dates (length of stay); discharge status; source of payment; hospital size, ownership, and region; from one to seven diagnoses coded using the ICD-9-CM; and, from zero to four procedures using the ICD-9-CM.	Utilizes a three-stage national probability design that includes primary sampling units (PSUs), hospitals within the PSUs, and discharges within the hospitals.	Approximately 300,000 discharges are sampled each year from about 500 hospitals.

CMF Compressed Mortality File	2006	The number of deaths, crude death rates or age-adjusted death rates can be obtained by place of residence (total US, census region, census division, state, and county), age group, race (years 1979-1998: White, Black, and Other; years 1999-present: American Indian or Alaska Native, Asian or Pacific Islander, Black or African American, and White), Hispanic origin (years 1979-1998: not available; years 1999-present: Hispanic or Latino, not Hispanic or Latino, not stated), gender, year of death, and underlying cause of death (years 1979-1998: 4-digit ICD-9 code and 72 cause-of-death recode; years 1999-present: 4-digit ICD-10 codes and 113 cause-of-death recode), and urbanization level of residence (2006 NCHS urban-rural classification scheme for counties).	Mortality information is collected by state registries and provided to the National Vital Statistics System. Underlying cause of death and demographic descriptors are indicated on the death certificates.	In 2006, among adults aged 20-84, 1,671,006 deaths were recorded in a population of 212,022,561.

Calibration Measures

Using the sources in Table 3, and considering the level of detail available in these sources and in the Archimedes Model, we identified the following types of measures as appropriate targets for the calibration process (Table 4).

Table 4. Types of Measures Used for the Calibration Process.

Measure	Description
Mean biomarker values	Mean of biomarkers compared to estimates from NHANES.
Prevalence demographics	Prevalence of gender, race, and ethnicity demographics compared to estimates from NHANES.
Prevalence of diagnosed conditions	Prevalence of diagnosed conditions at specified time and compared to estimates from NHANES and NAMCS/NHAMCS - OPD.
Incidence of diagnosed conditions	Incidence of diagnosed conditions over specified interval, then normalized to annual rate and compared to estimates from combined data from the NHDS and NHAMCS. Incidence is defined prospective per capita (number of diagnoses per capita in one year).

© 2012

Incidence of diagnosed deaths[6]	Incidence of deaths due to different causes over specified interval then normalized to annual incidence and compared to estimates from CMF. Incidence is defined prospective per capita (number of diagnoses per capita in one year).
Hospitalizations per capita	Number of inpatient admissions prospective per capita over specified interval, then normalized to annual rate and compared to estimates from NHDS (only admissions for conditions in the Model).
Outpatient visits per capita	Number of outpatient visits prospective per capita over specified interval, then normalized to annual rate and compared to estimates from NAMCS/NHAMCS - OPD.
Tests per capita	Number of tests performed prospective per capita over specified interval, then normalized to annual rate and compared to estimates from NAMCS and NHAMCS (both OPD and ED).
Treatments per capita	Number of treatments prospective per capita over specified interval, then normalized to annual rate and compared to estimates from combined data from the NHDS and NAMCS/NHAMCS - OPD.
Prevalence taking intervention	Prevalence of patients taking an intervention at specified time compared to estimates from NHANES.

Subpopulations

The calibration process involves not only calibrating care processes to match data for the entire population but also calibrating the Model for important subpopulations. Subject to the availability of data, for each of the measures in Table 4, we defined targets for the following subpopulations (ages and co-morbidities at baseline):

- All adults (age 20 to 85)
- Age 20 to 45
- Age 45 to 65
- Age 65 to 85
- Male
- Female
- Diabetic
- CAD

[6] A diagnosed death is a death attributed to a particular cause, such as “death from myocardial infarction.” The Model also calculates "deaths from other causes" and "all-cause mortality."

© 2012

Target Values for Calibration Measures

General Methods

To the greatest extent permitted by the available data, a target value was specified for each measure, and the care processes in the Model were calibrated to match those values. For some measures there were multiple data sources for specifying target values, in which case judgments had to be made about a "primary" target and upper and lower bounds. This section describes some of the methodological issues that arise in specifying target values and bounds.

The methodology for estimating targets requires mapping simulated outcomes to real-world diagnosis-related groups (DRGs), ICD codes, or CPT codes. Mappings were established by a committee of physiology modelers and internal medical staff. All primary datasets include weights for the individuals that enable generalization of the dataset to a nationally representative population. Subpopulation sample size estimates derived from 2005-2006 NHANES data using the interview weight[7] were used as normalization factors across the various source datasets. These steps ensured that the measures were as uniform as possible.

For many of the measures used to calibrate care processes, target values had to be obtained by adding rates obtained from multiple data sources. Setting a target for the incidence of myocardial infarctions is one example. MIs seen in hospital emergency departments and discharged without admission are recorded in NHACS. MIs seen in ambulatory care settings are recorded NACS, and MIs admitted are recorded in NHDS. Silent MIs and sudden deaths may not be recorded in any of these. Furthermore an event might be recorded in more than one dataset; a MI that occurred in an ambulatory setting but was later admitted might be recorded in both ambulatory and hospital datasets. Because of gaps and overlaps like these the data reported in each dataset had to be analyzed carefully to derive the most accurate possible estimate of the target value. In some cases the best that could be accomplished was to identify values that represented upper or lower bounds for the "true" value.

Another problem was that for some measures, such as the use of tests, a value reported in the dataset might be too inclusive. For example, a particular test might be used for a wide range of conditions, not all of which are currently in the Archimedes Model. In such cases the observed rate of test usage would be expected to overestimate the rate calculated by the Model. To the extent permitted by the available data, observed rates were adjusted to reflect the diseases in the Model.

Calculation of Target Values Used for Calibration

The following sources used to identify target values for each type of measure used for the calibration.

Target values for demographic information and biomarkers

Targets for mean biomarker values and prevalence of demographic factors were estimated from NHANES data. Comparisons of NHANES data collected from 1999-2006 and NHANES data collected from

[7] NHANES provides a number of weighting factors. In this case, interview weight, WTINT2YR, is appropriate.

© 2012

2005-2006, with and without imputation of missing values, revealed that all four approaches yielded consistent mean values for biomarkers and demographic information. Therefore, to determine the target values, we used the approach deemed to be the most complete, which was NHANES data from 1999-2006, with imputation of missing values.

Target values for prevalence of diagnosed conditions

Prevalence rates of diagnosed conditions were estimated primarily from NHANES and NAMCS/NHAMCS-OPD data. Neither NHANES nor NAMCS/NHAMCS-OPD has chronic disease markers that cover all the diseases that are in the Archimedes Model. NHANES was used because it has the best coverage. Another complication with the NAMCS/NHAMCS-OPD data was that the three diagnosis fields were related to visits and did not include all the diagnoses a person may have. Therefore NAMCS/NHAMCS-OPD data represented lower bounds for the target values. The prevalence rates of obesity and dyslipidemia are much higher in NHANES than in NAMCS/NHAMCS-OPD and probably too high for diagnosed conditions; however, NHANES was still used for these targets to maintain consistency.

Target values for incidence of diagnosed conditions (other than death)

No single data source reported all the possible ways the first occurrence of a condition might be recorded for estimating incidence rates. For example ambulatory-care surveys record cases that are seen in ambulatory care, while hospital-based surveys record cases first seen in that setting. To derive target values for incidence rates of diagnosed conditions, for most conditions it was necessary to add rates reported in NAMCS/NHAMCS-OPD and NHAMCS-ED. Exceptions were myocardial infarction (MI) and stroke. For these conditions it was necessary to also include incidence rates reported in NHDS. The NHDS dataset does not have sufficient markers of chronic condition to enable estimation of incidence for subpopulations based on disease histories; it provided information only for subpopulations based on gender and age.

When using data from NAMCS and NHAMCS, it was important to distinguish between visits for “new” conditions and existing conditions. Only the individual’s first diagnosis and the reason for the visit were considered. The "high," "best," and "low" estimates for incidence rates were based on the recorded reason for the visit.

Target values for diagnosed deaths

Incidence rates of diagnosed deaths were derived from CDC mortality data. As for other diagnoses, deaths that occur in the simulation had to be mapped to ICD codes (in this case ICD-113 group codes and ICD-10 codes). There is some uncertainty in this approach because definitions of conditions by ICD code are not consistent. For example the definition of CHD death can vary greatly across different studies and sources, if a definition is provided at all. Because of this, it was not always clear how conditions calculated in the Model should be mapped to ICD codes. Where there was ambiguity about whether a particular ICD code was calculated by the Model, we conducted a sensitivity analysis in which we designated the code as either “modeled,” “possibly modeled,” “probably not modeled,” or “not modeled.” This method was used to define a lower bound, best estimate, and upper bound for the target value for the outcome, as follows:

© 2012

- Lower bound: Count only persons who had a “modeled” ICD code
- Best estimate: Count all persons who had a “modeled” ICD code, and half of those with a “possibly modeled” ICD code
- Upper bound: Count all persons with “modeled,” “possibly modeled,” or “probably not modeled” ICD codes.

Target values for incidence of treatments/procedures

Incidence rates for treatments (procedures) were calculated as the sum of rates reported in the NAMCS/NHAMCS-OPD and NHDS datasets. When using the NAMCS/NHAMCS-OPD data, diagnoses associated with a visit were checked to confirm that the procedure of interest occurred. These data have several types of procedure codes spanning eight potential fields. Each of these eight procedure fields was checked. In the NHDS dataset the fields that describe procedures are not as complex but had to be checked to confirm the DRG codes associated with each visit. Because the NHDS dataset does not have fields that describe chronic conditions, it was not possible to estimate incidence rates for subpopulations that had chronic conditions.

Target values for prevalence of individuals receiving interventions

Prevalence rates of persons taking various treatments were calculated from NHANES. The primary limitation of using NHANES for this measure is that in this dataset information on medication usage is acquired by questionnaire. This methodology often overestimates true rates. For an extreme example, it has been observed that 87% of persons with diabetes report that they adhere to diabetes lifestyle changes, which is a much higher rate than reported the literature using more reliable methods.

Analysis of Measures Not Used for Calibration

Target values for hospitalizations

Data on hospitalization rates were obtained from NHDS. As described above, this dataset does not have sufficient markers of chronic conditions to enable estimation of hospitalizations for subpopulations based on disease histories; data are available only by gender and age. In the real world, admissions are grouped into hospitalization categories (DRGs), and these are used for estimating costs. To calibrate the care processes in the Archimedes Model, the data were queried in such a way as to mimic the DRG methodology.

The first step was to create a set of “Archimedes DRG” codes that map to “real-world DRG” codes. This was necessary because real-world DRGs are based on both diagnoses and procedures. While the DRG reported in NHDS may not be included in the Model, a component of the hospitalization may be included in the Model. For example, a patient could present with an MI but receive a heart transplant during the admission. The real-world DRG recorded would be for the heart transplant. The Archimedes Model does not include transplants, but it is important to include the admission for the MI in the data analysis. Therefore in this example, the real-world DRG for the heart transplant should be mapped to an Archimedes DRG for the MI. For these reasons we analyze hospitalization data at a finer level of detail than is captured by the real-world DRG classification.

© 2012

These DRG, ICD-9, and procedure code mappings enable us to query the NHDS dataset to determine all possible Archimedes-DRG categories in which a hospitalization could be classified. Archimedes-DRG outcomes are ranked by cost using Medicare data, and the most expensive Archimedes DRG is assigned to each hospitalization. This technique mimics how hospitalizations are classified into real-world DRGs, by cost.

Even after making these adjustments, it was apparent that target values estimated from the datasets did not always align with simulated points. In some cases the Model includes the “chronic” aspects of a disease but does not include acute exacerbations that could bring an individual to the hospital (e.g. atrial fibrillation). Most of the missed costs are corrected (e.g. an average cost of having atrial fibrillation is applied to life years spent with this chronic condition), but the true rate of hospitalizations was still low. Furthermore, hospitalizations for some conditions are relatively infrequent. For all these reasons we determined that the range of uncertainty and potential for errors were too high to justify using hospitalizations rates to calibrate the care processes. We calculate them but only use them for general insights into the model and the data, and therefore do not report the results here.

Target values for outpatient visits

Incidence rates of outpatient visits were calculated from NAMCS/NHAMCS-OPD datasets. Use of these datasets was complicated by the need to define which visits reported in the datasets are relevant to the diseases in the Model. This was addressed by reviewing the full set of ICD-9 codes covered by the Model and finding visits that include these codes as the primary diagnosis. All of the reasons for visits given in the dataset were reviewed, and visits for irrelevant reasons (such as visits for social-problem counseling, injuries, or administrative purposes like school exams or driving tests) were excluded. A visit was included in data analysis if a relevant diagnosis was made or if the reason for the visit is covered by the Model.

However, despite taking these steps, it was determined that the potential mismatches between visits calculated by the model and real-world visits recorded by ICD-nine codes were too great to justify reliance on these data for calibrating the Model. Therefore, as with hospitalizations, these results were calculated and examined for general insights, but are not reported here.

Target values for tests

Incidence rates for tests were estimated from NAMCS and NHAMCS (both outpatient and emergency department (ED)) datasets, which cover ambulatory care. Ideally the target values for rates of test use would also include tests performed on an inpatient basis. However, NHDS does not have information on tests. Furthermore, while the ED component of NHAMCS contains test information, it does not capture repeat tests or tests taken if someone is admitted to the hospital for several days. For these reasons, estimates based on these datasets almost certainly underestimate the actual rates of test use.

An additional problem is that use of tests calculated in the Model does not always align perfectly with the use of tests reported in the datasets, or with the reasons tests are given in the real world. For example, the NAMCS and NHAMCS-OPD datasets do not have specific fields for FPG tests, but instead have a general data field for "glucose tests." In contrast, the Archimedes Model includes specific fields

© 2012

for FPG, RPG, OGTT, and HbA1c tests. In the case of blood pressure tests, in the real world, blood pressure is measured almost every time a person makes contact with the healthcare system, but in many instances the provider does not act on an abnormal finding. In the Model, each test is taken with the purpose of making a diagnosis, and providers act on abnormal findings with 100% performance. This difference in performance rates either causes prevalence rates of conditions in the Model to be greater than observed prevalence rates (if testing is done at the same rate in both the Model and the real world), or causes rates of tests to be lower in the Model than in the real world (if testing rates are decreased to create alignment between prevalence rates in the Model and prevalence rates in the real world). The Model can be calibrated to either the rates of tests or the prevalence of conditions diagnosed by the tests, but not both.

For these and other reasons, we determined that rates of test usage were too uncertain and unreliable to justify using calibration. Results were calculated and examined for general insights, but are not reported here.

Example of Estimating a Target Value: Incidence of MIs

The previous section gives an overview of how target values are set for different types of calibration measures. This section illustrates the methods by describing how target values were estimated for the incidence of MI.

When specifying a target value for calibration, the objective is to use real data to determine the true rate of the event or outcome of interest. Occasionally a dataset will measure exactly the event or outcome of interest, and the rate reported in the dataset can be used without modification to set a target value for that measure. Far more commonly, estimation of the true rate requires examination and interpretation of multiple datasets. Myocardial infarctions provide a good example, because people with MIs can receive care in several different settings: ambulatory, hospital ambulatory, hospital admission, etc. Thus to capture all the MIs that occur it is necessary to add the incidences rates found in several different surveys, while trying to avoid double counting. An additional complication is that when using NAMCS and NHAMCS, one has to distinguish between visits for “new” conditions and existing conditions. This issue does not arise when using NHDS because this dataset only includes hospitalizations (inpatient stays), and these are a direct result of the condition diagnosed in that stay.

Use of NAMCS and NHAMCS Datasets

For these two datasets it is important to distinguish “new” from existing conditions when calculating incidence. For example, if the visit results in a final diagnosis of CAD, it should count as a CAD diagnosis and contribute to incidence only if the person had never been diagnosed with CAD previously. While there is no unequivocally correct way to determine this, for the NAMCS and NHAMCS datasets two steps were taken to address this issue. First, only the person’s first diagnosis (three are provided) was used, and second, the reason for the visit was taken into account. To determine the visits that were relevant to a condition, each visit was reviewed to see if the person's first diagnosis (DIAG1) was in the list of codes provided for that disease (see ICD-9 diagnosis codes listed for MIs below). The diagnosis was not

© 2012

counted if it was a second or third diagnosis (DIAG2 or DIAG3) because those are not incident (new) cases.

Specification of Lower and Upper Bounds and Best Estimates for MI Incidence Based on NAMCS and NHAMCS

After finding all visits with diagnoses relevant to MIs, lower and upper bounds and best estimates of incidence were specified based on the reason for the visit. There are variables in the NAMCS and NHAMCS datasets that describe the major reason for a visit (e.g., whether the visit was due to a new problem, a chronic problem, a chronic flare-up, post- /pre-operative, preventive, or none), and variables that describe the actual reasons for the visit (including disease-related codes). This information was used in conjunction with the ICD-9 codes to determine whether a particular visit should be included in the calculation of lower and upper bounds, and best estimates for the incidence rate.

The following ICD-9 codes are used for the diagnosis of MI with ST elevation (STEMI) and MI with no ST elevation (NSTEMI)

- STEMI: 41000, 41001, 41002, 41010, 41011, 41012, 41020, 41021, 41022, 41030, 41031, 41032, 41040, 41041, 41042, 41050, 41051, 41052, 41060, 41061, 41062, 41080, 41081, 41082, 41090, 41091, and 41092
- NSTEMI: 41070, 41071, and 41072

The following rules were used to combine information on the ICD-9 codes and the reasons for visits in order to derive lower-bound, best, and upper-bound estimates for the incidence rate:

- Upper bound: Person must have one of the ICD-9 diagnoses listed above, and the first reason for the visit must not be administrative (e.g., driver's test), adverse effect, or family planning.
- Best estimate: The person must meet all of the specifications for the upper bound calculation and must also have the first reason for visit be either a new problem or blank; here we exclude major reasons for visit that are coded as chronic, chronic flare-up, pre- /post-surgical, or preventive.
- Lower bound: Person must meet all of the specifications required for the best estimate and must also have one of his reasons for the visit be heart examination, EKG, treadmill test, heart catheterization, or review of EKG test results.

Use of NHDS Dataset

The NHDS includes only hospitalizations (inpatient stays), and these are a direct result of the condition diagnosed in that stay. To determine if a hospitalization is for an MI, the person's first diagnosis (DX1) is checked against the same diagnosis codes used when querying NAMCS and NHAMCS. As was done for NAMCS and NHAMCS, only the primary diagnosis code was considered. (NHDS can have seven diagnoses associated with each visit, labeled DX1-DX7).

© 2012

MI Incidence Estimates

Final estimates of MI incidence were calculated as the sum of incidences found in NAMCS, NHAMCS, and NHDS surveys. The incidence calculated with the NHDS dataset was summed with results of analyzing the NAMCS and NHAMCS datasets to calculate final values for lower and upper bounds and best estimates.

Although data from NAMCS, NHAMCS, and NHDS are highly reliable and our methodology was rigorous, additional sources were sought to confirm these estimates. The National Heart, Lung, and Blood Institute's (NHLBI) *Incidence & Prevalence: 2006 Chart Book on Cardiovascular and Lung Diseases* was used to provide additional estimates of MI incidence rates. The data sources are included in Table 5. Because the NHLBI resource includes many studies, Table 5 also provides information on the study and population from which the data were obtained.

Table 5. Data Sources for Estimates of MI Incidence

Outcome	Description of Estimate	Data Source	Annual Incidence	MI per Year*
MI	Low	NAMCS + NHAMCS + NHDS	0.0032	651,377
	Best	NAMCS + NHAMCS + NHDS	0.0034	697,962
	High	NAMCS + NHAMCS + NHDS	0.0036	742,075
	Low	NHLBI Table 4-10, ARIC Surveillance Study including ages 35-74	0.0027	556,556
	Low	NHLBI Table 4-2, ARIC Study including ages 45-84	0.0029	587,476
	High	NHLBI Table 4-26, FHS, both original and offspring cohorts, including ages 35-94	0.0044	896,674
	High	NHLBI Table 4-42, FHS offspring cohort including ages 35-84	0.0051	1,040,966

* MI per year is estimated assuming there are 206,131,855 individuals aged 20 to 85. This was derived from NHANES 2006 using the interview weight WTINT2Y.

Caveats

This example illustrates several of the limitations inherent in using datasets to define target values for calibration. Even with rigorous survey methods and highly detailed data it is not always clear how the observed data should be mapped to the outcomes of a simulation. Using upper and lower bounds helps show how sensitive the data are to unknown factors but it does not resolve all the uncertainties. It is often necessary to consider several datasets when estimating a target value for a measure because no single dataset covers all of the factors completely. When combining data in this way, care must be given

© 2012

to use a consistent set of assumptions and not double-count events. Finally it is necessary to confirm estimates with independent sources whenever possible.

Calibration Methods

Prioritization of Measures

As described above (Table 3), a wide range of measures have been identified for calibrating the care processes in the Model. The calibration process begins with an internal committee of science and medical staff who select the measures, outcomes, and subpopulations of greatest importance, based on new science of evidence, and the intended uses of the Model. Calibration objectives are then set for the chosen measures, outcomes, and subpopulations.

An important consideration when calibrating the care processes in the Model to a particular measure is the population to which the measure most closely applies. In general, matching utilization rates for a subpopulation is more important than matching rates for the total population. For example, the use of diabetic medications should be calibrated to rates observed in the diabetic subpopulation before attempting to match utilization rates in the total population. The objective is to match outcomes in all the relevant subpopulations first, and then to the total population. This will ensure that outcomes in the relevant subpopulations are correct.

Objectives of Measures

The measures are used in three main ways to help calibrate care processes. We use the terms “match,” “observe,” and “review” to describe the different objectives. The distinction is required by the fact that the measures vary in the extent to which they depend on the underlying physiology (i.e., the occurrence of diseases and their symptoms) versus the care processes used to prevent and manage the diseases. For example, the age-specific incidence of type II diabetes is determined by the interaction between two parts of the Archimedes Model. One is the physiology model which calculates glucose physiology and the development of elevated FPG levels. The other is the part of the Model that represents care processes and behaviors, and, in the context of this particular example, determines the use of FPG tests. In a setting in which everyone is screened frequently, the age-specific incidence rates will be shifted to younger ages and will be higher than in settings in which no testing is done. In the latter setting the disease is diagnosed only after a patient seeks care for symptoms, or perhaps not at all. Thus if there is a discrepancy between the diabetes incidence rates calculated by the Model and those observed in reality, the gap could be due to the physiology model, the care processes for FPG testing, or a combination.

The extent to which measures depend on the underlying physiology affects how discrepancies between the Model’s results versus observed results should be interpreted, and what modifications if any should be made to the care processes or physiology model. In general, measures of demographics, biomarkers, diagnosed conditions (prevalence and incidence), and diagnosed deaths are determined primarily by the physiology model. Measures of utilization of procedures, tests, drugs, and other interventions are more

© 2012

affected by care processes. Because of these differences, these two groups or types of measures have different objectives associated with them. The measures that primarily reflect utilization are used to calibrate the care processes and behaviors. Specifically they are the measures used to tune the parameters in Table 1 to achieve as close a fit or "match" as possible. In contrast, the measures that are primarily related to physiology are rarely used to modify any parameters in the care processes. They are simply "observed." If there are discrepancies between the values of these observed measures calculated by the Model compared to the values observed in reality, then that information is most often used to focus attention on the relevant parts of the physiology model to see if any changes may be indicated. That is, they serve as flags to help identify potential improvements in the physiology model, but they are rarely used directly to modify or tune the physiology model.

While calibration efforts focus on those measures that are intended to be matched or observed, other measures are calculated and reviewed to gain greater visibility into the Model. They may flag something that needs further investigation, or they may be helpful in understanding an issue flagged elsewhere in the model-building and validation processes.

In the tables below that report the results of the calibration exercises, each measure is marked as to whether it is used to seek a match (“M”), is observed (“O”), or is reviewed (“R”).

Calculation of Goodness of Fit of the Model to the Target Value

Calibration is driven by how close the value of the measure calculated by the Model is to the target value. To determine this "goodness of fit," for each measure we calculated a ratio of the calculated value to the target value. (For convenience we call this the “match ratio.”) Ideally the match ratio would be 1 for every measure, implying that the calculated values exactly matched the target values. With this said, the values are never expected to match exactly (i.e., match ratio = 1), due to sampling variability if nothing else, and the acceptable discrepancy between the calculated and target values can vary widely. Reasons include the quality of the data in the data sources, how well the data sources can be mapped to outcomes calculated by the Model, the frequency of the outcomes represented by the measure, and other factors discussed above.

Each of these was considered when defining the acceptable range of the match ratio for a measure. Additional factors considered were the importance of the measure in the disease area (e.g., incidence of MIs in people with diabetes is more important that frequency of FPG tests), and the extent to which a measure addresses the intended uses of the Model. The ranges of match ratios considered acceptable varied from 5% (i.e., a ratio of 0.95 to 1.05) to a factor of 5 (i.e., a ratio of 0.2 to 5), depending on the factors discussed above.

To provide additional context for interpreting the match ratio, we calculated the 95% confidence interval for the match ratio, based on the sample size of the data source and the simulation.

© 2012

Criteria for a measure passing

A measure is considered to "pass" if the match ratio is within the acceptable range or if the 95% confidence interval for ratio includes one. Otherwise the measure "fails." Calibration continues until each of the match measures passes.

Calibration Results

The tables in this section show the results of the calibrations. They show the values of the measures at the start of a simulation.

The method for creating a simulated population are described in the model description and validation papers. Briefly, individuals are sampled from the NHANES dataset. Each sampled individual is returned to birth in the simulation and then "grows up" through the simulation to the age at which they were sampled from NHANES. The proximity of the biomarkers of the simulated individual to their values in NHANES provides a measure of the performance of the Simulator. The results are shown in the tables for biomarkers.

Prevalence and incidence measures are also calculated to evaluate the performance of the Model at baseline. References, where available, are shown for the prevalence and incidence measures. The reference table is at the end of this section. As discussed in the "Methods of Data Collection" section, we do not have data on the incidence of diagnosed conditions, deaths, or hospitalizations for the DM and CAD subpopulations.

In the tables, each measure is marked "M","O", or "R" to indicate whether it is a matched, observed, or reviewed measure.

Demographics

Demographic	Subgroup	Objective	Simulation	Target
Age 20 to 85	Male	O	0.48	0.48
	Black	O	0.11	0.11
	Hispanic	O	0.05	0.05
	Mexican	O	0.08	0.08
	White	O	0.71	0.72
Age 20 to 40	Male	O	0.50	0.50
	Black	R	0.13	0.13
	Hispanic	R	0.07	0.07
	Mexican	R	0.12	0.12
	White	R	0.63	0.64
Age 40 to 65	Male	O	0.49	0.48
	Black	R	0.11	0.11
	Hispanic	R	0.04	0.04
	Mexican	R	0.06	0.05

	White	R	0.74	0.75
Age 65 to 85	Male	O	0.44	0.43
	Black	R	0.08	0.08
	Hispanic	R	0.04	0.04
	Mexican	R	0.03	0.03
	White	R	0.82	0.83
Male	Black	R	0.10	0.10
	Hispanic	R	0.05	0.05
	Mexican	R	0.08	0.08
	White	R	0.71	0.72
Female	Black	R	0.12	0.12
	Hispanic	R	0.06	0.05
	Mexican	R	0.07	0.07
	White	R	0.70	0.71
Diabetic	Male	O	0.48	0.48
	Black	R	0.15	0.17
	Hispanic	R	0.05	0.07
	Mexican	R	0.06	0.08
	White	R	0.71	0.63
CAD	Male	O	0.55	0.58
	Black	O	0.09	0.09
	Hispanic	R	0.04	0.03
	Mexican	R	0.05	0.03
	White	R	0.79	0.81

Biomarkers

US Population Age 20 to 85

Biomarker	Objective	Simulation	Target
age	O	46.37	45.44
bmi	O	28.23	28.25
cholesterol	O	199.65	201.62
creatinine	O	0.90	0.90
dbp	O	71.63	71.68
fastingPlasmaGlucose	O	101.00	103.76
hdl	O	54.07	52.68
HbA1c	R	5.48	5.47
ldl	O	117.98	119.19
sbp	O	121.72	122.47
smoke	O	0.24	0.26
triglycerides	O	139.19	150.73

Age 20 to 40

Biomarker	Objective	Simulation	Target
age	O	30.39	29.45
bmi	O	27.49	27.41
cholesterol	O	188.73	190.37
creatinine	O	0.85	0.86
dbp	O	69.31	69.27
fastingPlasmaGlucose	O	93.16	94.38
hdl	O	52.41	51.53
HbA1c	R	5.15	5.19
ldl	R	111.32	112.45
sbp	O	114.35	114.43
smoke	O	0.30	0.33
triglycerides	O	125.99	133.04

Age 40 to 65

Biomarker	Objective	Simulation	Target
age	O	50.94	53.44
bmi	O	28.86	29.08
cholesterol	O	207.25	210.75
creatinine	O	0.91	0.91
dbp	O	74.97	74.53
fastingPlasmaGlucose	O	103.49	109.65
hdl	O	54.42	53.21
HbA1c	R	5.60	5.66
ldl	R	123.29	125.07
sbp	O	123.59	126.43
smoke	O	0.24	0.24
triglycerides	O	148.79	165.19

Age 65 to 85

Biomarker	Objective	Simulation	Target
age	O	72.90	72.98
bmi	O	28.20	28.11
cholesterol	O	204.37	206.47
creatinine	O	0.99	1.02
dbp	O	67.45	67.08
fastingPlasmaGlucose	O	113.28	116.89
hdl	O	56.97	54.75
HbA1c	R	5.93	5.81
ldl	R	118.79	120.55
sbp	O	134.68	138.32

© 2012

smoke	O	0.10	0.10
triglycerides	O	143.65	156.07

Male

Biomarker	Objective	Simulation	Target
age	O	45.77	44.94
bmi	O	28.13	28.13
cholesterol	O	196.98	200.16
creatinine	O	1.01	1.02
dbp	O	73.05	73.10
fastingPlasmaGlucose	O	101.48	105.72
hdl	O	48.30	47.28
HbA1c	R	5.47	5.50
ldl	R	119.50	119.98
sbp	O	122.93	123.69
smoke	O	0.27	0.29
triglycerides	O	146.99	167.58

Female

Biomarker	Objective	Simulation	Target
age	O	46.92	46.23
bmi	O	28.32	28.34
cholesterol	O	202.14	203.07
creatinine	O	0.79	0.80
dbp	O	70.30	70.25
fastingPlasmaGlucose	O	100.56	101.92
hdl	O	59.40	57.73
HbA1c	R	5.48	5.44
ldl	R	116.55	118.48
sbp	O	120.59	121.52
smoke	O	0.21	0.23
triglycerides	O	131.91	135.08

Diabetic

Biomarker	Objective	Simulation	Target
age	O	60.28	58.35
bmi	O	34.90	32.13
cholesterol	O	195.89	201.89
creatinine	O	1.17	1.00
dbp	O	70.64	69.77
fastingPlasmaGlucose	O	165.77	165.77
hdl	O	51.21	48.69

© 2012

HbA1c	R	7.33	7.32
ldl	R	108.11	112.16
sbp	O	127.18	131.88
smoke	O	0.15	0.20
triglycerides	O	183.79	212.39

CAD

Biomarker	Objective	Simulation	Target
age	O	63.57	63.89
bmi	O	29.96	29.49
cholesterol	O	192.32	196.75
creatinine	O	1.00	1.07
dbp	O	68.38	68.51
fastingPlasmaGlucose	O	120.13	121.31
hdl	O	50.99	49.52
HbA1c	R	6.13	5.91
ldl	R	107.83	112.27
sbp	O	126.34	130.99
smoke	O	0.21	0.21
triglycerides	O	168.70	175.97

Diagnoses – Prevalence

US Population Age 20 to 85

Outcome	Objective	Simulation	Target	Min	Max	Ref.
BilateralBlindness	R	0.002				
DM	O	0.096	0.076	0.073	0.108	1
ESRD	O	0.002	0.001		0.002	1
FootUlcer	R	0.021	0.008			13
MI	O	0.026	0.032		0.042	1
PDR	R	0.008	0.008	0.002	0.034	7
Stage3CKD	O	0.036	0.054			4
Stroke	O	0.027	0.026		0.029	1

Age 20 to 40

Outcome	Objective	Simulation	Target	Min	Max	Ref.
BilateralBlindness	R	0.00013				
DM	O	0.017	0.024		0.038	1
ESRD	R	0.00049				
FootUlcer	R	0.002				
MI	O	0.001	0.005			1

© 2012

PDR	R	0.001				
Stage3CKD	R	0.001				
Stroke	O	0.005	0.004			1

Age 40 to 65

Outcome	Objective	Simulation	Target	Min	Max	Ref.
BilateralBlindness	R	0.001				
DM	O	0.110	0.092		0.116	1
ESRD	R	0.002	0.002			1
FootUlcer	R	0.020				
MI	O	0.022	0.030			1
PDR	R	0.006				
Stage3CKD	R	0.013				
Stroke	O	0.025	0.025			1

Age 65 to 85

Outcome	Objective	Simulation	Target	Min	Max	Ref
BilateralBlindness	R	0.009				
DM	O	0.250	0.171	0.166		1
ESRD	R	0.008			0.004	
FootUlcer	R	0.074				
MI	O	0.101	0.112			1
PDR	R	0.031		0.006		
Stage3CKD	R	0.191				
Stroke	O	0.087	0.089			1

Male

Outcome	Objective	Simulation	Target	Min	Max	Ref
BilateralBlindness	R	0.002				
DM	O	0.096	0.072		0.124	1
ESRD	R	0.002	0.002			1
FootUlcer	R	0.022				
MI	O	0.035	0.041		0.047	1
PDR	R	0.007		0.002		
Stage3CKD	R	0.023				
Stroke	O	0.034	0.021		0.026	1

Female

Outcome	Objective	Simulation	Target	Min	Max	Ref

BilateralBlindness	R	0.002				
DM	O	0.095	0.080		0.099	1
ESRD	R	0.002	0.001			1
FootUlcer	R	0.021				
MI	O	0.018	0.025		0.027	1
PDR	R	0.008		0.001		
Stage3CKD	R	0.048				
Stroke	O	0.021	0.031		0.032	1

Diabetic

Outcome	Objective	Simulation	Target	Min	Max	Ref
BilateralBlindness	R	0.020				
DM	R	1.000	1.000			1
ESRD	O	0.009	0.013			1
FootUlcer	R	0.207				
MI	O	0.091	0.122			1
PDR	R	0.083		0.020		
Stage3CKD	R	0.137				
Stroke	O	0.056	0.118			1

CAD

Outcome	Objective	Simulation	Target	Min	Max	Ref
BilateralBlindness	R	0.010				
DM	O	0.286	0.270		0.292	1
ESRD	R	0.006	0.005			1
FootUlcer	R	0.082				
MI	O	0.460	0.571			1
PDR	R	0.034		0.005		
Stage3CKD	R	0.112				
Stroke	O	0.068	0.160			1

Diagnoses – Incidence

US Population Age 20 – 85

Outcome	Objective	Simulation	Target	Min	Max	Ref
BilateralBlindness	R	0.0002				
DM	O	0.0050	0.0062		0.0074	11
ESRD	O	0.0003	0.0004		0.0011	8
FootUlcer	R	0.0022	0.0027	0.0015	0.0038	23
MI	O	0.0027	0.0034	0.0027	0.0051	12
PDR	R	0.0012	0.0004			11

Stage3CKD	O	0.0035	0.0017			11
Stroke	R	0.0023	0.0040	0.0036	0.0045	12

Age 20 to 40

Outcome	Objective	Simulation	Target	Min	Max	Ref
BilateralBlindness	R	0.00003				
DM	O	0.0021	0.0017			11
ESRD	R	0.0001				
FootUlcer	R	0.0004				
MI	O	0.0002	0.0003			12
PDR	R	0.0002				
Stage3CKD	R	0.0006	0.0001			11
Stroke	O	0.0006	0.0002			12

Age 40 to 65

Outcome	Objective	Simulation	Target	Min	Max	Ref
BilateralBlindness	R	0.0001				
DM	O	0.0071	0.0089			11
ESRD	R	0.0002				
FootUlcer	R	0.0024				
MI	O	0.0028	0.0037			12
PDR	R	0.0011				
Stage3CKD	R	0.0030	0.0030			11
Stroke	O	0.0021	0.0038			12

Age 65 to 85

Outcome	Objective	Simulation	Target	Min	Max	Ref
BilateralBlindness	R	0.0006				
DM	O	0.0061	0.0110			11
ESRD	R	0.0010	0.0016			8
FootUlcer	R	0.0063				
MI	O	0.0084	0.0038			12
PDR	R	0.0040	0.0021			11
Stage3CKD	R	0.0127	0.0038			11
Stroke	O	0.0072	0.0098			12

Male

Outcome	Objective	Simulation	Target	Min	Max	Ref
BilateralBlindness	R	0.0001				
DM	O	0.0054	0.0062			11
ESRD	R	0.0003			0.0006	
FootUlcer	R	0.0022				

© 2012

MI	O	0.0035	0.0040	0.0040	0.0078	12
PDR	R	0.0011	0.0001			11
Stage3CKD	R	0.0032	0.0011			11
Stroke	O	0.0025	0.0040		0.0048	12

Female

Outcome	Objective	Simulation	Target	Min	Max	Ref
BilateralBlindness	R	0.0002				
DM	O	0.0047	0.0087			11
ESRD	R	0.0003			0.0015	
FootUlcer	R	0.0022				
MI	O	0.0018	0.0028	0.0019	0.0033	12
PDR	R	0.0012	0.0007			11
Stage3CKD	R	0.0038	0.0023			11
Stroke	O	0.0021	0.0041			12

Diabetic

Outcome	Objective	Simulation	Target	Min	Max	Ref
BilateralBlindness	R	0.0015				
DM	R	0.0257				
ESRD	R	0.0013				
FootUlcer	R	0.0193				
MI	R	0.0086				
PDR	R	0.0118				
Stage3CKD	R	0.0096				
Stroke	R	0.0049				

CAD

Outcome	Objective	Simulation	Target	Min	Max	Ref
BilateralBlindness	R	0.0007				
DM	R	0.0084				
ESRD	R	0.0005				
FootUlcer	R	0.0072				
MI	R	0.0303				
PDR	R	0.0046				
Stage3CKD	R	0.0079				
Stroke	R	0.0061				

© 2012

Deaths

US Population Age 20 – 85

Cause of Death	Objective	Simulation	Target	Min	Max	Ref
CHDDeath	O	0.0004	0.0014	0.0014	0.0014	3
Death (all cause)	O	0.0084	0.0079			3
StrokeDeath	O	0.0005	0.0004	0.0003	0.0004	3

Age 20 – 45

Cause of Death	Objective	Simulation	Target	Min	Max	Ref
CHDDeath	O	0.00003	0.0001	0.00008	0.00008	17
Death	R	0.0019				17
StrokeDeath	O	0.0001	0.00002	0.00002	0.00002	17

Age 45 – 65

Cause of Death	Objective	Simulation	Target	Min	Max	Ref
CHDDeath	O	0.0004	0.0012	0.0011	0.0012	17
Death	R	0.0072				17
StrokeDeath	O	0.0004	0.0002	0.0002	0.0002	17

Age 65 – 85

Cause of Death	Objective	Simulation	Target	Min	Max	Ref
CHDDeath	O	0.0015	0.0141	0.0141	0.0142	3
Death	O	0.0323	0.0718			3
StrokeDeath	O	0.0023	0.0040	0.0036	0.0043	3

Male

Cause of Death	Objective	Simulation	Target	Min	Max	Ref
CHDDeath	O	0.0005	0.0018	0.0018	0.0018	3
Death	O	0.0097	0.0089			3
StrokeDeath	O	0.0005	0.0003	0.0003	0.0004	3

Female

Cause of Death	Objective	Simulation	Target	Min	Max	Ref
CHDDeath	O	0.0003	0.0011	0.0010	0.0011	3
Death	O	0.0073	0.0069			3
StrokeDeath	O	0.0005	0.0004	0.0003	0.0004	3

© 2012

Diabetic

Cause of Death	Objective	Simulation	Target	Min	Max	Ref
CHDDeath	R	0.0015				
Death	R	0.0214				
StrokeDeath	R	0.0015				

CAD

Cause of Death	Objective	Simulation	Target	Min	Max	Ref
CHDDeath	R	0.0043				
Death	R	0.0278				
StrokeDeath	R	0.0016				

Procedures – Incidence

US Population Age 20 – 85

Treatment	Objective	Simulation	Target	Min	Max	Ref
CABG	M	0.0005	0.0004		0.0015	16
FootAmputation	M	0.0005	0.0004	0.0003	0.0008	16
PCI	M	0.0023	0.0034			16

Age 20 – 40

Treatment	Objective	Simulation	Target	Min	Max	Ref
CABG	R	0.0001	0.00001		0.00003	2
FootAmputation	R	0.0001	0.0001			2
PCI	R	0.0001				

Age 40 – 65

Treatment	Objective	Simulation	Target	Min	Max	Ref
CABG	M	0.0005	0.0004		0.0017	16
FootAmputation	R	0.0004	0.0004			2
PCI	M	0.0025	0.0014		0.0040	16

Age 65 – 85

Treatment	Objective	Simulation	Target	Min	Max	Ref
CABG	M	0.0018	0.0012		0.0049	16
FootAmputation	R	0.0019	0.0011			16
PCI	M	0.0074	0.0032		0.0091	2

© 2012

Male

Treatment	Objective	Simulation	Target	Min	Max	Ref
CABG	R	0.0007	0.0006		0.0024	16
FootAmputation	R	0.0007	0.0005			2
PCI	R	0.0031	0.0016		0.0044	16

Female

Treatment	Objective	Simulation	Target	Min	Max	Ref
CABG	R	0.0004	0.0002		0.0007	2
FootAmputation	R	0.0003	0.0002			16
PCI	R	0.0016	0.0007		0.0021	2

Diabetic

Treatment	Objective	Simulation	Target	Min	Max	Ref
CABG	R	0.0018		0.0041		
FootAmputation	M	0.0048	0.00002			14
PCI	R	0.0075				

CAD

Treatment	Objective	Simulation	Target	Min	Max	Ref
CABG	R	0.0065		0.0055		
FootAmputation	R	0.0021				
PCI	R	0.0269		0.0016		

Interventions – Prevalence

US Population Age 20 – 85

Treatment	Objective	Simulation	Target	Min	Max	Ref
ACEInhibitor	M	0.10	0.09		0.11	1
Antihypertensive	M	0.23	0.24	0.22	0.24	1
Aspirin	M	0.20	0.19		0.36	10
BetaBlocker	M	0.10	0.10	0.08		1
CCB	M	0.07	0.07	0.06		1
Diuretic	M	0.11	0.11	0.10		1
DyslipidemiaMedication	M	0.13	0.13	0.11	0.14	1
Glitazone	R	0.03				
Insulin	R	0.02	0.02	0.01		1
Metformin	R	0.04	0.04			1

OralDMAgent	R	0.07	0.06	0.05		1
Sulfonylurea	R	0.03	0.03			1
WeightLossLifestyle Changes	M	0.16	0.16			1

Age 20 – 40

Treatment	Objective	Simulation	Target	Min	Max	Ref
ACEInhibitor	M	0.03	0.02			1
Antihypertensive	M	0.06	0.04			1
Aspirin	R	0.01				
BetaBlocker	M	0.01	0.01			1
CCB	M	0.01	0.01			1
Diuretic	M	0.02	0.02			1
DyslipidemiaMedication	M	0.03	0.01			1
Glitazone	R	0.004				
Insulin	R	0.004	0.01			1
Metformin	R	0.01	0.02			1
OralDMAgent	R	0.01	0.02			1
Sulfonylurea	R	0.01	0.004			1
WeightLossLifestyle Changes	M	0.09	0.11			1

Age 40 – 65

Treatment	Objective	Simulation	Target	Min	Max	Ref
ACEInhibitor	M	0.10	0.11			1
Antihypertensive	M	0.25	0.27			1
Aspirin	R	0.26				
BetaBlocker	M	0.10	0.11			1
CCB	M	0.07	0.08			1
Diuretic	M	0.12	0.12			1
DyslipidemiaMedication	M	0.14	0.14			1
Glitazone	R	0.03				
Insulin	R	0.02	0.02			1
Metformin	R	0.05	0.04			1
OralDMAgent	R	0.08	0.06			1
Sulfonylurea	R	0.04	0.03			1
WeightLossLifestyle Changes	M	0.18	0.19			1

© 2012

Age 65 – 85

Treatment	Objective	Simulation	Target	Min	Max	Ref
ACEInhibitor	M	0.28	0.25			1
Antihypertensive	M	0.62	0.65			1
Aspirin	R	0.48	0.49			10
BetaBlocker	M	0.34	0.33			1
CCB	M	0.27	0.19			1
Diuretic	M	0.33	0.33			1
DyslipidemiaMedication	M	0.33	0.42			1
Glitazone	R	0.07				
Insulin	R	0.06	0.03			1
Metformin	R	0.11	0.07			1
OralDMAgent	R	0.17	0.14			1
Sulfonylurea	R	0.04	0.07			1
WeightLossLifestyle Changes	M	0.26	0.21			1

Male

Treatment	Objective	Simulation	Target	Min	Max	Ref
ACEInhibitor	M	0.10	10			1
Antihypertensive	M	0.24	0.22			1
Aspirin	R	0.25				
BetaBlocker	M	0.10	0.09			1
CCB	M	0.06	0.06			1
Diuretic	R	0.11	0.09			1
DyslipidemiaMedication	M	0.15	0.14			1
Glitazone	R	0.03				
Insulin	R	0.02	0.01			1
Metformin	R	0.04	0.03			1
OralDMAgent	R	0.07	0.05			1
Sulfonylurea	R	0.03	0.03			1
WeightLossLifestyle Changes	M	0.16	0.15			1

Female

Treatment	Objective	Simulation	Target	Min	Max	Ref.
ACEInhibitor	M	0.10	0.09			1
Antihypertensive	M	0.23	0.25			1
Aspirin	R	0.14				
BetaBlocker	M	0.10	0.11			1

© 2012

CCB	M	0.07	0.07			1
Diuretic	R	0.12	0.13			1
DyslipidemiaMedication	M	0.10	0.12			1
Glitazone	R	0.03				
Insulin	R	0.02	0.02			1
Metformin	R	0.04	0.04			1
OralDMAgent	R	0.07	0.06			1
Sulfonylurea	R	0.03	0.03			1
WeightLossLifestyle Changes	M	0.15	0.18			1

Diabetic

Treatment	Objective	Simulation	Target	Min	Max	Ref
ACEInhibitor	M	0.42	0.41			1
Antihypertensive	M	0.54	0.68			1
Aspirin	R	0.40				
BetaBlocker	M	0.27	0.27			1
CCB	M	0.17	0.17			1
Diuretic	M	0.31	0.30			1
DyslipidemiaMedication	M	0.51	0.49			1
Glitazone	R	0.28				
Insulin	M	0.22	0.22			1
Metformin	M	0.45	0.46			1
OralDMAgent	R	0.72	0.70			1
Sulfonylurea	M	0.29	0.36			1
WeightLossLifestyle Changes	M	0.36	0.36			1

CAD

Treatment	Objective	Simulation	Target	Min	Max	Ref
ACEInhibitor	M	0.38	0.37			1
Antihypertensive	M	0.76	0.76			1
Aspirin	M	0.53	0.54			10
BetaBlocker	M	0.55	0.57			1
CCB	M	0.20	0.23			1
Diuretic	M	0.33	0.36			1
DyslipidemiaMedication	M	0.58	0.59			1
Glitazone	R	0.09				
Insulin	R	0.07	0.07			1
Metformin	R	0.12	0.11			1

© 2012

OralDMAgent	R	0.20	0.20			1
Sulfonylurea	R	0.07	0.11			1
WeightLossLifestyle Changes	M	0.28	0.27			1

Summary and Conclusions

This report describes how care processes and related behaviors are represented in the Model, how the care processes in the Simulator 2.3 version of the Model have been calibrated to the US population and care delivery setting, and the results of the calibration.

Care processes are a critical part of the Archimedes Model. They enable more accurate representations of the processes by which interventions are implemented in realistic settings, the background care against which interventions will be compared, and process improvement programs. They also enable the Model to be customized to different settings.

The calibration of care processes is limited by the quality and quantity of the available data. Particularly important are the sizes of the datasets (especially for the analysis of subpopulations and infrequent events), and the correspondence between outcomes calculated by the Model versus the type of data reported in the data sources (e.g. ICD and CPT codes, and DRGs). These and other factors affect the acceptable range around a target value, and decisions about when calculated values of a measure are sufficiently close to the target values to be considered a satisfactory match. Another factor that is considered when evaluating the calibration of the Model to a particular measure is the importance of the measure to the intended uses of the Model. A final factor is the extent to which the measure is determined by the underlying physiology versus the care processes.

For these reasons, the measures against which the care processes are calibrated are sorted into three categories. Those for which there are good data, that are considered particularly important for the intended applications of the Model, and that are determined primarily by care processes (not the underlying physiology), are used to tune the parameters of the care processes to achieve a good match (" matched" measures). Other measures for which the data are sufficiently good to enable specification of a target value, but that are determined more by the underlying physiology than care processes, are observed but not used to modify parameters of the care processes. They serve primarily as flags to help identify potential improvements in the physiology model, but they are rarely used directly to modify or tune the physiology model ("observed" measures). "Reviewed" measures," a third category, are calculated to gain greater visibility into the Model. They may flag something that needs further investigation, or they may be helpful in understanding an issue flagged elsewhere in the model-building and validation processes.

Results

Overall, for 87% of the match measures, the calculated values were within an acceptable range of their corresponding target values. For the observed measures, the calculated values were within an acceptable range of their target values 83% of the time.

Measures relating to biomarkers and demographics matched their target values very well. Most were within ±5% of the target values. The most important exceptions were seen in the diabetes subpopulation. The calibration process identified the physiology related to BMI and triglycerides in diabetics as opportunities for potential improvement.

Measures relating to prevalence and incidence rates of conditions varied in the extent to which the calculated and target values matched. Measures for the prevalence of chronic conditions defined by biomarkers, such as hypertension and dyslipidemia were very good. This is an important indication that care processes relating to screening for these biomarkers are accurately represented by the Model.

Measures for medication usage matched target values very well. All were within acceptable ranges of their target values except the prevalence of patients diagnosed with diabetes taking sulfonylurea. This measure was too low, even when adherence to the national guideline was set to 100%. This indicates a possibility that in the real world, providers are not observing contraindications for the use of this drug that are specified in the guidelines.

There were too few events and too much variability between data sources to enable specification of meaningful target values for the per-capita incidence rates of procedures. Better data are needed to improve the usefulness of this measure.

Future Work

The calibration of care processes could be improved by the availability of better data — specifically more and larger datasets, with a higher level of clinical detail to enable better mapping of observed events to events calculated by the Model. Larger datasets would also enable better analysis of events in subpopulations and of the events that are infrequent but still clinically important.

© 2012

References for Measures

1 National Health and Nutrition Examination Survey (NHANES) dataset collected by Centers for Disease Control and Prevention (CDC) and National Center for Health Statistics (NCHS). Available online at http://www.cdc.gov/nchs/nhanes.htm.

2 National Hospital Discharge Survey (NHDS) dataset collected by Centers for Disease Control and Prevention (CDC) and National Center for Health Statistics (NCHS). Available online at http://www.cdc.gov/nchs/nhds.htm.

3 Compressed Mortality File (CMF) dataset collected by Centers for Disease Control (CDC) and National Center for Health and Statistics (NCHS), 1999-2007. Available online at http://www.cdc.gov/nchs/data_access/cmf.htm.

4 Prevalence of Chronic Kidney Disease and Associated Risk Factors -- United States, 1999-2004, CDC Morbidity and Mortality Weekly Report, March 2, 2007, 56(08):161-165. Available online at http://www.cdc.gov/mmwr/preview/mmwrhtml/mm5608a2.htm.

5 Surveillance Epidemiology and End Results Cancer Statistics Fast Stats tool, National Cancer Institute. Available online at http://seer.cancer.gov/faststats/selections.php?series=cancer.

6 Blindness Caused by Diabetes - Massachusetts, 1987-1994, CDC Morbidity and Mortality Weekly Report, November 1, 1996, Vol. 45, No. 4. Available online at http://www.cdc.gov/mmwr/PDF/wk/mm4543.pdf.

7 Kempen JH et al. The prevalence of diabetic retinopathy among adults in the United States, Arch Ophth 122(4):552-563.

8 2010 Annual Data Report Reference Tables, United States Renal Data System (USRDS). Available online at http://www.usrds.org/reference.htm.

9 Cases and Deaths by Age 2009, American Cancer Society. Available online at http://www.cancer.org/Research/CancerFactsFigures/cases-and-deaths-by-age-2009.

10 Soni A et al. Aspirin use among the adult U.S. noninstitutionalized population, with and without indicators of heart disease, 2005. Medical Expenditure Panel Survey (MEPS) Statistical brief #179. Available online at http://www.meps.ahrq.gov/mepsweb/data_files/publications/st179/stat179.pdf.

11 Derived composite of NAMCS/NHAMCS OPD and ED.

12 Derived composite of NAMCS/NHAMCS OPD, ED, and NHDS.

13 Moss SE, Klein R, Klein BE. The prevalence and incidence of lower extremity amputation in a diabetic population. Arch Intern Med. 1992; 152:610-616.

14 Derived composite of NAMCS and NHAMCS-OPD.

15 DeFrances CJ et al. 2006 National Hospital Discharge Survey, National Health Statistics Reports Number 5, July 30, 2008. Available online at http://www.cdc.gov/nchs/data/nhsr/nhsr005.pdf.

16 Derived composite of NAMCS/NHAMCS OPD and NHDS.

17 Derived average of low and high estimates from Compressed Mortality File (CMF) dataset collected by Centers for Disease Control (CDC) and National Center for Health and Statistics (NCHS), 1999-2007. Available online at http://www.cdc.gov/nchs/data_access/cmf.htm.

18 Derived average of low and high estimates (i.e. average of NAMCS/NHAMCS OPD and NAMCS/NHAMCS OPD + NHDS).

19 Derived estimate based on the HEDIS national average of number of diabetics taking eye exams and the NHANES reported prevalence of diabetes in population.

20 Internal opinion: Diabetic Retinopathy Interventions Document.

21 Derived composite of NAMCS and NHAMCS-OPD where the high and low estimates are averaged.

© 2012

22 Average of following two references:
Prevalence of Chronic Kidney Disease and Associated Risk Factors -- United States, 1999--2004, CDC Morbidity and Mortality Weekly Report, March 2, 2007 /56(08):161-165. Available online at http://www.cdc.gov/mmwr/preview/mmwrhtml/mm5608a2.htm.
Chronic kidney disease in the general population USRDS 2010 Annual Data Report Slides, USRDS. Available online at http://www.usrds.org/2010/slides/flash/vol1_01_nhanes_10/aP%20Lite%20Flash/index.html.

23 Average of Abbott: Diabet Med 19(5):377-84 and Boyko: Diabetes Care 2006, 29:1202-1207.

ARCHIMEDES
... a KAISER PERMANENTE Innovation
123 Mission Street, 11th floor
San Francisco, CA 94105, USA
www.archimedesmodel.com
info@archimedesmodel.com
+1 415 490 0400

© 2012
