# Supplementary material for: A Standardized Vascular Disease Health Check in Europe: A Cost-Effectiveness Analysis
Source: PLoS One. 2013 Jul 15;8(7):e66454. doi: 10.1371/journal.pone.0066454 (PMC3712021; doi:10.1371/journal.pone.0066454)
Supplement: File S4 — Description of the model validation. (PDF) [file pone.0066454.s004.pdf]

Validation Methodology and Results

ARCHeS Simulator 2.3

December 2011

David Eddy, MD, PhD
Marc-david Cohen, PhD
Kenny Shum, PhD

Table of Contents

Introduction 4

Scope of Validations in this Report 4

External Validation 4

ARCHeS 4

Version Control 4

"One-Click" Validation Suite 5

Objectives of External Validation 5

Types of External Validations 6

Clinical Trials 7

Cohort Follow-Up Studies 8

Age-Specific Incidence Rates 8

Current US Studies 8

Dependent and Independent Validations 9

Validation Coverage 9

Clinical Trials and Cohort Studies 9

Diseases and Outcomes Covered by Validations in the Suite 12

Age-Specific Incidence Rate Studies 13

Additional Studies 15

Validation Methodology 15

Set Up a Simulated Trial That Matches the Real Trial 16

Compare the Baseline Characteristics and Design of the Simulated and Real Trials 17

Run the Simulation 20

Examine Trajectories of Biomarkers 20

Calculate a Metric That Compares the Simulated and Real Results 22

Interpret the Results 23

Draw Conclusions 27

Validation Results 28

Myocardial Infarction 28

© 2011-2012

Stroke 31

Diabetes 31

Mortality 35

Weight Loss 37

Age-Specific Incidence Rates 38

Limitations of External Validations 41

Summary and Conclusion 42

Acknowledgements.. 43

References 44

© 2011-2012

Introduction

This report describes a validation of the Archimedes Model. Specifically, it describes the methods and results of external validation against a suite of studies of Simulator 2.3, of the version and parts of the Archimedes Model that are used by ARCHeS. Each of these elements is described below. This report describes the scope of the validations, the objectives of external validation, the types of studies used for external validations, the specific studies included in the validation suite, the methods used to conduct the validations, the metric used to assess how well the Model reproduces the results of the studies, the results of the validations, limitations of external validations, a summary, and conclusions.

Scope of Validations in this Report

External Validation

The Archimedes Model is subjected to a variety of validation methods [**1**], including face validation, verification or internal validation, cross validation [**2**], external validation [**3**], and predictive validation (see "A Predictive Validation of the CARDS Trial" at www.archimedesmodel.com/tech-reports). Particularly important are the external validations, in which a model is set up to simulate a real study such as a clinical trial, and the model's results are compared to the actual outcomes. External validation of the Archimedes Model was first reported in 2003 [**3**]. Because the Archimedes Model is continually being expanded and updated, it is important to revalidate it every time a new version is released.

ARCHeS

The Archimedes Model includes many diseases. ARCHeS is a web-based interface that enables decision makers to directly access the parts of the Archimedes Model that address cardio-metabolic risk, including diabetes and its complications, coronary artery disease, stroke, dyslipidemia, hypertension, obesity, and smoking. A description of how these parts of the Archimedes Model are built is available at www.archimedesmodel.com/tech-reports. This report describes the validation of those parts.

Version Control

The Archimedes Model was designed for multiple applications and repeated use. To date, more than 100 analyses have been conducted using the Model. To enable application of the Model to multiple problems, many of them being conducted simultaneously, we maintain a "base version" of the Model, or "Base Model," that includes all the variables and equations needed to calculate a person's physiology, the occurrence and outcomes of diseases, and the effects of tests and interventions. Many questions can be answered with the Base Model. When there is a need to conduct an analysis that cannot be done with the Base Model – e.g., it requires a higher level of physiological or pharmacological detail, involves emerging risk factors or novel interventions, or involves different settings and care processes – then

© 2011-2012

appropriate parts of the Base Model are expanded or modified to address the new question[1]. The amended, project-specific versions are saved for those particular analyses, but the base version is maintained unaltered. Over time, new science, technology, and evidence warrant changes to the Base Model. At that point, a new version of the Base Model is released. ARCHeS uses version 2.3 of the Base Model, called "Simulator 2.3," and this report describes the validation of that version. Project-specific versions are validated separately, using studies that are particularly appropriate to each project. Results of those validations are included in the reports of those analyses.

"One-Click" Validation Suite

Conducting both dependent and independent validations is an integral part of the model-building process for Archimedes scientists, and scores of validations are continually being done. They are chosen to address particular parts of the Model and the needs of particular projects, and are set up manually. To date, more than 50 clinical trials and cohort studies have been used to validate the various versions of the Model. To address the need to repeat the validations every time there is a new release of the Base Model, we have created a "one-click" process that automatically calculates validations for a suite of studies[2] chosen to span the populations, interventions, and outcomes addressed by the Base Model. It is "automatic" in the sense that all the instructions for setting up the populations, treatment protocols, and other aspects of the design of each trial (see section on Validation Methodology below) are pre-coded, and the validations can be run by issuing a single instruction. Currently the suite includes 18 studies with a total of 80 different arms and outcomes (validation exercises). Fifteen studies, including 66 validation exercises, address cardio-metabolic risk. This report describes the results for that suite of validations. It does not describe the other validations conducted routinely as part of the model-building process, or in the course of conducting special projects. New studies are continually being added to the suite.

Objectives of External Validation

The essence of external validation is to use the Model to simulate empirical studies and then compare the outcomes that occur in the simulation (hereinafter "simulated outcomes" or "simulated results") with the outcomes of the empirical studies (hereinafter "real outcomes" or "real results"). These validations are intended to serve several objectives. Two of the most important are to enable judgments about how much confidence to place in the Model's results, and to identify limitations in the Model. In addition, the external validations can help users of the Model understand the Model's capabilities, such

---

[1] Examples are "Cardiovascular Outcomes Associated with a New Once-Weekly GLP-1 Receptor Agonist vs. Traditional Therapies for Type 2 Diabetes" [**47**], and "Estimating Health and Economic Benefits from Using Prescription Omega-3 Fatty Acids in Patients with Severe Hypertriglyceridemia" [**46**].

[2] We use the term "study" to describe any source of data or information used to conduct an external validation of the Model. Thus "study" includes clinical trials, cohort follow-up studies, surveys, databases, registries, and statistical reports.

© 2011-2012

as the ability to create virtual populations that match real populations, the ability to simulate detailed treatment protocols, and the ability to adjust the Model to fit different care settings. Studying external validations also helps users of a model determine how much confidence they should place in the model-building process; the care taken in conducting validations is an indication of the care taken in model building. As builders of the Archimedes Model, we have additional objectives. They include determining whether any changes made to the Model between versions affect its accuracy, and identifying parts of the Model that need improvement. From the perspective of model building, making these determinations is the ultimate conclusion of every validation exercise.

Validation is an integral part of the model-building process, constantly checking it and steering it. It is a continual process. A model can never be declared "valid" as though that were a property of the model that applies to all of its applications for all time. The value of validation is not determined by whether it produces a good "score," but by how well it helps the model builders identify ways to improve the model, and by how well it helps users of the model develop confidence in the model's results and the model-building process. The distinction is important. If validations are done to get a good score, one is looking for successes. If the objective is to find ways to improve a model, failures are just as important.

Types of External Validations

Three main types of external validations are conducted: simulation of clinical trials, simulation of cohort follow-up studies, and comparisons of age-specific incidence rates in cohort studies and registries. The distinctions are made because the amount and quality of information available for setting up a validation are different for the three types. The setup of a validation is extremely important. If the setup of a simulation does not match the design of the study[3], one should not expect the results to match. If the results match, but the setup is poor, the match more likely represents a problem than a success. And if there is great uncertainty about the correspondence between the setup and the real trial, then comparison of results can be meaningless. It is never possible to set up a simulation to exactly match a real study, if for no other reason than the information about the study is incomplete. But the closer the match between the setup of a simulation and the design of the real study, the more meaningful and useful the comparison of results.

Studies for inclusion in the "one-click" validation suite were identified using the following process. First, candidate studies where identified by searching PubMed and ClinicalTrials.gov using appropriate search terms. Internal and external advisors were also consulted, particularly to identify the "landmark" trials that are discussed widely in the field. Candidate studies were reviewed to determine their appropriateness for validating the Model for the particular diseases, populations, treatments, and

[3] We use the term "design" to represent all the aspects of a study that affect its results. It includes the inclusion and exclusion of the population, baseline characteristics of the population, treatment protocols, rates of adherence to treatment protocols (e.g., crossover), follow-up protocols, any aspects of background care that are described, and the definitions of outcomes.

© 2011-2012

outcomes in Simulator 2.3. A prioritized list was developed based on the extent to which each trial covered the diseases, populations, treatments, and outcomes of interest, as well as the rigor of the design, and the size, duration, time (current versus old), and location of the study. Another consideration was whether a study had already been used to validate the Model in the past, with some studies that had been subject to past validations being put lower on the list. An additional consideration was the complexity of the design or conduct of the study, with some studies that have very complex designs being put lower on the list[4]. This report describes the results for the studies against which Simulator 2.3 has been validated as of the date of the report. Validations against additional studies are continually being conducted, and their results will be added to future editions of this report.

Clinical Trials

Clinical trials are the most valuable for external validations because they include the most and best information about the design. Publications of the designs of trials include descriptions of the population (inclusion and exclusion criteria, baseline values of characteristics), treatment protocols, compliance rates, and definitions of outcomes. For every trial this information is available at a population level (e.g., mean age, proportion male/female, proportions taking particular treatments), and for an increasing number of trials information is available at a person-specific level. This information enables replication of the trial with relatively high, although not perfect, fidelity. This not only tests the capabilities of the Model to create simulations that match populations and settings of interest, but also improves the interpretation of the validation's results. Because clinical trials involve carefully defined populations,

---

[4] This is done for two reasons. First, studies that have more complex designs are more difficult to simulate accurately. As has been stated above and as will be described in greater detail below, in order to be meaningful, the setup of a validation must achieve a reasonably good match to the design and conduct of the trial. With more complex trials there is a greater risk that the setup will not be close enough to make the results of the simulation meaningful. A second reason is that setting up a validation at the same level of detail as described in the design of the trial can require considerable effort and resources. For these reasons higher priority is given to studies that provide the most valid tests of the Model, using the fewest resources. The potential complexity of a study is well illustrated by the UKPDS trial. The trial involved people who were "newly diagnosed" with diabetes, but their average FPG was about 200 mg/dL, with a very wide distribution. This implies that many of the participants were identified following the development of symptoms rather than based on FPG testing. The UKPDS design then called for everyone to follow a diet, which turned out to have a very large effect on FPG levels, larger than seen in any other diets trials of which we are aware. Following the diet, patients were randomized to "conventional care" and "intensive care" groups, but the actual care received in the conventional care group is not described in detail. Furthermore the treatment goals in the intensive care group were reached by only a small proportion of patients, and glucose levels steadily drifted up over the course of the study. In addition to these issues, the UKPDS included an embedded blood pressure control study, and there were amendments to other aspects of the protocol during the course of the study, such as the addition of metformin to sulfonylurea. Rather than conduct an oversimplified validation exercise whose results would be meaningless, even if there appeared to be a good match, our validation process is to take the time necessary to make the set up match the design of the real trial as closely as possible. A validation of UKPDS 33 is in progress.

ARCHIMEDES
© 2011-2012

they also provide a good test of the Model's ability to address specific populations and represent the effects of particular risk factors and other variables that determine the outcomes of interest. Another advantage is the ability to test the Model's accuracy in calculating the effects of treatments.

Cohort Follow-Up Studies

In cohort follow-up studies there is no manipulation of the treatments patients get, such as randomization into different treatment groups, and there are no treatment protocols. The people in the cohort are simply followed, they receive whatever care is "usual" or "customary" in their settings during the follow-up period, and their outcomes are recorded. Cohort studies usually have less restrictive inclusion/exclusion criteria than clinical trials and usually have longer study durations. Consequently observational studies measure the performance of the Model in broader population settings. The cohort studies we include in the validation suite have a large amount of person-specific data, including biomarkers and outcomes over the study duration. However, these studies rarely provide detailed information about the level of care people are receiving, and it is not possible to match care processes as well as for clinical trials. Furthermore, many of these studies were begun decades ago when care processes were different than today. These limitations need to be considered when interpreting the results.

Age-Specific Incidence Rates

Validation of age-specific incidence rates has a different objective. Instead of trying to match the design of a study and compare the simulated and real results using formal methods, the objective of this type of validation is to see if the base version of the model, which is based on the US population (NHANES) and calibrated to US care processes, generates incidence rates that are reasonably close to those seen in cohort follow-up studies and registries, and that accurately reflect the sex and age dependencies of incidence rates. Because there is no attempt to match the populations and care processes of the studies, there is no expectation that the results will match exactly, and the results are not subjected to statistical analysis. This type of validation is important because many projects done with ARCHeS are done for the general US population and setting. ARCHeS-related outcomes evaluated by this type of validation include first myocardial infarction, first stroke, diabetes diagnosis, end-stage renal disease (ESRD), and all-cause mortality. Because there is no attempt to match the study populations or level of care in the study, these validations must be interpreted more loosely than validations against clinical trials and cohort studies.

Current US Studies

This version of the Base Model represents the US population and care processes. The studies chosen for the validation suite are conducted in the US or similar populations (e.g., Canada, UK, Netherlands), and are as contemporary as possible. Studies from very different cultures (e.g. Japan) have been avoided for this version. Other versions designed for non-US settings or particular settings within the US can be validated using studies pertinent to those settings.

© 2011-2012

Dependent and Independent Validations

As in our previous validation [**3**] and as described in a recent ISPOR/SMDM task force report [**1**], we classify each source as "independent" (no information about the study was used to build the Model), "dependent" (the source was the only one used to build the particular part of the Model being elevated, or the Model was calibrated to fit the source), and "partially dependent" (the source was used to build or calibrate a part of the Model, but that part by itself does not wholly determine the outcome to be validated). Because the category "partially dependent" is so broad, we identify two subcategories. A validation is considered "largely dependent" if the source was one of very few used to build the parts of the Model tested by the validation. A validation is considered "largely independent" if it is only one of a large number of sources used to build the parts of the Model tested by the validation. Subclassification of partially dependent validations also depends on the objective of the validation. This is best illustrated by considering validation of a model against the control and treatment arms of a randomized clinical trial. If the trial was not used to build the physiology model that determines the outcomes in the control arm, and was only one of a few trials used in a meta-analysis to model the effect of treatment, then the validation of the control arm is fully independent, and the validation of the treatment effect is largely dependent. If the goal is to validate how well the model can predict the event rate in the treatment arm, then the validation exercise is largely independent. The reason is that the outcome of a treatment arm is determined primarily by the rate of the outcome in the absence of treatment (i.e. the "control arm"), and to a much lesser extent by the effect of the treatment.

Validation Coverage

Clinical Trials and Cohort Studies

Validation of Simulator 2.3 includes fifteen studies that cover the conditions included in ARCHeS (cardio-metabolic risk).

Table 1 and Table 2 describe the clinical trials and cohort studies in the current validation suite. In general, the outcomes are the primary outcomes of the trial. When primary outcomes are composite outcomes, the components are also included if the necessary data are reported and sample sizes are sufficient to make the results meaningful. If secondary outcomes are modeled in Simulator 2.3, they are included in the validation. For most studies, the outcomes of interest for the validation were health outcomes; for three trials, biomarker values were the outcomes of interest. The first ten studies (Table 1) are clinical trials (ALLHAT [**4**], CARDS [**5**], DPP [**6**], Flechtner-Mors et al. [**7**], HPS [**8**] [**9**], Look AHEAD [**10**], SHEP [**11**], TNT [**12**], UKPDS45 [**13**], and WHI Dietary Modification Trial(DMT) [**14**] [**15**]). Five of the studies (Table 2) are cohort follow-up studies (ARIC cohort [**16**], Framingham Heart Study original cohort [**17**], Medicare 1995 cohort [**18**], SEATTLE [**19**], and WESDR studies on neuropathy [**20**] [**21**] and on retinopathy [**22**] [**23**] [**24**] [**25**] [**26**]).

Table 1. Clinical Trials Used for Validation

Study	Start year	Duration (years)	Population	Age range at entry	Trial arm/ subpopulation (number of participants)	Outcomes and outcome groups tracked
ALLHAT [**4**]	1994	7	Hypertensive with at least one additional CHD risk factor	55+	Diuretic (15,255)<br>ACE (9,054)<br>CCB (9,048)	MI or CHD death; stroke; all-cause mortality.
CARDS [**5**]	1997	4.5	Diabetic with one CVD risk factor but no CHD history (UK & Republic of Ireland)	40 – 75	Placebo (1,410)<br>Atorvastatin (1,428)	MI; stroke.
DPP [**6**]	1996	4	Overweight with elevated fasting and post-load plasma glucose	25+	Control (1,082)<br>Lifestyle (1,079)<br>Metformin (1,073)	Progression to diabetes.
Flechtner-Mors et al [**7**]	NA	0.25	BMI between 25 and 40kg/m^2 (Germany)	18+	Targeted 10% weight loss (50)	Effect of weight loss on SBP, DBP, total cholesterol, HDL, FPG, HbA1c.
HPS [**8**] [**9**]	1994	6	High risk for CVD event (UK)	40 – 80	Placebo (10,267)<br>Simvastatin (10,269)	Major coronary events; stroke.
Look AHEAD [**10**]	2001	1	Type 2 diabetic with BMI > 25 kg/m^2	45 – 74	Lifestyle intervention (2,570)	Effect of weight loss on FPG, HbA1c, SBP, DBP, LDL, HDL.
SHEP [**11**]	1985	5	Isolated systolic hypertension	60+	Placebo (2,371)<br>Diuretic (2,365)	Stroke.

© 2011-2012

TNT [12]	1998	6	Population with clinically evident coronary heart disease (14 countries)	35 – 75	Atorvastatin 10mg (5,006)<br><br>Atorvastatin 80mg<br><br>(4,995)	Nonfatal MI or CHD death; Stroke; Major cardiovascular event.
UKPDS45 [13]	1977	0.25	Newly diagnosed diabetics (UK)	25 – 65	Diet (2,906)	FPG, HbA1c, total and HDL cholesterol.
WHI DMT [14] [15]	1993	8.1	Post-menopausal women without prior breast/colorectal cancers.	50 – 79	Comparison arm (29,294)	Nonfatal MI or CHD death; Stroke; Treated diabetes mellitus.

Table 2. Cohort Follow-up Studies Used for Validation

Study	Start<br><br>year	Duration<br><br>(years)	Population	Age range at entry	Trial arm/subpopulation<br><br>(number of participants)	Outcomes and outcome groups tracked
ARIC cohort [16]	1987	12	General population without MI at baseline	45 – 64	All (12,649)<br><br>Female, non-diabetic (6,533)<br><br>Female, diabetic (665)<br><br>Male, non-diabetic (4,946)<br><br>Male, diabetic (505)	MI; CHD death after first MI; all-cause death after first MI.
FHS original cohort [17]	1948	30+	General population without MI at baseline	30 – 62	All (5,209)<br><br>Female (2,873)<br><br>Male (2,336)	MI.

ARCHIMEDES
© 2011-2012

Medicare cohort [18]	1994	10	US Medicare population	65 – 95	Diabetes-free at baseline (33,772)	All-cause mortality.
SEATTLE [19]	1990	2.6	US veterans with diabetes		All (387)	Sensory neuropathy.
WESDR [20] [21] [22] [23] [24] [25] [26]	1979	10	Diabetic population	30+	Insulin-taking (674)<br><br>Non-insulin taking (696)<br><br>All (1,370)	Diabetic retinopathy (DR);<br><br>Proliferative diabetic retinopathy (PDR);<br><br>Bilateral blindness;<br><br>Foot ulcer;<br><br>Foot amputation.

Of the 15 clinical trials and cohort studies, 11 were not used to build the physiology model in Simulator 2.3 that determines the occurrence and progression of diseases (ALLHAT, CARDS, DPP, HPS, SHEP, TNT, Flechtner-Mors, Look-AHEAD, UKPDS45, WHI DMT, and Medicare cohort). These studies provide independent validations of the physiology model. Six clinical trials (ALLHAT, CARDS, DPP, HPS, SHEP, TNT) were used in combination with other clinical trials to estimate the effects of particular treatments, usually as part of meta-analyses. As described in the previous section (Dependent and Independent Validations), validation of the event rates in the control or placebo arms serve as independent validations, whereas validation of the event rates in the treatment arms serve as largely dependent validations. The remaining four studies (ARIC cohort, Framingham original cohort, SEATTLE, WESDR) were used along with other sources to build the physiology model in Simulator 2.3. Although their data do not wholly determine the outcomes being estimated, the equations they were used to estimate play such central roles in the physiology model that they should be considered dependent validations.

Diseases and Outcomes Covered by Validations in the Suite

To assess how the Model performs in different subpopulations, many health outcomes are validated against multiple studies. Eight studies validate the cardiovascular part of Simulator 2.3. Myocardial infarction incidence rates are validated against results from the ARIC cohort, the Framingham original cohort, and CARDS. Another MI-related outcome is non-fatal MI or CHD death. This composite outcome is validated in the ALLHAT, HPS, TNT, and WHI DMT validations. Each study covers a particular population including the general US population (ARIC, FHS, WHI DMT), the diabetic population (CARDS),

© 2011-2012

and high-risk populations (ALLHAT, HPS, TNT). To validate the stroke model, simulation results are compared against event rates in six studies (ALLHAT, CARDS, HPS, SHEP, TNT, WHI DMT). CHD death and all-cause death after MI are validated against the observed rates in the ARIC subpopulation that had a first MI during the study follow-up.

For diabetes, ARIC and CARDS provide validations for cardiovascular outcomes, and DPP, Look AHEAD, and UKPDS45 validate the progression of biomarkers and the effects of lifestyle interventions. In addition, four studies are used to validate Simulator 2.3 for onset of diabetes and diabetes complications. The onset of diabetes is validated against results from DPP and WHI DMT. The control group of DPP validates the rate of diabetes progression in patients at high risk of developing type 2 diabetes, whereas WHI DMT validates the diabetes incidence rate in a post-menopausal female population. Three outcomes in the retinopathy model – diabetic retinopathy (DR), proliferative diabetic retinopathy (PDR) and bilateral blindness – are checked (dependent validations) against the WESDR cohort. SEATTLE and WESDR provide dependent validations for three outcomes in the neuropathy model: sensory neuropathy, foot ulcer, and foot amputation. Rates of ESRD are calibrated (dependent validation) to the US Renal Data System (USRDS) [**27**]. As of the date of this report, the validation suite does not include and trials of intensive glucose control in people with diabetes. Several such trials are in the process of being added to the suite. In the meantime, results of validations of a previous version of the model against 5 such trials will be shown below, for reference.

The part of Simulator 2.3 that calculates mortality is validated using published results from the Medicare non-diabetic population and ALLHAT. To validate the weight-loss model in Simulator 2.3, the short-term effects of weight loss on biomarkers are compared with results from four clinical trials: DPP, Flechtner-Mors et al., Look AHEAD, and UKPDS45. Biomarkers studied include fasting plasma glucose (FPG), hemoglobin A_{1c} (HbA1c), blood pressures, and lipid levels. These validations evaluate the performance of the weight-loss model in three distinct populations: pre-diabetic, diabetic, and newly diagnosed diabetic.

Age-Specific Incidence Rate Studies

Table 3 describes the cohort studies and registries used to evaluate age-specific incidence rates of events. Simulator 2.3 is based on the US population in that it uses person-specific data from the NHANES 1999-2008 survey to generate the virtual populations used in the simulations. (See the Validation Methodology section below.) The cohorts in most of the datasets in

Table 3 involve populations that have different characteristics than those in the NHANES survey. For example, the people in the Framingham cohorts are entirely white, and the Rotterdam cohort [**28**] is Dutch. The care practices for these cohorts can also be substantially different from the care practices in

Simulator 2.3, which is calibrated to current US national guidelines and utilization rates. Lacking good information about the populations and levels of care, the objective of comparing age-specific incidence rates is only to check that the simulated rates are in reasonable agreement with the real rates, and to check that the simulated incidence rates capture the observed age trends for each gender.

Table 3. Studies Used for Incidence-By-Age Validation

Outcome	Population (data source)	Year	Age
First myocardial infarction	ARIC cohort [**29**]	1987 – 2001	35 – 74
	FHS original cohort and offspring [**29**]	1980 – 2003	35 – 84
	Cardiovascular Health Study (CHS) cohort [**29**]	1989 – 2000	
First stroke	ARIC cohort	1987 – 2001	35 – 74
	CHS cohort	1989 – 1999	65 +
	FHS original cohort and offspring [**30**]	1948 – 2005	45 +
	Rotterdam cohort [**28**]	1990 – 1999	55 +
Diabetes onset	US (National Health Interview Survey (NHIS)) [**31**]	1999 – 2008	18 – 79
	Canadian Study of Health and Aging cohort [**32**]	1991 – 1997	65 +
ESRD	US (United States Renal Data System (USRDS)) [**27**]	2006 – 2008	20 – 85
Cause-specific death: CHD, Stroke	US (National Vital Statistics System (NVSS)) [**33**]	1999 – 2007	20 – 85
All-cause mortality	US (National Vital Statistics Report (NVSR)) [**34**]	2006	20 – 90

© 2011-2012

Additional Studies

In addition to the studies described above, the Model has been validated against many other clinical trials and cohort studies. Some of the validations conducted using an earlier version of the Model have been described in a previous publication [**3**]. Others are done as parts of specific projects. For example, one project involved validating the Model against UKPDS 80 [**35**], ACCORD [**36**], PROactive [**37**], VADT [**38**], and ADVANCE [**39**].[5] Over time, many of these as well as new studies will be added to the automated validation suite; studies currently being added to the suite include ACCORD, Micro-HOPE [**40**], and UKPDS33 [**41**].) Furthermore, as will be described below, results of validation exercises in the current suite can indicate the need for additional validations that target specific diseases, populations, treatments, and/or outcomes. These too will be added to the suite in the future, and this report will be updated.

Validation Methodology

Validations are performed by a validation team that is independent from the model-building team. The full validation methodology is best described for clinical trials because they are most likely to provide information about all of the elements needed to set up a simulation: the population, treatment protocols, compliance rates, definitions of outcomes, and often descriptive information about the setting.

Validation of the Model against a clinical trial involves several steps:

- set up a simulated trial that matches the real trial
- compare the baseline characteristics and design of the simulated trial to those of the real trial and identify potential matches and mismatches
- run the simulation
- examines trajectories of biomarkers to identify potential matches and mismatches
- calculate a metric for how well the results match
- interpret the results in light of the potential matches and mismatches
- draw conclusions about whether the results of the validations indicate a limitation of the model, ways to improve the model, or that no changes need to be made to the model.

Performing these steps at the level of detail required to ensure that the results are meaningful requires considerable effort. On average, performing a validation against a clinical trial takes two to three person months.

---

[5] See http://www.archimedesmodel.com/sites/default/files/Poster-2010-Archimedes-Understanding-Glycemic-Control-Outcomes.pdf

Set Up a Simulated Trial That Matches the Real Trial

This step itself has several parts. The first is to use the Model[6] to generate a virtual population that matches the study population as closely as possible. The process begins by selecting people randomly from the NHANES 1999-2008 database. The Model then creates simulated people, one by one, who match the real people in the database in the sense that when the Model calculates their physiology starting from the birth of the simulated person (age = 0) up to the current age of the real person, the values of biomarkers, medical history, and other variables for the simulated person closely match the values of the real person. Additional information about the methods used to do this is available at www.archimedesmodel.com/tech-reports.

The next part is to screen the simulated people to see if their medical histories and baseline characteristics meet the inclusion and exclusion criteria for the study, as would happen in a real trial. Call this the "simulated trial-eligible population."

The third part is to select a subsample of people from the simulated trial-eligible population so that the subsample (call this the "simulated trial population") has the same baseline characteristics as the real trial population. The Archimedes Model contains automated methods that select simulated people in a way that causes the selected population to converge on any specified targets for biomarkers and other variables, subject to the limitations of the NHANES database. This method achieves the best match possible on each variable, and retains all the correlations present in the US population as represented by NHANES. This is very important: to evaluate the Model, the simulated and real trial populations should match each other as closely as possible with respect to all variables that affect the outcomes of interest. This is necessary to avoid having the comparison of event rates in the simulated and real trials confounded by differences in baseline characteristics. It is also important that these steps be done without using any information about the outcomes of the real trial. Thus in the Archimedes validation process these first three steps are done before the start of the trial simulation. The fact that the sampling process is automated also keeps these steps independent of the final simulation results.

After the simulated population has been created, the next part is to set up the treatment protocol. Treatment protocols can be complicated, involving pre-randomization tests and treatments, withdrawing treatments or giving placebos in control groups, and applying complex protocols for treatment groups. Protocols can involve detailed rules for determining which patients get which treatments, and can involve cascades of treatments intended to bring biomarkers to particular goals (e.g., HbA1c≤7%). In the setup of a simulation it is very important to match these aspects of a trial's design as closely as possible, because they affect the outcomes. The Model includes care processes and behaviors that can be set to replicate the processes and behaviors in real trials fairly closely. Any available information about adherence to treatment protocols can also be incorporated in the setup. The success of this part of the setup process can be checked by comparing the trajectories of biomarkers in the simulated and real trials over the course of the trial, as will be described below.

---

[6] In this description of methods, the version of the Archimedes Model is Simulator 2.3.

© 2011-2012

A related part of the setup is the need to replicate the background level of care that was being delivered at the time the real trial was conducted. The Model includes current guidelines, calibrated to current levels of biomarker control, performance, and compliance. For some trials it is necessary to turn off or modify some healthcare processes in the Model to reflect the level of care patients were receiving at the time the clinical study was conducted. For example, statins were not given to patients in the SHEP trial because the trial was begun before statins were marketed or promoted in guidelines in the United States. In contrast, the base version of the Model includes the ATP III guideline for statins, calibrated to currents rates of performance/adherence. Therefore, for validation of the Model against the SHEP trial, care processes in the Model are modified to "turn off" the use of statins. Similarly, use of aspirin for primary CVD prevention is turned off in a simulated trial if the real trial was conducted before the guideline for aspirin use was issued. All of these are done to the greatest extent permitted by the available information about the trial and the capabilities of the Model.

The final part of the setup is to review the definitions of the outcomes. For example, how was "onset of diabetes" defined: by FPG? OGTT? Both? Either/or? Self-reported? What FPG cutoff level was used: FPG ≥ 126 mg/dL? FPG ≥ 140 mg/dL? What events did the investigators include in their definition of "fatal and non-fatal MI": Admissions only? Include sudden deaths? Include silent MIs?

The parts of the setup just described are required for validations that involve clinical trials for which there is information about the trial's design. When any pieces of this information are not available, it is not possible to match the study as closely. In particular, for cohort studies there is no information about treatment protocols or performance/compliance levels. And for the studies of age-specific incidence rates there may not be good information even about the population. In the last case, the Model is simply run using the NHANES population and current US practices. The importance placed on comparisons of simulated and real results depends on how well the conditions of the real study can be matched by the simulation. This is particularly important for studies of age-specific incidence; because it is not possible to replicate the study populations used in those studies, they can only be used as general checks on the Model.

Compare the Baseline Characteristics and Design of the Simulated and Real Trials

The ability of the Model to create simulated populations that match real populations and the closeness of the match are checked by comparing the baseline information for the simulated and real trials. Typically, this is done using population-level information such as sample means and sample standard deviations, or quartiles of variables. These summary statistics can be placed side by side to assess their similarity, just as real trials compare baseline characteristics of people allocated to control and treatment groups. This step is illustrated with the ARIC cohort study (Table 4). In this case, the simulated and real populations match well on all important baseline characteristics. Depending on how specialized the trial population is (i.e. the extent to which it is not representative of the US population), it is not always possible to achieve this close a match on all the variables. This is illustrated with the ALLHAT trial (Table 5). For this trial most biomarkers are well matched, with the exception of HDL cholesterol (48

© 2011-2012

mg/dL vs. 46.8g/dL in the simulated and real trials, respectively) and total cholesterol (212.1 mg/dL vs. 216.2 mg/dL). Since the MI rate is affected by both HDL and total cholesterol, the combination of these differences could cause the Model to underestimate the MI rate in the ALLHAT trial, and this should be taken into account when interpreting the results of the validation. Tables like these are examined for every clinical trial and cohort study used for validating the Model. As occurs with comparisons of baseline characteristics of different treatment arms in real trials, the matches are never perfect for any variable. And as for real trials, there is no formal method for determining the importance of a mismatch. In general we ask whether the average value of important variables are within 3% for the simulated and real trials, and whether the values of less important variables are within 5%.[7] As with real trials, if there are differences in baseline characteristics that are deemed important, their effects can be explored through sensitivity analyses and related methods.

[7] The "importance" of a risk factor is determined by whether it is in the risk equation for the outcome of interest. Thus the importance of a biomarker depends on the outcome of interest; total and HDL cholesterol levels are important when MI is the outcome of interest, but are less important when the outcome is sensory neuropathy.

© 2011-2012

Table 4. Comparison of Baseline Characteristics of Simulated Population and ARIC Population

Characteristics	Simulation mean	ARIC mean	Simulation std. dev.	ARIC std. dev.
ABI	1.13	1.132	0.13	0.136
Age	54.07	54.07	5.72	5.73
BMI	27.51	27.5	5.2	5.2
CIMT	0.80	0.805	0.19	0.195
DBP	74.3	73.5	11.4	11.1
Fibrinogen	301.0	300.9	66.6	63.9
HDL	52.5	52.5	17.0	17.0
SBP	120.8	120.8	18.4	18.4
Total cholesterol	213.9	214	41.2	41.2
Triglycerides	123.3	122.7	63.3	63.2
% DM or FPG>126	0.09	0.0924		
% hypertensive	0.32	0.332		
% male	0.43	0.43		
% on ace	0.03	0.028		
% on betablocker	0.07	0.075		
% on other bp med	0.10	0.099		
% smoke	0.25	0.253		

Table 5. Comparison of Baseline Characteristics of Simulated Population and ALLHAT Population

Characteristics	Simulation mean	ALLHAT mean	Simulation std. dev.	ALLHAT std. dev.
Age	66.9	66.9	7.6	7.7
BMI	29.9	29.7	6.2	6.2
DBP	88.8	89.0	10.1	10.0
FPG	125.2	123.5	57.0	58.3
HDL	48.5	46.8	14.5	14.7
SBP	156.0	156.0	13.3	16.0
Total cholesterol	212.1	216.2	43.4	43.0
% had type 2 DM	0.37	0.361		

© 2011-2012

% had MI or Stroke	0.24	0.232		
% had HDL-C < 35	0.14	0.116		
% male	0.54	0.531		
% smoke	0.22	0.219		

Run the Simulation

After the simulated trial has been set up, the next step is to run the simulation. The sample size of the simulated trial is set to be the same as the real trial, or up to 20,000 simulated patients per arm for larger trials. The duration of the simulated trial is set to be the same as the duration of the real trial. When the simulation is complete, the important biomarker trajectories and the outcomes of interest are recorded, using the same follow-up protocols described for the real trial.

Examine Trajectories of Biomarkers

After the simulation has been run, we examine the trajectories of biomarkers in the simulated and real trial populations to identify matches and mismatches that could potentially affect a comparison of the results. If necessary, simulated protocols can be modified to improve the match between simulated and real levels of biomarkers. These modifications are done solely to ensure that the populations and effects of treatments in the simulation match what happened in the real trial at the level of biomarkers, so that the comparison of results will be meaningful. The modifications are not based on the results themselves and are not done to make the results match. This is illustrated in Figure 1 and Figure 2, which show the trajectories for systolic blood pressure in the control and treated groups in the SHEP trial. In this trial, there was a significant decrease in blood pressure in the control group (Figure 1). We attributed this to a placebo effect and used the observed effect in the placebo group to estimate the magnitude of the placebo effect. We then applied the same placebo effect to the treatment group (because at the time of the trial they were exposed to the same placebo effect). The combined effect of the placebo effect and the effect of the blood pressure treatment in the treatment group are shown in Figure 2. The close correspondence between the simulated and real blood pressures in this group support our assumption about the placebo effect.

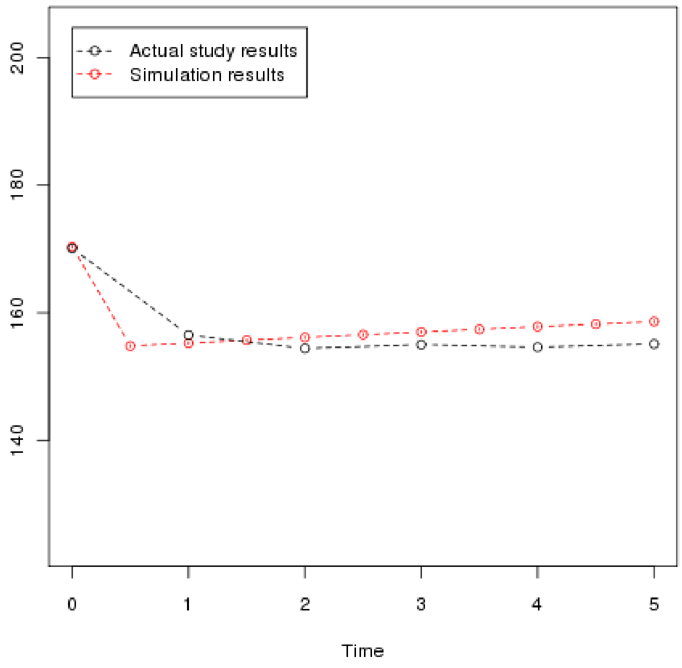

Figure 1. Systolic Blood Pressure in the Placebo Group of SHEP Trial

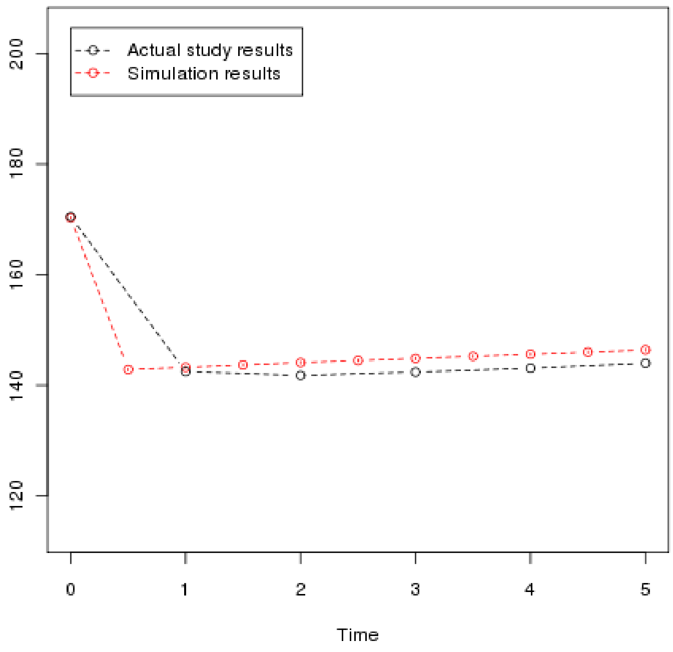

Figure 2. Systolic Blood Pressure in Treatment Group of SHEP Trial

© 2011-2012

Calculate a Metric That Compares the Simulated and Real Results

After a simulation is set up to replicate a clinical trial or cohort study, as described above, the next step is to calculate a metric for how closely the simulation's results match the real results. This is done for each validation exercise. The metric we use is the ratio of the outcome rate(s) in the simulated trial divided by the outcome rate(s) in the real trial. A confidence interval for the ratio is calculated to take into account the number of people at risk at the time(s) the outcomes were recorded. Typically the outcome rates in a real trial are recorded as a Kaplan-Meier curve, and we calculate a Kaplan-Meier curve for the simulated trial. This is desirable because it provides information about the outcomes for all of the time periods over the duration of the trial. It also accounts for censoring. For some studies cumulative rates are reported, in which case the ratio of cumulative rates is calculated. For some studies, only the rate at the end of the follow-up period is reported.

The concept is most easily illustrated with a trial that reports a rate at a single time period. If p_1 is the rate in the simulated trial and p_0 is the rate in the real trial, then the ratio is p_1/p_0. Using the SEATTLE trial as an example, $p_1 = 0.193$, $p_0 = 0.2$, and the ratio is 0.96. This metric takes the value 1 when the rates of outcomes in the simulated and real trials are identical. Statistical uncertainty due to the limited number of people in both the simulated and real trials is taken into account by calculating 95% confidence limits for the ratio of rates, using the formula

$$\frac{p_1}{p_0} \times \exp\left\{ \pm 1.96 \sqrt{\frac{1-p_1}{p_1 n_1} + \frac{1-p_0}{p_0 n_0}} \right\}$$

where n_1 and n_2 are the number of people in the simulated and real trials, respectively, at the time of measurement.

When a study reports outcome rates at several time periods it is possible to choose a particular time point, (e.g., mean follow-up time, median follow-up time, or end of study) and calculate the ratio as just described. However, this has several disadvantages. First, the choice of the particular time point is subjective. If a time point before the end of the study is used, some of the information provided by the trial is ignored. If the end point of the trial is used, the results may have large variability because only a small proportion of patients are followed for the full follow-up duration of the trial. Furthermore, it is important to know how well the Model performs at all time periods, not just one. For these reasons we calculate a metric that compares the entire Kaplan-Meier curve for the simulated and real trial.

We call the resulting metric the "validation hazard ratio" or "vHR." It is calculated by fitting a Cox proportional hazard model to the survival data from the simulation and from the study, where the only explanatory variable is an indicator variable X=1 for patients in the simulation and X=0 for patients in the actual study. The vHR is similar to the hazard ratio used in most randomized clinical trials to show the treatment effect, just renamed to emphasize its use for comparing the simulated and the actual event rates in validations. A classical interpretation is that if the 95% confidence interval of vHR contains 1, there is not sufficient evidence to conclude that the Model's result differs from the real result. This is

ARCHIMEDES
© 2011-2012

equivalent to the log-rank test failing to reject the hypothesis that the simulation and the real study have the same hazard at level α=0.05.

Because the results in different arms of a trial are highly correlated (they share the same calculation of the rate of the outcome without treatment), the interpretation of vHR in a treatment arm should examine the difference between the ratio in the treatment arm and the ratio in the control arm. For example, if the ratio in a control arm is 1.2, and if the effect of the treatment was modeled with perfect accuracy, then one would expect the ratio in the treatment arm to also be 1.2. A ratio of 1.2 would be a successful validation in such a case, whereas a ratio of 1.0 for the treatment arm would indicate an overestimation of the treatment's effect.

Interpret the Results

The metric just described takes the results of the simulated and real trials at face value, and incorporates only the statistical uncertainty in the simulated and real outcome rates. It is important to recall that any mismatches in the setup of the simulation could cause the simulated rate to differ from the observed rate. They include: imperfect matching of baseline characteristics or trial protocols, incomplete reporting of performance and/or compliance to a treatment protocol, changes in standard of care over time, differences in definitions of health outcomes, placebo effects, and so forth.

The next step is to interpret the validation hazard ratio of each validation exercise in the light of any mismatches that could potentially affect the comparison. As stated above, the ultimate objective is to answer the questions: "Is there reason to believe there is a flaw in the Model or code? Is there anything that can be improved? Are there any reasons to change the Model, or should it be left as it is?"

The types of judgments made in this step are illustrated with the CARDS trial. Figure 3 shows the independent validation of the MI rate in the control group of the trial. The validation hazard ratio is 1.2, implying that, on average over the duration of the trial, the Model is overestimating the MI rate by about 20%. Even though the difference is not statistically significant (95% CL: 0.92, 1.57), this result warrants further thought. The decline in MI rate seen in the trial starting at 3 years is also puzzling. We concluded that additional sources should be sought for modeling and validating the effect of diabetes (FPG, A1c, duration of diabetes, total glucose burden) on the development of unstable plaque, clotting, and the occurrence of MIs.

The results for the atorvastatin group (Figure 4) illustrate several additional issues. The vHR is 1.41, implying that the simulation is overestimating the real hazard rate by 41%, on average. First, this illustrates the importance of looking at all time points. If only the results at 5 years are considered, the simulated and real rates are 0.05 and 0.045, respectively. This difference of 10% is well within the confidence limits of the ratio and fails to identify the much larger differences in years 2, 3, and 4. A second point is that the high vHR in the treatment group (1.41) needs to be considered in light of the results in the control group. The results in the treatment group represent the combined effects of the rate in the control group and the effect of the treatment. If the overestimation of the rate without treatment (Figure 3) is taken into account, then the curve for the atorvastatin group in the simulated

© 2011-2012

trial (Figure 4, red line) is lowered about 20% and coincides with the real results (Figure 4, black line) at about the 4.5 year mark, indicating that the Model is capturing the effect of the treatment (difference between control and treatment groups) quite accurately at the end of the trial. One more factor to be considered is that the effect of atorvastatin was estimated from the CARDS trial and ASCOT-LLA [**42**], which showed a smaller effect of atorvastatin on MIs. Thus this is an additional explanation of the overestimation of the rate seen in Figure 4. We concluded that validation of the treatment arm, when considered in light of the results of the control arm (Figure 3) and other factors, does not indicate any need to revise the Model's representation of the effect of atorvastatin on coronary artery atherosclerosis in people with diabetes. This validation will be discussed further in the section on results.

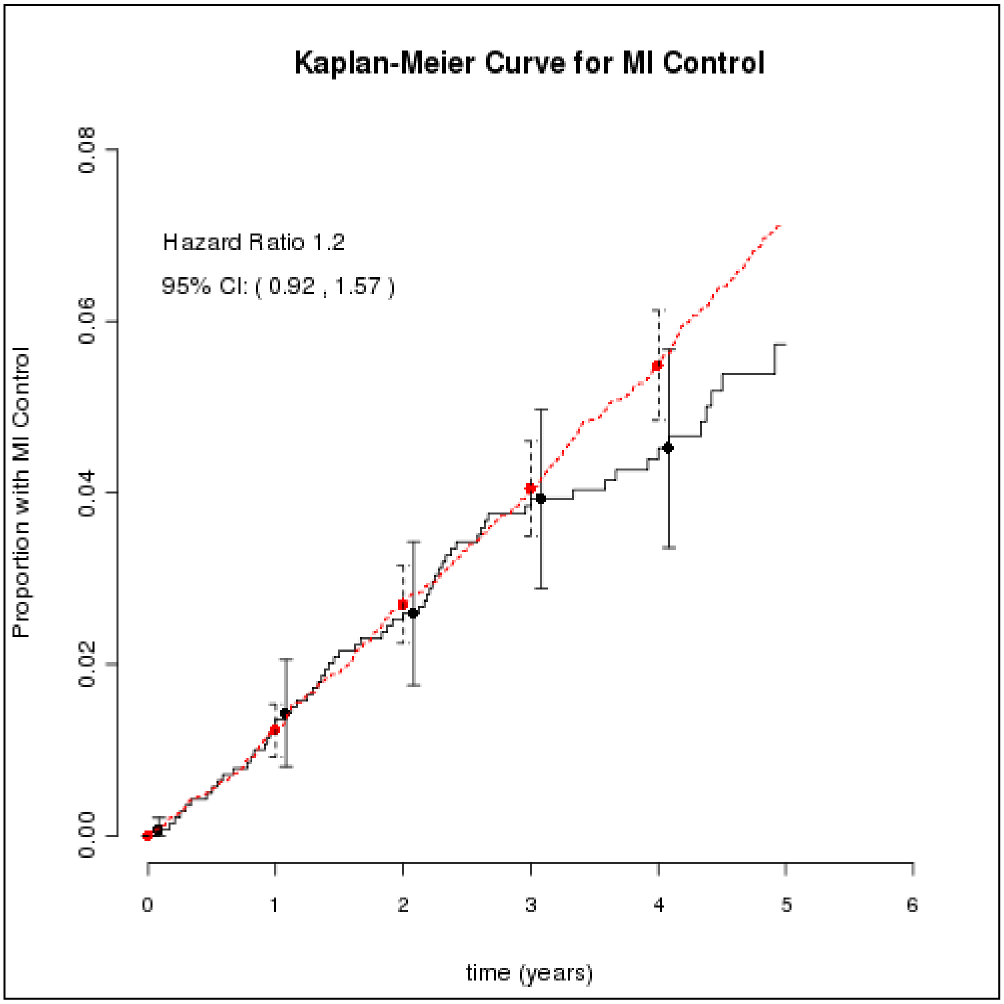

Figure 3. Comparison of Kaplan-Meier Curves for Myocardial Infarctions in the Control Group of the CARDS Trial (red dotted curve – simulation; black solid curve – CARDS)

ARCHIMEDES
© 2011-2012

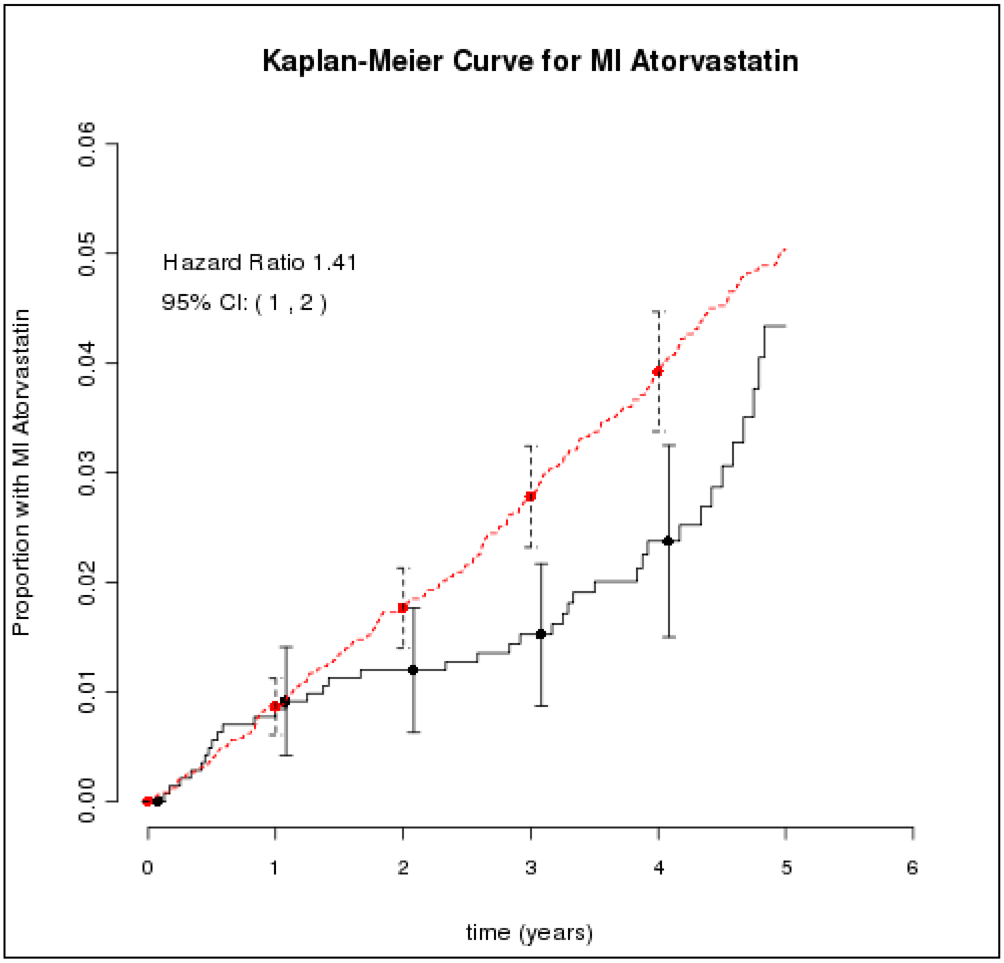

Figure 4. Comparison of Kaplan-Meier Curves for Myocardial Infarctions in the Atorvastatin Group of the CARDS Trial (red dotted curve – simulation; black solid curve – CARDS)

For another example of the interpretation of validations, consider the SHEP trial of diuretics in an elderly population. The results of the validation for stroke in the placebo group (an independent validation) are shown in Figure 5. The validation hazard ratio is less than 1 (vHR = 0.85), implying that the Model is underestimating the real stroke rate by about 15%, averaged over the duration of the trial. The 95% confidence interval for the validation hazard ratio (0.72, 1.02) shows that the simulated rate is not statistically different from the real rate. But beyond this there are several factors that may contribute to the lower simulated event rate. One is the Model's inability to match the age distribution of the patients in the trial. In the trial 13.4% of the participants were over 80 years old, and the fraction of patients over 85 was not reported. The Model uses NHANES population to create the simulated population. The NHANES population is limited to people below age 85, making it impossible to include the group over 85. Because age is an important risk factor for stroke, the missing subpopulation over age 85 could reduce the event rate in the simulated population. Another mismatch in baseline characteristics was caused by a change in the definition of diabetes. The SHEP trial ended before 1997. In 1998 the American Diabetes Association changed the standard for diagnosing diabetes from FPG≥140mg/dL to

FPG≥126mg/dL. Because the base version of the Archimedes Model uses the newer diagnostic criterion, the simulated diabetic patients have lower baseline glucose levels than the diabetic patients in SHEP. Without person-specific data from the SHEP trial it was not possible to adjust for this in the setup. This difference can be expected to lower the simulated event rate. Thus there are at least two explanations for the lower rate of strokes in the simulated trial. Considering all the factors together, a judgment was made that this validation exercise did not indicate any need to revise the parts of the Model that calculate strokes in elderly people with hypertension.

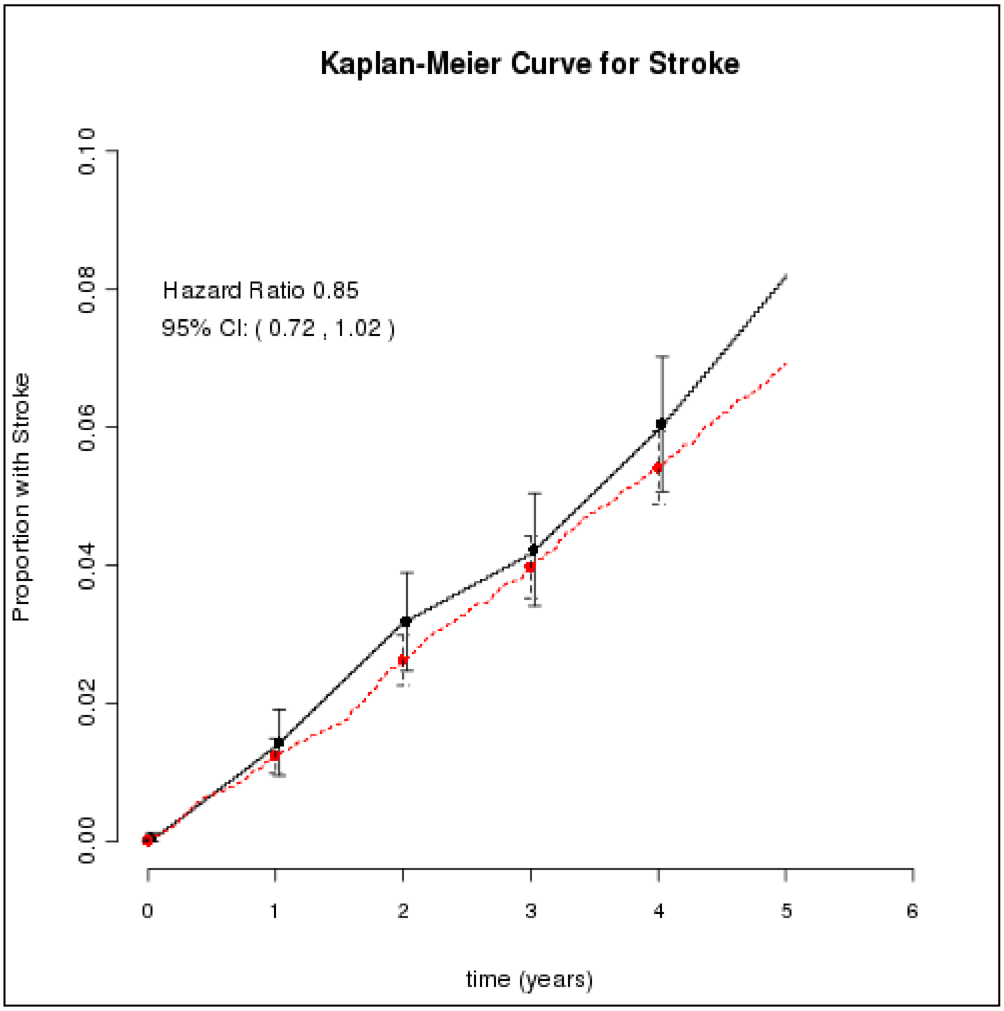

Figure 5. Comparison of Kaplan-Meier Curves for Stroke in the Placebo Group of the SHEP Trial (red dotted curve – simulation; black solid curve – SHEP)

ARCHIMEDES
© 2011-2012

Draw Conclusions

The last step is to draw conclusions about any limitations to the Model, need for additional validations, or need for changes to the Model. Examples of the types of conclusions and reasoning behind them are in the section above. Additional examples are in the section on results of the analyses.

We do not try to calculate an overall score for the Model as a whole. In a previous publication of validations of the Archimedes diabetes model [**3**], for each validation exercise we showed the simulated and actual rates of the outcome for the last observation period in the trial for which there were at least 100 people at risk. We summarized the collection of validation exercises by showing a scatter plot and calculating a correlation coefficient (R) and a coefficient of determination (R^2). We no longer advise using that metric. First, it does not include the results at earlier time points. If the points for all the observation times are included, it is improper to calculate the correlation coefficient or coefficient of determination because the longitudinal data are correlated. The outcomes in the different arms of a trial are also dependent and should not be combined in a calculation of R or R^2. Second, the last observation has the widest range of uncertainty because of the diminishing number of patients at risk. Third, calculation of a correlation coefficient or coefficient of determination is very sensitive to the range of values, and values at the extremes of the range can have a disproportionate effect on the coefficient. For an example drawn from a model built by a different group, consider a set of comparisons of outcome rates in the range of 0% to 10% that have a R^2 of 0.2. The R^2 can be raised to 0.92 simply by adding a study that reported all-cause mortality at 15 years, even though the other group's model was not particularly accurate for that outcome (0.47 and 0.56 in the simulated and treated groups, respectively). The R^2 can be driven to 0.98 by adding a registry result that 99% of people die by age 100. Finally, reporting an overall coefficient of determination (R^2) implies that the simulation's results can be taken at face value, and deemphasizes the importance of examining how well the simulation was set up to match the design of the trials.

With that said, when several validation exercises are conducted for an outcome it is possible to combine multiple validation hazard ratios into a summary statistic. We do this by performing a random-effects meta-analysis of the results of the individual validation exercises for the outcome, using methods that take into account random fluctuations in the rates observed in each trial, and calculating a combined validation hazard ratio. To be meaningful, this should be done for each outcome, one by one. The overall validation hazard ratio and the associated confidence interval together indicate the performance of the Model and the appropriate degree of uncertainty about the Model's overall performance when it is used to simulate the outcome in real-world settings. As in most meta-analyses, a trial inclusion is based on a well-defined but subjective guideline. The decision to include or exclude validation results in the combined hazard ratio can also be subjective. If a researcher is interested in estimating the stroke rate in high-risk population, only validation result in trials involving high-risk population would be of interest. Since our goal is to show the overall performance of the health outcome model, we include all validation exercises in the validation suite. In the current validation suite, the overall validation hazard ratio can be calculated for MI and stroke. The results are shown below.

© 2011-2012

Validation Results

Myocardial Infarction

Validation results for cardiovascular outcomes of the Model[8] are presented in Table 6. In the dependent validations the simulated MI rates match the real rates well for the ARIC cohort overall (vHR: 1.02; CI: 0.93, 1.12), and in all four gender-diabetic status subpopulations (vHR range: 0.94-1.06). The simulated and real results are also quite close for both gender groups of the Framingham original cohort study (vHR: 1.18 among female and vHR: 1.02 among male).

The results of the CARDS trial have already been discussed above but will be summarized here. In the CARDS validation, the comparison of MI incidence rates in the placebo arm serves as an independent validation in the diabetic population without prior CVD event. The simulated rate is not significantly different than the real CARDS result (vHR: 1.20; CI: 0.92, 1.57). The results at the beginning 3 years of the trial are quite similar, but the results in years 4 and 5 are low compared to the simulation (Figure 3). While these results do not indicate any flaws in the Model, we will search for additional sources for independent validations of the effect of diabetes on coronary artery plaque and occurrence of MIs. The result in the atorvastatin treatment arm of the CARDS trial serves as a largely independent validation. This is because, while the MI rate without atorvastatin was calculated without using any data from CARDS (independent), the effect of atorvastatin 10mg/dL in the Model was based on the effect seen in CARDS and ASCOT-LLA. The effect of atorvastatin simulated by the Model is lower than the observed effect in CARDS, leading to a higher rate of MIs and a higher validation hazard ratio (1.41). But about half of the elevation in the ratio is due to the outcome rate in the control group (vHR = 1.2). When the results for the control arm are taken into account, the Model is underestimating the effect of atorvastatin by about 20%. In the simulation the relative risk with atorvastatin is 70% (CI: 66%, 76%), implying that atorvastatin reduces the risk by about 30%. In the real trial the relative risk is 58% (CI: 29%, 86%), implying that atorvastatin reduces the risk by about 42%. (The confidence intervals for the real trial are wide due to the small number of MIs observed in the trial.) A final consideration is that because the effect of atorvastatin was based on CARDS and ASCOT-LLA, and because ASCOT-LLA showed a smaller effect than CARDS, the effect in the Model is expected to be smaller than the effect seen in CARDS. Thus, despite the vHR of 1.41, we concluded that validation of the atorvastatin arm of CARDS does not indicate any flaws in the representation of the effect of atorvastatin on MIs in people with diabetes, and no changes to the Model are indicated. A predictive validation against the CARDS trial of an earlier version of the Model is summarized below and described in detail on the Archimedes website (www.archimedesmodel.com/tech-reports).

The pooled analysis of eight validation exercises shows that the Model on average provides good estimates for the MI rate with an overall validation hazard ratio 1.07 (95% CI: 1.00, 1.14) (Figure 6). Because calculation of the overall validation hazard ratio includes validation hazard ratios from both the

[8] In this section on results the version of the Model is Simulator 2.3.

© 2011-2012

placebo and treatment arms of the CARDS trial, the event rates in the two arms of that trial are correlated. We are currently developing methods to adjust for this type of correlation.

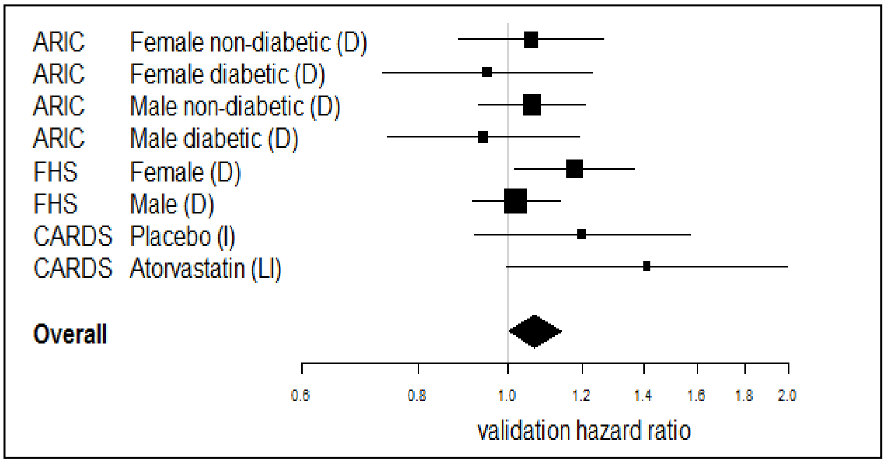

Figure 6. Validation Hazard Ratios of MI Validation Exercises

Another outcome used to validate the MI model is "major coronary event," defined as nonfatal MI or fatal CHD. One challenge in validating this outcome is the different definitions used for fatal CHD. The Archimedes Model calculates CHD death as death after an MI event, whereas in many studies the definitions of CHD death are broader (e.g. including death following angina pectoris or chronic ischemic heart disease). Given this limitation, the simulated event rates for MI/CHD death are expected to be lower than the real rates (i.e. vHR < 1). For the ALLHAT validations, validation hazard ratios for the three trial arms range from 0.75 to 0.87. These results are less than 1 but are considered acceptable because of the explanation just given. For the HPS and TNT validations, validation hazard ratios are between 0.99 and 1.0 which at first glance make the Model look perfect. However, because of the different definitions of CHD death, we expect the Model to slightly overestimate the real risks in these two high-risk populations. With this said, the magnitude of this bias is not considered great enough to cause any changes to this part of the Model. The WHI DMT validation (vHR: 1.14; CI: 0.97, 1.31) suggests that the predicted risk may be high for the WHI population (post-menopausal women without breast or colon cancer), although the difference between the simulated and real results can be explained by chance. Additional validations in ARIC have been conducted to check the CHD death and all-cause mortality after first MI. Both results, which are largely dependent, show that the death rates calculated by the Model are consistent with ARIC results.

© 2011-2012

Table 6. Validation Results for Cardiovascular Disease

Outcome	Study	Trial Arm/	Validation Hazard	95% CI
Myocardial infarction	ARIC	All	1.02 (D)	(0.93, 1.12)
		Female non-diabetic	1.06 (D)	(0.88, 1.26)
		Male non-diabetic	1.06 (D)	(0.93, 1.21)
		Female diabetic	0.95 (D)	(0.74, 1.24)
		Male diabetic	0.94 (D)	(0.74, 1.19)
	FHS	All	1.08 (D)	(0.99, 1.18)
		Female	1.18 (D)	(1.02, 1.37)
		Male	1.02 (D)	(0.92, 1.14)
	CARDS	Placebo	1.20 (I)	(0.92, 1.57)
		Atorvastatin	1.41 (LI)	(1.00, 2.00)
MI or CHD death	ALLHAT	Diuretic	0.75 (LI)	(0.69, 0.82)
		ACE Inhibitor	0.75 (LI)	(0.68, 0.82)
		CCB	0.87 (LI)	(0.79, 0.95)
	HPS	Placebo	1.00 (I)	(0.91, 1.10)
		Simvastatin	0.99 (LI)	(0.89, 1.11)
	TNT	Atorvastatin10	1.00 (I)	(0.89, 1.13)
		Atorvastatin80	0.99 (LI)	(0.86, 1.13)
	WHI	Comparison arm	1.14 (I)	(0.97, 1.31)
CHD death after 1st MI	ARIC		1.05 (LD)	(0.81, 1.37)
Any death after 1st MI	ARIC		1.22 (LD)	(1.02, 1.45)
Stroke	ALLHAT	Diuretic	0.95 (LI)	(0.85, 1.06)
		ACE Inhibitor	0.89 (LI)	(0.79, 1.00)
		CCB	0.97 (LI)	(0.86, 1.11)
	CARDS	Placebo	0.92 (I)	(0.65, 1.32)
		Atorvastatin	1.14 (LI)	(0.71, 1.82)
	HPS	Placebo	0.79 (I)	(0.70, 0.89)
		Simvastatin	0.79 (LI)	(0.69, 0.90)
	SHEP	Placebo	0.85 (I)	(0.72, 1.02)
		Diuretic	0.95 (LI)	(0.76, 1.17)
	TNT	Atorvastatin10	0.98 (LI)	(0.78, 1.24)
		Atorvastatin80	0.93 (LI)	(0.71, 1.22)
	WHI	Comparison arm	1.46 (I)	(1.30, 1.63)

---

[9] D = dependent, LD = largely dependent, LI –largely independent, I = independent. See discussion under Types of External Validations.

Stroke

The stroke model produces incidence rates that are consistent with the results from ALLHAT, CARDS, SHEP, and TNT (vHR range: 0.85-1.14). The validation hazard ratio for the HPS placebo arm is slightly low at 0.79 (CI: 0.69, 0.90). This can be explained by a mismatch between the virtual and the actual HPS population. Specifically, in the HPS trial 7.3% of participants had transient ischemic attacks (TIA). The base version of the Archimedes Model currently does not include TIA, and this subpopulation was not included in the simulation. Because this group is at higher than average risk of stroke, the simulated population is expected to have lower stroke risk than the HPS population. For this reason, and in light of the other validations (ALLHAT, CARDS, SHEP, and TNT), a judgment was made that the results of the HPS validation are not sufficient to cause any changes to the Model. In the WHI DMT validation, the validation hazard ratio of 1.46 (CI: 1.30, 1.63) indicates an overestimation of the risk of stroke in post-menopausal women. While there were some small mismatches in the baseline characteristics of the simulated and real populations, they were not considered sufficiently large to explain this magnitude of overestimation. Thus a judgment was made that the parts of the Model that determine the occurrence of strokes in postmenopausal women needs closer examination, and additional validation sources are being analyzed. The pooled analysis shows the overall validation hazard ratio for stroke is 0.95 (95% CI: 0.84, 1.07) (Figure 7).

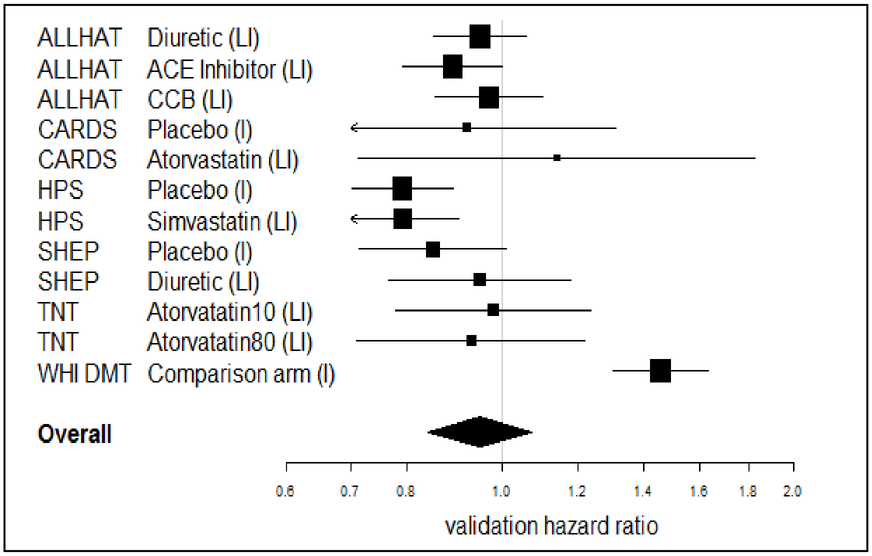

Figure 7. Validation Hazard Ratios of Stroke Validation Exercises

Diabetes

Validation results for diabetes are presented in

Table 7. Macrovascular outcomes (MI and stroke) in people with diabetes are validated using the ARIC study and the CARDS trial, which have already been presented (

Table 6) but are reproduced here for convenience. The onset of diabetes in a pre-diabetic population is validated using the DPP trial. The simulated rate for the control arm is slightly lower than the real rate (vHR: 0.80, CI: 0.70, 0.90). Although this difference is unlikely to be due to chance, the lower rate in the simulation is consistent with other studies. Indeed, in their report of the results the DPP [**43**] the investigators themselves interpreted the trial's results as unexpectedly high. They wrote that "The incidence of diabetes in our placebo group (11.0 cases per 100 person-years) was higher than we had anticipated" [**43**] and was higher than the incidence in observational studies [**44**]. Considering the body of evidence, a judgment was made that the diabetes progression rate in high-risk populations in the Archimedes Model is reasonable, and this validation exercise does not indicate a need to change that part of the Model.

For the lifestyle intervention arm, the simulated rate of progression to diabetes is lower than the rate seen in the DPP (vHR: 0.63, CI: 0.52, 0.76). Even when the lower rate in the control group is taken into account (vHR: 0.8), the Model is still slightly overestimating the effect of the lifestyle program on progression to diabetes (64% (95% CI: 61-67%) reduction in the simulation versus 58% (95% CI: 48-66%) reduction in reported in the real trial. One possible explanation is the changes in rates of compliance to the interventions over the duration of the trial. In the lifestyle intervention group, compliance to the intervention dropped from 74% at 24 weeks to 58% at the last visit. In the simulation, the compliance was set at a constant compliance rate of 66%, chosen to represent the average compliance rate in the trial. If the compliance rate in the lifestyle intervention arm is reduced to match the rate observed at the end of the study, the effect of treatment in the simulation drops from 64% towards the real effect (58%).

For the metformin arm, the estimated diabetes progression rate is low (vHR: 0.32, CI: 0.27, 0.38). The explanation is an error in the part of the Model that calculates OGTT. In the DPP trial, a person was defined to have diabetes if his or her FPG exceeded 125 mg/dL OR his or her OGTT exceeded 199 mg/dL. The Model calculates the effect of metformin on FPG quite accurately (Figure 8). In the Model, OGTT is strongly influenced by FPG, as well as other variables, so the Model also calculated a reduction in OGTT, which in turn lowered the rate of progression to diabetes. However, in the DPP metformin did not reduce OGTT by the same magnitude as metformin reduced FPG, and therefore the trial showed a smaller reduction in progression to diabetes. Based on this validation and other findings, the role of OGTT and its calculation in the Model is being reconsidered. Until the calculation of OGTT is improved,, diabetes projects will focus on FPG and HbA1c.

The diabetes model is also validated against the WHI DMT study. Since the definition of diabetes used in that trial is self-reported, treated diabetes, the rate of diagnosed diabetes as calculated in the simulation has to be adjusted to match that definition. To make this adjustment we used two rates published in the trial report. At baseline, 6.0% participants reported they were diagnosed with diabetes and 4.5% reported they were treated for diabetes, implying that for every 100 treated persons, there were 134 diagnosed persons. Using this adjustment, the validation hazard ratio was 0.73 (CI: 0.69, 0.78). Given the uncertainty in this adjustment, we concluded that the results of the WHI DMT validation exercises did not by themselves indicate or justify any changes to the Model.

© 2011-2012

Table 7. Validation Results for Diabetes

Outcome	Study	Trial Arm/ Subpopulation	Validation Hazard Ratio[10]	95% CI
MI	ARIC	Female diabetic	0.95 (D)	(0.74, 1.24)
		Male diabetic	0.94 (D)	(0.74, 1.19)
	CARDS	Placebo	1.20 (I)	(0.92, 1.57)
		Atorvastatin	1.41 (LI)	(1.00, 2.00)
Stroke	CARDS	Placebo	0.92 (I)	(0.65, 1.32)
		Atorvastatin	1.14 (LI)	(0.71, 1.82)
Diabetes incidence	DPP	Control	0.80 (I)	(0.70, 0.90)
		Lifestyle	0.63 (I)	(0.52, 0.76)
	WHI DMT	Metformin	0.32 (I)	(0.27, 0.38)
		Comparison arm	0.73 (I)	(0.69, 0.78)
Diabetic retinopathy[11]	WESDR	Insulin dependent	0.85 (LD)	(0.76, 0.95)
		Non-insulin dependent	0.80 (LD)	(0.72, 0.88)
Proliferative retinopathy	WESDR	Insulin dependent	0.63 (D)	(0.48, 0.79)
		Non-insulin dependent	1.21 (D)	(0.77, 1.65)
Bilateral Blindness	WESDR	Insulin dependent	1.06 (D)	(0.51, 1.61)
		Non-insulin dependent	0.49 (D)	(0.23, 0.75)
Sensory Neuropathy	SEATTLE		0.96 (D)	(0.75, 1.35)
Ulcer	WESDR		1.12 (D)	(0.89, 1.39)
Foot amputation	WESDR		1.15 (D)	(0.80, 1.52)

[10] D = dependent, LD = largely dependent, LI –largely independent, I = independent. See discussion under Types of External Validations.

[11] Includes non-proliferative as well as proliferative retinopathy.

ARCHIMEDES
© 2011-2012

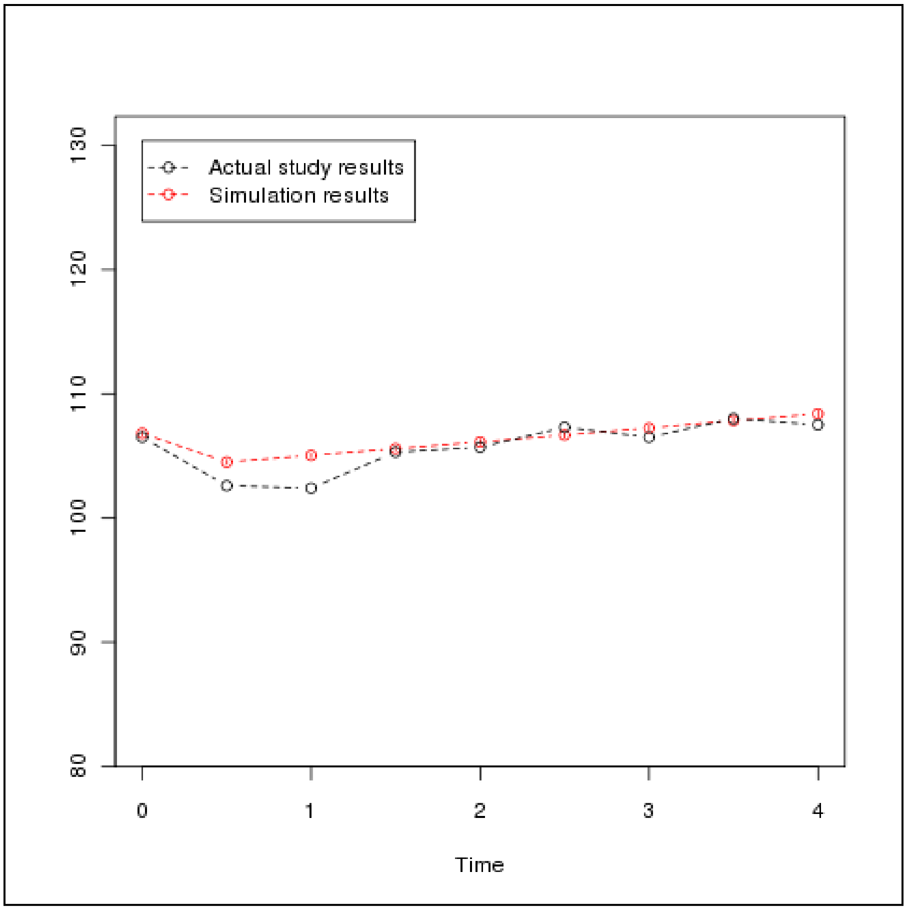

Figure 8. Average FPG in DPP Metformin Arm (red curve – simulation; black curve – DPP)

The incidence rate for diabetic retinopathy is validated against WESDR. WESDR is a partially-dependent validation because results from WESDR are used along with data from other sources [**45**] to build the retinopathy model. For diabetic retinopathy the results of the simulations are consistent with the results in WESDR, with validation hazard ratios of 0.85 in people with insulin-dependent diabetes and 0.80 in people with non-insulin dependent diabetes. For proliferative diabetic retinopathy, the simulated rate is consistent with real rate in the non-insulin dependent subpopulation (vHR: 1.21; CI: 0.77, 1.65), but low for the insulin-dependent subpopulation (vHR: 0.63; CI: 0.48, 0.79). The results for bilateral blindness have a similar pattern: consistent in the insulin-dependent subpopulation (vHR: 1.06; CI: 0.51, 1.61), and low in the non-insulin dependent subpopulation (vHR: 0.49; CI: 0.23, 0.75). While it is gratifying that the Model is accurate for the WESDR population as a whole (combining the results for the insulin dependent and non-insulin dependent sub-populations), these results do indicate that there are likely to be aspects of the different populations that the retinopathy model is not capturing. This is currently under study. For the three dependent validations of the neuropathy model, the simulated results are consistent with the actual results (vHR range: 0.96-1.15). Additional validations of the diabetes model are underway.

Currently the validation suite does not include any trials of intensive glucose control in people with diabetes. Several such trials are in the process of being added to the suite. Because of the importance of

these trials, we show here the results of validations against five of them conducted as part of a project sponsored by the American Diabetes Association [**35**] [**36**] [**37**] [**38**] [**39**]. These validations used an earlier version of the Model, and the results have been presented as a poster session (http://www.archimedesmodel.com/sites/default/files/Poster-2010-Archimedes-Understanding-Glycemic-Control-Outcomes.pdf).

The main results are in Table 8. In 15 of the exercises the Model's results were within the 95% confidence limits of the real results. In the other three, the confidence intervals of the simulated and real trials overlapped. While these trials are not yet part of the one-click validation suite, they are helpful for evaluating the accuracy of the Model for calculating macrovascular outcomes in diabetes populations.

Table 8. Results of Validations of the VAST Version of the Model against Five Clinical Trials of Glucose Control

Study	Trial Arm	Outcome					
		Myocardial Infarction		Stroke		Composite (MI, stroke, CVD death)	
		Trial	Model	Trial	Model	Trial	Model
UKPDS 80 [**35**]	Sulfonlyurea-insulin conventional	28.0%	25.1%†	10.2%	11.1%		
	Sulfonlyurea-insulin intensive	24.8%	22.4%†	9.5%	9.0%		
	Metformin conventional	30.7%	33.0%	10.2%	12.3%		
	Metformin intensive	23.7%	22.0%	9.9%	8.4%		
PROactive [**36**]	Standard	5.5%	5.6%	4.1%	3.2%†		
	Intensive	4.6%	4.5%	3.3%	3.1%		
VADT [**37**]	Control	10.0%	9.3%	5.0%	4.1%		
	Intensive	9.0%	8.4%	4.0%	3.9%		
ACCORD [**38**]	Standard					7.2%	7.7%
	Intensive					6.9%	6.9%
ADVANCE [**39**]	Standard					10.6%	10.6%
	Intensive					10.0%	9.3%

Mortality

Results of validations relating to overall mortality are shown in Table 9. In the Archimedes Model, overall mortality is based on 2006 data from the CDC National Vital Statistics Report (NVSR) [**34**]. For an independent validation (subject to the fact that both are based on the US population), we used a study of a Medicare cohort followed from 1994 and 2004 to validate the Model's calculation of overall

ARCHIMEDES
© 2011-2012

mortality in people over 65. The simulated results were slightly low (vHR: 0.86; CI: 0.83, 0.88). Simulated mortality rates are comparable to ALLHAT results with validation hazard ratios ranging from 0.85 to 0.88 for the three trial arms.

To help understand the difference observed in the morality model validations, we conducted a dependent validation comparing simulated age-specific death rate against those reported in NVSR. Figure 9 shows that the Model captures the increase in death rate up to age 80; however, the Model underestimates the death rate after age 80. This result in the very elderly population is consistent with the findings in the all-cause mortality validation in the Medicare non-diabetic population where the simulated rate is approximately 14% low. Part of this difference can be explained by the definition used for CHD death in the Archimedes Model. This issue will be discussed further in the section on age-specific incidence rates. We believe that ongoing work on the part of the Model that calculates CHD death model, to account for all CHD death, will increase the total death rate and improve these validation metrics. Users of ARCHeS should note that the estimated all-cause mortality rate in the very elderly population will be slightly low.

Table 9. Validation Results for Mortality Model

Outcome	Study	Trial Arm/ Subpopulation	Validation Hazard Ratio[12]	95% CI
All-cause mortality	Medicare	Non-diabetic	0.86 (I)	(0.83, 0.88)
	ALLHAT	Diuretic	0.85 (LI)	(0.80, 0.91)
		ACE inhibitor	0.86 (LI)	(0.80, 0.92)
		CCB	0.88 (LI)	(0.82, 0.95)

[12] D = dependent, LD = largely dependent, LI –largely independent, I = independent. See discussion under Types of External Validations.

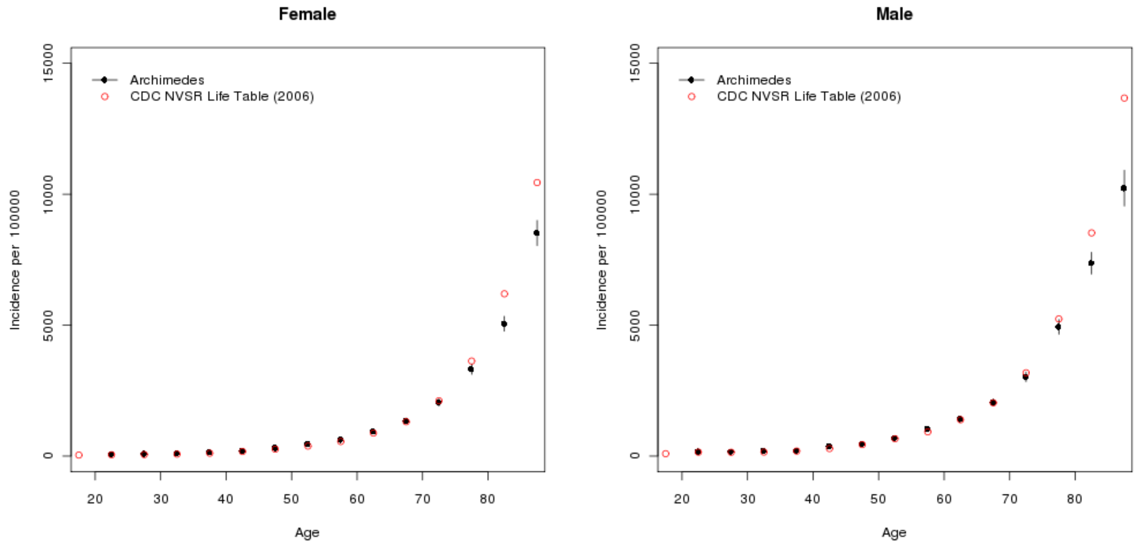

Figure 9. All-Cause Mortality Incidence by Age

Weight Loss

In the Archimedes Model the effects of interventions are drawn from meta-analyses either performed by us or published by others. As expected, they validate well against the combined results of the trials. Weight loss is more complicated because it affects so many other biomarkers. For this reason, and because of the large amount of interest in weight-loss programs, we include its validation against four trials in the automated validation suite. The short-term effects of weight loss on various biomarkers are independently validated by comparing the change in biomarker values relative to the baseline values (expressed as a percent change). Biomarker changes in the simulations should be similar to those observed in the trial, when the patients in the simulations are caused to lose the same amount of weight as occurred in the participants in the real trials. In the four trials, weight loss ranged from 5.4% to 8.6% of baseline weight (Table 10). In the DPP, Flechtner-Mors and Look AHEAD validations, the magnitude of changes in glucose concentrations, blood pressures, and lipid concentrations are similar between the simulation and the actual trial. The percent change in FPG and HbA1c are both within 2% in the DPP pre-diabetic population and the Look AHEAD diabetic population. In the UKPDS45 validation, the simulated changes in cholesterol levels are similar to those observed in the trial, but the reductions in FPG and HbA1c are about half what was observed in the trial. The average FPG in the simulation decreased by 16.7%, which is lower than the 25.8% decrease seen in the trial. The average reduction in HbA1c was 10.7% and 21.4% in the Model and trial, respectively. The UKPDS appears to be an outlier. For the other three trials, the differences between the simulated effect of weight loss on FPG and the real effect (both expressed as percent changes) is less than 1.8%. The change in FPG seen in the UKPDS45 was far larger and almost twice what was calculated by the Model (25.8% decrease in FPG in the trial versus 16.7% decrease in the simulation). The “newly diagnosed” patients in the UKPDS entered with baseline FPG levels averaging approximately 200 mg/dL. They were put on a diet that reduced the average FPG to

ARCHIMEDES
© 2011-2012

approximately 150 mg/dL. To our knowledge the effects seen in UKPDS45 have never been replicated in any other study. Our conclusion was to not change the weight-loss model to fit the UKPDS45 results.

Table 10. Validation Results of Weight-Loss Model

Study follow-up examination)	Biomarker	Archimedes % change	Study % change	Difference in % change
DPP (month 6)	BMI	-6.9	-6.8	-0.1
	FPG	-4.8	-4.3	-0.5
	HbA1c	-2.2	-1.5	-0.7
Flechtner-Mors et al. (month 3)	BMI	-7.7	-7.7	0.0
	FPG	-6.0	-7.8	1.8
	SBP	-4.7	-6.5	1.8
	DBP	-4.8	-2.4	-2.4
	Total cholesterol	-0.7	-0.7	0.0
	HDL	1.4	-0.8	2.2
Look AHEAD (month 12)	BMI	-9.0	-8.6	-0.4
	FPG	-15.4	-14.2	-1.2
	HbA1c	-9.3	-8.8	-0.5
	SBP	-6.3	-5.3	-1.0
	DBP	-7.0	-4.1	-2.9
	LDL cholesterol	-3.2	-4.6	1.4
	HDL cholesterol	5.8	7.8	-2.0
UKPDS45 (month 3)	BMI	-5.4	-5.4	0.0
	FPG	-16.7	-25.8	9.1
	HbA1c	-10.7	-21.4	10.7
	Total cholesterol	-3.5	-3.6	0.1
	HDL cholesterol	4.3	1.5	2.8

Age-Specific Incidence Rates

Results of the validations of age-specific incidence rates are shown in Figure 10 through Figure 15. For most health outcomes, the Model captures the effect of age and gender on incidence rates quite well. In Figure 9 we showed that the mortality model underestimates the all-cause mortality rate in people over age 80. One reason for this is the way CHD death is modeled in the Archimedes Model. Figure 11 compares the simulated age-specific incidence of CHD death for each gender against rates reported by National Vital Statistics System [**33**] using two definitions. One definition is that CHD death includes only death following acute myocardial infarctions (ICD10: I21-22). When this definition is used, the death rates simulated by the Model are similar to those reported in the NVSS. Another definition is more

broad – ischemic heart disease death – and includes additional codes (ICD10: I20-25). When this definition is used, the Model underestimates all-cause mortality for people in older age groups. This issue is currently being addressed. The incidence rates for diabetes for the age group 65 to 79 are lower than the rates observed in the National Health Interview Survey. For all other health outcomes, the simulated incidence rates are similar to the real rates.

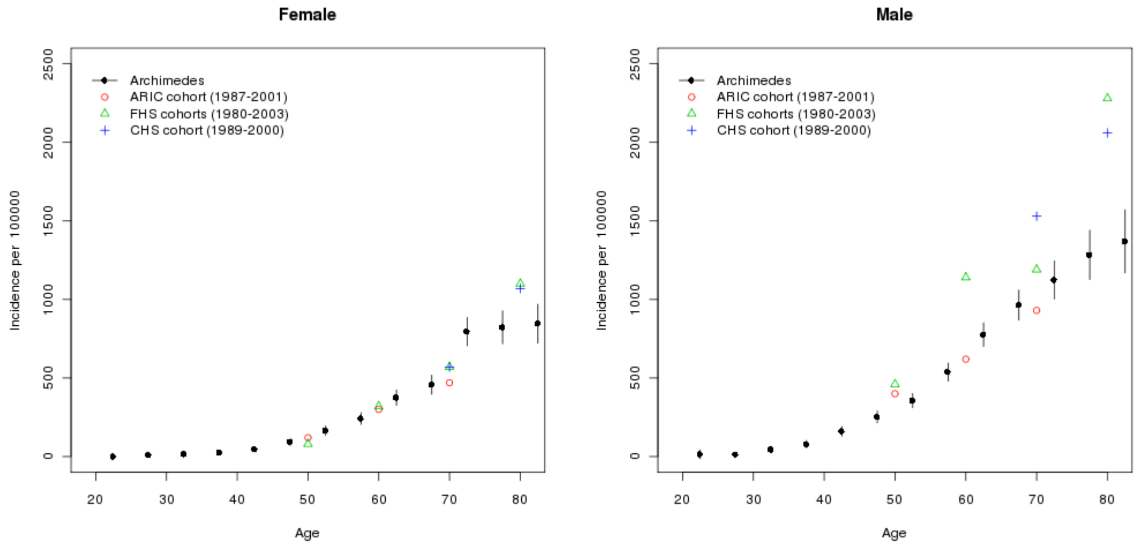

Figure 10. First Myocardial Infarction Incidence by Age

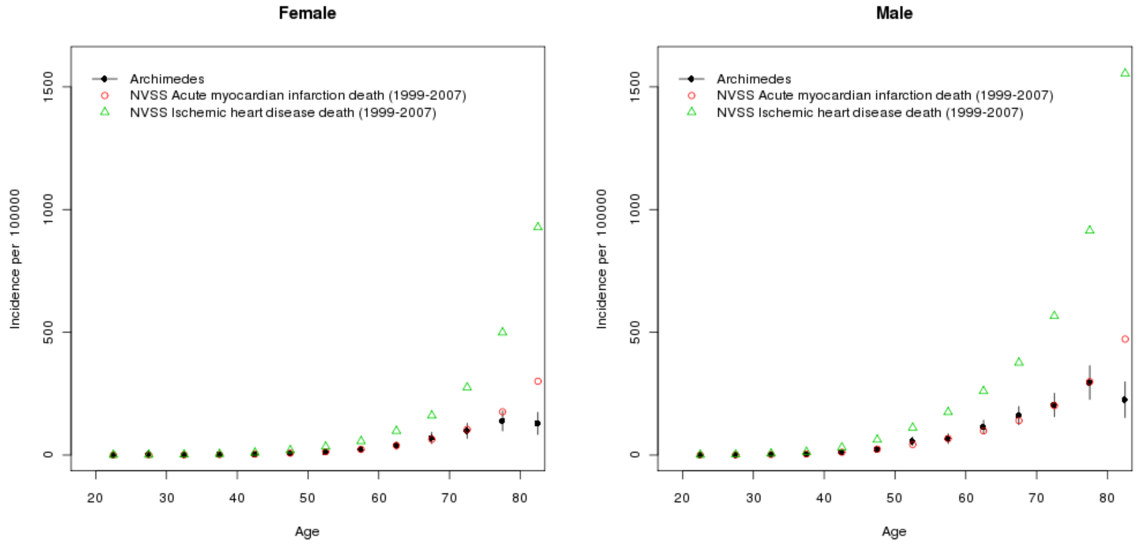

Figure 11. CHD Death Incidence by Age

ARCHIMEDES
© 2011-2012

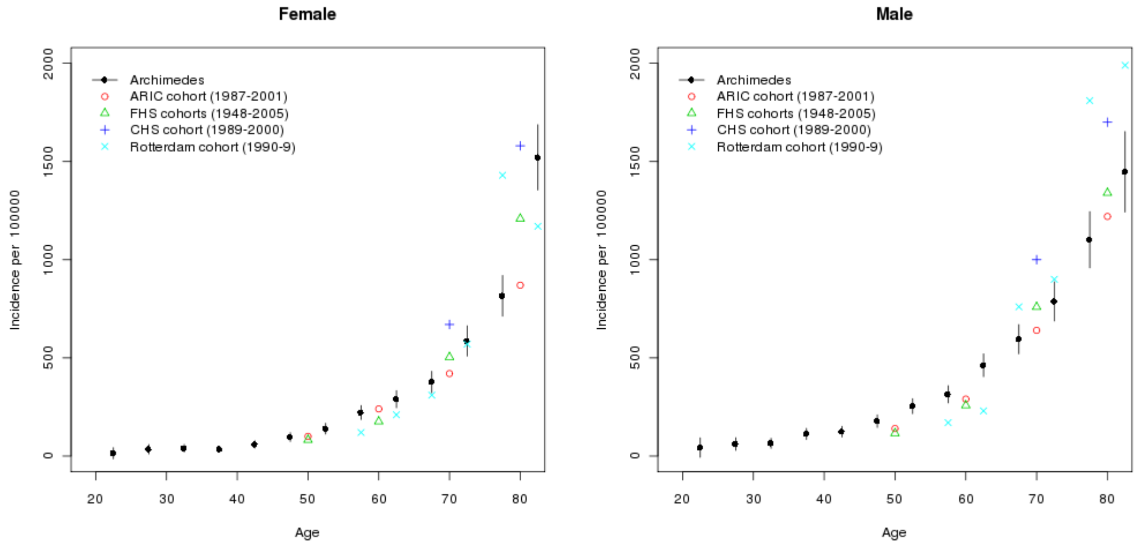

Figure 12. First Stroke Incidence by Age

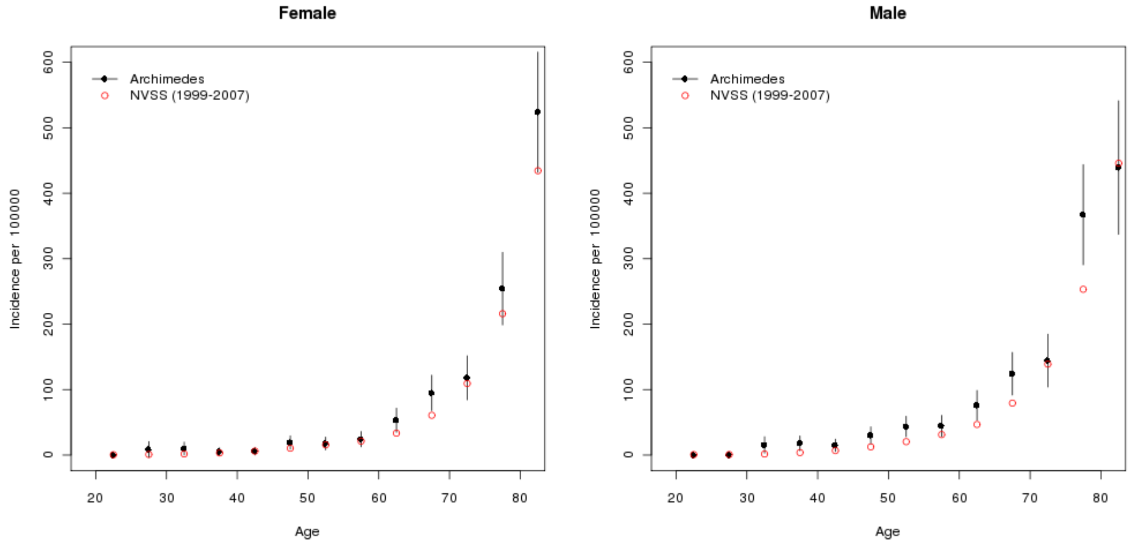

Figure 13. Stroke Death Incidence by Age

ARCHIMEDES
© 2011-2012

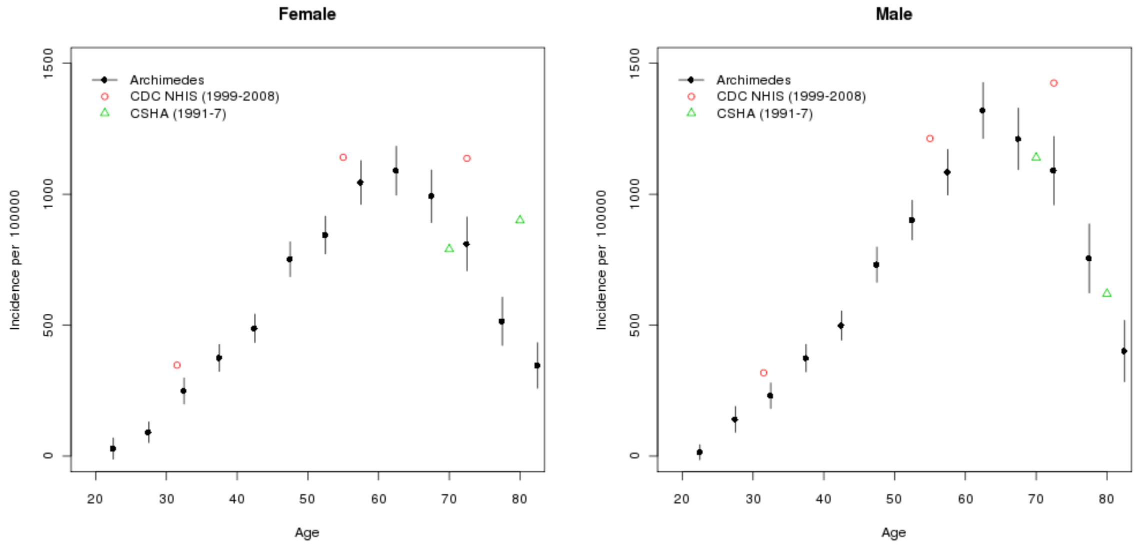

Figure 14. Diabetes Onset Incidence by Age

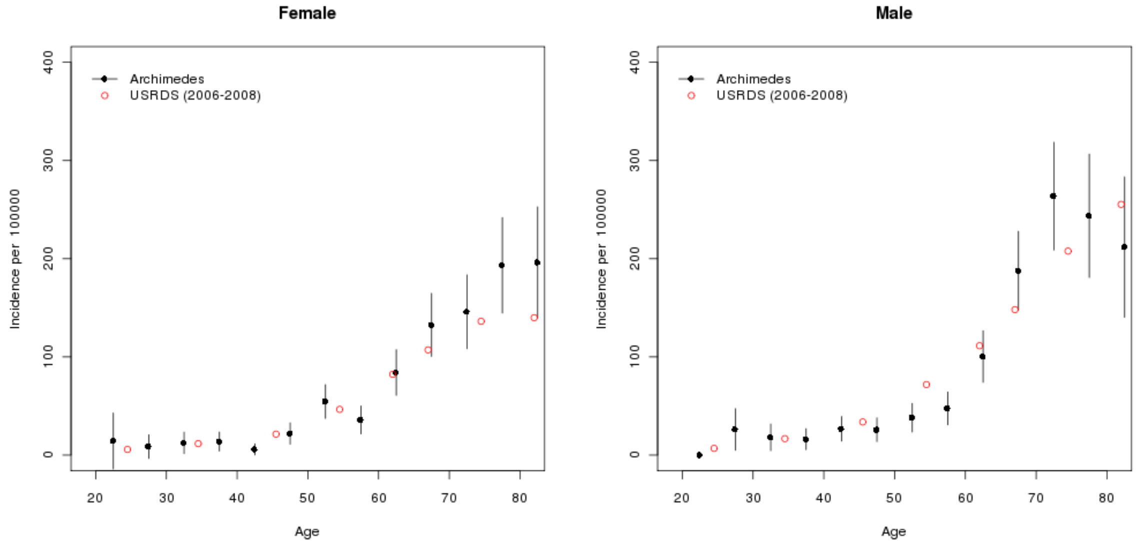

Figure 15. ESRD Incidence by Age

Limitations of External Validations

External validations have several inherent limitations [**1**]. Some of the most important are:

ARCHIMEDES
© 2011-2012

- As stressed in the sections above, the meaningfulness of any validation exercise depends on how well the model can replicate the design and conduct of the study.
- Even if the setup of a simulation matches the design and conduct of a study well, it may not accurately represent what actually happened because of changes during the conduct of the study. In addition, there are almost always factors that affect outcomes but that are not reported, and may not even be known.
- External validations only check a model's accuracy for the settings, populations, interventions, and outcomes in the studies used for the validations. They do not guarantee a model's accuracy for other settings, populations, interventions, and outcomes.
- Even within a trial, accurate matching of aggregated results does not validate results for subpopulations at a lower level of aggregation.
- External validations cannot validate the accuracy of a model for estimating rates of procedures, resource use, or costs. Those outcomes are strongly affected by local practice patterns, which are known to vary widely in different settings. For these outcomes the appropriate approach is to validate the model for calculating the rates of the resource-consuming and cost-generating events (e.g., occurrence of MIs) and then customize the calculation of procedure rates, utilization, and costs to particular settings based on local practices.

In addition to these general limitations, each validation exercise has its own limitations, primarily due to unavoidable mismatches between the simulated study and the real study.

In summary, like every other aspect of healthcare, external validations are only as good as the available evidence. The history of medicine is full of surprises. A suite of successful validations can indicate how well a model represents the current state of medical science and evidence. But it cannot guarantee that the model will be correct for questions that lie between or beyond the existing evidence. However, with this said, external validations are by far the best way to determine how well a model does represent the current state of science and evidence, and they provide the most rigorous way to build and improve models.

Summary and Conclusion

This report describes the external validation of the current base version of the Archimedes Model (Simulator 2.3) used in ARCHeS, against a suite of studies. Objectives are to enable judgments about: how much confidence to place in the Model's results; the ability of the Model to match real populations, treatment protocols, outcomes, and settings; the model-building process; limitations in the Model; and ways to improve the Model and its validation. It describes the methods used to conduct the validations, including setting up a simulation that matches the design of the real study, analyzing the setup to identify mismatches that could potentially affect interpretation of the results, running the simulation, calculating a metric for how well the simulated results match the real results, interpreting the results, and drawing conclusions about the Model.

© 2011-2012

The suite of studies used in this validation includes 15 clinical trials and cohort studies, for a total of 66 validation exercises involving MI, stroke, diabetes incidence, diabetes complications, all-cause death, and CVD death. The results for MIs show a good correspondence between the simulated and real results for all populations (primary and secondary prevention, people with and without diabetes). The results for CHD death show that the Model slightly underestimates the real results for this outcome. This is explained by the definition of CHD death used in the Model, which includes only deaths due to myocardial infarctions and does not include deaths due to non-infarction ischemic disease. When adjustment is made for this difference in definitions, the simulated results are quite consistent with the real results. The results for strokes also show a good correspondence to the real results, with the exception that the Model underestimates the rate of strokes in postmenopausal women. This is being examined. Incidence rates of diabetes calculated by the Model correspond reasonably well to incidence rates seen in people at high risk of developing diabetes. Simulation of the effect of metformin on the progression to diabetes highlighted a limitation of the Model with respect to calculation of oral glucose tolerance. This biomarker will not be included in ARCHeS or projects until this limitation is corrected or a decision is made to exclude OGTT entirely. The validation of diabetes incidence in postmenopausal average-risk women indicates that the Model's results may be low for this population. This is being examined. Validations of microvascular outcomes in people with diabetes indicate that the retinopathy model should be subjected to additional validations and most likely can be improved, particularly for people with type 2 diabetes.

Acknowledgements

The Model has been evolving since originally conceived by Dr. Eddy and first given life by Dr. Schlessinger so many years ago. As this document shows it has reached a level of maturity and a sophisticated state marked by careful review and continual scrutiny, evaluation, and improvement. This document marks but one post on this path of continuous growth. We are indebted to many who contributed and continue to contribute in this journey. Particularly, we are indebted to Dr. Kenny Shum for his leadership and for challenging all of us to be very careful in how we quantify Model quality. Of course many others have contributed, and we are indebted to them as well: Carl Anderson and Andrew Van Herick for making sure that our tools could handle the job; Julia Dziuba and Swathi Guda for helping to interpret the effects of the healthcare system and treatment protocols when questions arose; Tuan Dinh, Ian Blumenfeld, Joyce Noah-Vanhoucke, Stuart Samuel, Helene Grossman, and Andy Schuetz for helping to interpret the effects of the physiologies and helping with understanding the "right" way to model trials; Roshan Shah and Amory Schlender for their assistance; Barbara Peskin, Andrea Kress, and Jennifer Burton for careful editing and hard work in making this document unambiguous; Don Morris for supporting the vision of a rigorous and independent testing team; Brian Zuzga and Paul Jasper for providing leadership and direction in architectural integrity and coding standards; Patrick van der Valk for keeping the documentation process moving; and finally, all the former Archimedeans on whose shoulders we stand.

© 2011-2012

References

[1] Eddy DM, Hollingworth W, Caro JJ, Tsevat J, McDonald KM, Wong JB., "Model Transparency and Validation: A Report of the ISPOR-SMDM Modeling Good Research Practices Task Force Working Group - Part 4," *Value in Health*, p. in press, 2011.

[2] The Mount Hood 4 Modeling Group, "Computer Modeling of Diabetes and Its Complications: A report on the Fourth Mount Hood Challenge Meeting," *Diabetes Care*, vol. 30, pp. 1638-1646, 2007.

[3] Eddy DM, Schlessinger L, "Validation of the Archimedes Diabetes Model," *Diabetes Care*, vol. 26, no. 11, pp. 3102-3110, 2003.

[4] Allhat Officers Coordinators for the Allhat Collaborative Research Group, "Major outcomes in high-risk hypertensive patients randomized to angiotensin-converting enzyme inhibitor or calcium channel blocker vs diuretic: The Antinhypertensive and Lipid-Lowering Treatment to Prevent Heart Attack Trial (ALLHAT)," *JAMA: The Journal of the American Medical Association*, vol. 288, no. 23, pp. 2981-2997, 2002.

[5] Colhoun HM, et al., "Primary prevention of cardiovascular disease with atorvastatin in type 2 diabetes in the Collaborative Atorvastatin Diabetes Study (CARDS): multicentre randomised placebo-controlled trial," *Lancet*, vol. 364, no. 9435, pp. 685-696, 2004.

[6] Knowler WC, et al., "Reduction in the incidence of type 2 diabetes with lifestyle intervention or metformin," *The New England Journal of Medicine*, vol. 345, no. 6, pp. 393-403, 2002.

[7] Flechtner-Mors M, et al., "Metabolic and weight loss effects of long-term dietary intervention in obese patients: four-year results," *Obesity Research*, vol. 8, no. 5, pp. 399-402, 2000.

[8] Heart Protection Study Collaborative Group, "MRC/BHF Heart Protection Study of cholesterol lowering with simvastatin in 20,536 high-risk individuals: a randomised placebo-controlled trial," *Lancet*, vol. 360, no. 9326, pp. 7-22, 2002.

[9] Collins R, et al., "Effects of cholesterol-lowering with simvastatin on stroke and other major vascular events in 20536 people with cerebrovascular disease or other high-risk conditions," *Lancet*, vol. 363, no. 9411, pp. 757-767, 2004.

[10] Look Ahead Research Group, "Reduction in weight and cardiovascular disease risk factors in individuals with type 2 diabetes: one-year results of the Look AHEAD trial," *Diabetes Care*, vol. 30, no. 6, pp. 1374-1383, 2007.

[11] SHEP Cooperative Research Group, "Prevention of stroke by antihypertensive drug treatment in older persons with isolated systolic hypertension. Final results of the Systolic Hypertension in the

© 2011-2012

Elderly Program (SHEP)," *JAMA: The Journal of the American Medical Association*, vol. 265, no. 24, pp. 3255-3264, 1991.

[12] LaRosa JC, et al., "Intensive lipid lowering with atorvastatin in patients with stable coronary disease," *The New England Journal of Medicine*, vol. 352, no. 14, pp. 1425-1435, 2005.

[13] Manley SE, et al., "Effects of three months' diet after diagnosis of Type 2 diabetes on plasma lipids and lipoproteins (UKPDS 45). UK Prospective Diabetes Study Group," *Diabetic Medicine: A Journal of the British Diabetic Association*, vol. 17, no. 7, pp. 518-523, 2000.

[14] Howard BV, et al., "Low-fat dietary pattern and risk of cardiovascular disease: the Women's Health Iniative Randomized Controlled Dietary Modification Trial," *JAMA*, vol. 295, no. 6, pp. 655-666, 2006.

[15] Tinker LF, et al., "Low-fat dietary pattern and risk of treated diabetes mellitus in postmenopausal women: the Women's Health Initiative randomized controlled dietary modification trial," *Archives of Internal Medicine*, vol. 168, no. 14, pp. 1500-1511, 2008.

[16] The ARIC Investigators, "The Atherosclerosis Risk in Communities (ARIC) study: design and objectives," *American Journal of Epidemiology*, vol. 129, no. 14, pp. 687-702, 1989.

[17] Dawber TR, Meadors GF, and Moore Jr FE, "Epidemiological approaches to heart disease: the Framingham Study," *American Journal of Public Health and the Nation's Health*, vol. 41, no. 3, pp. 279-281, 1951.

[18] Bethel MA, et al., "Longitudinal incidence and prevalence of adverse outcomes of diabetes mellitus in elderly patients," *Archives of Internal Medicine*, vol. 167, no. 9, pp. 921-927, 2007.

[19] Adler AI, et al., "Risk factors for diabetic peripheral sensory neuropathy. Results of the Seattle Prospective Diabetic Foot Study," *Diabetes Care*, vol. 20, no. 7, pp. 1162-1167, 1997.

[20] Moss SE, Klein R, and Klein BE, "The prevalence and incidence of lower extremity amputation in a diabetic population," *Archives of Internal Medicine*, vol. 152, no. 3, pp. 610-616, 1992.

[21] Moss SE, Klein R, and Klein BE, "Long-term incidence of lower-extremity amputations in a diabetic population," *Archives of Family Medicine*, vol. 5, no. 7, pp. 391-398, 1996.

[22] Klein R, et al., "The Wisconsin Epidemiologic Study of Diabetic Retinopathy. III. Prevalence and risk of diabetic retinopathy when age at diagnosis is 30 years or more," *Archives of Ophthalmology*, vol. 102, no. 4, pp. 527-532, 1984.

[23] Moss SE, Klein R, and Klein BE, "Ten-year incidence of visual loss in a diabetic population,"

© 2011-2012

Ophthalmology, vol. 101, no. 6, pp. 1061-1070, 1994.

[24] Moss SE, Klein R, and Klein BE, "The incidence of vision loss in a diabetic population," *Ophthalmology*, vol. 95, no. 10, pp. 1340-1348, 1988.

[25] Klein R, et al., "The Wisconsin Epidemiologic Study of Diabetic Retinopathy. XIV. Ten-year incidence and progression of diabetic retinopathy," *Archives of Ophthalmology*, vol. 112, no. 9, pp. 1217-1228, 1994.

[26] Klein R, et al., "The Wisconsin Epidemiologic Study of Diabetic Retinopathy. X. Four-year incidence and progression of diabetic retinopathy when age at diagnosis is 30 years or more," *Archives of Ophthalmology*, vol. 107, no. 2, pp. 244-249, 1989.

[27] US Renal Data System, "USRDS 2010 Annual Data Report: Atlas of Chronic Kidney Disease and End-Stage Renal Disease in the United States," National Institutes of Health, National Institute of Diabetes and Digestive and Kidney Diseases, Bethesda, MD, 2010.

[28] Hollander M, et al., "Incidence, risk, and case fatality of first ever stroke in the elderly population. The Rotterdam Study," *Journal of Neurology, Neurosurgery and Psychiatry*, vol. 74, no. 3, pp. 317-321, 2003.

[29] National Insititutes of Health National Heart Lung and Blood Institute, "Incidence & Prevalence: 2006 Chart Book on Cardiovascular and Lung Diseases," 2006.

[30] Petrea RE, et al., "Gender differences in stroke incidence and poststroke disability in the Framingham heart study," *Stroke*, vol. 40, no. 4, pp. 1032-1037, 2009.

[31] Incidence of Diagnosed Diabetes per 1,000 Population Aged 18-79 Years, by Sex and Age, United States, 1997-2009. [Online]. http://www.cdc.gov/diabetes/statistics/incidence/fig5.htm

[32] Rockwood K, et al., "Incidence and outcomes of diabetes mellitus in elderly people in Canada: report from the Canadian Study of Health and Aging," *Canadian Medical Association Journal*, vol. 162, no. 6, pp. 769-772, 2000.

[33] Death Rates from 113 Selected Causes, Specified Hispanic Origin, Race for Non-Hispanic Population, United States, 2006. [Online]. http://www.cdc.gov/nchs/nvss/mortality/gmwkh210r.htm

[34] Arias, E, "United States Life Tables, 2006," National Center for Health Statistics, Hyattsville, MD, National Vital Statistics Reports vol 58 no 21, 2010.

[35] Holman RR, Paul SK, Bethel MA, Matthews DR, Neil HA, "10-year follow-up of intensive glucose control in type 2 diabetes," *New England Journal of Medicine*, vol. 359, no. 15, pp. 1577-89, 2008.

© 2011-2012

[36] The Action to Control Cardiovascular Risk in Diabetes Study Group, "Effects of intensive glucose lowering in type 2 diabetes," *New England Journal of Medicine*, vol. 358, pp. 2545-2559, 2008.

[37] Dormandy JA, et al., "Secondary prevention of macrovascular events in patients with type 2 diabetes in the PROactive Study (PROspective pioglitAzone Clinical Trial In macroVascular Events): a randomised controlled trial," *Lancet*, vol. 366, no. 9493, pp. 1279-1289, 2005.

[38] Duckworth W, Abraira C, Moritz T, Reda D, Emanuele N, Reaven PD, et al., "Glucose control and vascular complications in veterans with type 2 diabetes," *New England Journal of Medicine*, vol. 360, no. 2, pp. 129-139, 2009.

[39] The ADVANCE Collaborative Group, "Intensive blood glucose control and vascular outcomes in patients with type 2 diabetes," *New England Journal of Medicine*, vol. 358, no. 24, pp. 2560-2572, 2008.

[40] The Heart Outcomes Prevention Evaluation Study Investigators, "Effects of ramipril on cardiovascular and microvascular outcomes in people with diabetes mellitus: results of the HOPE study and the MICROHOPE substudy," *Lancet*, vol. 355, pp. 253-259, 2000.

[41] UK Prospective Diabetes Study (UKPDS) Group, "Intensive blood-glucose control with sulphonylureas or insulin compared with conventional treatment and risk of complications in patients with type 2 diabetes (UKPDS 33)," *Lancet*, vol. 352, pp. 837-852, 1998.

[42] Sever PS, Dahlöf B, Poulter NR, Wedel H, Beevers G, Caulfield M, et al., "Prevention of coronary and stroke events with atorvastatin in hypertensive patients who have average or lower-than-average cholesterol concentrations, in the Anglo-Scandinavian Cardiac Outcomes Trial--Lipid Lowering Arm (ASCOT-LLA)," *The Lancet*, vol. 361, no. 9364, pp. 1149-1158, April 2003.

[43] Diabetes Prevention Program Research Group, "Reduction in the incidence of type 2 diabetes with lifestyle intervention or metformin," vol. 356, pp. 393-402, 2002.

[44] Edelstein SL, Knowler WC, Bain RP, et al., "Predictors of progression from impaired glucose tolerance to NIDDM: an analysis of six prospective studies," vol. 46, pp. 701-710, 1997.

[45] Archimedes, Inc. Description of the Archimedes Model. [Online]. www.archimedesmodel.com

[46] Samuel S, Peskin B, Arondekar B, Alperin P, Johnson S, Blumenfeld I, Stone G, Jacobson TA, "Estimating health and economic benefits from using prescription omega-3 fatty acids in patients with severe hypertriglyceridemia," *American Journal of Cardiology*, vol. 108, no. 5, pp. 691-697, 2011.

[47] Peskin, BR., Shcheprov, AV, Boye, KS, Bruce, S, Maggs, DG, and Gaebler, JA, "Cardiovascular

© 2011-2012

outcomes associated with a new once-weekly GLP-1 receptor agonist vs. traditional therapies for type 2 diabetes: a simulation analysis," *Diabetes, Obesity and Metabolism*, vol. 13, no. 10, pp. 921–927, 2011.

ARCHIMEDES
... a KAISER PERMANENTE Innovation
123 Mission Street, 11th floor
San Francisco, CA 94105, USA
www.archimedesmodel.com
info@archimedesmodel.com
+1 415 490 0400

© 2011-2012
